# Transcriptome dynamic of Arabidopsis roots infected with *Phytophthora parasitica* identifies *VQ29*, a gene induced during the penetration and involved in the restriction of infection

**DOI:** 10.1371/journal.pone.0190341

**Published:** 2017-12-27

**Authors:** Jo-Yanne Le Berre, Mathieu Gourgues, Birgit Samans, Harald Keller, Franck Panabières, Agnes Attard

**Affiliations:** 1 INRA, Université Côte d'Azur, CNRS, ISA, France; 2 Department of Plant Breeding, Institute of Agronomy and Plant Breeding, Giessen, Germany; Agriculture and Agri-Food Canada, CANADA

## Abstract

Little is known about the responses of plant roots to filamentous pathogens, particularly to oomycetes. To assess the molecular dialog established between the host and the pathogen during early stages of infection, we investigated the overall changes in gene expression in *A*. *thaliana* roots challenged with *P*. *parasitica*. We analyzed various infection stages, from penetration and establishment of the interaction to the switch from biotrophy to necrotrophy.

We identified 3390 genes for which expression was modulated during the infection. The *A*. *thaliana* transcriptome displays a dynamic response to *P*. *parasitica* infection, from penetration onwards. Some genes were specifically coregulated during penetration and biotrophic growth of the pathogen. Many of these genes have functions relating to primary metabolism, plant growth, and defense responses. In addition, many genes encoding VQ motif-containing proteins were found to be upregulated in plant roots, early in infection. Inactivation of *VQ29* gene significantly increased susceptibility to *P*. *parasitica* during the late stages of infection. This finding suggests that the gene contributes to restricting oomycete development within plant tissues. Furthermore, the *vq29* mutant phenotype was not associated with an impairment of plant defenses involving SA-, JA-, and ET-dependent signaling pathways, camalexin biosynthesis, or PTI signaling. Collectively, the data presented here thus show that infection triggers a specific genetic program in roots, beginning as soon as the pathogen penetrates the first cells.

## Introduction

Plant organs are continually exposed to pathogenic microorganisms such as bacteria, fungi, and oomycetes. In most cases, such exposure does not result in disease, as plants have preformed defenses and immune responses that are activated by pathogen recognition [1]. In leaves, immune responses are activated by the recognition of pathogen-associated molecular patterns (PAMPs), small molecular motifs conserved within a class of microbes, or by the recognition of proteins (effectors) secreted by the pathogen into the apoplastic space or targeted to the host cell cytoplasm [1–3]. Early responses to pathogen perception include cytoskeletal reorganization, cell wall reinforcement, and the generation of reactive oxygen species, whereas late responses include the production of pathogenesis-related (PR) proteins and localized programmed cell death (PCD), to limit pathogen spread [1,4–7]. The defense responses are triggered and controlled by a crosstalk between signaling pathways involving phytohormones, such as salicylic acid (SA), ethylene (ET) or jasmonic acid (JA) [8,9]. Exceptions exist, but SA is generally thought to control PAMP-triggered immunity (PTI) and effector-triggered immunity (ETI) to biotrohic pathogens, whereas ET and JA regulate defense responses to necrotrophs. ET and JA have antagonistic effects on SA-mediated signalling pathway [10,11].

By contrast to the well documented responses of aerial plant organs to pathogen attack, we know little about root responses to infection, mostly because the process of root infection is difficult to handle experimentally [12–14]. Experimental systems have been developed to bridge this gap and to provide us with a better understanding of the responses of roots to biotic stress. These systems have revealed similarities between leaf and root responses, but have also revealed major differences. Whole-genome variation studies have shown differences in global genetic programs between roots and shoots during pathogen invasion [15–20]. For example, most of the genes activated in beech roots infected with *Phytophthora citrocola* had no known function or do not match database sequences for genes activated in the aerial parts of plants [15]. In *A*. *thaliana* roots, most of the genes found to be differentially expressed following infection with the fungus *Fusarium oxysporum* were less strongly expressed than in leaves inoculated with the same fungus and showed tissue-specific regulation [17,20]. Moreover, the behavior of the interaction may differ between different types of infected organ [12,21]. For instance, the infection of maize with the fungus *Colletotrichum graminicola* leads to the expression of defense genes in both roots and shoots. However, roots respond more rapidly and accumulate larger amounts of defense-related hormones, despite displaying slower disease development [16]. The differences in the responses of roots and shoots may result from differences in the signal perception and transduction mechanisms contributing to PTI and ETI. In rice roots, PTI-related genes are rapidly but transiently induced during early stages of infection, whereas the corresponding transcripts continue to accumulate in leaves throughout pathogen invasion [16]. In *Arabidopsis* leaves, the *RPP1* resistance gene confers ETI to *Hyaloperonospora arabidopsidis* (*Hpa*) strains carrying the corresponding *Avr* gene. *RPP1* is also expressed in roots, but it does not confer ETI to *Hpa* in this organ [22]. The most frequently reported differences in immune responses between roots and shoots concern the signaling pathways mediated by SA, ET and JA. In *F*. *oxysporum*-infected *Arabidopsis*, the defense-related genes encoding PLANT DEFENSIN 1.2 (PDF1.2a and PDF12b), PATHOGENESIS RELATED 4 (PR4), and other JA-associated proteins are strongly induced in leaves, but not in roots [17]. Following the inoculation of *A*. *thaliana* roots with the fungus *F*. *oxysporum* or the oomycete *Phytophthora parasitica*, ethylene (ET) appears to be the predominant defense hormone, with SA and JA playing only marginal roles [18,23]. The organ specificity of immune responses was clearly demonstrated in experiments in which *A*. *thaliana* roots and shoots were treated with the defense-inducing molecules SA and JA, and the PAMPs Flg22, peptidoglycan and chitin [24,25]. Furthermore, benzothiadiazole (BTH), a synthetic inducer of systemic acquired resistance, has been shown to protect rice leaves, but not roots against *M*. *grisea* invasion [26].

Global transcript profiling is a powerful approach for describing host plant responses to infection and for identifying the genes and pathways responsible for containing disease. However, such approaches focus on responses to invasive pathogen growth, and earlier stages of infection have only recently been considered [9,16,20,27–33]. The host genes and genetic programs activated in the plant early in the penetration process remain poorly documented, despite the key role of this process in determining the outcome of infection.

With this study, we aimed at increasing our knowledge of the responses triggered in plant roots during the very early stages of infection. Many devastating diseases of crops are caused by soilborne oomycete species [34–36]. *Phytophthora* species, in particular, have a major ecological and economic impact, causing annual losses estimated at 5 billion dollars [34–37]. *P*. *parasitica* is a typical root pathogen that can infect plants from more than 60 families [38]. We previously established a model pathosystem for the interaction of Arabidopsis and *P*. *parasitica* [18]. We used this system to perform a genome-wide analysis of the changes in the root transcriptome occurring during the onset of *P*. *parasitica* infection, to identify the principal functions underlying the responses of roots to oomycetes. We focused on selected key stages of *P*. *parasitica* development, including penetration, biotrophy, and the switch to necrotrophy. We present here our findings, characterizing infection stage-specific modulated genes, and identifying members of a gene family involved in the containment of disease in this organ.

## Materials and methods

### Plant material, growth conditions

The *A*. *thaliana* ecotype used in the study was N60000, and the mutants—N519428, N838800, N666741, N655201, N764496, N682939, N657520, N561438, N680896, N548279, N537796 and N559907—were all obtained from The European Arabidopsis Stock Centre, Nottingham (NASC), United Kingdom. For *in vitro* culture, *Arabidopsis* seeds were surface-sterilized and seeds were cold-stratified for two days and sown on 1 x Murashige and Skoog (MS) medium (Sigma Chemical Company, MO, USA) supplemented with 20 g l^-1^ sucrose (Prolabo), and 20 g l^-1^ agar. After 10 days, plants were transferred to square Petri dishes containing a 2 cm-wide strip of solid MS agar separating the root compartment (growing in 10 ml of 0.1 x MS medium) from a compartment without medium for the aerial parts of the plants. These dishes, each containing six plants, were then placed on end for 20 days, and incubated at 21°C under short-day conditions [18].

### Growth conditions for *P*. *parasitica* and inoculation of Arabidopsis plantlets

*P*. *parasitica* Dastur isolate 310 was initially isolated from tobacco in Australia, and was maintained in the *Phytophthora* collection at INRA, Sophia Antipolis, France. The conditions for *Phytophthora* growth and zoospore production were as previously described [39].

For studies of disease severity we added 500 of motile zoospores to the roots of the 30-days-old-plants obtained as described above. The plantlets were then further incubated at 21°C, as described above. Disease severity was recorded during the 20 days following infection and ranked from 1 (healthy plants) to 7 (dead plants) as previously described [18]. Disease development is presented during the 15 days following the inoculation considering that after this period the difference in phenotypes did not evolve anymore. Statistical analyses of disease severity were based on Scheirer–Ray–Hare nonparametric two-way analysis of variance (ANOVA) for ranked data (H test) [40]. The statistical analysis was carried out on 25 to 30 plants from each genotype, and all experiments were performed at least twice [18].

For studies of complemented lines, we used a rapid method adapted from previous work [19]. *Arabidopsis* seeds were surface sterilized and deposited in petri diches on a 34g m–2 sterile mesh (garden protection film) placed on top of 10ml of 0.5x MS medium. Plates were incubated at 25°C with 12h daily illumination. After 12 days, 500 motile zoospores of *P*. *parasitica* were added to the medium. Plates were incubated in the same conditions as above and invasion progression was scored 2 and 4 days after infection (2dai and 4dai) when the symptoms were already established, visible and progressed. The surface of green area of each plantlet was quantified upon image acquisition using ImageJ software [41]. Quantification of disease progression was achieved by measuring the ratio of green leaf area between 2dai and 4dai. One-way ANOVA with Bonferroni’s Multiple Comparison test identified lines, which differed significantly in terms of number of green pixels negatively correlating with the progress of disease. The statistical analysis was carried out on 35–50 plants from each genotype, and all experiments were performed at least twice [18].

### Quantification of oomycete biomass in roots by quantitative PCR

Roots of 21-days-old plants were inoculated with *P*. *parasitica* (10^6^ zoospores ml^-1^) and harvested 6 hours after infection. Genomic DNA was extracted from roots according to Ewards *et al*., 1991 [42]. DNA served as template for quantitative PCR (qPCR) analyses by using 10ng of DNA and SYBR Green, according to the manufacturer’s instructions (Eurogentec SA, Seraing, Belgium). Fungal colonization was determined by the 2^−ΔCt^ method [43] by subtracting the cycle threshold (Ct) values of *P*. *parasitica UBS* and *WS21* genes (*UBC*, CK859493, genes encoding ubiquitin conjugating enzyme; *WS21*, CF891675 gene encoding the 40S ribosomal protein S3A; [44]; S1 Table), from those of *A*. *thaliana*, *NADH* and *OXA1* genes (*NADH*, gene encoding a mitochondrial NADH-ubiquinone oxidoreductase subunit; *OXA1*, gene encoding a mitochondrial inner membrane translocase; S1 Table). Data were analyzed by Student’s t-test considering significant difference for p-values <0.05.

### Arabidopsis transformation

A transcriptional fusion was obtained by introducing the 2 kb sequence upstream from the *VQ29* (At4g37710) gene (Pro*VQ29*:GFP) into the Gateway vector pK2GWFS7 from Ghent University (http://gateway.psb.ugent.be/vector/show/pKGWFS7/search/index/transcriptional_reporters/any). The resulting construct was introduced into the *A*. *thaliana* N60000 ecotype by *Agrobacterium*-mediated transformation (*A*. *tumefaciens* strain GV3101), as previously described. Two independent transgenic lines (Pro*AT4G37710*:GFP#1 and Pro*AT4G37710*:GFP#5) were analyzed. Plants were self-crossed and homozygous progenies were selected on the basis of the segregation of the kanamycin resistance marker.

A complemented line of the *vq29* mutant was obtained by introducing the 1,542-bp promoter and the 372-bp coding sequence of *VQ29* into the Gateway vector pH7m24GW from Ghent University. The resulting construct was introduced into the *A*. *thaliana* N561438 (*vq29*) by Agrobacterium-mediated transformation (*A*. *tumefaciens* strain GV3101), as previously described. The transgenic lines, Comp1 and *Comp2* were analyzed. Plants were selfed and homozygous progenies were selected on the basis of the segregation of the hygromycin resistance marker.

### Microscopy

For the observation of early infection steps, we added 10^6^ of motile zoospores to the MS medium in Petri dishes containing 12-days-old plantlets grown as described above [18]. Image acquisition were performed on the Microscopy Platform-Sophia Agrobiotech Institut- INRA 1355-UNS-CNRS 7254-INRA PACA-Sophia Antipolis. Confocal laser scanning microscopy images were obtained with a Zeiss LSM 510 META confocal microscope (Carl Zeiss GmbH, Jena, Germany). For GFP visualization, an argon laser was used for excitation at 488 nm.

### RNA recovery

Total RNA was extracted from infected roots as previously described [45]. Total RNA was treated with DNAse I (Ambion, Austin, USA), and 1 μg was reverse-transcribed with the I Script cDNA synthesis kit according to the manufacturer’s instructions (Biorad, Hercules, USA).

### RT-quantitative PCR

Reverse transcription-quantitative polymerase chain reaction (RT-qPCR) experiments were performed with 5 μl of a 1:50 dilution of first-strand cDNA and SYBR Green, according to the manufacturer’s instructions (Eurogentec SA, Seraing, Belgium). Gene-specific oligonucleotides (S1 Table) were designed with Primer3 software (http://frodo.wi.mit.edu), and their specificity was checked by analyzing dissociation curves after each run. Genes encoding a mitochondrial NADH-ubiquinone oxidoreductase subunit (AT5G11770) and a mitochondrial inner membrane translocase (AT5G62050) were selected as constitutive internal controls [46]. For microarray validation, RNA was isolated from non-inoculated roots (NI), and from roots 2.5 hours after inoculation (hai), 6 hai, 10.5 hai and 30 hai with *P*. *parasitica*. Two biological replicates of the entire experiment were performed, each as a technical triplicate. For each time point, six results were analyzed. Gene expression was quantified and normalized with respect to constitutively expressed internal controls [18].

For *VQ29* and hormonal pathway expression analysis for mutant validation, RNA was isolated from non-inoculated roots (NI), and from roots 6 hours after inoculation. Three biological replicates of each time points were performed, each as a technical triplicate. Analysis was performed as above.

### Array hybridization and analysis

In two independent experiments, roots from the ecotype N60000 were inoculated with water or with *P*. *parasitica* to establish a compatible interaction. Total RNA was extracted as described above, and cDNA synthesis, sample labeling, hybridization procedures and data acquisition were performed at the NASC microarray platform [47]. The dataset is available from the GEO database at the NCBI under accession number GPL198. The transcriptome statistical analysis was performed as previously described [30]. After quality control with the Bioconductor package “simpleaffy” (Crisipn Miller), the cel-files were quantile-normalized with the “gcrma” package of Bioconductor [48]. Then, a quality control filter was performed. If the log2 ratios for the two time series differed by more than 75% of the mean of the two log2 ratios, the gene concerned was removed from subsequent analyses. Each of the remaining genes was tested for significant up- or downregulation by ANOVA analysis of variance and *p-value* correction by false discovery rate (FDR) [49]. Genes with adjusted *p-value* <0.05 and an absolute fold-change of 2 or more were considered to be differentially expressed. For clustering, the data were first mean-centered and log-2-transformed with Epclust (http://www.bioinf.ebc.ee/EP/EP/EPCLUST, [49]. Hierarchical clustering (Pierson correlations, mean linkage) and k-mean clustering (default parameters) were performed with Genesis software (http://genome.tugraz.at/genesisclient/genesisclient_description.shtml). For all cluster analyses, we used Virtual plant 1.3 programs to assess the overrepresentation of terms from the MIPS Functional Catalogue Database (FunCatDB, http://virtualplant.bio.nyu.edu/cgi-bin/vpweb/ [50,51]. Finally, we used the GENEVESTIGATOR online platform for the global analysis of publicly available expression data for *Arabidopsis* exposed to biotic stresses, PAMP and hormone treatments [52]. We selected as candidate genes, from the genes displaying a modulation of expression in our array analysis, those not deregulated in response to all biotic stresses, PAMP and hormone treatments.

## Results

### The root transcriptome of *Arabidopsis* upon infection with *P*. *parasitica*

We analyzed the transcriptional changes occurring in *Arabidopsis* roots during the first hours of infection with *P*. *parasitica*, using samples from time-course experiments corresponding to the previously characterized key stages of pathogen establishment [18]. Arabidopsis roots were collected 2.5 hours after inoculation (hai, during the penetration of the first cell by *P*. *parasitica*), 6 hai (when a few cortical cells are colonized), 10.5 hai (when *P*. *parasitica* grows along the stele and abundant haustoria are forming, reminiscent of the biotrophic phase) and 30 hai (during the switch from biotrophy to necrotrophy (S1 Fig) [18]. Two independent RNA samples were obtained from plants at each stage of the interaction or from non-inoculated plants, for the analysis of gene expression profiles. We used the Affymetrix ATH1 array to ensure that each condition can be analyzed independently and ensure that our data could be compared with other experiments of gene expression profiles established under various biological conditions with this system.

**Fig 1:**
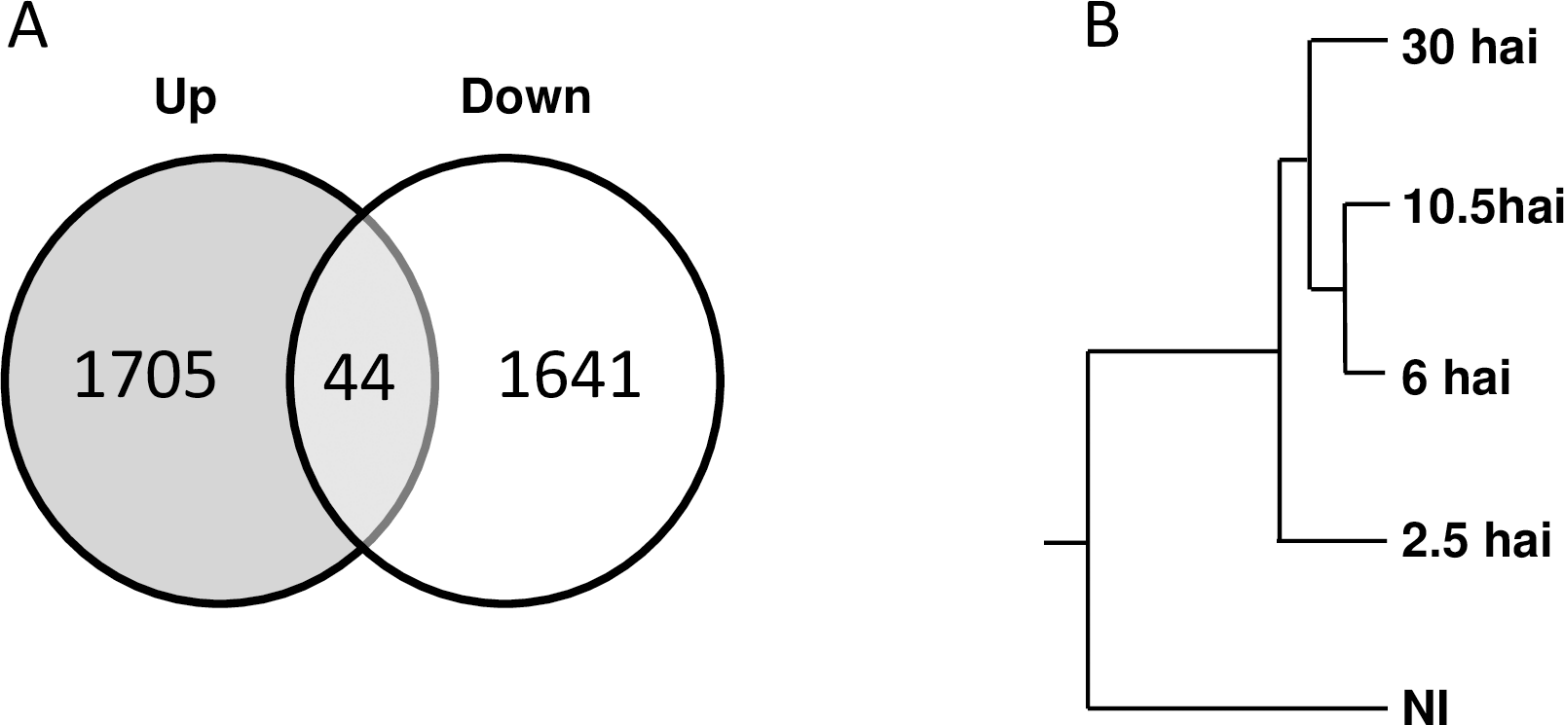
Global transcriptional changes during the infection of *Arabidopsis thaliana* roots with *Phytophthora parasitica*. (A) Number of *P*. *parasitica*-responsive genes in *A*. *thaliana* roots. The numbers of differentially expressed genes, upregulated and downregulated following the inoculation of roots with *P*. *parasitica* are displayed in a Venn diagram. (B) Hierarchical clustering of time-course transcription data according to the conditions. The conditions analyzed were: (NI) transcripts of non-infected roots; (2.5-hai) transcripts of roots 2.5 hours after infection (hai) with *P*. *parasitica*; (6-hai) transcripts of roots 6 hai; (10.5-hai) transcripts of roots collected 10.5 hai; (30-hai) transcripts of roots collected 30 hai.

Of the 22,746 genes represented on the *A*. *thaliana* ATH1 GeneChip, 17,974 (79%) were expressed in at least one of the five sets of biological conditions analyzed. Among these, 1680 genes were found to be differentially expressed at 2.5 hai, 2,477 at 6 hai, 2,589 at 10.5 hai and 2,456 at 30-hai (S2 Table). Overall, 3390 genes displayed a more than two-fold difference in expression with respect to non-inoculated roots for at least one of the infection stages considered, among which 1,749 genes were upregulated and 1,685 downregulated (Fig 1A). Hierarchical clustering was performed on biological conditions, to describe the different expression patterns (Fig 1B). These data revealed the activation of a genetic program in the host during penetration with *P*. *parasitica* (2.5 hai), which is different from all other infection conditions tested (Fig 1B). Moreover, the genetic programs triggered at 6 and 10.5 hai (considered together) were different from that operating at 30 hai (Fig 1B).

### Clusters of co-expressed genes in *Arabidopsis* roots infected with *P*. *parasitica*

**Fig 2:**
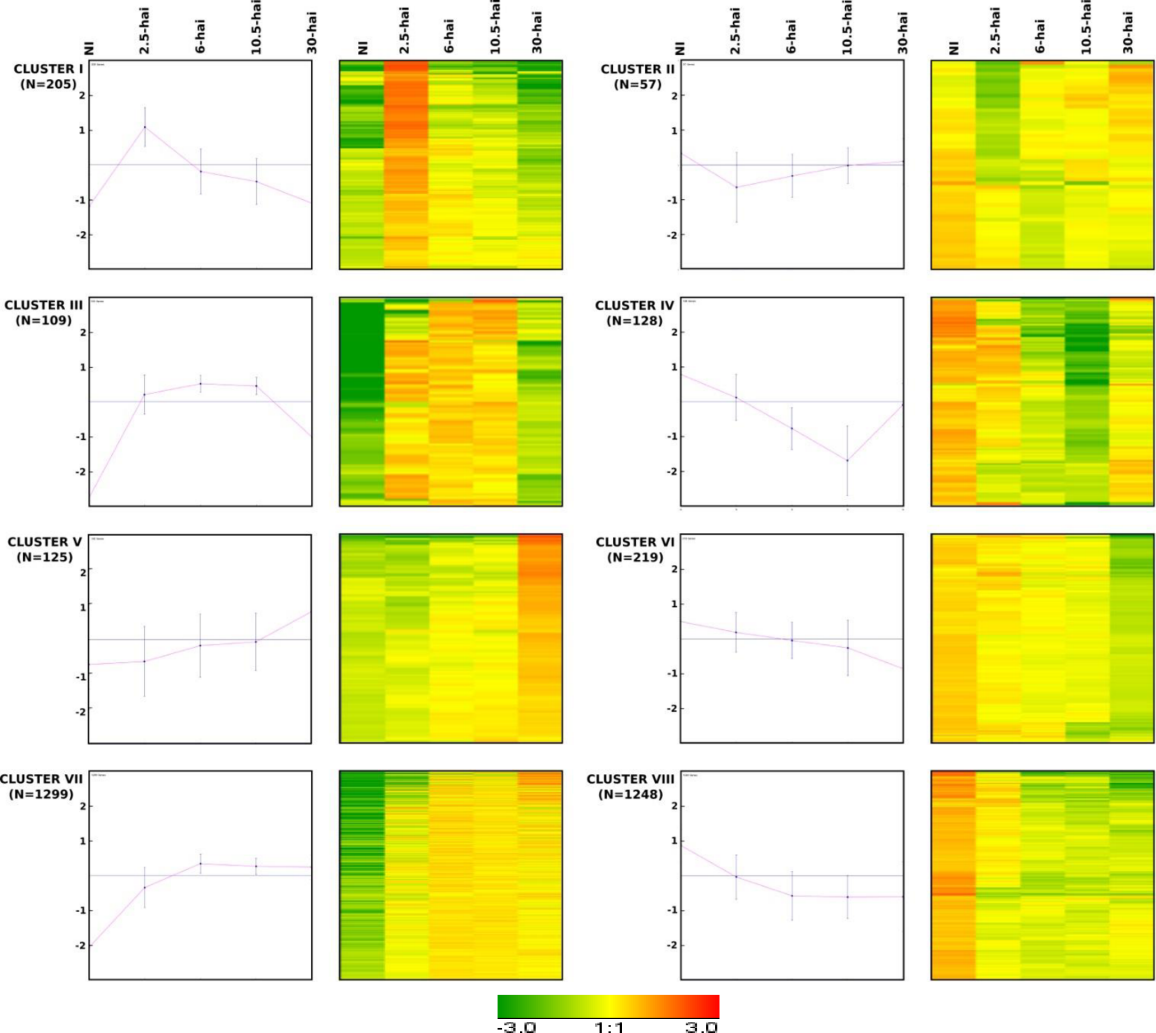
K-mean clustering and corresponding heat-map of the 3390 genes differentially expressed during the infection of *Arabidopsis* roots. *A*. *thaliana* roots were inoculated with *Phytophthora parasitica* and a time-course analysis was performed for five sets of conditions: (NI) transcripts of non-inoculated roots; (2.5-hai) transcripts of roots 2.5 hours after infection; (6-hai) transcripts of roots 6 hours after infection; (10.5-hai) transcripts of roots collected 10.5 hours after infection and (30-hai) transcripts of roots 30 hours after inoculation. K-mean clustering identified 8 clusters. For each cluster is indicated, left, k-mean cluster, right, heat maps. Red indicates upregulation and green indicates downregulation. Clusters I, III, V and VII correspond to upregulated genes. Clusters II, IV, VI and VIII correspond to downregulated genes.

We further analyzed the patterns of expression of the 3,390 genes displaying a more than two-fold modulation by carrying out K-mean clustering. The principal patterns were grouped together into eight major clusters (Fig 2, S3 Table). Four clusters contained genes with expression transiently modulated in infected roots (Clusters I, II, III and IV, Fig 2). Clusters I and II corresponded to genes transiently up- and downregulated, respectively, during penetration (2.5 hai, 262 genes, 7.7% of genes displaying a modulation of expression, Fig 2). Clusters III and IV grouped together genes transiently up- and downregulated, respectively, during the biotropic development of the oomycete, when root cells were still alive (237 genes, 7%, Fig 2). The genes of clusters I to IV were thus regulated during the first 10.5 hours, but not during the switch to necrotrophy. Two clusters, V and VI, grouped a set of genes that were up- or down-modulated, respectively, during the early switch to necrotrophy (30-hai, 344 genes, 10.1%, Fig 2). Some genes from clusters V and VI were slightly up- or downregulated respectively, 6-hai or 10-hai, but all modulations were less than two-fold with respect to control conditions. In these clusters, the only absolute FC > 2 was obtained at 30 hai. Finally, the two major clusters, clusters VII and VIII, contained genes with expression up- and downregulated, respectively, during infection from 2.5 hai or 6 hai to 30 hai (2547 genes, 75.1%, Fig 2).

### Validation of microarray data by RT-quantitative PCR

For the confirmation of microarray data, we performed reverse transcription-quantitative PCR (RT-qPCR) analyses of expression for the seven genes of each cluster displaying the strongest modulation (56 genes; S2 Fig, S4 Table). RNA samples independent of those used for microarray hybridization were generated and analyzed. In total, 45 of the 56 genes assessed (80%) displayed a change in expression during the onset of the interaction, consistent with the results of microarray hybridization (S4 Table). The expression patterns of genes from clusters I, III, IV, V, VI, VII and VIII were validated in 100%, 100%, 100%, 85.7%, 57.1%, 100% and 57.1% of cases, respectively (S4 Table). A lack of agreement was found only for the smallest cluster, cluster II (57 genes), for which agreement rates did not exceed 28.5%, probably due to technical limitations. These results demonstrate the reliability of our microarray data for most clusters. The expression profiles detected in all but cluster II by the microarray analysis reflect real modulation of gene expression. The cluster can thus be used to draw hypotheses on the genetic program occurring during the first hours of infection.

### Principal functions governed by transiently modulated genes in *P*. *parasitica*-infected *Arabidopsis* roots

We investigated the principal functions involved in early root responses to infection with *P*. *parasitica*, by identifying the cellular and molecular functions overrepresented among those triggered by the transiently regulated genes from clusters I, III and IV, and by the generally up and downregulated genes of clusters VII and VIII, respectively (S5 and S6 Tables). This analysis was performed with MIPS Functional Catalogue Database (FunCatDB) terminology [50,51]. Validation by RT-qPCR of cluster VIII can be considered as sub-optimal. We nevertheless analyzed it and results must be considered with caution.

The regulation of primary metabolism, such as amino-acid metabolism, carbon metabolism and polysaccharide metabolism, was significantly modulated during the colonization of *Arabidopsis* roots by *P*. *parasitica* (clusters IV, VII, and VIII, S6 Table). Genes involved in phosphate metabolism and the biosynthesis of secondary metabolites were significantly overrepresented among those transiently upregulated during penetration (cluster I) and those upregulated during infection (such as camalexin biosynthesis, cluster VII; S6 and S7 Tables). A larger number of genes than expected by chance alone were transiently upregulated during penetration (cluster I) and specifically encoded enzymes involved in energy generation (e.g. ATP synthase or respiration, S6 Table). The cluster of generally down regulated genes (cluster VIII) contained a significantly larger than expected number of genes involved in the generation of energy (such as glycolysis, S6 Table). Genes involved in the metabolism of lipids were significantly overrepresented among the genes transiently downregulated during biotrophy (cluster IV) and generally downregulated throughout the entire infection process (cluster VIII).

Root development appeared to be altered by *P*. *parasitica*, because genes involved in cell growth and morphogenesis (S6 Table) were down regulated during infection (cluster VIII. S5 Table). For instance, the *A*. *thaliana* genome contains 31 genes encoding expansins, which are involved in cell wall loosening during plant cell growth, cell wall disassembly and cell separation [53,54]. In our array data, four of these genes displayed a modulation of expression during infection, with downregulation observed for all of them (cluster VIII, S7 Table).

Many genes involved in the perception of stimuli and the resulting responses displayed a significant modulation of expression during infection. Genes corresponding to the MIPS FunCatDB terms “cellular communication”, “response to biotic stimulus”, or “plant defense responses”, were overrepresented among the genes generally upregulated during the infection process (cluster VII, S6 and S7 Tables). These overrepresented genes included genes encoding defense-related proteins, transcription factors of the WRKY family, and cell death-related proteins. Several enzymes involved in the detoxification of reactive oxygen species were found to be up- or downregulated during infection and transiently upregulated during penetration (clusters VII, VIII and I respectively, S6 Table). Several genes encoding defense-related proteins, such as *FRK1* and *WRKY11*, were transiently upregulated from the start of penetration (cluster I, S6 and S7 Tables). Most of the genes transiently downregulated during biotrophy (cluster IV) encoded genes involved in perception to stimuli and in resulting responses (S6 and S7 Tables).

### Imbalance in defense hormone homeostasis during root infection

The induction of defenses against pathogens is dependent on crosstalk between several signaling pathways, such as those regulated by the phytohormones SA, JA and ET. We analyzed our microarray data, to identify modulations of these pathways. Active SA may be generated by *de novo* biosynthesis, or by remobilization from its stored forms, SA 2-*O*-β-D-glucoside (SAG), SA glucose ester (SGE), methyl salicylate (MeSA), and methyl salicylate 2-*O*-β-D-glucose. We analyzed the regulation of genes involved in SA synthesis and homeostasis, and we found that only four of these genes displayed a modulation of expression during infection (S7 Table). A gene encoding isochorismate synthase, which is involved in SA synthesis, *ICS2*, was downregulated from penetration onwards and then no transcripts were detectable for this gene (cluster VIII). The gene *ICS1* gene, encoding another isoform of this enzyme, was not expressed during the first six hours of infection, and was only weakly induced 10 and 30 hours after infection, during the switch to necrotrophy (cluster VII, S7 Table). *MES9*, a gene encoding a methylesterase catalyzing the conversion of MeSA to SA, is downregulated by *P*. *parasitica* and turned off 6 hai (cluster IV, S7 Table). Finally, *UGT74F2*, which glucosylates SA to generate SAG, is transiently overexpressed during penetration (cluster I, S7 Table). These data suggest that the production of active, unconjugated SA is coordinately repressed, as soon as *P*. *parasitica* penetrates the roots. These findings are supported by the lack of expression of three marker genes for SA signaling, *PR1*, *PR2*, and *PR5*, in infected roots (S7 Table).

Expression of some genes coding enzymes involved in JA biosynthesis is modulated (S7 Table). The expression of allene oxide cyclase genes (*AOCs*) and the jasmonic acid resistant 1 gene (*JAR1*) was downregulated, whereas genes encoding acyl-CoA oxidase 1 (ACX1), oxophytodienoate reductase 3 (OCPR3), allene oxide synthase (AOS), and OCP CoA ligase 1 (*OCPC1*) were upregulated (S7 Table). In addition, *MYC2* and *ERF1* encode transcription factors induced by JA and ET; the expression of *MYC2* was unaffected by infection, whereas that of *ERF1* was upregulated. By contrast, seven of the 12 *A*. *thaliana jasmonate-ZIM-domain protein* genes (*JAZ*), encoding proteins shown to impair MYC2 function, were upregulated (cluster VII; *JAZ1*, *JAZ2*, *JAZ5*, *JAZ6*, *JAZ7*, *JAZ10*- cluster III; *JAZ8* [55,56].

1-aminocyclopropane-1-carboxylic acid oxidase genes *(ACO4* and *ACO1)* and ACC synthase genes *(ACS2*, *ACS6* and *ACS7)* encoding proteins involved in ET biosynthesis, and *ERF1*, *PR3* and *PR4* [57], which are induced by treatment with JA or ET, were all upregulated during infection (cluster VII, S7 Table). Surprisingly, no transcripts of *PDF1*.*2*, another gene activated by treatment with ET or JA, were detected (S7 Table).

### *VQ29* encodes a VQ motif domain-containing protein involved in restricting *P*. *parasitica* infection

In order to validate the role of the genes modulated during early infection in the outcome of the interaction, we performed a functional analysis of a set of genes. We then focused on genes strongly induced upon infection, either transiently during penetration (cluster I), or continuously, throughout the establishment of infection (cluster VII). Candidate genes were selected on the basis of several criteria, including their fold-change expression upon infection (from 2.2 to 1587). We focused principally on uncharacterized genes, to identify new functions, omitting genes identified with GENEVESTIGATOR software to be activated nonspecifically by foliar biotic stress or by hormone applications, and PAMP or elicitor treatments. We adopted this approach to avoid the identification of well characterized PTI or hormone signaling pathways. Ten candidate genes met our criteria (Table 1, S8 Table). The corresponding knockout (ko) mutants were obtained and we assessed the response of homozygous lines to *P*. *parasitica* infection. Seven of the 10 lines analyzed responded to *P*. *parasitica* similarly to the wild type (Table 1, S3 Fig). The other three mutants (N519428, N666741 and *vq29*) were more susceptible to *P*. *parasitica*. These mutants corresponded to 2 genes (At2g44370 and At5g40590) encoding for members of the DC1 domain-containing family, and one gene (*VQ29*) encoding a member of the VQ motif-containing family.

**Fig 3:**
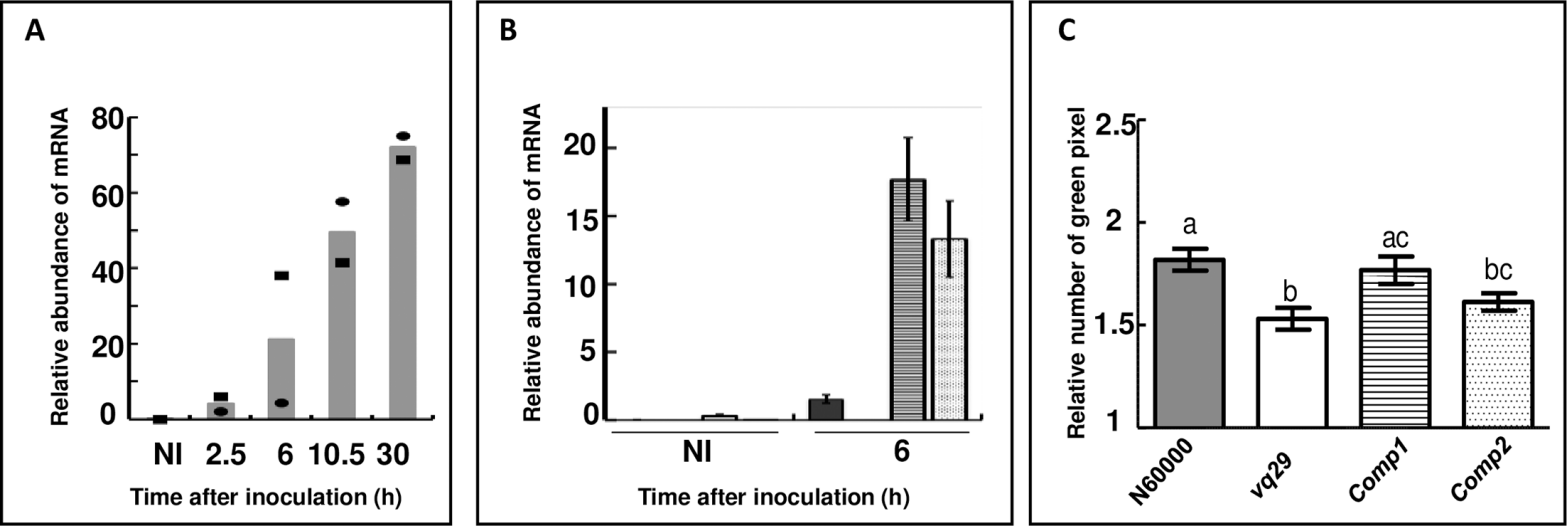
Expression profile of *VQ29* in wild type, *vq29* and *vq29* complemented lines infected with *Phytophtora parasitica* and response of *vq29* and *vq29* complemented lines to *P*. *parasitica*. (A) Accumulation of *VQ29* transcripts during the time course of infection. RNA was isolated from non-inoculated roots (NI), and from roots 2.5 hours after inoculation (hai), 6 hai, 10.5 hai and 30 hai with *P*. *parasitica*. RT-qPCR data presented as the mean relative transcript abundance values. For each time point, the RT-qPCR values for the 2 independent replicates are indicated as dark dots and scares. (B) Accumulation of *VQ29* transcripts during infection of N60000, *vq29* and *vq29* complemented lines (*Comp1* and *Comp2*). RNA was isolated from non-inoculated roots (NI), and from roots 6hai. Data are presented as the mean relative transcript abundance values, and the mean SE of 3 independent biological replicates. Transcript levels are normalized with respect to the expression of the At5g11770 and At5g62050 genes determined for the same samples. (C) Susceptibility of *vq29* and *vq29* complemented lines (*comp1* and *comp2*) to *P*. *parasitica*. Twelve days old plantlets were inoculated with 500 zoospores from *P*. *parasitica* 310 strain. Quantification of disease was achieved by measuring ratio of green leaf area between 2 days after infection (dai) and 4dai when the symptoms were already visible. One-way ANOVA test identified lines which differed significantly in terms of response to *P*. *parasitica* (n = 60–70 plants from each genotype). A different letter above the columns indicate significant differences (p-value<0.05).

*Vq29* mutant was found to be particularly more susceptible to infection (Table 1, S3 Fig). Furthermore, the *VQ29* gene showed the largest fold-change in expression on infection (FC = 1587, Table 1, S7 Table). We therefore carried out a more detailed analysis for this gene. *VQ29* was upregulated from the start of penetration, and its level of expression increased steadily during infection (cluster VII, Fig 3A, S4 Table). To confirm that the *vq29* disease phenotype is caused by the associated T-DNA insertion, *vq29* mutant was complemented with genomic *VQ29* under control of *VQ29* promoter. Two independent homozygous T3 lines were recovered, *Comp1* and *Comp2* (Fig 3B). These lines showed an overexpression of *VQ29* in roots and were subsequently inoculated with *P*. *parasitica* strain 310. Construct *Comp1* was able to complement *vq29* mutant and Comp2 showed an intermediated complemented phenotype (Fig 3C). *VQ29* was thus considered to be involved in limiting the root invasion by pathogens.

**Table 1:** Infection assays for knockout (ko) lines for candidate genes selected from the array data.

Cluster	Ko-lines NASC ID ^a^	Gene AGI No ^b^	Gene description	fold change	Infection essay phenotypes	Infection assay p-value
**I**	**N519428**	**AT2G44370**	**DC1 domain-containing protein**	**40,6**	**more susceptible than WT**	**1.6E-11**
	N838800	AT5G43520	DC1 domain-containing protein	20,1	WT	0.53
	**N666741**	**At5G40590**	**DC1 domain-containing protein**	**18,7**	**more susceptible than WT**	**6.1E-27**
	N655201/N655172	AT5G11920	ATCWINV6 (6-&1-FRUCTAN EXOHYDROLASE)	14	WT	0.16
	N674496	AT1G29020	calcium-binding EF hand family protein	10,7	WT	0.18
	N682939	AT1G11540	unknown	7,9	WT	0.83
	N657520	AT2G46600	calcium-binding protein, putative	2,2	WT	0.89
**VII**	**N561438**	**AT4G37710**	**VQ29; VQ motif-containing protein**	**1587**	**more susceptible than WT**	**2.9E-05**
	N680896	AT5G06730	peroxidase, putative	111,4	WT	0.64
	N548279	AT4G10520	Subtilase family protein	100,7	WT	0.36

a Lines ID number obtained from NASC catalog.

b AGI number of inactivated genes

### In roots, *VQ29* transcription is induced by *P*. *parasitica* penetration

The *A*. *thaliana* genome contains 34 genes encoding proteins with a VQ motif (S7 Table) [58]. The expression of 18 of these genes (53%) was upregulated during infection (S7 Table) and the expression of eight of these genes was induced by *P*. *parasitica*, because no transcript of these genes was detected in non-inoculated roots (S7 Table). The genes of this group were therefore mostly upregulated during infection with *P*. *parasitica* (S7 Table).

**Fig 4:**
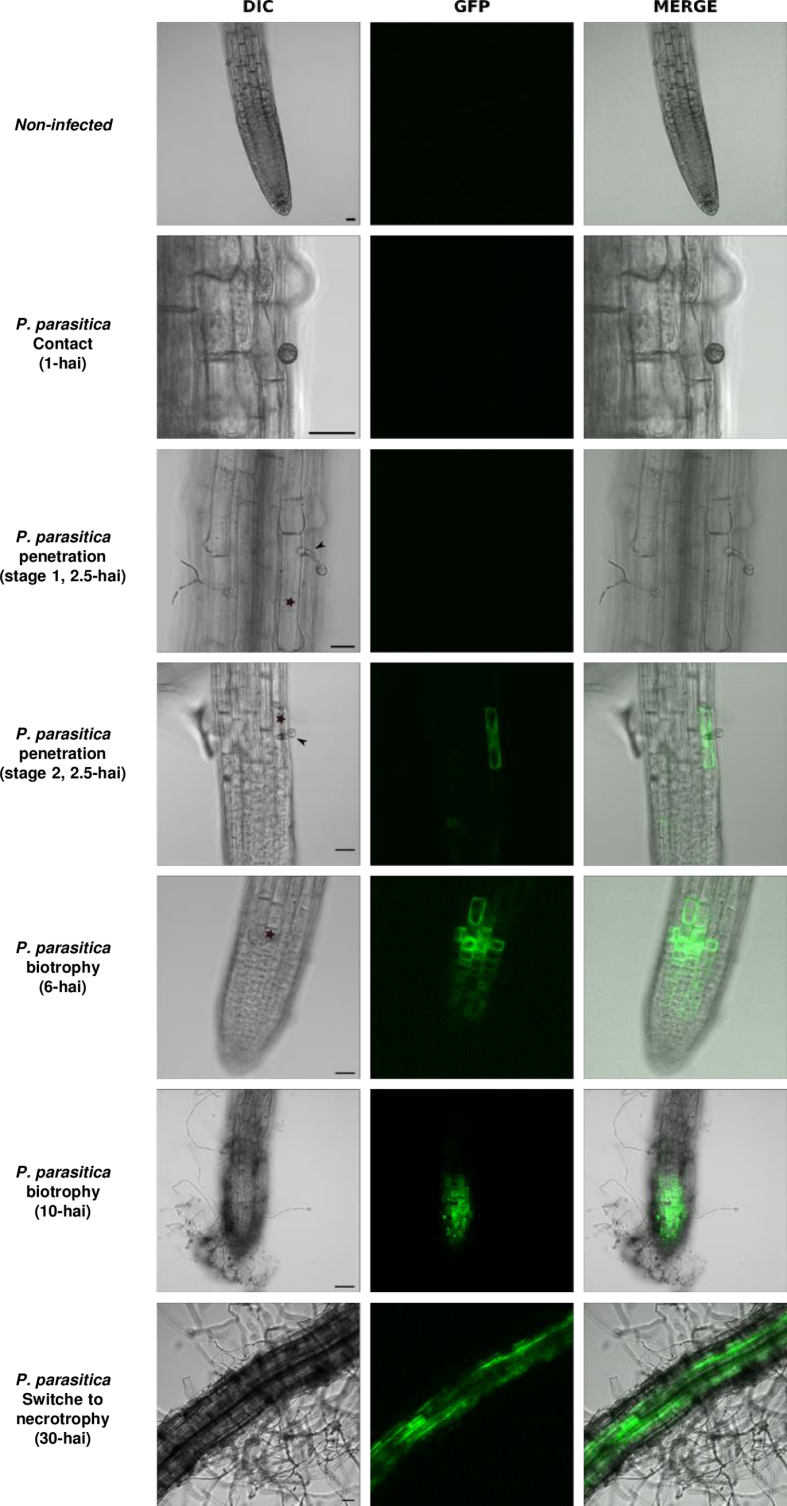
Pattern of *VQ29* expression in *Arabidopsis thaliana* roots infected with *Phytophthora parasitica*. Confocal image of N60000 expressing the *ProVQ29*:*GFP* transgene. Two independent lines (#1 and #5) were analyzed and gave similar results. Only the results for *ProVQ29*:*GFP* #1 are presented here. (GFP) *ProVQ29*:*GFP* expression, (DIC) differential interference contrast, and (MERGE) merged images are shown. Roots were infected with 1x10^6^ zoospores of *P*. *parasitica* and GFP expression was followed for 30 hours after infection. No GFP was detected in non-infected roots. Bars, 20 μm. Arrows indicate appressoria, and stars indicate penetration points.

We generated reporter lines expressing *GFP*, encoding green fluorescent protein, under the control of the *VQ29* promoter. Two individual transgenic lines in the N60000 background (Pro*VQ29*:GFP#1 and Pro*VQ29*:GFP#5; Fig 4) were analyzed for GFP accumulation during root infection with *P*. *parasitica*. Consistent with our transcriptome data, GFP fluorescence was not detected in roots before infection (Fig 4). Following inoculation with *P*. *parasitica* zoospores, GFP was not detected in host cells in contact with encysted zoospores, or in the cells supporting appressorium differentiation or, even, very early during penetration by *P*. *parasitica* (Fig 4). This stage of penetration, during which GFP was not detected, is referred to here as stage 1. GFP then began to accumulate while the pathogen was still in the first cell. At this stage (stage 2), fluorescence was detected only in the cell in contact with the infection hyphae (Fig 4). As *P*. *parasitica* grew across the cortex, GFP fluorescence was detected in the cells surrounding the initial point of infection (Fig 4). GFP accumulation subsequently spread, with an increasing number of cells displaying detectable fluorescence (Fig 4).

### *VQ29* does not interfere with the early infection stages of *P*. *parasitica*

Since *VQ29* is induced by penetration and limits the development of *P*. *parasitica* in roots, we further analyzed whether increased disease symptoms on *vq29* mutant coincide with increase oomycete colonization at early stage of infection. We thus evaluated the growth rate of *P*. *parasitica* in *vq29* and wild-type roots, 6 hours after infection (S4 Fig). *P*. *parasitica* was found to grow similarly in v*q29* and N60000 roots early during infection, indicating that VQ29 does not interfere with disease development at this early stage.

### *VQ29-*related defense is not associated with SA-, JA-, or ET- mediated signaling

**Fig 5:**
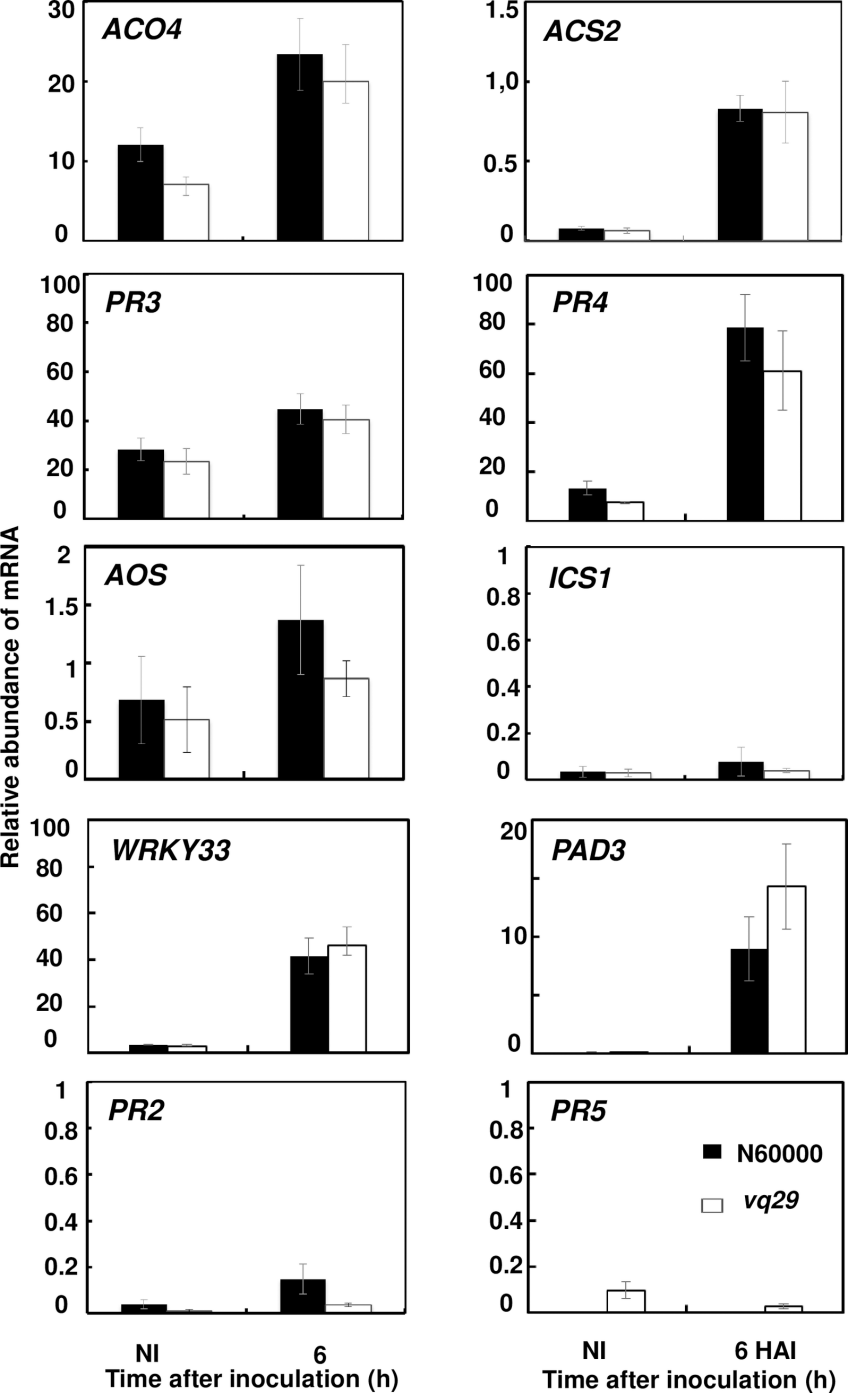
Defense-related gene expression in *vq29* mutant *Arabidopsis thaliana* roots infected with *Phytophthora parasitica*. Analysis of defense-related marker genes expression in roots from wild-type and *vq29* mutant plants. RNA was isolated from non-inoculated roots (NI) and from roots 6 hours after infection (6 hai). RT-qPCR data are presented as relative transcript abundance for genes: *ACS2* and *ACC*, two markers for the ET biosynthesis; *AOS*, a marker for the JA biosynthesis; *PR4* and *PR3*, two marker genes for the ET and JA signaling pathways; *ICS1*, a marker for the SA biosynthesis; *PR2* and *PR5*, a marker gene for the SA-mediated signaling pathway; *WRKY33*, involved in the PTI pathway and *PAD3*, involved in Camalexin biosynthesis. *FAD8*, a marker for the JA biosynthesis, *PFD1*.*2*, a marker gene for the ET and JA signaling pathways and, *PR1*, a marker for SA signaling pathways are not represented as they were not detectable. Transcript levels were normalized with respect to those for AT5G11770 and AT5G62050, determined for the same samples. No significant difference was observed between N60000 and *vq29* marker gene transcripts abundance (T-test on biological replicates). The means, with error bars (2 standard errors), of 3 independent replicates are indicated. Ni, non-inoculated roots. Dark bar, N60000; White bar, *vq29*.

To determine whether *VQ29* restricts *P*. *parasitica* development in roots through known defense signaling pathways, we performed RT-qPCR experiments. The expression profile of marker genes that are associated with plant SA-, JA-, ET-, and PTI-mediated defense signaling, as well as with Camalexin biosynthesis were compared between *vq29* mutant and wild type plants (Fig 5). We followed JA- and ET-mediated signaling events by studying *ACO4*, a gene encoding 1-aminocyclopropane-1-carboxylate oxidase and *ACS2* encoding 1-amino-cyclopropane-1-carboxylase synthase 2, both enzymes involved in ET biosynthesis, and *AOS* and *FAD8*, genes encoding allene oxide synthase and fatty acid desaturase 8, respectively, both participating in the JA biosynthetic pathway [59–61]. PR3, PR4 and PDF1.2 are downstream markers of ET- and JA-mediated signaling pathways [62–64]. The marker genes studied for SA-mediated signaling were *ICS1*, encoding the isochorismate synthase involved in SA biosynthesis, and PR1, PR2 and *PR5*, downstream markers of SA signaling [65–68]. Finally, we followed PTI-mediated defense signaling and camalexin biosynthesis, by studying the expression of *WRK33* and *PAD3*. The transcripts of *PR1*, *FAD8* and *PDF1*.*2* were not detectable, and those of *ICS1*, *PR2* and *PR5* were weakly detectable in non-inoculated and inoculated roots (Fig 5). By contrast, transcripts of *ACS2*, *WRKY33*, *ACO4*, *PR4*, AOS and *PAD3* accumulated 6 hours after infection but their abundance was not significantly different between infected *vq29* and wild-type plants (Fig 5).

## Discussion

Much is known about the plant defense mechanisms occurring in leaves, but little is known about the genetic basis of root responses to soilborne pathogens. This study was designed to increase our knowledge about early root responses to oomycetes. We carried out a genome-wide analysis of gene expression, to describe root responses during the onset of the compatible interaction between Arabidopsis and *P*. *parasitica*. We found that 7.3% to 10.7% of the genome was differentially regulated with respect to non-inoculated roots, at specific stages of the interaction. This is consistent with the proportions reported for other genome-wide expression profiling studies of *A*. *thaliana* interactions [27,30,32,69,70].

We found that a distinct set of genes was associated with the first contact and penetration by the pathogen (2.5 hai). Furthermore, the biotrophic phase of the interaction (6 hai and 10.5 hai) triggered global modulations of the transcriptome, different from those observed during the switch to necrotrophy (30 hai). Overall, 14.7% of *A*. *thaliana* genes modulated were transiently expressed during the very early stages of infection with *P*. *parasitica* (clusters I, II, III and IV). This study is the first to report global changes of the root transcriptome during the onset of infection with a soilborne oomycete pathogen, covering all important stages (from penetration to the biotrophy-necrotrophy switch) determining disease outcome. Only a few other studies have described the host transcriptome at particular steps in the infection process. The analyses of *M*. *oryzae*-infected rice roots or *Verticillium longisporum*-infected Arabidopsis roots confirm that penetration triggers distinctive genetic reprogramming of the host cell [16,71]. Conversely, more recently, *Nicotiana benthamiana* roots infected with the oomycete *P*. *palmivora* showed steady responses of the plant transcriptome with no genes transiently expressed during early infection [72]. This work did not include very early stage such as penetration [72]. Taken together, the penetration-associated program of gene expression we highlighted may indicate initiation of the counterattack by plant cells in response to infection, or the pathogen driven early modulation of plant transcriptome, which determines the outcome of the interaction. One can suppose that the small part of genes transiently modulated could represent a tight response adapted to particular infection, whereas the gene up- or downregulated during the infection process could reflect more general responses.

An analysis of the overrepresented functions, based on the genes identified in the clusters provided insight into the various genetic programs activated during successive stages of infection. There was a large overlap between clusters in the functions overrepresented, but several features were highlighted.

First, genes involved in the production of energy through ATP biosynthesis were transiently upregulated during penetration (cluster I). By contrast, genes involved in energy generation, such as glycolysis, or involved in lipid and fatty acid metabolism were gradually downregulated during the course of the infection (clusters VIII). An association between the regulation of energy production and responses to biotic stress has already been reported for leaves [73,74]. It is generally thought that processes involved in energy production are upregulated during infection, whereas those associated with assimilatory processes are downregulated, to favor plant defenses [73]. Our results go against this hypothesis, because energy production through glycolysis appeared to be downregulated in infected roots, whereas ATP synthase genes were upregulated during penetration. Glycolysis contributes to ATP production through glucose consumption. Plant defenses to pathogen infection are commonly fuelled by increases in the amount of glucose [75]. However, the pathogen can exploit this response to satisfy its own metabolic requirements [76]. Plant root cells may limit glycolysis to avoid the highjacking of their intermediates by the pathogen, with ATP production for defense purposes instead being ensured by direct synthesis via a stimulated ATP synthase.

Our data also showed that cell fate functions were downregulated during infection (cluster VIII). Such downregulation was observed, for genes involved in cell growth and morphogenesis, such as the *Arabidopsis* expansin genes. It has been suggested that the cessation of cell growth in cotton hypocotyls infected with *F*. *oxysporum* is a global response to stress rather a specific response to pathogen infection [77]. Nevertheless, inactivation of the expansin-like A2 gene has been reported to result in the limited invasion of *Arabidopsis* leaves by the fungal necrotroph, *Botrytis cinerea* [78]. Conversely, pathogens such as *P*. *parasitica* transiently express cell wall-degrading enzymes, thereby favoring penetration [79]. Cell wall loosening thus appears to favor the penetration of plant cells by *P*. *parasitica* and the development of this pathogen within roots. Decreasing the amount of expansins in the cell wall may therefore constitute a defense response in *Arabidopsis* roots, leading to a strengthening of the cell wall to limit colonization.

We also found that genes encoding defense-related proteins, including enzymes involved in camalexin biosynthesis, were upregulated during *Arabidopsis* root infection (clusters VII). This finding is in line with data obtained from *V*. *longisporum*-infected Arabidopsis roots and suggests the contribution of phytoalexin biosynthesis during infection, as a basal defense against *P*. *parasitica* [71,80]. Cluster I includes genes encoding defense-related proteins such as MEK1 and FRK1, proteins involved in early defense signaling [81]. Our observations suggest that essential mechanisms of PTI are transiently upregulated during the penetration of *Arabidopsis* roots, as reported for rice roots penetrated by *M*. *oryzae* [16]. The subsequent downregulation observed suggests that hemibiotrophs suppress this defense before the onset of invasive growth. *WRKY11* was transiently upregulated upon penetration. WRKY11 is a negative regulator of PTI [82], and its activation may contribute to the observed subsequent downregulation of immune responses during invasive growth.

Plant defense is dependent on crosstalk between signaling pathways regulated principally by the phytohormones, SA, JA and ET. We found that penetration of the root by *P*. *parasitica* activated several mechanisms decreasing the availability of active SA. Surprisingly, WRKY38, a transcription factor upregulated by SA in leaves and that reduces PR1 expression, appeared weakly induced in roots during penetration [83]. Nevertheless these finding are consistent with the lack of expression of three marker genes for SA signaling, *PR1*, *PR2*, and *PR5*, in infected roots. We observed no clear change in the expression of genes involved in JA biosynthesis, but the higher abundance of transcripts encoding various JAZ repressors indicates that penetration by *P*. *parasitica* downregulates the expression of JA target genes. By contrast, we found that ET-mediated pathways and the downstream transcription factor gene *ERF1* were activated from the start of penetration and that the expression of ET-responsive genes steadily increased during infection. Studies of root defense responses to filamentous pathogens have shown that not all pathogens activate the same signaling pathways [18,29,84–90]. Nevertheless, ET- and JA-dependent defenses seem to be frequently triggered in infected roots.

*P*. *parasitica* has also been reported to colonize *A*. *thaliana* leaves, and a cDNA library was generated from infected leaves. Nineteen genes were identified as significantly upregulated in infected leaves [29]. Eleven of these genes were also upregulated during infection in our array data (cluster VII), two were downregulated (cluster VIII) and six displayed no modulation of expression. Our findings highlight the importance of analyzing root responses to pathogens and demonstrate that leaves are not appropriate study models for investigating interactions occurring underground.

We therefore evaluated the involvement of several genes strongly upregulated during infection. We analyzed a set of 10 genes displaying strong upregulation either transiently during *P*. *parasitica* penetration, or throughout infection. Functional analyses of these genes revealed that knockout mutants for three genes (At2g44370, At5g40590 and *VQ29*) were significantly more susceptible to *P*. *parasitica* infection than wild-type plants. The two genes, At2g44370 and At5g40590, encode members of the DC1 domain-containing family. The *A*. *thaliana* genome encodes 132 DC1 proteins. Changes to the accumulation of 14 of these proteins were observed during infection, with 8 of these proteins displaying a transient increase in accumulation during penetration (S7 Table). Only few studies have investigated the involvement of DC1 proteins in responses to biotic stresses, and these studies focused on responses in aerial organs [91,92]. Our results indicate that DC1 genes, different from those identified in leaves, could be involved in plant response in underground plant-microbe interactions, with a possible role in the control of responses to penetration.

We further analyzed *VQ29*, a gene encoding a VQ motif-containing protein, *VQ29*. Proteins of this class share a conserved FxxxVQxxTG motif (VQ motif) of unknown function. Previous studies demonstrate that members from the VQ family of *Arabidopsis* proteins that show no functionally characterized domain structures, but plays a major role in growth regulation, plant development, and responses to biotic stress [58,93,94]. *In planta* analyses of *VQ29* expression confirmed that this gene was not transcribed in non-infected roots, and that transcriptional activation occurred during the various early steps of infection with *P*. *parasitica*. After penetration, *VQ29* expression was activated in the first rhizodermis cells penetrated by *P*. *parasitica*. The induction of *VQ29* expression in roots therefore appears to be controlled by a signaling event occurring immediately after the entry of the pathogen into the first cell. We think that this may define a switch in the plant response, so we divided penetration into two consecutive steps, characterized by the absence (step 1) or presence (step 2) of *VQ29* expression. We recently showed that *P*. *parasitica* expresses an effector repertoire from the penetration of the first root cells onwards [44]. It is possible that step 2 is triggered by effector secretion into the host cells. The activation of *VQ29* transcription may thus be one of the immediate early responses of plants, enabling them to cope with the cellular reprogramming events induced by effectors.

VQ29 was shown to bind to PHYTOCHROME INTERACTING FACTOR 3_LIKE1 (PIF1, AT2G46970), a key transcription factor involved in light signaling [95]. This interaction with PIF1 activates the expression of *XTR7*, a gene encoding XYLOGLUCAN ENDOTRANSGLYCOSYLASE 7 (AT4G14130), which is involved in the elongation of hypocotyl cells [95,96]. In our study, *VQ29* was not coregulated with *XTR7*, its expression instead being switched off as soon as penetration occurred. This is not consistent with the previously described coregulation of these two genes [95]. Hypocotyl elongation and root responses to *P*. *parasitica* infection thus appear to have different requirements for *VQ29*.

VQ proteins were described in higher plants only recently [58,93,97–99]. More than half of the 34 VQ-encoding genes identified in the *Arabidopsis* genome were upregulated in infected roots. Moreover, eight of these upregulated genes are clearly specific for infection, as they are not expressed in non-infected roots. Two genes are upregulated transiently during penetration by *P*. *parasitica*. These findings suggest that VQ motif-containing proteins play a specific role in controlling the plant response to *P*. *parasitica*.

The constitutive overexpression of *VQ20*, *VQ12* and *VQ29* has been shown to increase the susceptibility of *Arabidopsis* to *B*. *cinerea*, suggesting a role for the corresponding proteins in the downregulation of defense responses [58,94]. By contrast, VQ23, VQ16 and VQ21 bind and activate WRKY33, to induce camalexin biosynthesis, consistent with an upregulation of plant defenses by these VQ proteins [93,100–102]. Fourteen of the 34 VQ motif-containing proteins in *Arabidopsis* bind to different WRKY proteins [58]. This suggests that VQ motif-containing proteins are involved in fine-tuning responses to the biotic environment. The same study showed an absence of physical interaction between VQ29 and the WRKY transcription factors analyzed [58]. We found that late during infection, the *vq29* mutant was more susceptible to *P*. *parasitica* infection than the wild type. However, the overexpression of *VQ29* transcripts in complemented lines did not lead to increased resistance to *P*. *parasitica*. Moreover, the *vq29* mutant phenotype was not associated with an impairment of plant defenses, as marker genes for the SA-, JA-, and ET-dependent signaling pathways, camalexin biosynthesis and PTI signaling were not downregulated in the mutant with respect to the wild type (Fig 5). In contrast to previous findings showing that VQ29 downregulates basal defenses in leaves thus enhancing susceptibility of Arabidopsis to *B*. *cinerea* [94], we provide evidence that *VQ29* does not interfere with these defenses in roots. Although VQ29 interferes with infection of both roots and leaves, the protein appears to have different roles in these organs.

## Conclusions

In conclusion, we provide the first genomic data concerning the responses of root cells to the onset of infection with an oomycete pathogen. Our findings indicate that host gene expression is finely regulated during the first 30 hours of infection, with this regulation beginning with the first contact between the plant root and the pathogen. The findings furthermore indicate that the establishment of a compatible interaction with *P*. *parasitica* involves complex regulation of the host’s primary metabolism (including energy supply), and the mechanisms underlying growth and defense. Data analyses and functional studies led to the identification of the *VQ29* gene, which is required to restrict *P*. *parasitica* development in roots independently of defense activation. The data presented here thus show that infection triggers the modulation of specific gene sets in roots, beginning as soon as the pathogen penetrates the first cells. The corresponding genetic program differs from that in leaves, and its elucidation should help us to understand the interactions between the plant and microbes occurring underground, leading to the development of innovative crop protection strategies.

## Supporting information

S1 Fig Schematic representation of *P*. *parasitica* development stages in roots. We indicated the key stages of the infection as already described [18] and the sample recovered for the transcriptomic analysis.
(TIF)

S2 Fig Microarray data validation by reverse transcription-quantitative polymerase chain reaction (RT-qPCR). The RT-qPCR profiles and Affymetrix signals are given for one gene of each of the eight clusters identified from microarray data. RNA was isolated from non-inoculated roots (0), and from roots 2.5 hours after inoculation (hai), 6 hai, 10.5 hai and 30 hai with *P*. *parasitica*. For each gene represented, left is indicated the Affymetrix signal. Gray bars, mean normalized Affymetrix signals. Gray dots and scares, Affymetrix signals are indicated for the 2 independent replicates. Right, RT-qPCR profiles. Dark bars, RT-qPCR data presented as the mean relative transcript abundance values. For each time point, the RT-qPCR values for the 2 independent replicates are indicated as dark dots and scares.
(TIF)

S3 Fig Disease progression on 10 *A*. *thaliana* mutants inoculated with *P*. *parasitica*. Mutant and wild-type plants were inoculated with *P*. *parasitica* strain 310. Disease severity was recorded over time, with a disease index ranging from 1 to 7. The illustrations show the results of a representative experiment. Differences between ecotypes upon inoculation with *P*. *parasitica* were statistically significant, as determined by Scheirer–Ray–Hare nonparametric 2-way analysis of variance (ANOVA) for ranked data (H<0.05). Significant difference with respect to wild type ecotype N60000 was observed for N519428, N666741 and *vq29* mutants.
(TIF)

S4 Fig Colonization of *vq29* roots by *P*. *parasitica*. Twenty-one-days-old plants from the wild-type (N60000) and the *vq29* mutant were inoculated with *P*. *parasitica*, and oomycete biomass was determined by qPCR during biotrophic growth at 6-hai. Data are means from three independent experiments, and error bars represent the standard deviation. For each replicate, 50 plants from each line were analyzed. Data were analyzed by Student’s *t* test showing that differences between genotypes were not significant.
(TIF)

S1 Table Primers used in this study. (PDF)

S2 Table Number of *Arabidopsis thaliana* genes differentially expressed in roots infected with *Phytophtora parasitica*. Hai, hours after infection.
(PDF)

S3 Table List of genes from each cluster. The corresponding AGI for each probe are given. Probes are from ATH1 Affymetrix array.
(PDF)

S4 Table Validation of microarray data by RT-qPCR. The RT-qPCR profile and Affymetrix signal are given for each gene. NI, RNA isolated from non-inoculated roots. RNA isolated from roots 2.5 hai, 6 hai, 10.5 hai and 30 hai with *Phytophthora parasitica*. RT-qPCR data are presented as the value of the 2 independent replicates for each time point. Transcript levels were normalized with respect to those for At5g11770 and At5g62050 determined for the same samples. For normalized Affymetrix signals, the values of the 2 independent replicates are indicated. Hai, hours after infection.
(PDF)

S5 Table Representation of the principal terms within the MIPS functional catalogue database of *Arabidopsis thaliana* genes differentially expressed in roots infected with *Phytophthora parasitica* (Klatari et al., 2010, Virtual plant 1.3). Each term, is indicated in parenthesis the corresponding code number followed by the number of genes from Arabidopsis genome. Significant overrepresented of terms is indicated in bold.
(PDF)

S6 Table Terms within the MIPS Functional Catalogue Database (FunCatDB) overrepresented (p-value<0,5) in clusters I, III, IV, VII and VIII. Clusters were identified from microarray data GEO:GPL198. Clusters I, III and VII group genes transiently upregulated throughout infection, whereas clusters IV and VIII group genes downregulated. For each MIPS FunCatDB terminology, corresponding gene list and p-value are indicated. Highlighted classes are described in the manuscript.
(PDF)

S7 Table Microarray data for all the genes described. FC, Fold Change.
(PDF)

S8 Table Microarray data of genes selected for infection essay of knockout (Ko) lines and described Table 1. FC, Fold Change.
(PDF)
