# Supplementary figures and images for: Transcriptome dynamic of Arabidopsis roots infected with Phytophthora parasitica identifies VQ29, a gene induced during the penetration and involved in the restriction of infection
Source: PLoS One. 2017 Dec 27;12(12):e0190341. doi: 10.1371/journal.pone.0190341 (PMC5744986; doi:10.1371/journal.pone.0190341)

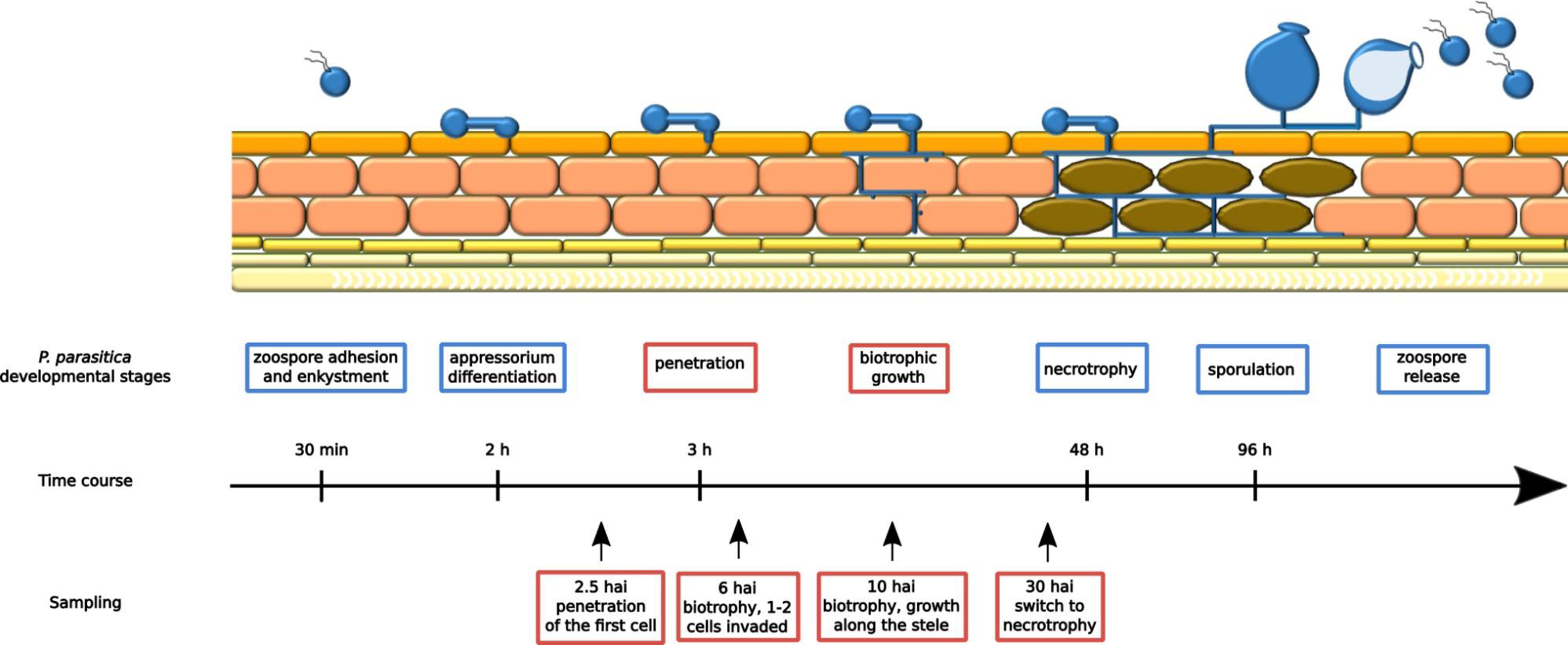

Supplement: S1 Fig — We indicated the key stages of the infection as already described [18] and the sample recovered for the transcriptomic analysis. (TIF) [file pone.0190341.s001.tif]

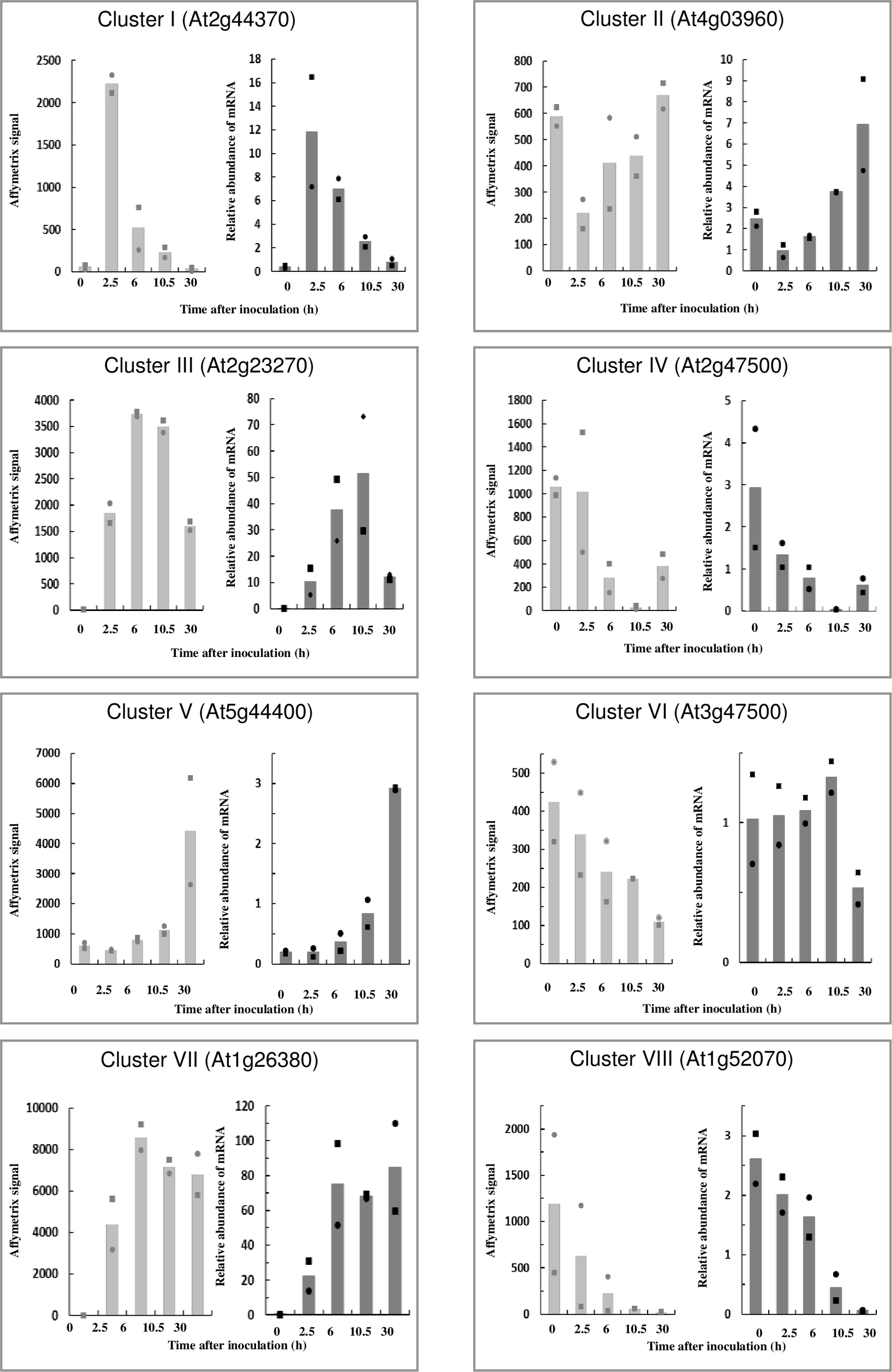

Supplement: S2 Fig — The RT-qPCR profiles and Affymetrix signals are given for one gene of each of the eight clusters identified from microarray data. RNA was isolated from non-inoculated roots (0), and from roots 2.5 hours after inoculation (hai), 6 hai, 10.5 hai and 30 hai with P. parasitica. For each gene represented, left is indicated the Affymetrix signal. Gray bars, mean normalized Affymetrix signals. Gray dots and scares, Affymetrix signals are indicated for the 2 independent replicates. Right, RT-qPCR profiles. Dark bars, RT-qPCR data presented as the mean relative transcript abundance values. For each time point, the RT-qPCR values for the 2 independent replicates are indicated as dark dots and scares. (TIF) [file pone.0190341.s002.tif]

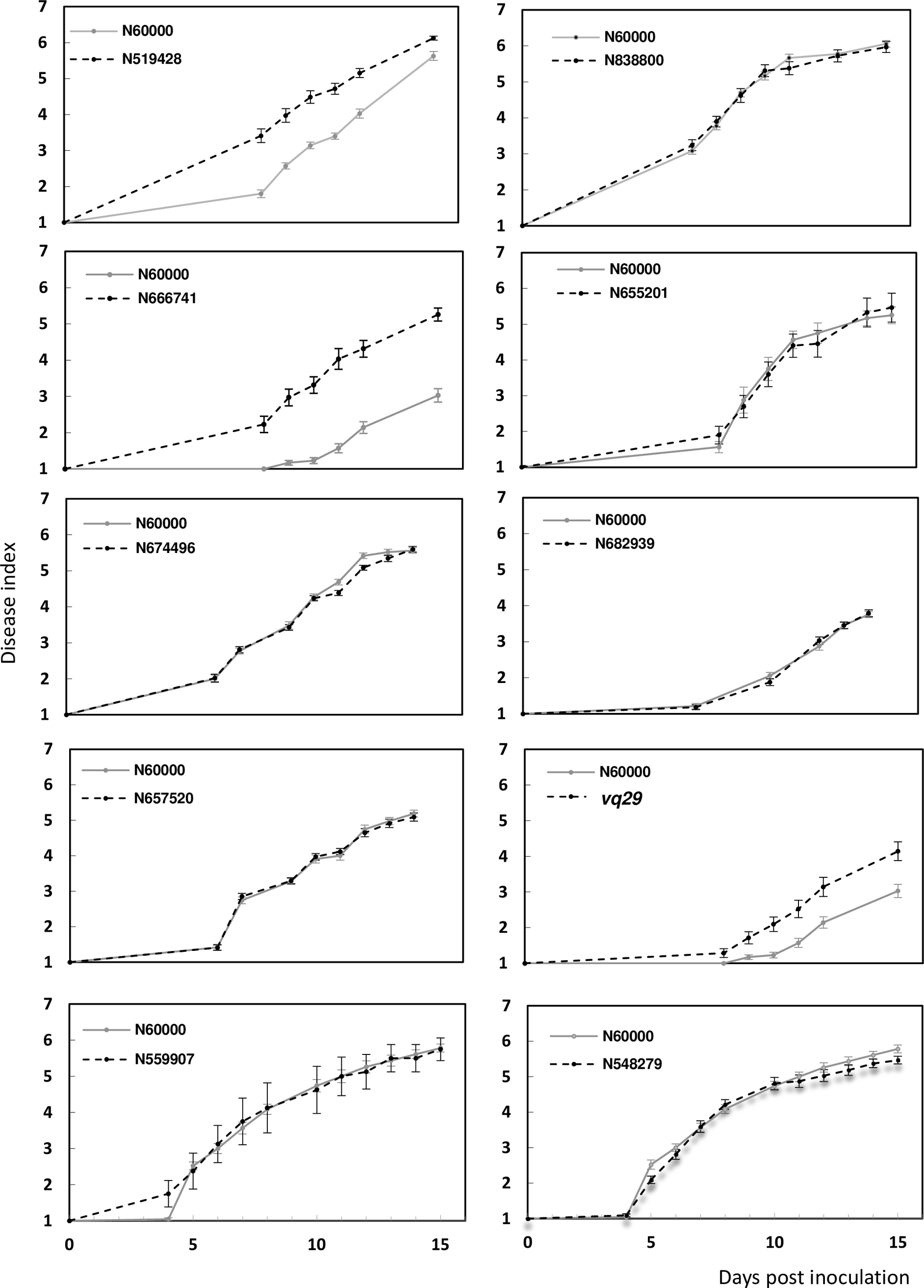

Supplement: S3 Fig — Mutant and wild-type plants were inoculated with P. parasitica strain 310. Disease severity was recorded over time, with a disease index ranging from 1 to 7. The illustrations show the results of a representative experiment. Differences between ecotypes upon inoculation with P. parasitica were statistically significant, as determined by Scheirer–Ray–Hare nonparametric 2-way analysis of variance (ANOVA) for ranked data (H<0.05). Significant difference with respect to wild type ecotype N60000 was observed for N519428, N666741 and vq29 mutants. (TIF) [file pone.0190341.s003.tif]

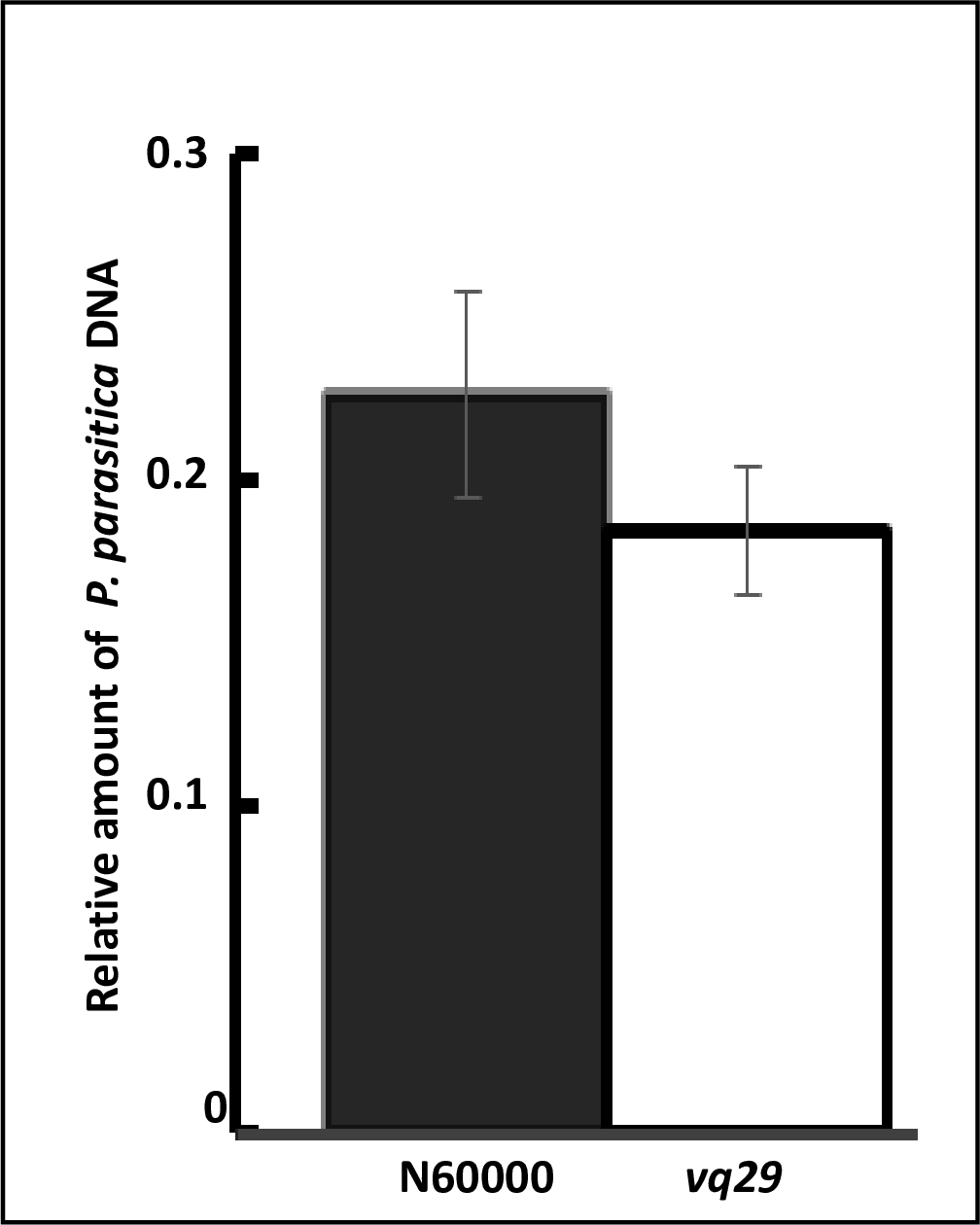

Supplement: S4 Fig — Twenty-one-days-old plants from the wild-type (N60000) and the vq29 mutant were inoculated with P. parasitica, and oomycete biomass was determined by qPCR during biotrophic growth at 6-hai. Data are means from three independent experiments, and error bars represent the standard deviation. For each replicate, 50 plants from each line were analyzed. Data were analyzed by Student’s t test showing that differences between genotypes were not significant. (TIF) [file pone.0190341.s004.tif]
