# Supplementary material for: Transcriptome dynamic of Arabidopsis roots infected with Phytophthora parasitica identifies VQ29, a gene induced during the penetration and involved in the restriction of infection
Source: PLoS One. 2017 Dec 27;12(12):e0190341. doi: 10.1371/journal.pone.0190341 (PMC5744986; doi:10.1371/journal.pone.0190341)
Supplement: S1 Table — (PDF) [file pone.0190341.s005.pdf]

S1 Table: Primers used in this study

Organism	Gene ID	Gene Name	Forward Primer (5'>3')	Reverse Primer (5'>3')
A. thaliana	AT1G02220	ANAC003	GGTGAGCCAAGCTCTAATAACC	CTCACCAATCTCAGTCCCTTG
A. thaliana	AT1G03850	GRXS13	ACATGGTCGTCGTTCTCAGTC	CGTTACCGTGGATGCTTCTTAC
A. thaliana	AT1G05880	ARI12	AGACAGCGTCGGCTTATTG	TGATATGGCTTGTCCAGCAG
A. thaliana	AT1G11540		AGCAACCACACATCGCATAG	AGCATTCCACCACCAATACC
A. thaliana	AT1G18570	MYB51	TCGGGTTCTCCACATTTCTG	ACGTCGTTTTCATCCTCGTG
A. thaliana	AT1G26380		CATCGTAGTGGCGAAAGATG	ACAAATGGCACAGACGACAC
A. thaliana	AT1G29020		GAGTCTTGATCCGCCAAATG	CCGATGATCAAGATGCAACC
A. thaliana	AT1G30135	JAZ8	CTCCAAACAAGTCGACCTCA	TATCGTCGTGAATGGTACGG
A. thaliana	AT1G30870		ACTTTCGCAGAGCAATCACC	GCAAACCTTCCTGATCTCTCC
A. thaliana	AT1G32640	MYC2	TGAATCACGCGAGTATGTCG	TTGCTCTGAGCTGTTCTTGC
A. thaliana	AT1G49600	RBP47A	TCAGGACTCAAACATGTACGC	GATCACTTTGATCACCCAACG
A. thaliana	AT1G52070		GCAAGATGGTGATTGGCTTC	TTAGAGCCATCATCCCAAGG
A. thaliana	AT1G70410	BCA4	GCTGTGAACGTATCGCTTG	CAGGAGTGGTCTTGAAATCG
A. thaliana	AT1G73220	oct-01	AGAGCTGTTCCCAACAAACG	GCACTGATGCAACACCAAAC
A. thaliana	AT1G75040	PR5	CGTACAGGCTGCAACTTTGA	TGAATTCAGCCAGAGTGACG
A. thaliana	AT1G80240	DGR1	ATCACTCTGGCTCGCTATGTG	AATGAGTCAACGACCCCAAC
A. thaliana	AT2G05070	LHCB2	AAGACGCCCACCTTTTGG	TTATGGCCACATCAGCTATCC
A. thaliana	AT2G20610	SUR1	TGCTTGAGGATGCACTTGAG	TCTTTTGGGCACACACATCC
A. thaliana	AT2G23270		GGACTGAGACGCTTGAATATGG	TTTCCGGAGCTAGACCAAAG
A. thaliana	AT2G26020	PDF1.2B	ACCAACAATGGTGGAAGCAC	CACTTGTGAGCTGGGAAGAC
A. thaliana	AT2G29470	GSTU3	CAATGGCCGAGAAAGAAGAG	AGTAGCAACGGGCTCTTGAC
A. thaliana	AT2G34590		GACACACCGGGTTTTGATTG	GCGTAAGGAGTTGGGACATC
A. thaliana	AT2G37430	ZAT11	AGAGTTTTGGGACCGGACAG	TGGATGGAATCATCGGAGAG
A. thaliana	AT2G38470	WRKY33	TCCAGTGAGGAAACATGTGG	TGAAGACGAATCCTGTGGTG
A. thaliana	AT2G39030	NATA1	CGGAAGAGTGGAGTGGATTG	CGATGGGTCTCATGCAAAT
A. thaliana	AT2G43510	ATTI1	CAAGATGCCGTGAGAACAAG	TGTGGAGTATCGTCGCAGAAG
A. thaliana	AT2G44370		CCGGATTCACTTACAACTGC	TCAAACCGTTCTGGTAAGGAG
A. thaliana	AT3G02240	RGF7	GGGCAACGAAAGTACAAACC	AACCATAATTTGCCCGGTATC
A. thaliana	AT3G02800	PFA-DSP3	GGATCCTCCAACTCCTATGC	CGCGTTCTTCTGGTACTCC
A. thaliana	AT3G04720	PR4	TAGTGGACCAATGCAGCAAC	GATCAATGGCCGAAACAAG
A. thaliana	AT3G07350		CCATCGGTTTCGATAATGC	GACACGTGGTCTTTTTGGTC
A. thaliana	AT3G20340		ACCGGTTCTACAGAGCATACC	GAAGCGTCATGTGTCCAAAC
A. thaliana	AT3G22840	ELIP	CAGACGCTGAGCTTTGGAAC	AACATCCTCCCCATAACGTG
A. thaliana	AT3G26650	GAPA	TAAAGCTGTGGCTCTTGTGC	AAAGCAGCGTTGACTTCCTC
A. thaliana	AT3G26830	PAD3	AACACAAGAACAGGGCAAGG	GGACGAATCCGTATTGGAGA
A. thaliana	AT3G47500	CDF3	TGCCTCCACCAGGTTTTTAC	TAGGCGATGAGGATTGATGC
A. thaliana	AT3G49620	DIN11	ATCGGAAAGGTCTTGGAAGG	TGATCCACCAAGGGCTAATG
A. thaliana	AT3G62680	PRP3	ATACCGTTTCTACCCGGACAAG	CACGATCAGTATTTGGGAGTGG
A. thaliana	AT4G03960	PFA-DSP4	GTACCACAATCGGAGAACTCG	ACGCAAAGACTGGAGAAAGC
A. thaliana	AT4G16660		GGATGGAGAAGATGCTAATGC	TGCTACTGGTCGTGCTGTAAG
A. thaliana	AT4G17870	PYR1	ATATGCCGGAAGGTAACTCG	TTTCACGTCACCTGAGAACC
A. thaliana	AT4G21620		ACTGGAGTTGGTGGTGGATAC	AGCTTCTTGCCGTTACAAGG
A. thaliana	AT4G22640		CAGTCAATGCCACCAATGC	GTATCGTGCTCGACGATTTG
A. thaliana	AT4G24780		GAGCAGCTAGCCAGAGGTTG	TGCTGATTGTGGCTTAGTGG
A. thaliana	AT4G31730	GDU1	AAGATGTGGCAACGTCAACC	TAAACCGCCGAAGAGGTAAG
A. thaliana	AT4G37710	VQ29	ATGCATCCTCATGTCTACCG	CTCTGCTTTGCTGCATCATC
A. thaliana	AT4G38470	STY46	AGGAGGGGGTATATTTGCAG	AAGGGGAGCAACACACAAAC
A. thaliana	AT5G02020	SIS	TCTGTGCGACAAGAGACTGTG	CTCAGATGACAGGGTTGTACTCTC
A. thaliana	AT5G07030		TCGGTTTGGCACGTGAAC	CTCTAAGACTGGTGAAGCAAACG
A. thaliana	AT5G11920	CWINV6	CGTTTCTGACCCTTTGCTTC	CTCTCCATTTCCCATCTTGC
A. thaliana	AT5G18030	SAUR21	TCACGATGTGTCGGAAAGG	CCGTTCCAGCTCCTATCATC
A. thaliana	AT5G18680	TLP11	GCGTCAGTGAAGAACTTTCAGC	CGGGTATCCATAATCCATCG
A. thaliana	AT5G25460	DGR2	TTGGGAAGACTTACGTGCTG	AGCTCGTTTAAACCCTCCTG
A. thaliana	AT5G40590		TTCACGACAATCGGTGAGG	CAACCGGAGCAGATGATTTC
A. thaliana	AT5G40990	GLIP1	AGGAGGGGAAGAAAGCATGT	AGCTCAGCGATCTGTCGATT
A. thaliana	AT5G43520		CGAAGAAGGAGAAGCGAGTTC	TCATCCCACAGATCAAGTGC
A. thaliana	AT5G44400		TCCTTACGGAGGAATGATGG	GATCCAGTTGATGTGTCTGCTC
A. thaliana	AT5G52310	COR78	GCCGAGAAACTTCAGATTGG	AATCCTCCCAACCATTCCTC
A. thaliana	AT5G53970	TAT7	GCCTGGTTTCCCAATCTATG	AGCAACCGTGTTTTCGTCTG
A. thaliana	AT5G56540	AGP14	AGTTCACCAGACGGTTGCAG	AGAATCCAAAGGCCATCACC
A. thaliana	AT5G60760		CCGGTGAATCTTCAGTTTGG	CAGCCATGTCAGCTTTACAGTC
A. thaliana	AT5G64170	LNK1	TAGACCCGAAGCAGAAGTTG	TCGCTTAGCCAAAATCGAG
A. thaliana	AT5G65040		CTCGATACACCCACGATCTATG	ATGGCAGAGACTTCGATTGC
A. thaliana	AT5G67480	ATBT4	CGAGATGGAAACTTCTGGTG	ACTCGCCCTTCTTGAGATTG
A. thaliana	AT1G05010	ACO4	GCTGATCGGAAAAGAAGCAG	CATTGTTGGCCACAGTTGTC
A. thaliana	AT1G01480	ACS2	TGTGTCTCCTGGCTCTTCCT	CCTCGTAAAGTCGTCGGAAA
A. thaliana	AT2G14610	PR1	CGGAGCTACGCAGAACAACT	CTCGCTAACCCACATGTTCA
A. thaliana	AT3G57260	PR2	TGGTGTCAGATTCCGGTACA	TCATCCCTGAACCTTCCTTG
A. thaliana	AT3G12500	PR3	CAATGCAACTGTCGTGGAAC	TGAGCAGTCATCCAGAACCA
A. thaliana	AT3G04720	PR4	TAGTGGACCAATGCAGCAAC	GATCAATGGCCGAAACAAG
A. thaliana	AT1G75040	PR5	CGTACAGGCTGCAACTTTGA	TGAATTCAGCCAGAGTGACG
A. thaliana	AT1G74710	ICS1	CAAATTGACCAGCAAATCG	CAAGGTCACGGAAGAAAACTG
A. thaliana	AT5G42650	AOS	ATGCCGTCAACGGAACTAAC	ACTCAGGGAAGATCCGGT TT
A. thaliana	At2G26020	PDF1,2b	ACCAACAATGGTGGAAGCAC	CACTTGTGAGCTGGGAAGAC
A. thaliana	AT5G05580	FAD8	CTTTGTCATGGGTCCAATCC	TGAGCCCTCCTCTCAGGTAA
A. thaliana	AT2G38470	WRKY33	TCCAGTGAGGAAACATGTGG	TGAAGACGAATCCTGTGGTG
A. thaliana	AT5G11770	OXA1	AACAGGACTCAGCGATGTTG	TACCTGATCTGCCTCCACCT
A. thaliana	AT5G62050	NADH	GAAGTTGTGCCAATGGAGGT	CCACCAATGCAAGAAATCCT
A. thaliana	AT3G26830	PAD3	AACACAAGAACAGGGCAAGG	GGACGAATCCGTATTGGAGA
P. parasitica	CK859493	UBC	CCACTTAGAGCCGCTAGGA	TACCGACTGTCCTTCGTTCA
P. parasitica	CF891675	WS21	CTCCAGAACGTGTACATCCG	TAGCGCCCTTCTCCTCAG
