# Supplementary material for: Transcriptome dynamic of Arabidopsis roots infected with Phytophthora parasitica identifies VQ29, a gene induced during the penetration and involved in the restriction of infection
Source: PLoS One. 2017 Dec 27;12(12):e0190341. doi: 10.1371/journal.pone.0190341 (PMC5744986; doi:10.1371/journal.pone.0190341)
Supplement: S2 Table — Hai, hours after infection. (PDF) [file pone.0190341.s006.pdf]

S2 Table. Number of *Arabodipsis thaliana* genes differentially expressed in roots infected with *Phytophtora parasitica.*
hai, hours after infection.

	2.5 hai	6 hai	10.5 hai	30 hai
**Number of up-regulated genes**	1137	1388	1376	1306
**Number of down-regulated genes**	543	1089	1213	1150
**Total**	**1680**	**2477**	**2589**	**2456**
