# Supplementary material for: Transcriptome dynamic of Arabidopsis roots infected with Phytophthora parasitica identifies VQ29, a gene induced during the penetration and involved in the restriction of infection
Source: PLoS One. 2017 Dec 27;12(12):e0190341. doi: 10.1371/journal.pone.0190341 (PMC5744986; doi:10.1371/journal.pone.0190341)
Supplement: S3 Table — The corresponding AGI for each probe are given. Probes are from ATH1 Affymetrix array. (PDF) [file pone.0190341.s007.pdf]

S3 Table: List of genes from each cluster.

The corresponding AGI for each probe are given. Probes are from ATH1 affymetrix array.

Cluster I		Cluster II		Cluster III		Cluster IV		Cluster V		Cluster VI		Cluster VII		Cluster VIII	
Probe	AGI	Probe	AGI	Probe	AGI	Probe	AGI	Probe	AGI	Probe	AGI	Probe	AGI	Probe	AGI
258947_at	AT3G01830	266950_at	AT2G18910	250149_at	AT5G14700	266155_at	At1g64950	264936_at	AT1G61140	261746_at	AT1G08380	247033_at	AT5G67250	248595_at	AT5G49230
258566_at	AT3G04110	250377_at	AT5G11560	258130_at	AT3G24510	266988_at	AT2G39310	250646_at	AT5G06720	257808_at	AT3G26935	245832_at	AT1G48850	247295_at	AT5G64180
265208_at	AT2G36690	266365_at	AT2G41220	261567_at	AT1G33055	261081_at	AT1G07350	267550_at	AT2G32800	245639_at	AT1G25260	245305_at	AT4G17215	247067_at	AT5G66950
259272_at	AT3G01290	262366_at	AT1G72890	259546_at	AT1G35350	259537_at	AT1G12370	258810_at	AT3G03970	266636_at	AT2G35370	245156_at	AT5G12480	248796_at	AT5G47180
256245_at	AT3G12580	263226_at	AT1G30690	254324_at	AT4G22640	258856_at	AT3G02040	248087_at	AT5G55060	262112_at	AT1G02870	246131_at	AT5G20990	247360_at	AT5G63450
245487_at	AT4G16250	257288_at	AT3G29670	250464_at	AT5G10040	246330_at	AT3G43600	264750_at	AT1G22870	245893_at	AT5G09270	245613_at	AT4G14450	249242_at	AT5G42250
260754_at	AT1G49000	249403_at	AT5G40270	258100_at	AT3G23550	263407_at	AT2G04038	253022_at	AT4G38060	266005_at	AT2G37340	246547_at	AT5G14970	247601_at	AT5G60850
259447_s_at	AT1G02430	265617_at	AT2G25520	245566_at	AT4G14610	262609_at	AT1G13930	262188_at	AT1G77810	246058_at	AT5G08430	247448_at	AT5G62770	247678_at	AT5G59520
	AT1G02440	259683_at	AT1G63050	254665_at	AT4G18340	265033_at	AT1G61520	258984_at	AT3G08970	260968_at	AT1G12250	245119_at	AT2G41640	248555_at	AT5G50340
256598_at	AT3G30180	257739_at	AT3G27470	250443_at	AT5G10520	255764_at	AT1G16720	265909_at	AT2G25720	258776_at	AT3G11830	246327_at	AT1G16670	248366_at	AT5G52510
252098_at	AT3G51330	265067_at	AT1G03850	267493_at	AT2G30400	256504_at	AT1G75210	245293_at	AT4G16660	259437_at	AT1G01540	247629_at	AT5G60410	250569_at	AT5G08130
247707_at	AT5G59450	263265_at	AT2G38820	266010_at	AT2G37430	262826_at	AT1G13080	254780_s_at	AT4G12770	260034_at	AT1G68810	245249_at	AT4G16760	245387_at	AT4G17520
245008_at	ATCG00360	259955_s_at	AT1G75080	257918_at	AT3G23230	262380_at	AT1G72810		AT4G12780	253116_at	AT4G35850	246984_at	AT5G67310	248092_at	AT5G55170
253992_at	AT4G26060		AT1G19350	263571_at	AT2G17050	258181_at	AT3G21670	264957_at	AT1G77000	266322_at	AT2G46690	245252_at	AT4G17500	249354_at	AT5G40480
246870_at	AT5G26030	258511_at	AT3G06590	266878_at	no_match	257722_at	AT3G18490	248619_at	AT5G49630	250353_at	AT5G11630	245250_at	AT4G17490	247748_at	AT5G58920
252631_s_at	AT3G44470	256513_at	AT1G31480	254473_s_at	AT4G20570	265119_at	AT1G62570	248254_at	AT5G53320	250752_at	AT5G05690	247789_at	AT5G58680	249514_at	AT5G38480
	AT2G15210	260887_at	AT1G29160		AT4G20560	262526_at	AT1G17050	263359_at	AT2G15230	250448_at	AT5G10820	245051_at	AT2G23320	247589_at	AT5G60690
	AT2G07250	265423_at	AT2G20790		AT4G20540	258321_at	AT3G22840	245277_at	AT4G15550	260595_at	AT1G55890	247811_at	AT5G58430	247289_at	AT5G64290
	AT3G47280	264856_at	AT2G17370		AT4G20590	262181_at	AT1G78060	263426_at	AT2G31570	265994_at	AT2G24170	247674_at	AT5G59930	250969_at	AT5G02740
	AT2G02190	253171_at	AT4G35040		AT4G20550	253011_at	AT4G37890	266590_at	AT2G46240	262727_at	AT1G75800	248162_at	AT5G54500	246996_at	AT5G67420
	AT5G34890	265718_at	AT2G03340		AT4G20600	260262_at	AT1G68470	264717_at	AT1G70140	265374_at	AT2G06520	248014_at	AT5G56340	247692_s_at	AT5G59970
	AT2G15150	256198_at	AT1G58220		AT4G20650	253302_at	AT4G33660	259150_at	AT3G10320	262820_at	AT1G11680	247817_at	AT5G58375		AT5G59690
252466_x_at	AT3G47270	262487_at	AT1G21610		AT4G20630	253619_at	AT4G30460	247338_at	AT5G63680	258374_at	AT3G14360	246845_at	AT5G26710	249424_s_at	AT5G40080
	AT1G32830	252233_at	AT3G49690		AT4G20530	257222_at	AT3G27925	261250_at	AT1G05890	249121_at	AT5G43830	245711_at	AT5G04340		AT5G39800
256647_at	AT3G13610	260926_at	AT1G21360		AT4G20640	264211_at	AT1G22770	246115_at	AT5G20300	262840_at	AT1G14900	245381_at	AT4G17785	246982_s_at	AT5G04860
266967_at	AT2G39530	249999_at	AT5G18640		AT4G20710	258017_at	AT3G19370	251623_at	AT3G57390	247608_at	AT5G60990	247913_at	AT5G57510		AT2G10560
258262_at	AT3G15770	265471_at	AT2G37130		AT4G20520	248258_at	AT5G53400	266452_at	AT2G43320	262544_at	AT1G15420	244990_s_at	ATCG00870	250285_at	AT5G13300
266992_at	AT2G39200	263699_at	AT1G31120		AT4G20620	262396_at	AT1G49470	256229_at	AT1G58200	261439_at	AT1G28395		ATCG01270	245993_at	AT5G20700
266731_at	AT2G03260	262728_at	AT1G75820		AT4G20610	261071_at	AT1G07380	266181_at	AT2G02390	261197_at	AT1G12900	248316_at	AT5G52670	250861_at	AT5G04740
255266_at	AT4G05200	245063_at	AT2G39795		AT4G20700	246272_at	AT4G37150	249046_at	AT5G44400	259141_at	AT3G10220	247870_at	AT5G57580	249581_at	AT5G37600
262225_at	AT1G53840	252991_at	AT4G38470		AT4G20580	259070_at	AT3G11670	262900_at	AT1G59890	257817_at	AT3G25150	246228_at	AT4G36430	246888_at	AT5G26270
265728_at	AT2G31990	250008_at	AT5G18630	255945_at	AT5G28610	250243_at	AT5G13630	258796_at	AT3G04630	249776_at	AT5G24170	245929_at	AT5G24760	247965_at	AT5G56540
249617_at	AT5G37450	264989_at	AT1G27200	247685_at	AT5G59680	256216_at	AT1G56340	256624_at	AT3G19990	259818_at	AT1G49890	245393_at	AT4G16260	247791_at	AT5G58710
244966_at	ATCG00600	260201_at	AT1G67600	255064_at	AT4G08950	266974_at	AT2G39370	245699_at	AT5G04250	250272_at	AT5G13000	247137_at	AT5G66210	246919_at	AT5G25460
259466_at	AT1G19050	260222_at	AT1G74380	263830_at	AT2G40260	266132_at	AT2G45130	247723_at	AT5G59220	260157_at	AT1G52930	246189_at	AT5G20910	251060_at	AT5G01820
257697_at	AT3G12700	250001_at	AT5G18680	259921_at	AT1G72540	251256_at	AT3G62300	264522_at	AT1G10050	262235_at	AT1G48350	248686_at	AT5G48540	247284_at	AT5G64410
244934_at	ATCG01080	265025_at	AT1G24575	261450_s_at	AT1G21110	260097_at	AT1G73220	264119_at	AT1G79180	252127_at	AT3G50960	248199_at	AT5G54170	249282_at	AT5G41760
266346_at	AT2G01430	266758_at	AT2G46920		AT1G21120	245790_at	AT1G32200	264040_at	AT2G03730	267409_at	AT2G34910	248726_at	AT5G47960	250769_at	AT5G05680
265175_at	AT1G23480	256244_at	AT3G12520	246108_at	AT5G28630	260922_at	AT1G21560	265761_at	AT2G01330	265258_at	AT2G20390	247177_at	AT5G65300	245117_at	AT2G41560
255059_at	AT4G09420	257852_at	AT3G12950	250633_at	AT5G07460	263851_at	AT2G04460	251039_at	AT5G02020	261229_at	AT1G20090	247061_at	AT5G66780	245947_at	AT5G19530
245334_at	AT4G15800	257641_s_at	AT3G25760	252045_at	AT3G52450	260236_at	AT1G74470	254050_s_at	AT4G25670	253536_at	AT4G31580	246099_at	AT5G20230	245784_at	AT1G32190
266461_at	AT2G47730		AT3G25770	264836_at	AT1G03610	264968_at	AT1G67360		AT4G25672	254171_at	AT4G24530	246627_s_at	AT2G45300	248588_at	AT5G49540
262942_at	AT1G79450	265560_at	AT2G05520	256159_at	AT1G30135	260955_at	AT1G06000		AT4G25690	262173_at	AT1G74920		AT1G48860	247779_at	AT5G58760
244940_at	ATCG01230	260264_at	AT1G68500	244924_at	ATMG01040	262231_at	AT1G68740		AT4G25692	264545_at	AT1G55670	245247_at	AT4G17230	246135_at	AT5G20885
	ATCG00905	247312_at	AT5G63970	250796_at	AT5G05300	245090_at	AT2G40900	266747_at	AT2G02870	250547_at	AT5G08100	245533_at	AT4G15130	245392_at	AT4G15680
265301_s_at	AT5G02320	264454_at	AT1G10320	252458_at	AT3G47210	261224_at	AT1G20160	267483_at	AT2G02810	256071_at	AT1G13640	245319_at	AT4G16146	248380_at	AT5G51820
	AT2G13960	259015_at	AT3G07350	260472_at	AT1G10990	255456_at	AT4G02920	265955_at	AT2G37280	248223_at	AT5G53620	246653_at	AT5G35200	248729_at	AT5G48010
263769_at	AT2G06390	261425_at	AT1G18880	266276_at	AT2G29330	259768_at	AT1G29390	262038_at	AT1G35580	259840_at	AT1G52230	245197_at	AT1G67800	248786_at	AT5G47410
266841_at	AT2G26150	260652_at	AT1G32360	265063_at	AT1G61500	260626_at	AT1G08040	248146_at	AT5G54940	259485_at	AT1G15700	247675_at	AT5G59940	247929_at	AT5G57330
259260_at	AT3G11370	266313_at	AT2G26980	260092_at	AT1G73280	258158_at	AT3G17790	263680_at	AT1G26930	263687_at	AT1G26940	247197_at	AT5G65240	247519_at	AT5G61430
249515_at	AT5G38530	263438_at	AT2G28660	266851_at	AT2G26820	267152_at	AT2G31040	255995_at	AT1G29750	245502_at	AT4G15640	247788_at	AT5G58730	248727_at	AT5G47990
261832_at	AT1G10650	260803_at	AT1G78340	265797_at	AT2G35710	267336_at	AT2G19310	249903_at	AT5G22690	265998_at	AT2G24270	248091_at	AT5G55120	247156_at	AT5G65760
262703_at	AT1G16510	245601_at	AT4G14240	263836_at	AT2G40330	261504_at	AT1G71692	247716_at	AT5G59350	263131_at	AT1G78630	247655_at	AT5G59820	246278_at	AT4G37190
261729_s_at	AT1G47860	264894_at	AT1G23040	260399_at	AT1G72520	260693_at	AT1G32450	250265_at	AT5G12900	263437_at	AT2G28670	246955_at	AT5G04870	245594_at	AT4G14570
	AT1G47840	251453_at	AT3G60070	251746_at	AT3G56060	245348_at	AT4G17770	254372_at	AT4G21620	261417_at	AT1G07700	245731_at	AT1G73500	248235_at	AT5G53860
259120_at	AT3G02240	257670_at	AT3G20340	259370_at	AT1G69050	263083_at	AT2G27190	256385_at	AT1G66580	265383_at	AT2G16780	246988_at	AT5G67340	247541_at	AT5G61660
266977_at	AT2G39420	260412_at	AT1G69830	248448_at	AT5G51190	259848_at	AT1G72180	263868_at	AT2G36840	250254_at	AT5G13710	246858_at	AT5G25930	245278_at	AT4G17730
266247_at	AT2G27660	247013_at	AT5G67480	254673_at	AT4G18430	265569_at	AT2G05620	265350_at	AT2G22620	261492_at	AT1G14290	245273_at	AT4G14950	248043_s_at	AT5G56010
261650_at	AT1G27770	255360_at	AT4G03960	248971_at	AT5G45000	260455_at	AT1G72500	261485_at	AT1G14360	261512_at	AT1G71790	247272_at	AT5G64300		AT5G56000
253377_at	AT4G33300			249021_at	AT5G44820	250533_at	AT5G08640	253268_s_at	AT4G34131	246621_at	AT5G36230	245765_at	AT1G33600	248913_at	AT5G45760
258342_at	AT3G22800			261143_at	AT1G19770	253263_at	AT4G34000		AT4G34135	251889_at	AT3G54080	246034_at	AT5G08350	247337_at	AT5G63660
263336_x_at	AT5G35050			257985_at	AT3G20810	263433_at	AT2G22240	260365_at	AT1G70630	260294_at	AT1G63660	246146_at	AT5G20050	250882_at	AT5G04000
257377_at	AT2G28890			249459_at	AT5G39580	248499_at	AT5G50400	256783_at	AT3G13670	247465_at	AT5G62190	245998_at	AT5G20830	249398_at	AT5G40250
253993_at	AT4G26070			261235_x_at	AT1G32840	250881_at	AT5G04080	267026_at	AT2G38340	263371_at	AT2G20490	246528_at	AT5G15640	250095_at	AT5G17230
265948_at	AT2G19590			250142_at	AT5G14650	266720_s_at	AT2G46670	265872_at	AT2G01670	258823_at	AT3G07200	247930_at	AT5G57060	245065_at	AT2G39750
264042_at	AT2G03760			253915_at	AT4G27280		AT2G46790	257880_at	AT3G16910	260448_at	AT1G72410	244970_at	ATCG00660	245736_at	AT1G73330
244933_at	ATCG01070			245082_at	AT2G23270	255645_at	AT4G00880	259489_at	AT1G15790	256158_at	AT1G13590	245734_at	AT1G73480	250813_at	AT5G05000
244962_at	ATCG01050			254487_at	AT4G20780	258794_at	AT3G04710	249963_at	AT5G19000	265671_at	AT2G32060	248570_at	AT5G49780	248878_at	AT5G46160
267385_at	AT2G44380			266052_at	AT2G40740	261751_at	AT1G76080	264001_at	AT2G22420	262029_at	AT1G35680	247583_at	AT5G60750	245399_at	AT4G17340
247394_at	AT5G62860			250920_at	AT5G03390	256983_at	AT3G13470	253212_s_at	AT4G34890	246400_at	AT1G57540	246405_at	AT1G57630	250689_at	AT5G06610
266373_x_at	AT4G10580			259197_at	AT3G03670	264783_at	AT1G08650		AT4G34900	260553_at	AT2G41800	245755_at	AT1G35210	251140_at	AT5G01090
	AT3G31970			260451_at	AT1G72360	265480_at	AT2G15970	252097_at	AT3G51090	263286_at	AT2G36160	245209_at	AT5G12340	249608_at	AT5G37310
	AT2G14650			262099_s_at	AT4G37390	251644_at	AT3G57540	259757_at	AT1G77510	265645_at	AT2G27370	248293_at	AT5G53050	248721_at	AT5G47780
	AT1G42605				AT1G59500	261891_at	AT1G80790	249811_at	AT5G23760	246173_s_at	AT5G28370	247831_at	AT5G58540	245697_at	AT5G04200
244993_s_at	ATCG01000			266784_at	AT2G28960	247025_at	AT5G67030	263827_at	AT2G40420		AT5G28460	248767_at	AT5G47710	248268_at	AT5G53480
	ATCG01130			266872_at	AT2G44730	258652_at	AT3G09910	259478_at	AT1G18980		AT3G61520	245840_at	AT1G58420	248095_at	AT5G55230
266965_at	AT2G39510			254291_at	AT4G23010	264940_at	AT1G60470	248862_at	AT5G46730	248027_at	AT5G55630	246312_at	AT1G31930	248149_at	AT5G54855
260656_at	AT1G19380			248991_at	AT5G45220	250665_at	AT5G06980	248207_at	AT5G53970	254705_at	AT4G17870	245244_at	AT1G44350	245364_at	AT4G15790
265992_at	AT2G24130			257536_at	AT3G02800	263345_s_at	AT2G05070	262912_at	AT1G59740	255570_at	AT4G01100	247389_at	AT5G63490	249801_at	AT5G23580
260567_at	AT2G43820			267288_at	AT2G23680		AT2G05100	261221_at	AT1G19960	260765_at	AT1G49240	246158_at	AT5G19855	249842_at	AT5G23550
266670_at	AT2G29740			254091_at	AT4G25070	252429_at	AT3G47500	248176_at	AT5G54650	266466_at	AT2G47790	245854_at	AT5G13490	248477_at	AT5G50900
263208_at	AT1G10480			261029_at	AT1G17330	249769_at	AT5G24120	259618_at	AT1G48000	264264_at	AT1G09250	245976_at	AT5G13080	247945_at	AT5G57150
250302_at	AT5G11920			245757_at	AT1G35140	263073_at	AT2G17500	255723_at	AT3G29575	260368_at	AT1G69700	246750_at	AT5G27860	246026_at	AT5G21070
263935_at	AT2G35930			245838_at	AT1G58410	255877_at	AT2G40460	264629_at	AT1G65540	267318_at	AT2G34770	246195_at	AT4G36410	249152_s_at	AT5G43350
253999_at	AT4G26200			248703_at	AT5G48430	259399_at	AT1G17710	254630_at	AT4G18360	260898_at	AT1G29070	245600_at	AT4G14230		AT5G43370
266798_at	AT2G22850			256170_at	AT1G51790	263954_at	AT2G35840	264852_at	AT2G17480	259013_at	AT3G07430	248394_at	AT5G52070	248249_at	AT5G53140
263374_at	AT2G20560			264766_at	AT1G61420	246481_s_at	AT5G15960	245200_at	AT1G67850	266334_at	AT2G32380	246927_s_at	AT5G25260	250474_at	AT5G10230
244994_at	ATCG01010			264008_at	AT2G21120		AT5G15970	260517_at	AT1G51420	263247_at	AT2G31440		AT5G25250	246191_at	AT5G20890
266834_s_at	AT2G05050			261249_at	AT1G05880	261574_at	AT1G01190	260243_at	AT1G63720	258421_at	AT3G16690	247618_at	AT5G60280	250144_at	AT5G14680
	AT3G27140			259896_at	AT1G71500	264402_at	AT2G25140	267367_at	AT2G44210	248746_at	AT5G47890	246600_at	AT5G14930	246759_at	AT5G27950
	AT4G08260			252739_at	AT3G43250	245904_at	AT5G11110	264507_at	AT1G09415	253043_at	AT4G37540	246368_at	AT1G51890	246681_at	AT5G33280
	AT2G30020			259841_at	AT1G52200	258167_at	AT3G21560	253219_at	AT4G34990	263892_at	AT2G36890	247922_at	AT5G57500	246735_at	AT5G27670
254347_at	AT4G22070			256128_at	AT1G18140	252123_at	AT3G51240	255725_at	AT1G25540	260984_at	AT1G53645	246779_at	AT5G27520	251109_at	AT5G01600
260227_at	AT1G74450			264232_at	AT1G67470	260956_at	AT1G06040	249491_at	AT5G39130	265416_at	AT2G37120	245158_at	AT2G33130	249778_at	AT5G24165
255564_s_at	AT4G01770			247925_at	AT5G57560	247918_at	AT5G57610	262691_at	AT1G62740	261614_at	AT1G49760	248335_at	AT5G52450	248797_at	AT5G47210
	AT4G01750			254521_at	AT5G44820	256266_at	AT3G12320	254843_at	AT4G11860	257076_at	AT3G19680	247864_s_at	AT1G25155	248778_at	AT5G47940
254384_at	AT4G21870			247524_at	AT5G61440	258860_at	AT3G02050	245742_at	AT1G44170	261930_at	AT1G22440		AT5G57890	247185_at	AT5G65460
244973_at	ATCG00690			254163_s_at	AT4G24340	250906_at	AT5G03650	247178_at	AT5G65205	254923_at	AT4G11240		AT1G25083	245482_at	AT4G16180
257319_at	ATMG01100				AT4G24350	254166_at	AT4G24190	247044_at	AT5G66850	264189_s_at	AT1G54580		AT1G24909	246288_at	AT1G31850
266749_at	AT2G47060			267024_s_at	AT2G34390	254766_at	AT4G13280	251766_at	AT3G55910		AT1G54630		AT1G25220	249790_at	AT5G24290
266546_at	AT2G35270				AT2G29870	259491_at	AT1G15820	261395_at	AT1G79700	264092_at	AT1G79040		AT1G24807	245361_at	AT4G17790
262743_at	AT1G29020			261449_at	AT1G21120	263875_at	AT2G21970	250221_at	AT5G14220	255131_at	AT4G08280	247458_at	AT5G62180	246103_at	AT5G28640
259443_at	AT1G02360			245708_at	AT5G04310	262644_at	AT1G62710	251743_at	AT3G55890	265182_at	AT1G23740	246976_s_at	AT5G24810	249599_at	AT5G37990
266039_s_at	AT2G07739			247582_at	AT5G60760	251827_at	AT3G55120	258524_at	AT3G06810	265101_at	AT1G30880	245363_at	AT4G15120	245301_at	AT4G17190
	ATMG00370			254200_at	AT4G24110	247293_at	AT5G64510	265077_at	AT1G55530	265594_at	AT2G20060	247819_at	AT5G58350	245637_at	AT1G25230
249097_at	AT5G43520			251459_at	AT3G60200	266555_at	AT2G46270	264690_at	AT1G70060	263737_at	AT1G60010	247571_at	AT5G61210	247266_at	AT5G64570
266371_at	AT2G41410			265841_at	AT2G35710	246055_at	AT5G08380	267181_at	AT2G37760	262162_at	AT1G78020	247071_at	AT5G66640	245260_at	AT4G16360
265668_at	AT2G32020			258364_at	AT3G14225	267002_s_at	AT2G34430	261838_at	AT1G16030	261672_at	AT1G18450	247630_at	AT5G60410	250844_at	AT5G04470
245016_at	ATCG00500			263893_at	AT2G21900		AT2G34420	260303_at	AT1G70520	266625_at	AT2G35380	246863_at	AT5G25940	246476_at	AT5G16730
253249_at	AT4G34680			263854_at	AT2G04430	266170_at	AT2G39050	256666_at	AT3G20670	262587_at	AT1G15490	246406_at	AT1G57650	250794_at	AT5G05270
267436_at	AT2G19190			263126_at	AT1G78460	248337_at	AT5G52310	266221_at	AT2G28760	251173_at	AT3G63270	247145_at	AT5G65600	248419_at	AT5G51550
263280_x_at	AT1G32830			245254_at	AT4G14680	267590_at	AT2G39700	266290_at	AT2G29490	264907_at	AT2G17280	248327_at	AT5G52750	245525_at	AT4G15930
	AT2G14140			262001_at	AT1G33790	260076_at	AT1G73630	250930_at	AT5G03160	261685_at	AT1G47290	247412_at	AT5G63010	248674_at	AT5G48800
248306_at	AT5G52830			259028_at	AT3G09290	256999_at	AT3G14200	248124_at	AT5G54730	262846_at	AT1G14670	248440_at	AT5G51260	246281_at	AT4G36940
257387_s_at	AT2G14510			251059_at	AT5G01810	256454_at	AT1G75280	265700_at	AT2G03470	260541_at	AT2G43530	248011_at	AT5G56300	247288_at	AT5G64330
	AT2G14440			248569_at	AT5G49770	249763_at	AT5G24010	267341_at	AT2G44200	258251_at	AT3G15810	247554_at	AT5G61010	248728_at	AT5G48000
266012_s_at	ATMG00410			260783_at	AT1G06160	262780_at	AT1G13090	259706_at	AT1G77540	248739_at	AT5G48030	248416_at	AT5G51630	249810_at	AT5G23920
	AT2G07741			246167_at	no_match	266900_at	AT2G34610	250077_at	AT5G16680	266500_at	AT2G06925	247240_at	AT5G64660	249677_at	AT5G35970
	ATMG01170			265725_at	AT2G32030	250661_at	AT5G07030	260551_at	AT2G43510	266051_at	AT2G40760	245041_at	AT2G26530	247746_at	AT5G58970
	AT2G07699			264475_s_at	AT1G77150	251869_at	AT3G54500	264148_at	AT1G02220	267138_s_at	AT2G38230	245660_at	AT1G28240	248723_at	AT5G47950
261063_at	AT1G07520				AT1G77170	263128_at	AT1G78600	260837_at	AT1G43670		AT2G38210	246214_at	AT4G36988	248042_at	AT5G55960
254211_at	AT4G23570			265648_at	AT2G27500	266209_at	AT2G27550	256022_at	AT1G58360	264313_at	AT1G70410		AT4G36990	249710_at	AT5G35630
245457_s_at	AT4G16940			245567_at	AT4G14630	247222_at	AT5G64840	254781_at	AT4G12840	245427_at	AT4G17550	246335_at	AT3G44880	245015_at	ATCG00490
	AT4G16960			258727_at	AT3G11930	255016_at	AT4G10120	245775_at	AT1G30270	251610_at	AT3G57930	247392_at	AT5G63570	245621_at	AT4G14070
244959_s_at	AT2G07708			261459_at	AT1G21100	247921_at	AT5G57660	261176_at	AT1G04780	265247_at	AT2G43030	248164_at	AT5G54490	248140_at	AT5G54980
	ATMG00500			245706_at	AT5G04310	247323_at	AT5G64170	251541_at	AT3G58750	249544_at	AT5G38110	245768_at	AT1G33590	245915_s_at	AT5G19780
	ATMG00490			252851_at	AT4G40080	262940_at	AT1G79520	265559_at	AT2G05530	266904_at	AT2G34590	246239_at	AT4G36790		AT5G19770

267530_at	AT2G41890	247693_at	AT5G59730	267612_at	AT2G26690	257951_at	AT3G21700	261761_at	AT1G15660	248333_at	AT5G52390	245991_at	AT5G20660
262905_at	AT1G59730	264759_at	AT1G61480	266327_at	AT2G46680	258880_at	AT3G06420	260426_at	AT1G72370	246460_at	AT5G16910	247935_at	AT5G56940
244995_at	ATCG00150			262388_at	AT1G49320	260649_at	AT1G08080	261774_at	AT1G76260	248564_at	AT5G49700	250429_at	AT5G10470
262517_at	AT1G17180			263320_at	AT2G47180	253064_at	AT4G37730	258249_s_at	AT3G15830	246250_at	AT4G36880	246216_at	AT4G36380
263231_at	AT1G05680			264260_at	AT1G09210				AT3G15820	248487_at	AT5G51070	248687_at	AT5G48300
247326_at	AT5G64110							264198_at	AT1G22800	246233_at	AT4G36550	251078_at	AT5G01990
265460_at	AT2G46600							254535_at	AT4G19710	245777_at	AT1G73540	251152_at	AT3G63130
261234_x_at	AT2G04970							246060_at	AT5G08420	248706_at	AT5G48530	249452_at	AT5G39500
	AT1G32830							266264_at	AT2G27775	246018_at	AT5G10695	249765_at	AT5G24030
	AT3G47270							258124_at	AT3G18215	245447_at	AT4G16820	245127_at	AT2G47600
	AT2G14140							266554_s_at	AT2G46290	248209_at	AT5G53990	250305_at	AT5G12150
262525_at	AT1G17060								AT2G46280	247686_at	AT5G59700	249797_at	AT5G23750
259454_at	AT1G44050							261583_at	AT1G01090	247165_at	AT5G65790	246830_at	AT5G26360
265435_s_at	AT2G21020							255619_s_at	AT5G59920	246370_at	AT1G51920	247175_at	AT5G65280
	AT1G31885								AT4G01350	246880_s_at	AT5G26000	247469_at	AT5G62165
251832_at	AT3G55150							267526_at	AT2G30570		AT5G25980	248412_at	AT5G51590
266769_s_at	AT1G18930							264337_at	AT1G70350	248629_at	AT5G48970	246580_at	AT1G31770
	AT3G29440							260282_at	AT1G80410	245266_at	AT4G17070	249347_at	AT5G40830
	AT3G44390							262958_at	AT1G54410	247370_at	AT5G63320	249202_at	AT5G42580
	AT4G12426							252472_at	AT3G46830	247205_at	AT5G64890	249193_at	AT5G42480
	AT3G47165							262813_at	AT1G11670	245365_at	AT4G17720	247249_at	AT5G64670
	AT5G33315							257807_at	AT3G26650	246945_s_at	AT5G25050	248511_at	AT5G50375
	AT5G26590							266690_at	AT2G19900	247438_at	AT5G62460	250278_at	AT5G12860
	AT3G42883							265798_at	AT2G35790	248719_at	AT5G47910	247801_at	AT5G58560
	AT1G12935							267435_at	AT2G33800	248568_at	AT5G49760	247771_at	AT5G58590
	AT5G28466							265042_at	AT1G04040	245866_s_at	AT1G57990	249267_at	AT5G41600
	AT2G03080							265776_at	AT2G07340		AT1G57980	249165_at	AT5G42810
	AT3G50490							254562_at	AT4G19230	245875_at	AT1G26240	247192_at	AT5G65360
	AT2G04140							262045_at	AT1G80240	246325_at	AT1G16540	245748_at	AT1G51140
	AT2G04130							258486_at	AT3G02560	247790_at	AT5G58720	247108_at	AT5G66160
	AT5G56605							260840_at	AT1G29050	248615_at	AT5G49570	248283_at	AT5G52920
	AT4G17820							252508_at	AT3G46210	246631_at	AT1G50740	250315_at	AT5G12130
	AT4G20180							260910_at	AT1G02690	247132_at	AT5G66200	246483_at	AT5G16000
244977_at	ATCG00730							266613_at	AT2G14900	246620_at	AT5G36220	248395_at	AT5G52120
265414_at	AT2G16660							260914_at	AT1G02640	245694_at	AT5G04170	250203_at	AT5G13980
262822_at	AT1G11630							262128_at	AT1G52690	246221_at	AT4G37120	250420_at	AT5G11260
259937_s_at	AT3G13080							248655_at	AT5G48760	245666_at	AT1G28280	245281_at	AT4G15560
	AT1G71330							266885_at	AT2G44610	247125_at	AT5G66070	248801_at	AT5G47370
252596_at	AT3G45330							245146_at	AT2G45200	244920_s_at	AT2G07751	249299_at	AT5G41340
261090_at	AT1G07560							267337_at	AT2G39980		ATMG00990	247477_at	AT5G62340
262131_at	AT1G02900							259687_at	AT1G63110	247940_at	AT5G57190	249293_at	AT5G41260
244932_at	ATCG01060							250538_at	AT5G08620	244957_at	ATMG00400	245386_at	AT4G14010
267588_at	AT2G42060							262946_at	AT1G79390	247086_at	AT5G66320	250016_at	AT5G18100
244965_at	ATCG00590							248103_at	AT5G55160	246777_at	AT5G27420	250562_at	AT5G08040
266032_x_at	AT2G05890							251018_at	AT5G02450	247395_at	AT5G62910	250625_at	AT5G07340
260540_at	AT2G43500							256010_at	AT1G19220	245038_at	AT2G26560	247059_at	AT5G66690
264685_at	AT1G65610							258412_at	AT3G17210	245662_at	AT1G28190	249036_at	AT5G44200
245011_at	ATCG00430							259727_at	AT1G60950	247949_at	AT5G57220	246076_at	AT5G20280
255502_at	AT4G02410							261267_at	AT1G26750	247611_at	AT5G60710	250125_at	AT5G16390
244999_at	ATCG00190							265895_at	AT2G15000	245329_at	AT4G14365	248418_at	AT5G51660
267064_at	AT2G41110							263973_at	AT2G42740	247487_at	AT5G62150	250735_at	AT5G06280
266045_s_at	AT2G07727							265445_at	AT2G37190	246744_at	AT5G27760	251122_at	AT5G01020
	ATMG00220							261418_at	AT1G07830	248154_at	AT5G54400	246045_at	AT5G19430
265733_at	AT2G01190							263375_s_at	AT2G20530	245226_at	AT3G29970	248558_at	AT5G49990
261846_at	AT1G11540								AT4G28510	248195_at	AT5G54110	247770_at	AT5G58930
263357_s_at	AT2G07250							262712_at	AT1G16460	245323_at	AT4G16500	247356_at	AT5G63800
	AT5G34890							255830_at	AT2G33340	245173_at	AT2G47520	248454_at	AT5G51350
	AT3G30455							249955_at	AT5G18840	245151_at	AT2G47550	245556_at	AT4G15400
	AT3G32901							262172_at	AT1G74970	248562_at	AT5G49680	245139_at	AT2G45430
	AT1G32820							253558_at	AT4G31120	244944_s_at	ATMG00080	249545_at	AT5G38030
	AT2G15210							266225_at	AT2G28900		ATMG00090	251151_at	AT3G63170
	AT3G47280							261664_s_at	AT1G18320	245686_at	AT5G22060	246714_at	AT5G28220
	AT4G03980								AT3G10110	248424_at	AT5G51680	248034_at	AT5G55910
	AT2G04950							258152_at	AT3G18190	247138_at	AT5G66120	245730_at	AT1G73470
	AT2G15150							260442_at	AT1G68220	245702_at	AT5G04220	247845_at	AT5G58090
	AT2G14150							260781_at	AT1G14620	246238_at	AT4G36670	248865_at	AT5G46790
	AT2G06450							261377_at	AT1G18850	245148_at	AT2G45220	250502_at	AT5G09590
255116_at	AT4G08850							254617_s_at	AT5G45775	247509_at	AT5G62020	249147_at	AT5G43330
262254_at	AT1G53920								AT4G18730	245178_at	AT5G12390	249309_at	AT5G41410
244961_at	ATCG01040							247212_at	AT5G65040	246785_at	AT5G27380	250817_at	AT5G04940
245000_at	ATCG00210							266924_at	AT2G45730	248322_at	AT5G52760	249093_at	AT5G43880
255787_at	AT2G33590							262618_at	AT1G06560	246293_at	AT3G56710	247058_at	AT5G66680
266785_at	AT2G28970							258090_at	AT3G14600	247532_at	AT5G61560	248459_at	AT5G51020
245025_at	ATCG00130							261603_at	AT1G49600	246931_at	AT5G25170	245282_at	AT4G14990
250777_at	AT5G05440							246768_at	AT5G27400	246485_at	AT5G16080	250740_at	AT5G05760
252634_x_at	AT4G04010							263844_at	AT2G36930	245794_at	AT1G32170	246017_at	AT5G10730
	AT3G44500							246003_at	AT5G20720	245550_at	AT4G15330	250407_at	AT5G10870
	AT3G30440							261696_at	AT1G08470	246989_at	AT5G67350	247964_at	AT5G56600
249364_at	AT5G40590							267439_at	AT2G19060	246453_at	AT5G16830	249327_at	AT5G40890
252474_at	AT3G46620							253881_at	AT4G27640	247835_at	AT5G57910	248557_at	AT5G49980
267384_at	AT2G44370							259640_at	AT1G52400	247215_at	AT5G64905	249400_at	AT5G40300
263279_x_at	AT3G47260							260946_at	AT1G06010	246409_at	AT1G57700	250671_at	AT5G06950
	AT2G14130							264702_at	AT1G70190	247679_at	AT5G59540	247214_at	AT5G64850
244992_s_at	ATCG00900							258747_at	AT3G05810	246790_at	AT5G27610	247077_at	AT5G66420
	ATCG01240							246521_at	AT5G15810	247740_at	AT5G58940	245202_at	AT1G67720
267372_at	AT2G26290							260226_at	AT1G74660	245167_s_at	AT2G33120	245888_at	AT5G09450
267069_at	AT2G41010							265010_at	AT1G24510		AT2G33110	250724_at	AT5G06330
251096_at	AT5G01550							265076_at	AT1G55490	245193_at	AT1G67810	249994_at	AT5G18590
255197_x_at	AT4G07450							257104_at	AT3G25040	245599_at	AT4G14220	250002_at	AT5G18690
257479_at	AT1G16150							248127_at	AT5G54750	248462_at	AT5G50960	250527_at	AT5G08590
248964_at	AT5G45340							261991_at	AT1G33700	247026_at	AT5G67080	246478_at	AT5G15980
260037_at	AT1G68840							251552_at	AT3G58700	246440_at	AT5G17650	250928_at	AT5G03280
267240_at	AT2G02680							262632_at	AT1G06680	247313_at	AT5G63980	249318_at	AT5G40870
263254_x_at	AT3G44500							255789_at	AT2G33370	246213_at	AT4G36480	247101_at	AT5G66530
	AT2G06430							260670_at	AT1G19520	247368_at	AT5G63330	249736_at	AT5G24460
	AT4G04010							258269_at	AT3G15690	245035_at	AT2G26400	251143_at	AT5G01220
	AT2G02210							260767_s_at	AT1G49140	247708_at	AT5G59550	246855_at	AT5G26280
244983_at	ATCG00790								AT3G18410	248618_at	AT5G49620	245644_at	AT1G25320
248979_at	AT5G45080							263632_at	AT2G04795	246270_at	AT4G36500	259240_at	AT3G11590
264000_at	AT2G22500							260639_at	AT1G53180	248134_at	AT5G54860	252824_at	AT4G40030
247327_at	AT5G64120							245116_at	AT2G41620	247351_at	AT5G63790	253200_at	AT4G34720
254195_at	AT4G24020							262557_at	AT1G31330	248392_at	AT5G52050	252737_at	AT3G43220
254723_at	AT4G13510							264346_at	AT1G12010	246529_at	AT5G15730	258849_at	AT3G03250
264005_at	AT2G22470									246702_at	AT5G28050	258795_at	AT3G04570
265186_at	AT1G23560									246832_at	AT5G26600	258393_at	AT3G15480
244972_at	ATCG00680									247493_at	AT5G61900	259040_at	AT3G09270
259251_at	AT3G07600										Arabidopsis	259476_at	AT1G19000
249779_at	AT5G24230									253588_at	AT4G30790	253483_at	AT4G31910
252465_x_at	AT3G44500									249580_at	AT5G37740	253538_at	AT4G31460
	AT3G47260									252825_at	AT4G39890	251269_at	AT3G62360
	AT3G30440									249730_at	AT5G24430	256387_at	AT3G06170
248978_at	AT5G45070									250793_at	AT5G05600	257879_at	AT3G17160
251922_at	AT3G54030									250267_at	AT5G12930	252975_s_at	AT4G38430
266203_at	AT2G02230									251774_at	AT3G55840	251641_at	AT3G57470
254586_at	AT4G19510									253646_at	AT4G29810	254636_at	AT4G18700
248607_at	AT5G49480									253074_at	AT4G36140	256156_at	AT3G08510
245370_at	AT4G16840									252602_at	AT3G45040	255924_at	AT1G22170
254197_at	AT4G24040									249050_at	AT5G44290	252677_at	AT3G44320
264282_at	AT1G61840									252547_at	AT3G45840	258887_at	AT3G05630
260481_at	AT1G10960									253075_at	AT4G36150	258474_at	AT3G02650
266222_at	AT2G28780									251037_at	AT5G02100	259287_at	AT3G11490
245005_at	ATCG00330									249337_at	AT5G41080	257313_at	AT3G26520
259888_at	AT1G76350									253606_at	AT4G30530	254456_at	AT4G21150
254053_s_at	AT4G25310									252134_at	AT3G50910	258050_at	AT3G16200
	AT4G25300									253910_at	AT4G27290	252730_at	AT3G43110
266787_at	AT2G28990									250692_at	AT5G06560	257730_at	AT3G18420
262793_at	AT1G13110									252993_at	AT4G38540	253005_at	AT4G38100
245010_at	ATCG00420									252427_at	AT3G47640	259357_at	AT1G13380
245171_at	AT2G47560									249481_at	AT5G38900	252308_at	AT3G49310
266196_at	AT2G39110									253630_at	AT4G30490	256092_at	AT1G20696
249480_s_at	AT5G38990									250415_at	AT5G11210	259094_at	AT3G04940
	AT5G39000									251790_at	AT3G55470	254729_at	AT4G13700

244974_at	ATCG00700
267034_at	AT2G38310
263800_at	AT2G24600
253535_at	AT4G31550
254759_at	AT4G13180
256417_s_at	AT3G11170
	AT5G05580
263232_at	AT1G05700
245152_at	AT2G47490
254093_at	AT4G25110
266719_at	AT2G46830
256169_at	AT1G51800
267293_at	AT2G23810
245018_at	ATCG00520
250153_at	AT5G15130
247297_at	AT5G64100
266561_at	AT2G23960
266112_x_at	AT2G02210
	AT3G47260
250941_at	AT5G03320

251106_at	AT5G01500	252969_at	AT4G38900
252908_at	AT4G39670	258015_at	AT3G19340
251063_at	AT5G01850	258128_at	AT3G24590
250911_at	AT5G03730	256002_at	AT1G29900
251973_at	AT3G53180	252488_at	AT3G46700
250607_at	AT5G07370	255486_at	AT4G02600
250264_at	AT5G12890	255365_at	AT4G04040
252870_at	AT4G39940	258286_at	AT3G16060
251769_at	AT3G55950	252181_at	AT3G50685
252053_at	AT3G52400	259384_at	AT3G16450
251597_at	AT3G57750	256192_at	AT1G30110
250496_at	AT5G09650	255279_at	AT4G04950
248981_at	AT5G45110	259319_at	AT3G01090
252905_at	AT4G39720	254791_at	AT4G12910
250833_at	AT5G04540	253158_at	AT4G35780
253099_s_at	AT4G37530	252884_at	AT4G39170
	AT4G37520	259010_at	AT3G07340
251950_at	AT3G53600	252512_at	AT3G46290
249835_s_at	AT5G23490	254827_at	AT4G12650
	AT5G23510	258403_at	AT3G15380
249339_at	AT5G41100	257638_at	AT3G25820
253271_s_at	AT4G34470	253481_at	AT4G31990
	AT4G34210	252502_at	AT3G46900
252831_at	AT4G39980	259328_at	AT3G16440
251053_at	AT5G01490	253225_at	AT4G35020
253874_at	AT4G27450	254693_at	AT4G17880
251918_at	AT3G54040	258329_at	AT3G16110
253339_at	AT4G33520	256677_at	AT3G52190
253467_at	AT4G32140	251830_at	AT3G55010
249361_at	AT5G40540	259383_at	AT3G16470
250472_at	AT5G10210	251741_at	AT3G56040
252170_at	AT3G50480	258735_at	AT3G05880
249476_at	AT5G38910	253547_at	AT4G30950
248868_at	AT5G46780	257798_at	AT3G15950
252378_at	AT3G47570	253692_at	AT4G29720
251475_at	AT3G59660	256722_at	AT2G34050
249264_s_at	AT5G41750	255587_at	AT4G01480
	AT5G41740	253175_at	AT4G35050
253841_at	AT4G27830	257375_at	AT2G38640
253573_at	AT4G31020	259382_s_at	AT3G16430
250575_at	AT5G08240		AT3G16420
249471_at	AT5G39360	255735_at	AT1G25520
251901_at	AT3G54100	255631_at	AT4G00710
251624_at	AT3G57280	252282_at	AT3G49360
249001_at	AT5G44990	258933_at	AT3G09980
251847_at	AT3G54640	258526_at	AT3G06790
253819_at	AT4G28350	253765_at	AT4G28740
250599_at	AT5G07730	259276_at	AT3G01190
250990_at	AT5G02290	258805_at	AT3G04010
249942_at	AT5G22300	259231_at	AT3G11410
248994_at	AT5G45250	257911_at	AT3G25530
253813_at	AT4G28150	255621_at	AT4G01390
253828_at	AT4G27970	258585_at	AT3G04340
249747_at	AT5G24600	255674_at	AT4G00430
251705_at	AT3G56400	253189_at	AT4G35335
253505_at	AT4G31970	254417_at	AT4G21470
253343_at	AT4G33540	259381_s_at	AT3G16410
253950_at	AT4G26910		AT3G16390
249197_at	AT5G42380		AT3G16400
253063_at	AT4G37640	253775_at	AT4G28440
249589_at	AT5G37850	259277_at	AT3G01180
252928_at	AT4G38940	258866_at	AT3G03180
251684_at	AT3G56410	252591_at	AT3G45600
251028_at	AT5G02230	259318_at	AT3G01100
250306_at	AT5G12170	255721_at	AT1G31940
250846_at	AT5G04590	251169_at	AT3G63210
251144_at	AT5G01210	255686_at	AT4G00630
253830_at	AT4G27652	253525_at	AT4G31330
253184_at	AT4G35230	258992_at	AT3G08930
253643_at	AT4G29780	257253_at	AT3G24190
252882_at	AT4G39675	253607_at	AT4G30330
250286_at	AT5G13320	251658_at	AT3G57020
250875_at	AT5G04020	253359_at	AT4G33080
253277_at	AT4G34230	259237_at	AT3G11630
253173_at	AT4G35110	253053_at	AT4G37470
251884_at	AT3G54150	256091_at	AT1G20693
249346_at	AT5G40780	258092_at	AT3G14595
253046_at	AT4G37370	254037_at	AT4G25760
250483_at	AT5G10300	251307_at	AT3G61200
249487_at	AT5G39040	255035_at	AT4G09550
250177_at	AT5G14420	259060_at	AT3G07400
249188_at	AT5G42830	255638_at	AT4G00740
252478_at	AT3G46540	253218_at	AT4G34980
249987_at	AT5G18490	253386_at	AT4G33030
250335_at	AT5G11650	258109_at	AT3G23640
253124_at	AT4G36030	259310_s_at	AT3G05160
251121_at	AT3G63420		AT3G05165
250014_at	AT5G17990	251309_at	AT3G61220
250289_at	AT5G13190	257959_at	AT3G25560
250821_at	AT5G05190	254820_s_at	AT4G12510
253167_at	AT4G35310		AT4G12520
252133_at	AT3G50900	259366_at	AT1G13280
250582_at	AT5G07580	252926_at	AT4G38920
250662_at	AT5G07010	251924_at	AT3G53730
253321_at	AT4G33910	256110_at	AT1G16900
249893_at	AT5G22555	253706_at	AT4G29120
249652_at	AT5G37070	253903_at	AT4G27180
249928_at	AT5G22250	255298_at	AT4G04840
253181_at	AT4G35180	251285_at	AT3G61770
251742_at	AT3G56050	259291_at	AT3G11550
253431_at	AT4G32440	258416_at	AT3G17310
253038_at	AT4G37790	259343_s_at	AT5G17920
252126_at	AT3G50950		AT3G03780
249983_at	AT5G18470	257596_at	AT3G24760
249719_at	AT5G35735	251396_at	AT3G60750
250948_at	AT5G03490	255561_at	AT4G02050
249072_at	AT5G44060	252878_at	AT4G39460
253284_at	AT4G34150	253440_at	AT4G32570
248814_at	AT5G46910	252982_at	AT4G38130
252939_at	AT4G39230	254721_at	AT4G13520
250435_at	AT5G10380	252064_at	AT3G51520
251745_at	AT3G55980	253510_at	AT4G31730
248848_at	AT5G46520	254828_at	AT4G12550
252037_at	AT3G51920	256828_at	AT3G22970
249941_at	AT5G22270		AT3G22968
251494_at	AT3G59350	251679_at	AT3G57030
252131_at	AT3G50930	253188_at	AT4G35300
251478_at	AT3G59690	259505_at	AT1G15810
249841_at	AT5G23520	255630_at	AT4G00700
249495_at	AT5G39100	253475_at	AT4G32290
249333_at	AT5G40990	252669_at	AT3G44100
251786_at	AT3G55270	254901_at	AT4G11530
248934_at	AT5G46080	257044_at	AT3G19720
253534_at	AT4G31500	256482_at	AT1G31420
253476_at	AT4G32300	257710_at	AT3G27350
252903_at	AT4G39570	256994_s_at	AT3G25830
250944_at	AT5G03380		AT3G25820
248821_at	AT5G47070	252010_at	AT3G52740
252751_at	AT3G43430	259300_at	AT3G05100
251370_at	AT3G60450	258020_at	AT3G19490
249081_at	AT5G44090	256960_at	AT3G13510
250248_at	AT5G13740	258637_at	AT3G07880

248996_at	AT5G45260	251315_at	AT3G61410
251971_at	AT3G53160	257227_at	AT3G27820
252940_at	AT4G39270	257673_at	AT3G20370
253664_at	AT4G30210	253300_at	AT4G33580
253323_at	AT4G33920	258368_at	AT3G14240
248794_at	AT5G47220	251993_at	AT3G52960
253414_at	AT4G33050	258613_at	AT3G02870
253057_at	AT4G37670	253842_at	AT4G27860
252331_s_at	AT3G48780	251695_at	AT3G56590
	AT3G48790	255243_at	AT4G05590
249417_at	AT5G39670	258264_at	AT3G15790
252900_at	AT4G39540	254225_at	AT4G23670
251839_at	AT3G54950	256134_at	AT1G13560
251400_at	AT3G60420	257855_at	AT3G13040
253654_at	AT4G30060	253317_at	AT4G33960
250738_at	AT5G05730	254874_at	AT4G11570
251848_at	AT3G54620	254749_at	AT4G13130
251904_at	AT3G54130	253980_at	AT4G26620
251649_at	AT3G57330	253559_at	AT4G31140
252470_at	AT3G46930	257218_at	AT3G15000
250385_at	AT5G11520	254405_at	AT4G21350
251507_at	AT3G59080	252954_at	AT4G38660
249255_at	AT5G41610	258755_at	AT3G11945
252862_at	AT4G39830	253059_s_at	AT4G37680
251763_at	AT3G55730		AT4G38320
253012_at	AT4G37900	253073_at	AT4G37410
253257_at	AT4G34390	258454_at	AT3G22300
251248_at	AT3G62150	256131_at	AT1G13600
253702_at	AT4G29900	253519_at	AT4G31240
251379_at	AT3G60680	256029_at	AT1G34130
251456_at	AT3G60120	252712_at	AT3G43800
252906_at	AT4G39640	255798_at	AT2G33255
253458_at	AT4G32070	251305_at	AT3G62030
253324_at	AT4G33940	259288_at	AT3G11500
249208_at	AT5G42650	251856_at	AT3G54720
250025_at	AT5G18290	253920_at	AT4G27230
249329_at	AT5G40960	259327_at	AT3G16460
250055_at	AT5G17770	259131_at	AT3G02180
249251_at	AT5G42000	257162_s_at	AT3G24290
248995_at	AT5G45260		AT3G24300
250292_at	AT5G13220	251226_at	AT3G62680
253162_at	AT4G35630	253977_at	AT4G26630
253578_at	AT4G30340	253079_s_at	AT4G36190
249029_at	AT5G44870		AT4G36195
249078_at	AT5G44070	253216_at	AT4G34960
251086_at	AT5G01450	253731_at	AT4G29260
252300_at	AT3G49160	254461_at	AT4G21230
252214_at	AT3G50260	251254_at	AT3G62270
249334_at	AT5G41000	254054_at	AT4G25320
249984_at	AT5G18400	259426_at	AT1G01470
251970_at	AT3G53150	255858_at	AT1G67030
250676_at	AT5G06320	254153_at	AT4G24450
253044_at	AT4G37290	259012_at	AT3G07360
249806_at	AT5G23850	255650_s_at	AT5G37190
253193_at	AT4G35380		AT4G00930
251071_at	AT5G01950	258646_at	AT3G08040
252364_at	AT3G48450	254119_at	AT4G24780
248967_at	AT5G45350	254631_at	AT4G18610
250580_at	AT5G07440	259417_at	AT1G02340
253342_at	AT4G33520	259000_at	AT3G01860
253332_at	AT4G33420	256072_at	AT1G18080
248845_at	AT5G46470	256986_at	AT3G28700
251176_at	AT3G63380	254703_at	AT4G17960
250097_at	AT5G17280	252094_at	AT3G51260
253637_at	AT4G30390	255743_at	AT1G25375
250492_at	AT5G09790	259416_at	AT1G02305
249967_at	AT5G19050	253774_at	AT4G28530
249896_at	AT5G22530	252232_at	AT3G49760
252379_at	AT3G47730	254915_s_at	AT4G11320
250279_at	AT5G13200		AT4G11310
252265_at	AT3G49620	256848_at	AT3G27960
250098_at	AT5G17350	252033_at	AT3G51950
251640_at	AT3G57450	254239_at	AT4G23400
253709_at	AT4G29220	256441_at	AT3G10940
252679_at	AT3G44260	256728_at	AT3G25660
250818_at	AT5G04930	255982_at	AT1G34000
250493_at	AT5G09800	256902_s_at	AT3G23910
251689_at	AT3G56500		AT3G24255
248770_at	AT5G47740	257628_at	AT3G26290
253780_at	AT4G28400	259248_at	AT3G07770
250629_at	AT5G07390	259125_at	AT3G02300
251422_at	AT3G60540	257933_at	AT3G25585
252338_at	AT3G48890	253387_at	AT4G33010
253768_at	AT4G28550	253235_at	AT4G34350
249415_at	AT5G39660	258977_s_at	AT3G02020
253784_at	AT4G28610		AT5G14060
251984_at	AT3G53260	252337_at	AT3G48750
252076_at	AT3G51660	255953_at	AT1G22070
250201_at	AT5G14230	258048_at	AT3G16290
250798_at	AT5G05340	251324_at	AT3G61430
251797_at	AT3G55560	253662_at	AT4G30080
253827_at	AT4G28085	253392_at	AT4G32650
249485_at	AT5G39020	259379_at	AT3G16350
253060_at	AT4G37710	253609_at	AT4G30190
251603_at	AT3G57760	258705_at	AT3G09470
248980_at	AT5G45090	259034_at	AT3G09410
253614_at	AT4G30350	251666_at	AT3G57050
250850_at	AT5G04550	259460_at	AT1G44000
249171_at	AT5G42940	254275_at	AT4G22670
253485_at	AT4G31800	253562_at	AT4G31130
253503_at	AT4G31950	251331_s_at	AT3G61650
249474_s_at	AT5G39160		AT5G05620
	AT5G39190	253701_at	AT4G29890
	AT5G39130	251251_at	AT3G62220
252736_at	AT3G43210	259334_at	AT3G03790
249057_at	AT5G44480	253712_at	AT4G29330
251279_at	AT3G61800	258958_at	AT3G01390
252383_at	AT3G47780	251665_at	AT3G57040
250185_at	AT5G14310	254280_at	AT4G22756
249889_at	AT5G22540	251879_at	AT3G54200
248799_at	AT5G47230	258485_at	AT3G02630
251643_at	AT3G57550	258825_at	AT3G07180
250291_at	AT5G13280	258710_s_at	AT4G08520
252515_at	AT3G46230		AT3G09800
252827_at	AT4G39950	256253_at	AT3G11250
251334_at	AT3G61390	251466_at	AT3G59340
253898_s_at	AT5G54810	251596_at	AT3G57650
	AT4G27070	251958_at	AT3G53560
253796_at	AT4G28460	255348_at	AT4G03820
249252_at	AT5G42010	254361_at	AT4G22212
249490_s_at	AT5G39110	255752_at	AT1G32050
	AT5G39150	251881_at	AT3G54250
	AT5G39120	255559_at	AT4G02010
	AT5G39180	257608_at	AT3G13860
251773_at	AT3G55960	259302_at	AT3G05120
250695_at	AT5G06740	258736_at	AT3G05900
251479_at	AT3G59700	259143_at	AT3G10190
249325_at	AT5G40850	253474_at	AT4G32270
253958_at	AT4G26400	253172_at	AT4G35060
253779_at	AT4G28490	259221_s_at	AT3G03530
248769_at	AT5G47730		AT3G03540
252437_at	AT3G47380	253174_at	AT4G35090

252222_at	AT3G49845	256021_at	AT1G58270
252533_at	AT3G46110	253296_at	AT4G33770
249025_at	AT5G44720	259365_at	AT1G13300
249494_at	AT5G39050	254656_at	AT4G18070
252421_at	AT3G47540	258775_at	AT3G10850
249890_at	AT5G22570	251931_at	AT3G53950
251722_at	AT3G56200	255236_at	AT4G05520
252422_at	AT3G47550	251962_at	AT3G53420
249705_at	AT5G35580	254520_at	AT4G19960
252592_at	AT3G45640	252023_at	AT3G52920
251336_at	AT3G61190	254515_at	AT4G20270
253111_at	AT4G35940	256073_at	AT1G18100
252417_at	AT3G47480	252350_at	AT3G48190
249032_at	AT5G44910	256836_at	AT3G22960
252303_at	AT3G49210	254392_at	AT4G21600
250702_at	AT5G06730	255926_at	AT1G22190
253147_at	AT4G35600	258163_at	AT3G17940
252278_at	AT3G49530	258038_at	AT3G21260
250323_at	AT5G12880	252698_at	AT3G43670
249586_at	AT5G37840	254684_at	AT4G13850
252652_at	AT3G44720	256340_at	AT1G72070
253327_at	AT4G33450	259431_at	AT1G01620
251910_at	AT3G53810	255718_at	AT1G32070
252334_at	AT3G48850	257299_at	AT3G28050
263472_at	AT2G31955	258622_at	AT3G02720
267548_at	AT2G32660	255829_at	AT2G40540
263274_at	AT2G11520	253545_at	AT4G31310
262605_at	AT1G15170	257580_at	AT3G06210
258907_at	AT3G06370	254290_at	AT4G23000
261476_at	AT1G14480	255332_at	AT4G04340
260592_at	AT1G55850	260385_at	AT1G74090
260924_at	AT1G21590	262797_at	AT1G20840
261394_at	AT1G79680	266802_at	AT2G22900
257264_at	AT3G22060	262796_at	AT1G20850
256833_at	AT3G22910	261740_at	AT1G47740
260833_at	AT1G06800	261131_at	AT1G19835
261690_at	AT1G50090	261040_at	AT1G17370
257038_at	AT3G19260	267553_s_at	AT2G32180
260971_at	at1g53620		AT2G32650
258614_at	AT3G02770	264338_at	AT1G70300
254249_at	AT4G23280	260714_at	AT1G14980
260387_at	AT1G74100	261749_at	AT1G76180
260823_at	AT1G06770	265795_at	AT2G35780
266037_at	AT2G05940	266119_at	AT2G02100
263845_at	AT2G37040	261635_at	AT1G50020
257950_at	AT3G21780	263391_at	AT2G11810
263722_at	AT2G13650	265405_at	AT2G16750
256351_at	AT1G54960	261549_at	AT1G63470
258201_at	AT3G13910	260033_at	AT1G68760
257206_at	AT3G16530	267516_at	AT2G30520
264660_at	AT1G09940	260949_at	AT1G06110
260477_at	AT1G11050	262736_at	AT1G28570
266615_s_at	AT2G35660	264209_at	AT1G22740
	AT2G29720	265066_at	AT1G03870
265539_at	AT2G15830	264199_at	AT1G22700
261445_at	AT1G28380	266057_at	AT2G40660
263402_at	AT2G04050	262304_at	AT1G70890
254256_at	AT4G23180	266413_at	AT2G38740
264757_at	AT1G61360	263957_at	AT2G35880
263228_at	AT1G30700	267562_at	AT2G39670
257235_at	AT3G15060	261814_at	AT1G08310
264923_s_at	AT1G65970	260036_at	AT1G68830
	AT1G60740	264371_at	AT1G12090
262745_at	AT1G28600	267191_at	AT2G44110
258915_at	AT3G10640	260028_at	AT1G29980
261179_at	AT1G04985	260856_at	AT1G21910
260943_at	AT1G45145	264581_at	AT1G05210
258682_at	AT3G08720	264988_at	AT1G27140
257348_at	AT2G42140	266941_at	AT2G18980
262542_at	AT1G34180	265807_at	AT2G17990
258493_at	AT3G02555	259964_at	AT1G53680
261618_at	AT1G33110	265804_at	AT2G18030
265244_at	AT2G43018	266649_at	AT2G25810
	AT2G43020	261924_at	AT1G22550
265583_at	AT2G20010	267595_at	AT2G32990
258908_at	AT3G06350	266165_at	AT2G28190
254204_at	AT4G24160	264070_at	AT2G27960
257277_at	AT3G14470	263473_at	AT2G31750
254975_at	AT4G10500	259596_at	AT1G28130
259211_at	AT3G09020	267172_at	AT2G37660
258746_at	AT3G05950	264317_at	AT1G70310
266071_at	AT2G18680	261576_at	AT1G01070
	AT2G18670	265250_at	AT2G01950
267623_at	AT2G39650	263865_at	AT2G36910
265385_at	AT2G20900	262725_at	AT1G43580
256972_at	AT3G21140	261415_at	AT1G07750
259410_at	AT1G13340	263636_at	AT2G11910
254321_at	AT4G22590	259939_s_at	AT1G71270
	AT4G22592		AT1G71300
266609_at	AT2G35510	265387_at	AT2G20670
263565_at	AT2G15390	264301_at	AT1G78780
259134_at	AT3G05390	266262_at	AT2G27590
261405_at	AT1G18740	263628_at	AT2G04780
261933_at	AT1G22410	265159_at	AT1G31060
266186_at	AT2G38960	266131_at	AT2G45160
265333_at	AT2G18350	265214_at	AT1G05000
261020_at	AT1G26390	263225_at	AT1G30650
253987_at	AT4G26270	263979_at	AT2G42840
266983_at	AT2G39400	264331_at	AT1G04130
257828_at	AT3G26670	259878_at	AT1G76790
255151_at	AT4G08180	261500_at	AT1G28400
256835_at	AT3G22890	260074_at	AT1G73640
265062_at	AT1G61550	264262_at	AT1G09200
267084_at	AT2G41180	267103_at	AT2G41490
261551_at	AT1G63440	262685_at	AT1G76010
258173_at	AT3G21630	259617_at	AT1G47970
254784_at	AT4G12720	266353_at	AT2G01520
263866_at	AT2G36950	266892_at	AT2G26080
266360_at	AT2G32250	260857_at	AT1G21880
260556_at	AT2G43620	263319_at	AT2G47160
260005_at	AT1G67920	259771_at	AT1G29470
256793_at	AT3G22160	267556_at	AT2G32810
255479_at	AT4G02380	267457_at	AT2G33790
259330_at	AT3G16270	264916_at	AT1G60810
257902_at	AT3G28450	260704_at	AT1G32470
255845_at	AT2G33600	266684_at	AT2G19720
263478_at	AT2G31880	266566_at	AT2G24040
255411_at	AT4G03110	267130_at	AT2G23390
254432_at	AT4G20830	261073_at	AT1G07300
254409_at	AT4G21400	260007_at	AT1G67870
254710_at	AT4G18050	263595_at	AT2G01890
267496_at	AT2G30550	263047_at	AT2G17630
258833_at	AT3G07274	261078_at	AT1G07320
256129_at	AT1G18210	259665_at	AT1G55160
267391_at	AT2G44480	266865_at	AT2G29980
259987_at	AT1G75030	264577_at	AT1G05260
266106_at	AT2G45170	266689_at	AT2G19930
262769_at	AT1G13180	266874_at	AT2G44760
261109_at	AT1G75450	261859_at	AT1G50490
266474_at	AT2G31110	260137_at	AT1G66330
259694_at	AT1G63180	262164_at	AT1G78070
262930_at	AT1G65690	262871_at	AT1G65010

257844_at	AT3G28480	262051_at	AT1G79990
264841_at	AT1G03740	262346_at	AT1G63980
255339_at	AT4G04480	263456_at	AT2G22125
261021_at	AT1G26380	266687_at	AT2G19670
260581_at	AT2G47190	260281_at	AT1G80500
266976_at	AT2G39410	261421_at	AT1G18840
265093_at	AT1G03905	262880_at	AT1G64880
262303_at	AT1G70920	266331_at	AT2G01570
261475_at	AT1G14550	265160_at	AT1G31050
256050_at	AT1G07000	262195_at	AT1G78040
262360_at	AT1G73080	265858_at	AT2G01720
262518_at	AT1G17170	260014_at	AT1G68010
260648_at	AT1G08050	261806_at	AT1G30510
263972_at	AT2G42760	260482_at	AT1G10950
256178_s_at	AT1G51760	267423_at	AT2G35060
	AT1G51780	264692_at	AT1G70000
262507_at	AT1G11330	266223_at	AT2G28790
257745_at	AT3G29240	262239_at	AT1G48230
263221_at	AT1G30620	263574_at	AT2G16990
265070_at	AT1G55510	263900_at	AT2G36290
260394_at	AT1G74080	264664_at	AT1G09660
259552_at	AT1G21320	267640_at	AT2G32950
265885_at	AT2G42330	266418_at	AT2G38750
261216_at	AT1G33030	264462_at	AT1G10200
266000_at	AT2G24180	264028_at	AT2G03680
259876_at	AT1G76700	264572_at	At1g05320
255605_at	AT4G01090	259954_at	AT1G75130
259369_s_at	AT3G43890	259983_at	AT1G76490
	AT1G69150	260950_s_at	AT1G06120
262461_at	AT1G50360		AT1G06090
257927_at	AT3G23240	261080_at	AT1G07370
258786_at	AT3G11820	264031_at	AT2G03780
259309_at	AT3G05050	261632_at	AT1G50120
259213_at	AT3G09010	260302_at	AT1G80310
263551_at	AT2G17110	264476_at	AT1G77130
261899_at	AT1G80820	266297_at	AT2G29570
261474_at	AT1G14540	266075_s_at	AT4G38290
264703_at	AT1G69960		AT2G40710
262772_at	AT1G13210		AT4G37680
261674_at	AT1G18270	260548_at	AT2G43360
255381_at	AT4G03510	261129_at	AT1G04820
266901_at	AT2G34600	265634_at	AT2G25530
257751_at	AT3G18690	266716_at	AT2G46820
262572_at	AT1G15140	265290_at	AT2G22590
260439_at	AT1G68340	265050_at	AT1G52070
267229_s_at	AT5G38640	264188_at	AT1G54690
	AT2G44070	266295_at	AT2G29550
258501_at	AT3G06780	264159_at	AT1G65270
267523_at	AT2G30600	263780_at	AT2G46340
257919_at	AT3G23250	260044_at	AT1G73655
257784_at	AT3G26980	267132_at	AT2G23420
265260_at	AT2G43000	263962_at	AT2G36350
255504_at	AT4G02200	260675_at	AT1G19430
263063_s_at	AT2G18150	261469_at	AT1G28340
	AT2G18140	266927_at	AT2G45960
262085_at	AT1G56060	262230_at	AT1G68560
263460_at	AT2G31810	259891_at	AT1G72730
266271_at	AT2G29440	266737_at	AT2G47140
264912_at	AT1G60750	265581_at	AT2G20050
266552_at	AT2G46330	260027_at	AT1G29952
254223_at	AT4G23730		AT1G29950
265136_at	AT1G51270		AT1G29951
262118_at	AT1G02850	267637_at	AT2G42190
259520_at	AT1G12320	264987_at	AT1G27030
264645_at	AT1G08940	263183_at	AT1G05570
262827_at	AT1G13100	261768_at	AT1G15550
263019_at	AT1G23870	263431_at	AT2G22170
263931_at	AT2G36220	260629_at	AT1G62330
254812_at	AT4G12250	266644_at	AT2G29660
261826_at	AT1G11580	262838_at	AT1G14960
256948_at	AT3G18930	264990_at	AT1G27210
261615_at	AT1G33050	262157_at	AT1G52670
260560_at	AT2G43590	261970_at	AT1G65960
264304_at	AT1G78895	267307_at	AT2G30210
263871_at	AT2G22010	267121_at	AT2G23540
260225_at	AT1G74590	260636_at	AT1G62430
267096_at	AT2G38180	265118_at	AT1G62660
259340_at	AT3G03870	262039_at	AT1G80050
264867_at	AT1G24150	259882_at	AT1G76670
265452_at	AT2G46510	266256_at	AT2G27710
267163_at	AT2G37520	266560_at	AT2G23950
256522_at	AT1G66160	264057_at	AT2G28550
264178_at	AT1G02170	263904_at	AT2G36380
255784_at	AT1G19860	261056_at	AT1G01360
259400_at	AT1G17750	265272_at	AT2G28350
256499_at	AT1G36640	264099_at	AT1G79050
256147_at	AT1G55080	263249_at	AT2G31360
259550_at	AT1G35230	262260_at	AT1G70850
264756_at	AT1G61370	263552_x_at	AT2G24980
260261_at	AT1G68450	263834_at	AT2G40316
260671_at	AT1G19310	263728_at	AT1G60070
262382_at	AT1G72920	263406_at	AT2G04160
256306_at	AT1G30370	260615_at	AT1G53240
267392_at	AT2G44490	262451_at	AT1G11130
257805_at	AT3G18830	263696_at	AT1G31230
254916_at	AT4G11340	265120_at	AT1G62640
267451_at	AT2G33710	264191_at	AT1G54730
258516_at	AT3G06490	264056_at	AT2G28510
264635_at	AT1G65500	260941_at	AT1G44970
263216_s_at	AT1G30720	263867_at	AT2G36830
	AT1G30730	259750_at	AT1G71130
259149_at	AT3G10340	260254_at	AT1G74210
255080_at	AT4G09030	260238_at	AT1G74520
254575_at	AT4G19460	267093_at	AT2G38170
266269_at	AT2G29480	261636_at	AT1G50110
260568_at	AT2G43570	259984_at	AT1G76460
266792_at	AT2G02860	259588_at	AT1G27930
258953_at	AT3G01430	261953_at	AT1G64440
259461_at	AT1G18900	265855_at	AT2G42390
257922_at	AT3G23150	263325_at	AT2G04240
261506_at	AT1G71697	259736_at	AT1G64390
256356_s_at	AT5G43620	261745_at	AT1G08500
	AT1G66500	262354_at	AT1G64200
259445_at	AT1G02400	262209_at	AT1G74720
254660_at	AT4G18250	265946_s_at	AT2G19520
261518_at	AT1G71695		AT4G29730
266816_at	AT2G44970	261428_at	AT1G18870
261242_at	AT1G32960	261076_at	AT1G07420
259952_at	AT1G71400	259653_at	AT1G55240
258463_at	AT3G17410	261170_at	AT1G04910
256081_at	AT1G20700	261479_at	AT1G14380
261525_at	AT1G14330	262301_at	AT1G70880
265482_at	AT2G15780	261273_at	AT1G26650
260367_at	AT1G69760	264680_at	AT1G65510
261448_at	AT1G21140	260331_at	AT1G80270
257124_at	AT3G20040	261900_at	AT1G80940
265132_at	AT1G23830	267261_at	AT2G23120
265530_at	AT2G06050	261579_at	AT1G01050
256547_at	AT3G14840	266118_at	AT2G02130
267028_at	AT2G38470	266001_at	AT2G24150
259734_at	AT1G77500	262608_at	AT1G14120
264777_at	AT1G08630	262842_at	AT1G14720

266267_at	AT2G29460	259860_at	AT1G80640
265221_s_at	AT2G02000	267619_at	AT2G26730
	AT2G02010	267592_at	AT2G39710
255967_at	AT1G22280	263412_at	AT2G28720
264627_at	AT1G65520	266035_at	AT2G05990
260847_s_at	AT1G17290	263619_at	AT2G04650
	AT1G72330	262400_at	AT1G49480
265572_at	AT2G28210	261189_at	AT1G33040
256627_at	AT3G19970	263882_at	AT2G21790
258615_at	AT3G02740	266574_at	AT2G23890
256576_at	AT3G28210	260501_at	AT2G41770
263147_at	AT1G53980	260549_at	AT2G43535
259626_at	AT1G42990	265319_at	AT2G22670
259507_at	AT1G43910	262577_at	AT1G15290
254905_at	AT4G11170	259570_at	AT1G20440
258002_at	AT3G28930	261727_at	AT1G76090
259033_at	AT3G09410	262978_at	AT1G75780
259216_at	AT3G09000	265183_at	AT1G23750
254410_at	AT4G21410	261700_at	AT1G32690
259975_at	AT1G76470	261228_at	AT1G20050
254158_at	AT4G24380	264903_at	AT1G23190
262026_at	AT1G35670	262853_at	AT1G20890
264746_at	AT1G62300	262753_at	AT1G16340
261006_at	AT1G26410	265576_at	AT2G20190
260668_at	AT1G19530	260590_at	AT1G53310
259421_at	AT1G13910	259871_at	AT1G76800
256321_at	AT1G55020	261886_s_at	AT1G80700
263919_at	AT2G36470		AT1G80980
267610_at	AT2G26650	266838_at	AT2G25980
262183_at	AT1G77890	265656_at	AT2G13820
255844_at	AT2G33580	261400_at	AT1G79630
256720_at	AT2G34140	263134_at	AT1G78570
258631_at	AT3G07970	261049_at	AT1G01430
265199_s_at	AT2G36780	261319_at	AT1G53090
	AT2G36770	266506_at	AT2G47850
256376_s_at	AT1G66690	266414_at	AT2G38700
	AT1G66700	264687_at	AT1G09850
258665_at	AT3G08710	262729_at	AT1G75840
255095_at	AT4G08500	266897_at	AT2G45820
255881_at	AT1G67070	264527_at	AT1G30760
264869_at	AT1G24350	261646_at	AT1G27690
266800_at	AT2G22880	267497_at	AT2G30540
265499_at	AT2G15480	259903_at	AT1G74160
260983_at	AT1G53560	264857_at	AT1G24170
261443_at	AT1G28480	259846_at	AT1G72140
264929_at	AT1G60730	266526_at	AT2G16980
264866_at	AT1G24140	266702_at	AT2G19860
267383_at	AT2G44360	262975_at	AT1G75540
261676_at	AT1G18480	262532_at	AT1G17210
266781_at	AT2G28940	262881_at	AT1G64890
254270_at	AT4G23100	263153_s_at	AT1G54000
266287_at	AT2G29150		AT1G54010
265450_at	AT2G46620	264372_at	AT1G11840
259557_at	AT1G21210	260619_at	AT1G08110
258692_at	AT3G08640	266099_at	AT2G38040
265536_at	AT2G15880	264523_at	AT1G10030
266017_at	AT2G18690	264728_at	AT1G22850
254396_at	AT4G21680	267264_at	AT2G22970
266428_at	AT2G07180	263333_at	AT2G03890
257632_at	AT3G26190	261509_at	AT1G71740
258063_at	AT3G14620	259978_at	AT1G76540
259474_at	AT1G19130	261363_at	AT1G41830
257621_at	AT3G20410	260895_at	AT1G29250
265796_at	AT2G35730	266205_s_at	AT1G08200
263182_at	AT1G05575		AT2G27860
265440_at	AT2G20960	264656_at	AT1G09010
267202_s_at	AT2G31020	267005_at	AT2G34460
	AT2G31030	261691_at	AT1G50060
262325_at	AT1G64160	266335_at	AT2G32440
256616_at	AT3G22260	264615_at	AT1G04590
254248_at	AT4G23270	265338_at	AT2G18400
266732_at	AT2G03240	261607_at	AT1G49660
264873_at	AT1G24100	263591_at	AT2G01910
264107_s_at	AT2G13790	262855_at	AT1G20860
	AT2G13800	262454_at	AT1G11190
262607_at	AT1G13990	261878_at	AT1G50560
267576_at	AT2G30640	259958_at	AT1G53730
266101_at	AT2G37940	262868_at	AT1G64980
259495_at	AT1G15890	260179_at	AT1G70690
256183_at	AT1G51660	266456_at	AT2G22770
265008_at	AT1G61560	262210_at	AT1G74690
260101_at	AT1G73260	262944_at	AT1G79550
256735_at	AT3G29400	260268_at	AT1G68490
264351_at	AT1G03370	267432_at	AT2G35020
257978_at	AT3G20860	264605_at	AT1G04550
262124_at	AT1G59660	260000_at	AT1G68060
260208_s_at	AT1G70670	267040_at	AT2G34300
	AT1G70680	266257_at	AT2G27820
267300_at	AT2G30140	261303_at	AT1G48620
262910_at	AT1G59710	263181_at	AT1G05720
255421_at	AT4G03260	259541_at	AT1G20650
267565_at	AT2G30750	267353_at	AT2G40070
265723_at	AT2G32140	267088_at	AT2G38140
262251_at	AT1G53760	263638_at	AT2G25310
263558_at	AT2G16380	264069_at	AT2G28000
264415_at	AT1G43160	262909_at	AT1G59830
254042_at	AT4G25810	261055_at	AT1G01300
265200_s_at	AT2G36800	260058_at	AT1G78100
	AT2G36790	264102_at	AT1G79270
260405_at	AT1G69930	263987_at	AT2G42690
260397_at	AT1G69680	262590_at	AT1G15100
267045_at	AT2G34180	265377_at	AT2G05790
258792_at	AT3G04640	263220_at	AT1G30610
264834_at	AT1G03730	260814_at	AT1G43710
254926_at	AT4G11280	264859_at	AT1G24280
266469_at	AT2G31180	263970_at	AT2G42850
255854_at	AT1G67050	261883_at	AT1G80870
258608_at	AT3G03020	259594_at	AT1G28140
257010_at	AT3G14090	262226_at	AT1G53885
262741_at	AT1G28760		AT1G53903
255753_at	AT1G18570	261728_at	AT1G76160
262640_at	AT1G62760	266848_at	AT2G25950
260370_at	AT1G69740	267611_at	AT2G26660
260932_s_at	AT1G02530	267308_at	AT2G30200
	AT1G02520	266489_at	AT2G35190
266326_at	AT2G46650	262135_at	AT1G78080
259434_at	AT1G01490	261796_at	AT1G30440
256664_at	AT3G12040	265884_at	AT2G42320
254069_at	AT4G25434	260653_at	AT1G32440
255733_at	AT1G25400	267339_at	AT2G39870
257185_at	AT3G13100	259591_at	AT1G28150
262899_at	AT1G59870	259564_at	AT1G20540
266964_at	AT2G39480	261044_at	AT1G01290
261979_at	AT1G37130	263036_at	AT1G23890
259792_at	AT1G29690	264926_at	AT1G60660
265732_at	AT2G01300	264850_at	AT2G17340
266116_at	AT2G02180	263004_at	AT1G54510
261378_at	AT1G18890	261456_at	AT1G21050
254082_at	AT4G25720	267184_at	AT2G44150
266364_at	AT2G41230	264438_at	AT1G27400
265155_at	AT1G30990	266359_at	AT2G32260
262244_at	AT1G48260	264584_at	AT1G05140

257785_at	AT3G26980	266820_at	AT2G44940
261429_at	AT1G18860	260570_at	AT2G43710
254040_at	AT4G25900	262094_at	AT1G56110
260248_at	AT1G74310	260676_at	AT1G19450
264561_at	AT1G55810	265439_at	AT2G21045
262165_at	AT1G75020	262954_at	AT1G54500
261748_at	AT1G76070	266588_at	AT2G14890
260522_x_at	AT2G41730	261663_at	AT1G18330
258602_at	AT3G02750	265929_s_at	AT2G18560
262047_at	AT1G80160		AT2G18570
266296_at	AT2G29420	266323_at	AT2G46700
263157_at	AT1G54100	263939_at	AT2G36070
261354_at	AT1G79690	261373_at	AT1G53000
259479_at	AT1G19020	266721_at	AT2G03220
256989_at	AT3G28580	260065_at	AT1G73760
263032_at	AT1G23850	261032_at	AT1G17430
257636_at	AT3G26200	261309_at	AT1G48598
264701_at	AT1G70160		AT1G48600
258971_at	AT3G01990	265512_at	AT2G05590
265713_at	AT2G03530	259680_at	AT1G77690
265395_at	AT2G20850	265856_at	AT2G42430
254063_at	AT4G25390	262751_at	AT1G16310
261763_at	AT1G15520	259754_at	AT1G71090
264647_at	AT1G09090	264054_at	AT2G22540
256633_at	AT3G28340	261120_at	AT1G75410
257071_at	AT3G28180	264802_at	AT1G08560
267314_at	AT2G34730	261945_at	AT1G64530
265306_at	AT2G20320	266575_at	AT2G24060
266693_at	AT2G19800	265169_x_at	AT1G23720
254894_at	AT4G11840	261337_at	AT1G44810
264809_at	AT1G08830	266735_at	AT2G46930
256328_at	AT3G02360	261410_at	AT1G07610
259481_at	AT1G18970	259713_at	AT1G77610
262279_at	AT1G68630	262315_at	AT1G70990
262381_at	AT1G72900	263285_at	AT2G36120
267147_at	AT2G38240	266975_at	AT2G39380
260651_at	AT1G32460	260427_at	AT1G72430
255110_at	AT4G08770	262615_at	AT1G13950
262171_at	AT1G74950	264675_at	AT1G09830
260468_at	AT1G11100	265588_at	AT2G19970
261005_at	AT1G26420	259804_at	AT1G72160
264379_at	AT2G25200	263386_at	AT2G40150
258282_at	AT3G26910	264893_at	AT1G23140
258855_at	AT3G02070	260758_at	AT1G48930
261023_at	AT1G12200	259758_s_at	AT1G77530
262515_at	AT1G34200		AT1G77520
264624_at	AT1G08930	261297_at	AT1G48500
259296_at	AT3G05350	261927_at	AT1G22500
266246_at	AT2G27690	265353_at	AT2G16800
262455_at	AT1G11310	259516_at	AT1G20450
264758_at	AT1G61340	261252_at	AT1G05810
260135_at	AT1G66400	260528_at	AT2G47260
254331_s_at	AT4G22710	260985_at	AT1G53500
	AT4G22690	263513_at	AT2G12400
254524_at	AT4G20000	263912_at	AT2G36390
263804_at	AT2G40270	263963_at	AT2G36080
254847_at	AT4G11850	263952_s_at	AT2G35830
255344_s_at	AT4G04570		AT2G35810
	AT4G04540	263491_at	AT2G42600
267134_at	AT2G23450	267517_at	AT2G30520
259749_at	AT1G71100	264497_at	AT1G30840
265721_at	AT2G40090	262943_at	AT1G79470
263776_s_at	AT2G46430	263288_at	AT2G36130
	AT2G46440	261926_at	AT1G22530
257868_at	AT3G25070	263786_at	AT2G46370
263150_at	AT1G54050	263662_at	AT1G04430
266580_at	AT2G46260	264824_at	AT1G03420
256922_at	AT3G19010	267494_at	AT2G30410
260608_at	AT2G43870	264254_at	AT1G09150
267112_at	AT2G14750	264078_at	AT2G28470
266658_at	AT2G25735	259525_at	AT1G12560
264736_at	AT1G62200	260271_at	AT1G63690
256756_at	AT3G25610	264250_at	AT1G78680
260820_at	AT1G06840	260527_at	AT2G47270
267289_at	AT2G23770	266015_at	AT2G24190
262512_at	AT1G17145	263995_at	AT2G22540
261973_at	AT1G64610	264567_s_at	AT1G05250
266884_at	AT2G44790		AT1G05240
261756_at	AT1G08315	261286_at	AT1G35780
261155_at	AT1G04960	262495_at	AT1G21780
263847_at	AT2G36970	262963_at	AT1G54220
257758_at	AT3G22980	267070_at	AT2G41000
266536_at	AT2G16900	266889_at	AT2G44640
264669_at	AT1G09630	260352_at	AT1G69295
259518_at	AT1G20510	260944_at	AT1G45130
265034_at	AT1G61660	263326_at	AT2G04280
256755_at	AT3G25600	262248_at	AT1G48370
264986_at	AT1G27130	265817_at	AT2G18050
260614_at	AT1G53390	266978_at	AT2G39430
263118_at	AT1G03090	266906_at	AT2G34585
267537_at	AT2G41880	261791_at	AT1G16170
265359_at	AT2G16720	267214_at	AT2G43970
256243_at	AT3G12500	266330_at	AT2G01530
256526_at	AT1G66090	266215_at	AT2G06850
261581_at	AT1G01140	267310_at	AT2G34680
262762_at	AT1G10700	262658_at	AT1G14220
261205_at	AT1G12790	266617_at	AT2G29670
254897_at	AT4G11470	263496_at	AT2G42570
256933_at	AT3G22600	262849_at	AT1G14710
265737_at	AT2G01180	265444_s_at	AT2G37180
267624_at	AT2G39660		AT2G37170
267485_at	AT2G02820	259803_at	AT1G72150
257184_at	AT3G13090	265818_at	AT2G18040
266486_at	AT2G47950	264729_at	AT1G22990
260232_at	AT1G74640	264574_at	AT1G05300
260536_at	AT2G43400	261139_at	AT1G19700
264951_at	AT1G76970	263098_at	AT2G16005
255568_at	AT4G01250	261667_at	AT1G18460
259428_at	AT1G01560	261803_at	AT1G30500
261052_at	AT1G01440	260875_at	AT1G21410
264820_at	AT1G03475	266123_at	AT2G45180
263461_at	AT2G31800	262399_at	AT1G49500
256285_at	AT3G12510	264344_at	AT1G11910
265327_at	AT2G18210	266770_at	AT2G03090
261027_at	AT1G01340	262883_at	AT1G64780
265470_at	AT2G37150	266517_at	AT2G35120
256046_at	no_match	261868_s_at	AT1G11450
256442_at	AT3G10930		AT1G11460
261526_at	AT1G14370	262951_at	AT1G75500
263379_at	AT2G40140	260879_at	AT1G21480
261934_at	AT1G22400	267498_at	AT2G45720
254120_at	AT4G24570	265663_at	AT2G24290
261606_at	AT1G49570	261258_at	AT1G26640
260784_at	AT1G06180	261944_at	AT1G64650
265192_at	AT1G05060	261285_at	AT1G35720
262119_s_at	AT1G02930	260616_at	AT1G53280
	AT1G02920	266158_at	AT2G28070
263429_at	AT2G22250	262166_at	AT1G74840
258606_at	AT3G02840	263494_at	AT2G42590
259619_at	AT1G07130	261335_at	AT1G44800
257840_at	AT3G25250	263352_at	AT2G22080
261261_at	AT1G26730	267159_at	AT2G37650

262649_at	AT1G14040	263537_at	AT2G24790
254788_at	AT4G12790	264611_at	AT1G04680
260276_at	AT1G80450	261138_at	AT1G19710
262228_at	AT1G68690	267577_at	AT2G30710
265620_at	AT2G27310	264111_at	AT2G13690
261718_at	AT1G18390	262024_at	AT1G35620
258338_at	AT3G16150	267356_at	AT2G39930
257643_at	AT3G25730	261099_at	AT1G62980
261481_at	AT1G14260	260465_at	AT1G10910
260030_at	AT1G68880	261769_at	AT1G76100
261938_at	AT1G22510	267059_at	AT2G32520
258108_at	AT3G23570	262434_at	AT1G47670
258481_at	AT3G02600	266641_at	AT2G35605
264328_at	AT1G04100	264207_at	AT1G22750
256012_at	AT1G19250	264316_at	AT1G70330
255595_at	AT4G01700	266669_at	AT2G29750
254364_at	AT4G22020	264526_at	AT1G10130
254681_at	AT4G18140	264913_at	AT1G60770
266995_at	AT2G34500	260165_at	AT1G79850
262072_at	AT1G59590	266495_at	AT2G07050
266835_at	AT2G29990	266356_at	AT2G32300
260881_at	AT1G21550	264238_at	AT1G54740
263241_at	AT2G16500	263656_at	AT1G04240
263502_s_at	ATMG00980	266532_at	AT2G16890
	AT2G07675	265935_at	AT2G19580
260975_at	AT1G53430	260214_at	AT1G74510
264229_at	AT1G67480	264185_at	AT1G54780
262383_at	AT1G72940	267001_at	AT2G34470
265417_at	AT2G20920	263998_at	AT2G22510
265170_at	AT1G23730	265283_at	AT2G20370
257700_at	AT3G12740	261639_at	AT1G50010
260900_s_at	AT1G21400	261794_at	AT1G16060
	AT5G34780	265111_at	AT1G62510
267230_at	AT2G44080	259688_at	AT1G63120
254922_at	AT4G11370	262284_at	AT1G68670
264725_at	AT1G22885	263016_at	AT1G23410
265711_at	AT2G03360	262523_at	AT1G17110
258677_at	AT3G08730	264579_at	AT1G05205
254857_at	AT4G12120	263599_at	AT2G01830
264279_s_at	AT1G78820	263553_at	AT2G16430
	AT1G78830	261157_at	AT1G34510
265511_at	AT2G05540	263779_at	AT2G46340
264543_at	AT1G55780	267606_at	AT2G26640
263539_at	AT2G24850	264318_at	AT1G04220
260064_at	AT1G73730	263673_at	AT2G04800
261402_at	AT1G79670	260530_at	AT2G47320
261413_at	AT1G07630	259625_at	AT1G42970
260988_at	AT1G53570	259664_at	AT1G55330
262177_at	AT1G74710	260055_at	AT1G78150
264083_at	AT2G31230	261105_at	AT1G63000
262901_at	AT1G59910	262277_at	AT1G68650
257830_at	AT3G26690	266422_at	AT2G38650
258084_at	AT3G26020	265961_at	AT2G37400
260403_at	AT1G69810	263432_at	AT2G22230
259512_at	AT1G12360	260547_at	AT2G43550
265670_s_at	AT2G32190	267358_at	AT2G39890
	AT2G32210	266307_at	AT2G27000
256017_at	AT1G19180	260967_at	AT1G12230
267319_at	AT2G34660	267405_at	AT2G33740
255794_at	AT2G33480	262504_at	AT1G21750
255740_at	AT1G25390	262877_at	AT1G64850
266166_at	AT2G28080	260181_at	AT1G70710
265433_at	AT2G20950	264342_at	AT1G12080
265674_at	AT2G32190	264474_s_at	AT5G38420
264485_at	AT1G77220		AT5G38430
266070_at	AT2G18660		AT5G38410
263807_at	AT2G04400		AT1G67090
262896_at	AT1G59820	261136_at	AT1G19600
254408_at	AT4G21390	264998_at	AT1G67330
254977_s_at	AT4G10530	263956_at	AT2G35940
	AT4G10520	265415_at	AT2G20890
259297_at	AT3G05360	260902_at	AT1G21440
264800_at	AT1G08800	266514_at	AT2G47890
263403_at	AT2G04040	262831_at	AT1G14730
256451_s_at	AT1G75170	263151_at	AT1G54120
255543_at	AT4G01870	263236_at	AT1G10470
258537_at	AT3G04210	263449_at	AT2G31670
260697_at	AT1G32530	264122_at	AT1G02130
257061_at	AT3G18250	262947_at	AT1G75750
259511_at	AT1G12520	265330_at	AT2G18440
262911_s_at	AT1G59860	262637_at	AT1G06640
	AT1G07400	265102_at	AT1G30870
259502_at	AT1G15670	260337_at	AT1G69310
262671_at	AT1G76040	263679_at	AT1G59990
265289_at	AT2G22690	261790_at	AT1G16000
254103_at	AT4G25030	264657_at	AT1G09100
259864_at	AT1G72800	266635_at	AT2G35470
255624_at	AT4G01370	264845_at	AT1G03680
255880_at	AT1G67060	266920_at	AT2G45750
266142_at	AT2G39030	262784_at	AT1G10760
264052_at	AT2G22330	262446_at	AT1G49310
261892_at	AT1G80840	267135_at	AT2G23430
256583_at	AT3G28850	266368_at	AT2G41380
256380_at	AT1G66680	260089_at	AT1G73170
258623_at	AT3G02790	259755_at	AT1G71070
262832_s_at	AT1G14870	267092_at	AT2G38120
	AT1G14880	261014_at	AT1G26460
259312_at	AT3G05200	266861_at	AT2G26830
259517_at	AT1G20630	267591_at	AT2G39705
267567_at	AT2G30770	264607_at	AT1G04690
256262_at	AT3G12150	260686_at	AT1G17620
261719_at	AT1G18380	260230_at	AT1G74500
265618_at	AT2G25460	261065_at	AT1G07500
258683_at	AT3G08760	259863_at	AT1G72630
265495_at	AT2G15695	266065_at	AT2G18790
266557_at	AT2G46080	260682_at	AT1G17510
257088_at	AT3G20510	262402_at	AT1G49410
264716_at	AT1G70170	263373_at	AT2G20515
258577_at	AT3G04220	266419_at	AT2G38760
262082_s_at	AT1G56140	261982_at	AT1G33780
	AT1G56130	260151_at	AT1G52910
	AT1G56120	266701_at	AT2G19760
264299_s_at	AT1G78850	266703_at	AT2G19880
	AT1G78860	263880_at	AT2G21960
262444_at	AT1G47480	262626_at	AT1G06430
260804_at	AT1G78410	266079_at	AT2G37860
263075_at	AT2G17570	260347_at	AT1G69420
255252_at	AT4G04990	266827_at	AT2G22920
255773_at	AT1G18590	264637_at	AT1G65560
266712_at	AT2G46750	261279_at	AT1G05850
255879_at	AT1G67000	261562_at	AT1G01750
254032_at	AT4G25940	267031_at	AT2G38420
264463_at	AT1G10150	260390_at	AT1G73940
258507_at	AT3G06500	262575_at	AT1G15210
267142_at	AT2G38290	262969_at	AT1G75710
266231_at	AT2G02220	264098_at	AT1G79260
266294_at	AT2G29500	261559_at	AT1G01780
264588_at	AT2G17730	261141_at	AT1G19740
258787_at	AT3G11840	264228_at	AT1G67490
260919_at	AT1G21520	266125_at	AT2G45050
265597_at	AT2G20142	261553_at	AT1G63420
259737_at	AT1G64400	260130_s_at	AT1G66280

257829_at	AT3G26680		AT1G66270
257062_at	AT3G18290	262516_at	AT1G17190
254416_at	AT4G21380	265028_at	AT1G24530
260496_at	AT2G41700	263170_at	AT1G03000
267389_at	AT2G44460	260667_at	AT1G19440
266270_at	AT2G29470	260991_at	AT1G12160
258636_at	AT3G07890	260325_at	AT1G63940
265920_s_at	AT2G15120	263284_at	AT2G36100
	AT2G15220	260144_at	AT1G71960
266624_s_at	AT1G32380	262666_at	AT1G14080
	AT2G35390	267355_at	AT2G39900
259499_at	AT1G15730	260774_at	AT1G78290
267199_at	AT2G30990	266905_at	AT2G34560
264314_at	AT1G70420	262427_s_at	AT1G47600
259710_at	AT1G77670		AT1G51470
267524_at	AT2G30600	263758_s_at	AT2G21260
265772_at	AT2G48010		AT2G21250
260741_at	AT1G15040	260324_at	AT1G63970
258277_at	AT3G26830	259854_at	AT1G72200
260974_at	AT1G53440	267175_s_at	AT2G37620
261215_at	AT1G32970		AT3G53750
258350_at	AT3G17510	259883_at	AT1G76400
253963_at	AT4G26470	267235_at	AT2G43945
263714_at	AT2G20610	262937_at	AT1G79560
259743_at	AT1G71140	265049_at	AT1G52060
264130_at	AT1G79160	260489_at	AT1G51610
256117_at	AT1G16840	267060_at	AT2G32580
256567_at	AT3G19553	260056_at	AT1G78140
260492_at	AT2G41850	263227_at	AT1G30750
261193_at	AT1G32920	259576_at	AT1G35330
260046_at	AT1G73805	264394_at	AT1G11860
266182_at	AT2G02370	264668_at	AT1G09780
264772_at	AT1G22930	264790_at	AT2G17820
258275_at	AT3G15760	263664_at	AT1G04250
258075_at	AT3G25900	266939_at	AT2G18960
260406_at	AT1G69920	263831_at	AT2G40300
254878_at	AT4G11660	264466_at	AT1G10380
264433_at	AT1G61810	264532_at	AT1G55740
260415_at	AT1G69790	262287_at	AT1G68660
263198_at	AT1G53990	263483_at	AT2G04030
262093_at	AT1G56145	259678_at	AT1G77750
264223_s_at	AT3G16030	264315_at	AT1G70370
266168_at	AT2G38870	259627_at	AT1G42960
262488_at	AT1G21830	266314_at	AT2G27040
264553_s_at	AT1G09480	259592_at	AT1G27950
	AT1G09490	264521_at	AT1G10020
254014_at	AT4G26120	262600_at	AT1G15340
254380_at	AT4G21865	266303_at	AT2G27060
260408_at	AT1G69880	262105_at	AT1G02810
254241_at	AT4G23190	260882_at	AT1G29280
255074_at	AT4G09100	261165_at	AT1G34430
263457_at	AT2G22300	267500_s_at	AT2G45510
261668_at	AT1G18500		AT2G44890
257363_at	AT2G45760	263788_at	AT2G24580
265075_at	AT1G55450	267459_at	AT2G33850
263541_at	AT2G24860	261114_at	AT1G75390
265679_at	AT2G32240		AT1G75388
263948_at	AT2G35980	262313_at	AT1G70900
265479_at	AT2G15760	264979_s_at	AT1G27170
259178_at	AT3G01650		AT1G27180
264832_at	AT1G03660	267211_at	AT2G44065
260706_at	AT1G32350	267188_at	AT2G44050
266273_at	AT2G29410	260453_s_at	AT1G72510
261815_at	AT1G08320		AT2G09970
255872_at	AT2G30360	263534_at	AT2G24945
256574_at	AT3G14780		AT2G24940
258259_s_at	AT3G26820	263942_at	AT2G35860
	AT3G26840	262806_at	AT1G20950
254605_at	AT4G18950	261931_at	AT1G22430
259558_at	AT1G21230	266533_s_at	AT2G16850
261050_at	AT1G01260		AT4G35100
266476_at	AT2G31090	264653_at	AT1G08980
267083_at	AT2G41100	266289_at	AT2G29390
259076_at	AT3G02140	260494_at	AT2G41820
266908_at	AT2G34650	266968_at	AT2G39360
263783_at	AT2G46400	263276_at	AT2G14100
254610_at	AT4G18890	265570_at	AT2G28310
256291_at	AT3G12200	262850_at	AT1G14920
259852_at	AT1G72280	266928_at	AT2G45790
262571_at	AT1G15430	262247_at	AT1G48420
265204_at	AT2G36650	264348_at	AT1G12110
264380_at	AT2G25190	265464_at	AT2G37080
258436_at	AT3G16720	260235_at	AT1G74560
255599_at	AT4G01010	261268_at	AT1G26740
256366_at	AT1G66880	264910_at	AT1G61100
264774_at	AT1G22890	260511_at	AT1G51570
264460_at	AT1G10170	260565_at	AT2G43800
261033_at	AT1G17380	266911_at	AT2G45910
259488_at	AT1G15780	267127_at	AT2G23610
265685_at	AT2G24430	265059_at	AT1G52080
265194_at	AT1G05010	266184_s_at	AT3G54700
256799_at	AT3G18560		AT2G38940
266167_at	AT2G38860	264144_at	AT1G79320
265849_at	AT2G35736	266599_at	AT2G46100
259764_at	AT1G64280	261956_at	AT1G64590
261240_at	AT1G32940	264793_at	AT1G08660
264751_at	AT1G23020	259723_at	AT1G60960
254231_at	AT4G23810	267343_at	AT2G44260
265461_at	AT2G46500	264575_at	AT1G05190
258791_at	AT3G04720	265339_at	AT2G18230
259019_at	AT3G07370	259545_at	AT1G20560
265680_at	AT2G32150	266957_at	AT2G34640
266265_at	AT2G29340	266336_at	AT2G32270
256958_at	AT3G13430	264131_at	AT1G79150
260429_at	AT1G72450	261201_at	AT1G12850
267411_at	AT2G34930	266022_at	AT2G05920
266100_at	AT2G37980	261825_at	AT1G11545
262374_s_at	AT1G72910	263132_at	AT1G78560
	AT1G72930	263594_at	AT2G01880
260239_at	AT1G74360	261149_s_at	AT1G19570
259316_at	AT3G01175		AT1G19550
261771_at	AT1G76150	261999_at	AT1G33800
257583_at	AT1G66480	263499_at	AT2G42580
262782_at	AT1G13195	266325_at	AT2G46630
267381_at	AT2G26190	266109_at	AT2G37890
254292_at	AT4G23030	261230_at	AT1G20010
266711_at	AT2G46740	262473_at	AT1G50250
256891_at	AT3G19030	266578_at	AT2G23910
259997_at	AT1G67880	263318_at	AT2G24762
255284_at	AT4G04610	266857_at	AT2G26900
259442_at	AT1G02310	262980_at	AT1G75680
264365_s_at	AT1G03220	265664_at	AT2G24420
	AT1G03230	265048_at	AT1G52050
256058_at	AT1G07240		
260662_at	AT1G19540		
264082_at	AT2G28570		
258351_at	AT3G17700		
259856_at	AT1G68440		
262229_at	AT1G68620		
261470_at	AT1G28370		
261534_at	AT1G01820		
264419_s_at	AT1G43310		

	AT5G33320
264280_at	AT1G61820
265501_at	AT2G15490
266880_at	AT2G44770
258117_at	AT3G14700
256763_at	AT3G16860
259230_at	AT3G07780
264953_at	AT1G77120
262314_at	AT1G70810
258975_at	AT3G01970
258401_at	AT3G15430
267470_at	AT2G30490
260401_at	AT1G69840
254741_s_at	AT4G13920
	AT4G13900
260845_at	AT1G17310
261453_at	AT1G21130
265184_at	AT1G23710
262259_s_at	AT1G53870
	AT1G53890
260035_at	AT1G68850
254898_at	AT4G11480
255923_at	AT1G22180
258287_at	AT3G15990
266746_s_at	AT2G02930
	AT4G02520
259911_at	AT1G72680
267559_at	AT2G45570
262237_at	AT1G48320
259439_at	AT1G01480
259879_at	AT1G76650
258650_at	AT3G09830
260190_at	AT1G67560
261648_at	AT1G27730
254010_at	AT4G26240
263165_at	AT1G03060
265276_at	AT2G28400
254571_at	AT4G19370
266685_at	AT2G19710
260206_at	AT1G70740
260290_at	AT1G80510
260143_at	AT1G71880
263072_at	AT2G17530
262114_at	AT1G02860
263305_at	AT2G01930
267154_at	AT2G30870
250983_at	AT5G02780
249550_at	AT5G38210
252976_s_at	AT4G38550
252014_at	AT3G52870
250018_at	AT5G18150
251809_at	AT3G55600
249393_at	AT5G40170
251148_at	AT3G63180
