# Supplementary material for: Transcriptome dynamic of Arabidopsis roots infected with Phytophthora parasitica identifies VQ29, a gene induced during the penetration and involved in the restriction of infection
Source: PLoS One. 2017 Dec 27;12(12):e0190341. doi: 10.1371/journal.pone.0190341 (PMC5744986; doi:10.1371/journal.pone.0190341)
Supplement: S4 Table — The RT-qPCR profile and Affymetrix signal are given for each gene. NI, RNA isolated from non-inoculated roots. RNA isolated from roots 2.5 hai, 6 hai, 10.5 hai and 30 hai with Phytophthora parasitica. RT-qPCR data are presented as the value of the 2 independent replicates for each time point. Transcript levels were normalized with respect to those for At5g11770 and At5g62050 determined for the same samples. For normalized Affymetrix signals, the values of the 2 independent replicates are indicated. Hai, hours after infection. (PDF) [file pone.0190341.s008.pdf]

S4 Table : Validation of microarray data by RT-qPCR.

ARRAY DATA

gene ID	Cluster	NI rep. 1	NI rep. 2	NI rep. mean	2.5 hai rep. 1	2.5 hai rep. 2	2.5 hai rep. mean	2.5 hai Relative fold change 2,5hai/NI	6 hai rep. 1	6 hai rep. 2	6 hai rep. mean	6 hai Relative fold change 6hai/NI	10.5 hai rep. 1	10.5 hai rep. 2	10.5 hai rep. mean	10.5 hai Relative fold change 10.5hai/NI	30 hai rep. 1	30 hai rep. 2	30 hai rep. mean	30 hai Relative fold change 30hai/NI
AT2G44370	I	28,5	81,0	54,7	2328,1	2113,5	2328,1	42,5	263,5	765,6	263,5	4,8	165,2	287,0	226,1	4,1	7,4	47,2	27,3	-2,0
AT5G64120	I	807,7	510,3	659,0	3803,4	3997,9	3803,4	5,8	647,3	2569,6	647,3	-1,0	1064,4	1029,8	1047,1	1,6	96,1	337,0	216,6	-3,0
AT5G40590	I	383,5	66,2	224,9	5079,6	3339,1	5079,6	22,6	946,3	1061,3	946,3	4,2	1388,9	386,5	887,7	3,9	182,6	274,1	228,3	1,0
AT3G02240	I	69,2	66,8	68,0	1404,6	970,8	1404,6	20,6	289,9	370,1	289,9	4,3	289,7	128,5	209,1	3,1	29,1	79,7	54,4	-1,2
AT5G11920	I	107,2	78,2	92,7	1909,0	686,7	1909,0	20,6	692,5	662,0	692,5	7,5	553,2	403,7	478,4	5,2	350,4	347,7	349,0	3,8
AT1G29020	I	77,2	21,6	49,4	955,9	99,2	955,9	19,4	84,3	24,2	84,3	1,7	33,1	22,0	27,5	-1,8	17,0	28,9	22,9	-2,2
AT1G11540	I	99,9	54,7	77,3	1015,4	210,1	1015,4	13,1	141,0	94,9	141,0	1,8	286,6	62,7	174,6	2,3	71,0	36,7	53,9	-1,4
AT3G07350	II	1657,7	1951,8	1804,8	694,6	380,8	694,6	-2,6	1597,0	961,2	1597,0	-1,1	1271,1	949,4	1110,3	-1,6	1239,4	1587,0	1413,2	-1,3
AT3G20340	II	157,3	1083,2	620,3	122,2	249,2	122,2	-5,1	104,8	347,9	104,8	-5,9	244,3	1008,4	626,4	1,0	422,1	800,1	611,1	-1,0
AT5G67480	II	215,4	1317,9	766,7	126,3	390,9	126,3	-6,1	298,8	797,6	298,8	-2,6	246,9	572,9	409,9	-1,9	302,2	531,0	416,6	-1,8
AT4G38470	II	976,9	2763,5	1870,2	694,5	607,3	694,5	-2,7	2726,8	2887,8	2726,8	1,5	3498,9	3421,6	3460,3	1,9	1887,5	3782,7	2835,1	1,5
AT4G03960	II	551,5	624,3	587,9	273,3	161,5	273,3	-2,2	582,9	237,4	582,9	-1,0	511,8	361,2	436,5	-1,3	618,4	716,7	667,6	1,1
AT1G03850	II	1011,3	1491,1	1251,2	566,4	483,5	566,4	-2,2	1628,9	1465,0	1628,9	1,3	1855,0	1663,5	1759,2	1,4	2124,5	2434,6	2279,5	1,8
AT5G18680	II	523,1	1074,3	798,7	306,1	415,5	306,1	-2,6	330,0	550,5	330,0	-2,4	407,9	489,8	448,9	-1,8	418,1	423,6	420,9	-1,9
AT2G23270	III	11,5	11,5	11,5	2037,7	1659,9	2037,7	177,6	3686,4	3780,6	3686,4	321,3	3376,3	3611,7	3494,0	304,5	1521,1	1687,8	1604,4	139,8
AT5G60760	III	7,7	7,9	7,8	344,6	61,8	344,6	44,1	971,9	412,2	971,9	124,5	927,3	187,0	557,1	71,4	274,7	167,5	221,1	28,3
AT1G30135	III	8,0	10,5	9,3	66,1	25,1	66,1	7,1	1138,1	276,4	1138,1	122,7	1486,0	122,8	804,4	86,7	333,5	158,2	245,9	26,5
AT3G02800	III	19,9	23,5	21,7	940,8	487,9	940,8	43,3	1932,8	1381,6	1932,8	89,1	1320,1	1642,3	1481,2	68,2	989,1	1195,8	1092,5	50,3
AT2G37430	III	11,1	10,8	11,0	630,7	395,8	630,7	57,4	217,3	365,4	217,3	19,8	246,9	382,7	314,8	28,6	103,1	239,5	171,3	15,6
AT1G05880	III	6,8	12,4	9,6	502,7	313,0	502,7	52,2	155,2	427,6	155,2	16,1	241,9	352,9	297,4	30,9	131,7	185,4	158,5	16,5
AT4G22640	III	46,1	10,5	28,3	965,5	563,6	965,5	34,1	488,6	1350,5	488,6	17,2	612,1	848,1	730,1	25,8	166,7	346,5	256,6	9,1
AT3G22840	IV	2463,0	1124,6	1793,8	1493,0	5031,6	1493,0	-1,2	231,9	319,6	231,9	-7,7	30,3	20,9	25,6	-70,1	366,1	381,8	373,9	-4,8
AT2G05070	IV	300,7	559,4	430,1	128,6	1487,0	128,6	-3,3	49,7	395,6	49,7	-8,6	4,9	8,1	6,5	-66,1	26,8	195,2	111,0	-3,9
AT3G47500	IV	1134,8	987,2	1061,0	503,5	1525,4	503,5	-2,1	155,1	402,3	155,1	-6,8	12,4	42,4	27,4	-38,7	277,4	485,3	381,3	-2,8
AT5G64170	IV	599,7	425,7	512,7	257,9	757,7	257,9	-2,0	146,2	205,2	146,2	-3,5	13,3	14,1	13,7	-37,5	304,6	285,0	294,8	-1,7
AT5G07030	IV	2050,0	460,7	1255,4	1001,8	237,6	1001,8	-1,3	111,2	164,2	111,2	-11,3	75,5	11,0	43,3	-29,0	79,0	172,5	125,8	-10,0
AT1G73220	IV	4782,2	2063,1	3422,6	1177,1	1194,6	1177,1	-2,9	198,9	88,2	198,9	-17,2	425,9	34,0	230,0	-14,9	430,3	718,2	574,3	-6,0
AT5G52310	IV	173,7	1231,5	702,6	91,4	158,5	91,4	-7,7	46,1	400,0	46,1	-15,2	37,5	21,5	29,5	-23,8	49,0	603,2	326,1	-2,2
AT5G44400	V	693,4	514,2	603,8	474,2	431,6	474,2	-1,3	735,9	862,4	735,9	1,2	1259,6	1005,9	1132,8	1,9	2629,5	6185,3	4407,4	7,3
AT5G53970	V	33,5	122,3	77,9	64,6	152,3	64,6	-1,2	67,1	139,8	67,1	-1,2	104,6	197,8	151,2	1,9	153,1	705,8	429,5	5,5
AT1G02220	V	177,4	473,3	325,4	151,6	563,5	151,6	-2,1	688,1	592,1	688,1	2,1	607,6	568,1	587,8	1,8	2197,6	1096,2	1646,9	5,1
AT4G21620	V	110,5	116,1	113,3	81,0	99,1	81,0	-1,4	242,7	165,8	242,7	2,1	241,8	161,2	201,5	1,8	410,6	644,0	527,3	4,7
AT5G02020	V	75,8	489,0	282,4	208,5	180,9	208,5	-1,4	464,0	435,2	464,0	1,6	342,8	616,8	479,8	1,7	304,9	1729,2	1017,0	3,6
AT2G43510	V	339,0	2248,3	1293,7	150,2	2288,7	150,2	-8,6	382,3	2617,9	382,3	-3,4	1883,6	2615,4	2249,5	1,7	4626,2	4458,6	4542,4	3,5
AT4G16660	V	205,6	114,0	159,8	160,3	147,4	160,3	1,0	266,4	215,7	266,4	1,7	228,5	340,1	284,3	1,8	544,3	515,2	529,8	3,3
AT1G80240	VI	605,1	89,8	347,4	785,5	73,0	785,5	2,3	510,1	97,9	510,1	1,5	359,4	84,8	222,1	-1,6	25,5	17,9	21,7	-16,0
AT3G26650	VI	98,2	1718,1	908,2	38,0	1222,8	38,0	-23,9	84,4	971,9	84,4	-10,8	78,3	841,7	460,0	-2,0	87,9	255,1	171,5	-5,3
AT5G65040	VI	568,6	633,3	601,0	660,4	526,7	660,4	1,1	315,8	382,0	315,8	-1,9	543,9	423,0	483,5	-1,2	112,5	168,5	140,5	-4,3
AT1G49600	VI	528,5	320,9	424,7	447,8	232,7	447,8	1,1	321,6	161,7	321,6	-1,3	220,4	223,6	222,0	-1,9	120,4	101,3	110,9	-3,8
AT4G17870	VI	1136,2	509,9	823,1	1759,1	681,2	1759,1	2,1	513,2	397,1	513,2	-1,6	712,2	240,1	476,2	-1,7	230,2	214,1	222,2	-3,7
AT2G34590	VI	643,9	456,3	550,1	420,2	327,9	420,2	-1,3	458,2	209,7	458,2	-1,2	305,2	266,2	285,7	-1,9	141,2	180,7	161,0	-3,4
AT1G70410	VI	620,4	366,9	493,7	1162,6	656,5	1162,6	2,4	248,2	348,3	248,2	-2,0	443,1	270,9	357,0	-1,4	106,1	185,4	145,7	-3,4
AT4G37710	VII	4,6	4,6	4,6	3250,2	1450,9	3250,2	704,6	8331,1	6313,5	8331,1	1806,1	3588,2	7228,6	5408,4	1172,5	3461,2	5330,2	4395,7	952,9
AT1G26380	VII	5,5	15,2	10,4	3169,3	5615,5	3169,3	306,0	7965,9	9191,8	7965,9	769,0	6855,6	7508,5	7182,1	693,4	7802,1	5802,7	6802,4	656,7
AT3G49620	VII	10,9	10,8	10,9	45,4	34,6	45,4	4,2	2164,1	1020,0	2164,1	198,9	3933,3	2977,6	3455,5	317,6	5534,0	5217,8	5375,9	494,1
AT2G39030	VII	11,2	8,5	9,8	37,8	68,3	37,8	3,8	4682,2	2442,0	4682,2	475,8	5249,8	739,5	2994,6	304,3	2832,6	1115,3	1973,9	200,6
AT3G26830	VII	30,2	12,9	21,6	601,6	253,9	601,6	27,9	4659,8	2544,8	4659,8	215,8	4361,9	3416,9	3889,4	180,1	5739,9	4490,8	5115,4	236,9
AT5G40990	VII	4,6	4,6	4,6	48,6	37,7	48,6	10,6	870,8	571,4	870,8	189,7	554,6	556,1	555,3	121,0	908,5	897,8	903,2	196,8
AT3G23250	VII	23,7	37,5	30,6	1146,9	1190,6	1146,9	37,5	1413,3	1477,3	1413,3	46,2	1127,5	1207,7	1167,6	38,2	1166,6	994,4	1080,5	35,3
AT1G52070	VIII	1939,7	450,5	1195,1	1172,3	79,5	1172,3	-1,0	406,4	40,9	406,4	-2,9	58,1	57,3	57,7	-20,7	18,2	29,7	23,9	-49,9
AT1G30870	VIII	1032,9	315,8	674,3	715,7	263,0	715,7	1,1	125,2	28,8	125,2	-5,4	300,6	35,9	168,2	-4,0	8,7	31,3	20,0	-33,8
AT3G62680	VIII	1158,0	382,4	770,2	494,0	198,4	494,0	-1,6	90,2	29,9	90,2	-8,5	272,8	29,1	150,9	-5,1	9,3	36,5	22,9	-33,6
AT5G25460	VIII	1889,9	388,9	1139,4	374,5	34,9	374,5	-3,0	109,1	19,9	109,1	-10,4	157,7	12,4	85,0	-13,4	62,8	15,7	39,2	-29,0
AT5G56540	VIII	2161,4	587,2	1374,3	1017,2	403,4	1017,2	-1,4	309,7	129,8	309,7	-4,4	291,0	119,3	205,2	-6,7	21,6	80,8	51,2	-26,8
AT4G24780	VIII	809,4	198,9	504,1	274,1	130,5	274,1	-1,8	28,0	29,9	28,0	-18,0	37,9	15,4	26,7	-18,9	44,7	59,7	52,2	-9,7
AT4G31730	VIII	1239,5	893,7	1066,6	949,9	1829,7	949,9	-1,1	264,9	269,7	264,9	-4,0	199,4	175,5	187,4	-5,7	359,5	362,1	360,8	-3,0

Q-RT-PCR ANALYSIS

gene ID	NI rep. 3	NI rep. 4	NI rep. mean	2.5 hai rep. 3	2.5 hai rep. 4	2.5 hai rep. mean	2.5 hai Relative fold change 2,5hai/NI	6 hai rep. 3	6 hai rep. 4	6 hai rep. mean	6 hai Relative fold change 6hai/NI	10.5 hai rep. 3	10.5 hai rep. 4	10.5 hai rep. mean	10.5 hai Relative fold change 10.5hai/NI	30 hai rep. 3	30 hai rep. 4	30 hai rep. mean	30 hai Relative fold change 30hai/NI	ARRAY VALIDATION
AT2G44370	0,3	0,5	0,4	7,1	16,4	11,8	31,6	7,9	6,1	7,0	18,7	2,9	2,1	2,5	6,7	1,1	0,5	0,8	2,1	yes
AT5G64120	3,4	3,2	3,3	46,5	61,6	54,1	16,3	110,3	121,4	115,8	35,0	42,5	29,8	36,1	10,9	15,0	7,5	11,3	3,4	yes
AT5G40590	0,6	1,0	0,8	9,2	11,8	10,5	12,6	7,8	6,8	7,3	8,8	4,8	2,5	3,7	4,4	3,9	0,7	2,3	2,8	yes
AT3G02240	0,0	0,0	0,0	0,2	0,6	0,4	19,0	0,4	0,5	0,5	23,0	0,2	0,2	0,2	7,1	0,0	0,0	0,0	2,1	yes
AT5G11920	0,4	1,0	0,7	3,7	4,9	4,3	6,6	3,4	2,4	2,9	4,4	3,9	2,7	3,3	5,0	4,5	2,8	3,7	5,6	yes
AT1G29020	0,7	0,1	0,4	1,1	1,7	1,4	3,4	0,0	0,0	0,0	-13,0	0,1	0,1	0,1	-4,1	0,2	0,1	0,2	-2,6	yes
AT1G11540	0,5	1,6	1,1	1,3	3,5	2,4	2,2	1,2	0,3	0,8	-1,4	1,1	0,9	1,0	-1,1	1,3	0,4	0,9	-1,3	yes
AT3G07350	0,5	0,5	0,5	0,2	0,3	0,2	-1,9	0,5	0,5	0,5	1,0	0,7	0,8	0,7	1,5	0,5	0,9	0,7	1,5	yes
AT3G20340	0,2	0,1	0,1	0,3	0,4	0,4	2,7	1,1	1,3	1,2	8,8	2,2	2,3	2,3	16,5	2,4	1,4	1,9	14,1	no
AT5G67480	0,1	0,0	0,1	0,1	0,1	0,1	-1,0	0,3	0,3	0,3	4,1	0,2	0,2	0,2	2,9	0,3	0,2	0,2	2,9	no
AT4G38470	0,9	0,2	0,6	1,0	1,0	1,0	1,7	2,6	5,9	4,2	7,3	9,9	13,0	11,5	19,6	4,8	2,6	3,7	6,3	no
AT4G03960	2,1	2,8	2,5	0,6	1,2	0,9	-2,6	1,7	1,6	1,6	-1,5	3,7	3,8	3,7	1,5	4,8	9,1	6,9	2,8	yes
AT1G03850	0,0	0,0	0,0	0,0	0,0	0,0	1,2	0,1	0,2	0,1	3,5	0,1	0,1	0,1	3,7	0,3	0,4	0,3	10,8	no
AT5G18680	5,0	3,3	4,2	4,1	4,4	4,3	1,0	6,1	6,3	6,2	1,5	5,0	5,3	5,1	1,2	12,3	6,4	9,3	2,2	no
AT2G23270	0,0	0,1	0,1	5,3	15,3	10,3	197,9	26,0	49,3	37,6	724,3	73,2	29,7	51,4	989,7	13,0	11,1	12,1	232,1	yes
AT5G60760	0,4	1,1	0,7	1,7	11,4	6,5	9,1	8,4	25,3	16,9	23,6	18,3	18,9	18,6	26,0	25,3	12,7	19,0	26,6	yes
AT1G30135	0,0	0,0	0,0	0,1	0,1	0,1	12,5	0,2	0,9	0,5	58,6	3,6	5,9	4,8	522,8	2,5	2,2	2,3	252,8	yes
AT3G02800	0,0	0,1	0,1	0,4	1,4	0,9	17,7	1,8	9,5	5,7	108,2	11,0	9,7	10,3	197,6	5,4	8,6	7,0	134,0	yes
AT2G37430	0,0	0,0	0,0	0,2	0,5	0,4	87,6	0,4	1,2	0,8	188,1	0,5	0,4	0,5	107,8	0,3	0,3	0,3	59,4	yes
AT1G05880	0,0	0,0	0,0	0,9	2,1	1,5	130,8	2,5	1,9	2,2	194,4	2,7	1,8	2,2	193,0	1,6	0,8	1,2	101,3	yes
AT4G22640	0,0	0,0	0,0	0,3	0,8	0,6	16,8	1,2	3,0	2,1	64,2	1,9	2,4	2,1	65,0	1,0	0,5	0,8	24,1	yes
AT3G22840	1,2	2,4	1,8	1,0	1,6	1,3	-1,4	1,9	1,1	1,5	-1,2	0,1	0,1	0,1	-17,5	1,2	1,9	1,5	-1,2	yes
AT2G05070	0,5	3,5	2,0	1,1	1,2	1,2	-1,7	0,2	0,0	0,1	-18,5	0,0	0,0	0,0	-384,0	0,3	0,1	0,2	-9,1	yes
AT3G47500	1,5	4,3	2,9	1,0	1,6	1,3	-2,2	1,0	0,5	0,8	-3,7	0,0	0,0	0,0	-72,6	0,4	0,8	0,6	-4,8	yes
AT5G64170	1,7	1,3	1,5	1,7	1,7	1,7	1,2	2,1	3,7	2,9	2,0	0,6	0,9	0,7	-2,0	1,8	5,3	3,5	2,4	yes
AT5G07030	17,8	17,8	17,8	7,2	17,9	12,6	-1,4	12,0	5,8	8,9	-2,0	3,7	4,0	3,8	-4,6	2,7	4,9	3,8	-4,7	yes
AT1G73220	3,4	2,6	3,0	0,2	0,6	0,4	-7,3	0,6	0,1	0,4	-8,1	0,2	0,1	0,2	-16,1	0,2	0,3	0,2	-12,8	yes
AT5G52310	1,4	4,4	2,9	0,9	1,8	1,3	-2,2	2,8	0,5	1,6	-1,8	0,2	0,2	0,2	-12,4	0,7	1,4	1,0	-2,8	yes
AT5G44400	0,2	0,2	0,2	0,1	0,3	0,2	-1,0	0,2	0,5	0,4	1,9	0,6	1,1	0,8	4,4	2,9	2,9	2,9	15,3	yes
AT5G53970	0,4	0,6	0,5	1,0	0,9	0,9	1,9	1,7	3,4	2,5	5,1	2,5	2,5	2,5	5,0	7,9	14,4	11,1	22,3	yes
AT1G02220	0,8	1,7	1,2	2,2	2,0	2,1	1,7	8,5	25,9	17,2	13,8	10,7	9,5	10,1	8,1	30,2	44,2	37,2	29,9	yes
AT4G21620	1,9	5,8	3,8	3,2	3,1	3,2	-1,2	3,0	5,2	4,1	1,1	6,8	4,5	5,6	1,5	20,5	43,6	32,0	8,4	yes
AT5G02020	0,2	0,2	0,2	0,5	0,6	0,6	2,4	1,3	2,1	1,7	7,3	4,3	2,5	3,4	14,9	2,9	4,8	3,8	16,8	no
AT2G43510	0,0	0,1	0,1	0,2	0,2	0,2	3,4	0,6	2,0	1,3	24,6	3,4	3,3	3,3	63,2	4,4	6,8	5,6	106,9	yes
AT4G16660	3,6	6,3	4,9	6,2	6,2	6,2	1,3	6,2	13,8	10,0	2,0	5,1	7,6	6,3	1,3	14,6	13,7	14,2	2,9	yes
AT1G80240	6,2	21,0	13,6	11,7	26,0	18,8	1,4	29,0	51,1	40,1	3,0	23,4	21,1	22,2	1,6	4,2	18,0	11,1	-1,2	no
AT3G26650	0,2	0,4	0,3	0,5	0,3	0,4	1,3	0,7	0,9	0,8	2,6	1,1	0,8	0,9	3,0	1,6	1,5	1,6	5,2	no
AT5G65040	1,1	1,6	1,3	1,1	2,0	1,6	1,2	2,0	1,6	1,8	1,4	4,0	3,0	3,5	2,6	1,3	0,9	1,1	-1,2	no
AT1G49600	1,3	0,7	1,0	1,3	0,8	1,1	1,0	1,2	1,0	1,1	1,1	1,4	1,2	1,3	1,3	0,6	0,4	0,5	-1,9	yes
AT4G17870	0,1	0,1	0,1	0,1	0,1	0,1	1,6	0,0	0,0	0,0	-1,4	0,1	0,1	0,1	-1,1	0,0	0,0	0,0	-3,4	yes
AT2G34590	1,5	1,1	1,3	1,7	1,3	1,5	1,2	2,2	2,5	2,3	1,9	2,0	1,5	1,7	1,4	0,9	0,8	0,9	-1,5	yes
AT1G70410	0,6	0,3	0,5	0,9	1,5	1,2	2,7	1,8	1,3	1,5	3,4	0,6	0,5	0,6	1,3	0,2	0,2	0,2	-2,4	yes
AT4G37710	0,0	0,0	0,0	2,1	6,0	4,1	195,2	4,4	38,0	21,2	1012,0	57,6	41,6	49,6	2364,2	75,1	68,8	72,0	3431,4	yes
AT1G26380	0,0	0,0	0,0	13,8	30,8	22,3	892,2	51,7	98,4	75,0	2998,0	66,8	69,2	68,0	2717,1	109,9	59,6	84,8	3386,6	yes
AT3G49620	0,0	0,0	0,0	0,2	0,2	0,2	11,3	1,0	25,2	13,1	801,5	18,5	18,8	18,7	1141,7	61,2	74,5	67,9	4154,4	yes
AT2G39030	0,0	0,0	0,0	0,0	0,0	0,0	1,2	0,1	1,0	0,6	53,1	6,0	9,0	7,5	722,6	9,1	3,2	6,2	591,2	yes
AT3G26830	0,0	0,1	0,1	0,1	0,3	0,2	3,1	4,4	14,2	9,3	177,6	15,0	16,6	15,8	300,9	23,1	24,7	23,9	455,3	yes
AT5G40990	0,0	0,0	0,0	0,0	0,1	0,0	7,3	1,3	9,0	5,2	777,1	6,4	7,4	6,9	1038,9	5,2	3,5	4,3	653,0	yes
AT3G23250	0,0	0,0	0,0	0,2	0,0	0,1	9,1	0,4	2,1	1,3	136,7	2,3	2,3	2,3	249,1	1,1	1,6	1,4	145,7	yes
AT1G52070	2,2	3,0	2,6	1,7	2,3	2,0	-1,3	2,0	1,3	1,6	-1,6	0,7	0,2	0,5	-5,8	0,1	0,1	0,1	-40,6	yes
AT1G30870	3,6	8,0	5,8	8,5	13,2	10,9	1,9	8,1	6,3	7,2	1,2	2,3	1,4	1,8	-3,1	0,5	0,2	0,4	-15,3	no
AT3G62680	1,2	1,6	1,4	2,3	4,7	3,5	2,5	2,6	0,7	1,6	1,2	0,4	0,3	0,3	-4,3	0,0	0,0	0,0	-39,9	no
AT5G25460	0,1	0,0	0,1	0,1	0,4	0,3	2,9	0,6	0,0	0,3	3,7	0,1	0,5	0,3	3,6	0,0	0,0	0,0	-3,3	no
AT5G56540	0,7	2,0	1,3	1,2	1,3	1,2	-1,1	1,1	0,7	0,9	-1,5	0,3	0,1	0,2	-6,6	0,0	0,1	0,0	-27,4	yes
AT4G24780	2,2	0,9	1,5	0,6	1,0	0,8	-2,0	0,5	0,4	0,5	-3,4	0,5	0,6	0,5	-3,0	0,2	0,1	0,2	-9,8	yes
AT4G31730	0,7	2,2	1,5	0,7	1,2	0,9	-1,6	0,6	0,2	0,4	-3,7	0,3	0,2	0,3	-5,3	0,4	0,5	0,4	-3,6	yes
