# Supplementary material for: Transcriptome dynamic of Arabidopsis roots infected with Phytophthora parasitica identifies VQ29, a gene induced during the penetration and involved in the restriction of infection
Source: PLoS One. 2017 Dec 27;12(12):e0190341. doi: 10.1371/journal.pone.0190341 (PMC5744986; doi:10.1371/journal.pone.0190341)
Supplement: S5 Table — Each term, is indicated in parenthesis the corresponding code number followed by the number of genes from Arabidopsis genome. Significant overrepresented of terms is indicated in bold. (PDF) [file pone.0190341.s009.pdf]

S5 Table. Representation of the pricipal terms within the MIPS Functional catalogue database of Arabidopsis thaliana genes differentially expressed in roots infected with the oomycete Phytophthora parasitica (Klatari et al., 2010, Virtual plant 1.3)
Each term, is indicated in parenthesis the corresponding code number followed by the number of genes from Arabidopsis genome.
Significant overrepresented of terms is indicated in bold.

	**Up-regulated clusters**								**Down-regulated clusters**							
**MIPS Functional Category database terminology (FunCatDB)**	**VII-Interaction (n=1299)**		**I-penetration (n=205)**		**III-Biotrophy (n=109)**		**V-Switch (n=125)**		**VIII-Interaction (n=1248)**		**II-penetration (n=57)**		**IV-Biotrophy (n=128)**		**VI-Switch (n=219)**	
	p-value	nb (%)	p-value	nb (%)	p-value	nb (%)	p-value	nb (%)	p-value	nb (%)	p-value	nb (%)	p-value	nb (%)	p-value	nb (%)
**METABOLISM (FunCat 01, n=4823)**	**1,20E-35**	446 (33.3%)	**1,40E-04**	63 (31.8%	**6,00E-01**	27 (22.9%)	**2,10E-01**	31 (24%)	**3,40E-13**	353 (27.4%)	1,10E-01	17 (28.8%)	**3,00E-02**	36 (27.9%)	9,60E-01	37 (16%
**ENERGY (FunCat 02, n=451)**	9,40E-01	446 (33.3%)	**0.07**	9 (4.5%)	nd	nd	**1,50E-01**	6 (4.7%)	**5,00E-02**	35 (2.7%)	7,20E-01	1 (1.7%)	**2,20E-04**	11 (8.5%)	**5,00E-02**	11 (4.8%)
**STORAGE PROTEIN (FunCat 04, n=65)**	**2,10E-03**	12 (0.9%)	nd	nd	4,00E-01	2 (1.7%)	**1,80E-01**	2 (1.6%)	1,00E+00	1 (0.1%)	2,50E-01	1 (1.7%)	nd	nd	nd	nd
**CELL CYCLE AND DNA PROCESSING (FunCat 10, n=1450)**	1,00E+00	63 (4.7%)	0.51	13 (6.6%)	9,10E-01	5 (4.2%)	3,60E-01	10 (7.8%)	9,90E-01	59 (4.6%)	4,80E-01	4 (6.8%)	8,30E-01	6 (4.7%)	1,00E+00	8 (3.5%)
**TRANSCRIPTION (FunCat 11, n=2621),**	1,00E+00	96 (7.2%)	0.53	22 (11.1%)	9,10E-01	10 (8.5%)	7,40E-01	12 (9.3%)	1,00E+00	95 (7.4%)	1,10E-01	11 (18.6%)	7,40E-01	13 (10.1%)	1,00E+00	16 (6.9%)
**PROTEIN SYNTHESIS (FunCat 12, n=1326)**	1,00E+00	13 (1%)	0.95	6 (3%)	nd	nd	9,90E-01	2 (1.6%)	1,00E+00	23 (1.8%)	nd	nd	1,00E+00	1 (0.8%)	**7,70E-04**	28 (12.1%)
**PROTEIN FATE (FunCat 14, n=3013)**	**2,00E-07**	223 (16.6%)	0.00509	40 (20.2%)	7,00E-01	17 (14.4%)	5,50E-01	16 (12.4%)	2,90E-01	23 (1.8%)	5,90E-01	7 (11.9%)	6,80E-01	16 (12.4%)	9,70E-01	67 (29%)
**PROTEIN WITH BINDING FUNCTION (FunCat 16, n=6922)**	**2,00E-02**	393 (29.3%)	0.40	58 (29.3%)	9,30E-01	25 (21.2%)	3,30E-01	40 (31%)	6,70E-01	337 (26.2%)	9,10E-01	12 (20.3%)	9,60E-01	26 (20.2%)	5,00E-01	67 (29%)
**REGULATION OF METABOLISM (FunCat 18, n=n=603)**	7,00E-02	43 (3.2%)	0.79	4 (2%)	9,10E-01	2 (1.7%)	5,10E-01	4 (3.1%)	3,30E-01	37 (2.9%)	1,30E-01	4 (6.8%)	9,60E-01	1 (0.8%)	9,30E-01	4 (1.7%)
**CELLULAR TRANSPORT (FunCat 20, n=2404)**	**5,40E-05**	172 (12.8%)	0.36	23 (11.6%)	9,10E-01	9 (7.6%)	3,80E-01	15 (11.6%)	**6,80E-07**	180 (14%)	1,80E-01	9 (15.3%)	**2,40E-01**	17 (13.2%)	5,30E-01	25 (10.8%)
**CELLULAR COMMUNICATION / SIGNAL TRANSDUCTION MECHANISM (FunCat 30, n=1235)**	**1,10E-08**	117 (8.7%)	0.15	16 (8.1%)	6,00E-01	9 (7.6%)	8,00E-01	5 (3.9%)	5,60E-01	64 (5%)	1,50E-01	6 (10.2%)	**5,50E-01**	17 (13.2%)	1,00E+00	4 (1.7%)
**CELL RESCUE, DEFENSE AND VIRULENCE (FunCat 32, n=1349)**	**9,00E-26**	176 (13.1%)	**2.08e-05**	30 (15.2%)	4,00E-01	12 (10.2%)	**8,50E-03**	18 (14%)	**1,10E-04**	105 (8.2%)	1,10E-01	7 (11.9%)	**3,20E-07**	25 (19.4%)	7,60E-01	12 (5.2%)
**INTERACTION WITH THE ENVIRONMENT (FunCat 34, n=1451)**	**6,10E-11**	140 (10.4%)	**0.01**	23 (11.6%)	9,10E-01	5 (4.2%)	**5,70E-01**	12 (9.3%)	**6,30E-08**	127 (9.9%)	**8,00E-02**	8 (13.6%)	**8,20E-08**	27 (20.9%)	3,90E-01	18 (7.8%)
**SYSTEMIC INTERACTION WITH THE ENVIRONMENT (FunCat 36, n=664)**	**3,50E-11**	83 (6.2%)	0.57	6 (3%)	8,60E-01	3 (2.5%)	5,70E-01	4 (3.1%)	**3,00E-02**	49 (3.8%)	7,00E-02	6 (10.2%)	**4,00E-02**	9 (7%)	5,50E-01	8 (3.5%)
**CELL FATE (FunCat 40, n=378)**	2,30E-01	26 (1.9%)	0.39	1 (0.5%)	9,10E-01	1 (0.8%)	8,50E-01	12 (9.3%)	**4,90E-03**	35 (2.7%)	3,00E-01	2 (3.4%)	9,00E-01	1 (0.8%)	3,10E-01	7 (3%)
