# Supplementary material for: Transcriptome dynamic of Arabidopsis roots infected with Phytophthora parasitica identifies VQ29, a gene induced during the penetration and involved in the restriction of infection
Source: PLoS One. 2017 Dec 27;12(12):e0190341. doi: 10.1371/journal.pone.0190341 (PMC5744986; doi:10.1371/journal.pone.0190341)
Supplement: S6 Table — Clusters were identified from microarray data GEO:GPL198. Clusters I, III and VII group genes transiently upregulated throughout infection, whereas clusters IV and VIII group genes downregulated. For each MIPS FunCatDB terminology, corresponding gene list and p-value are indicated. Highlighted classes are described in the manuscript. (PDF) [file pone.0190341.s010.pdf]

S6 Table. Terms within the MIPS Functional Catalogue Database (FunCatDB) overrepresented (p-value<0,5) in clusters I, III, IV, VII and VIII.

Clusters were identified from microarray data GEO:GPL198. Clusters I, III and VII group genes transiently upregulated throughout infection, whereas clusters IV and VIII group genes downregulated. For each MIPS FunCatDB terminology, corresponding gene list and p-value are indicated. Highlighted classes are described in the ma

Cluster	FunCatDB	Term	Number of gene observed	Total gene in Cluster	p-value	% of gene per cluster	*A. thaliana* expected frequency	Genes list
I	1	METABOLISM	63	198	1,4E-04	31.8%	4823 out of 26623 genes, 18.1%	AT3G13080|AT2G47730|AT2G19190|AT2G39110|AT4G25310|AT2G30020|AT2G43820|AT5G38530|AT3G54030|AT2G03760|AT5G26030|AT2G2 5G38990|AT3G11170|AT1G65610|AT2G32020|AT1G51800|AT3G30180|AT2G36690|ATCG01070|AT2G28990|AT2G47060|ATMG00410|AT5G39 G71330|AT2G28890|AT2G29740|AT4G26200|AT5G05580|AT1G23480|AT2G14440|AT1G47840|AT1G53920|AT1G02360|AT4G24040|AT2G3942 5300|AT1G16150|AT5G11920|AT4G08260|ATCG00130|AT5G03320|AT5G37450|AT1G05680|AT2G26290|AT1G05700|AT1G07560|AT5G01550|
I	14	PROTEIN FATE (folding, modification, destination)	40	198	5,1E-03	20.2%	3013 out of 26623 genes, 11.3%	AT2G14440|AT2G47730|AT4G25110|AT4G23570|AT2G39110|AT3G12580|AT1G02440|AT2G30020|AT3G54030|AT4G08850|AT2G28970|AT1G5 4G08260|AT2G20560|AT3G45330|AT5G38990|AT5G03320|AT5G37450|AT3G51330|AT5G64120|AT1G51800|AT1G05700|AT1G07560|AT5G015 39000|AT4G02410|AT4G26070|AT4G05200|AT2G41890|AT2G28890|AT3G27140|AT3G12700
I	32	CELL RESCUE, DEFENSE AND VIRULENCE	30	198	2,1E-05	15.2%	1349 out of 26623 genes, 5.1%	AT3G13080|AT5G45080|AT2G47730|AT4G23570|AT2G19190|AT5G64120|AT3G12580|AT5G45340|AT4G16940|AT5G45070|AT2G03760|AT1G5 4G31550|AT2G23960|AT4G16960|AT4G19510|AT4G33300|AT5G64110|AT2G26150|AT1G17180|AT5G64100|AT3G11170|AT2G41010|ATCG004
I	34	INTERACTION WITH THE ENVIRONMENT	23	198	1,0E-02	11.6%	1451 out of 26623 genes, 5.5%	AT2G19190|AT2G32020|AT5G64120|AT3G12580|AT5G45340|AT1G16510|AT3G04110|AT5G49480|AT4G26070|AT2G41110|AT1G17060|AT2G3 2G26150|AT1G19050|AT3G11170|AT2G41010|ATCG00420|AT5G05580|AT2G46830
I	01.04	phosphate metabolism	32	198	3,3E-04	16.2%	1845 out of 26623 genes, 6.9%	AT5G37450|AT3G13080|AT2G14440|AT2G19190|AT2G39110|AT1G47840|AT2G26290|AT2G30020|AT1G51800|AT1G05700|AT3G54030|AT1G0 2G47060|AT5G39000|AT4G02410|AT4G26070|AT2G24130|AT4G05200|AT2G41890|AT2G14510|AT1G16150|AT1G71330|AT2G28890|AT3G271
I	01.20	secondary metabolism	11	198	6,2E-03	5.6%	410 out of 26623 genes, 1.5%	AT3G13610|AT1G17060|AT1G17180|AT2G19590|AT2G47730|AT4G26200|ATCG01070|AT5G26030|AT2G33590|AT4G25310|AT4G25300
I	02.13	respiration	5	198	2,0E-02	2.5%	99 out of 26623 genes, 0.4%	ATCG00420|ATMG00220|ATMG00410|ATCG01010|ATCG00130
I	02.30	photosynthesis	4	198	2,0E-02	2%	63 out of 26623 genes, 0.2%	ATCG01080|ATCG01010|ATCG01060|ATCG00690
I	02.45.15	energy generation (e.g. ATP synthase)	3	198	5,0E-02	1.5%	42 out of 26623 genes, 0.2%	AT2G07741|ATMG00410|ATCG00130
I	14.07	protein modification	35	198	3,5E-04	17.7%	2142 out of 26623 genes, 8%	AT2G14440|AT2G47730|AT4G23570|AT2G39110|AT1G02440|AT2G30020|AT3G54030|AT4G08850|AT2G28970|AT1G59730|AT3G01290|AT2G2 5G38990|AT5G03320|AT5G37450|AT5G64120|AT1G51800|AT1G05700|AT1G07560|AT5G01550|AT1G02430|AT2G28990|AT2G47060|AT2G359 41890|AT2G28890|AT3G27140
I	14.07.03	modification by phosphorylation, dephosphorylation, auto	27	198	4,9E-05	13.6%	1235 out of 26623 genes, 4.6%	AT5G37450|AT2G14440|AT2G39110|AT2G30020|AT1G51800|AT1G05700|AT3G54030|AT1G07560|AT5G01550|AT4G08850|AT2G28970|AT2G4 2G24130|AT4G05200|AT2G41890|AT2G14510|AT1G16150|AT2G28890|AT3G27140|AT4G08260|AT3G45330|AT5G38990|AT5G03320
I	16.17.01	calcium binding	6	198	3,0E-02	3%	172 out of 26623 genes, 0.6%	AT2G46600|AT2G41110|AT2G41410|AT3G01830|AT5G49480|AT1G29020
I	20.01.15	electron transport	14	198	2,0E-02	7.1%	717 out of 26623 genes, 2.7%	ATMG00410|AT1G27770|AT2G07741|ATCG01050|AT2G07727|ATCG01080|ATCG00130|ATCG01010|ATCG00150|AT1G13110|ATCG01070|ATCG
I	30.05	transmembrane signal transduction	10	198	5,1E-03	5.1%	324 out of 26623 genes, 1.2%	AT5G37450|AT4G16960|AT4G19510|AT4G16940|AT5G45070|AT2G24130|AT5G45080|AT2G28970|AT4G09420|AT4G16250
I	30.05.01	receptor enzyme mediated signalling	9	198	6,2E-03	4.5%	281 out of 26623 genes, 1.1%	AT5G37450|AT4G16960|AT4G19510|AT4G16940|AT5G45070|AT2G24130|AT5G45080|AT2G28970|AT4G09420
I	32.05	disease, virulence and defense	16	198	2,1E-05	8.1%	424 out of 26623 genes, 1.6%	AT4G16960|AT4G19510|AT4G33300|AT5G64120|AT4G26070|AT4G16940|AT5G45070|AT5G45080|AT2G39200|AT2G47730|AT4G23570|AT2G1
I	32.07	detoxification	7	198	5,0E-02	3.5%	261 out of 26623 genes, 1%	AT3G13080|AT5G64110|AT5G64120|AT1G17180|AT2G47730|AT5G64100|AT1G59730
I	32.07.07	oxygen and radical detoxification	7	198	4,0E-02	3.5%	256 out of 26623 genes, 1%	AT3G13080|AT5G64110|AT5G64120|AT1G17180|AT2G47730|AT5G64100|AT1G59730
I	34.11	cellular sensing and response to external stimulus	23	198	3,6E-03	11.6%	1296 out of 26623 genes, 4.9%	AT2G19190|AT2G32020|AT5G64120|AT3G12580|AT5G45340|AT1G16510|AT3G04110|AT5G49480|AT4G26070|AT2G41110|AT1G17060|AT2G3 2G26150|AT1G19050|AT3G11170|AT2G41010|ATCG00420|AT5G05580|AT2G46830
IV	1	METABOLISM	36	129	3,0E-02	27.9%	4823 out of 26623 genes, 18.1%	AT1G74470|AT3G02040|AT3G17790|AT1G75210|AT5G13630|AT5G11110|AT3G51240|AT1G06000|AT5G03650|AT1G32200|AT4G17770|AT1G1 4G10120|AT1G72180|AT2G47180|AT2G25140|AT3G43600|AT1G08650|AT3G09910|AT2G35840|AT1G78060|AT2G22240|AT3G11670|AT5G576 08380|AT1G17050|AT1G72810|AT5G08640
IV	2	ENERGY	11	129	2,2E-04	8.5%	451 out of 26623 genes, 1.7%	AT2G35840|AT4G17770|AT1G15820|AT2G34430|AT1G08650|AT5G03650|AT1G61520|AT2G05100|AT2G34420|AT2G05070|AT2G21970
IV	32	CELL RESCUE, DEFENSE AND VIRULENCE	25	129	3,2E-07	19.4%	1349 out of 26623 genes, 5.1%	AT3G22840|AT5G52310|AT2G22240|AT2G19310|AT2G26690|AT4G24190|AT1G56340|AT1G09210|AT5G67030|AT1G76080|AT5G15960|AT1G2 4G34000|AT1G12370|AT1G75280|AT2G46680|AT2G15970|AT1G29390|AT2G27190|AT5G15970|AT1G06040
IV	34	INTERACTION WITH THE ENVIRONMENT	27	129	8,2E-08	20.9%	1451 out of 26623 genes, 5.5%	AT3G22840|AT5G52310|AT2G22240|AT3G51240|AT2G40460|AT2G19310|AT2G26690|AT4G24190|AT5G67030|AT3G02050|AT5G15960|AT2G4 2G25140|AT4G00880|AT2G46270|AT5G64840|AT4G34000|AT1G12370|AT2G46680|AT2G15970|AT1G13080|AT5G15970|AT1G06040
IV	36	SYSTEMIC INTERACTION WITH THE ENVIRONMENT	9	129	4,0E-02	7%	664 out of 26623 genes, 2.5%	AT5G52310|AT4G00880|AT2G46270|AT2G26690|AT4G34000|AT2G46680|AT2G15970|AT5G15960|AT5G15970
IV	01.05	C-compound and carbohydrate metabolism	17	129	2,0E-02	13.2%	1625 out of 26623 genes, 6.1%	AT2G35840|AT1G78060|AT2G22240|AT1G06000|AT5G03650|AT3G11670|AT4G17770|AT4G10120|AT2G47180|AT3G02040|AT3G17790|AT5G5 5G11110
IV	01.05.02	sugar, glucoside, polyol and carboxylate metabolism	12	129	9,7E-03	9.3%	846 out of 26623 genes, 3.2%	AT2G35840|AT1G78060|AT1G60470|AT5G08380|AT2G22240|AT1G06000|AT3G11670|AT3G21560|AT4G17770|AT4G10120|AT2G47180|AT5G1
IV	01.05.02.04	sugar, glucoside, polyol and carboxylate anabolism	5	129	2,2E-04	3.9%	56 out of 26623 genes, 0.2%	AT2G35840|AT4G10120|AT5G11110|AT2G22240|AT4G17770
IV	01.06.02	membrane lipid metabolism	4	129	4,0E-02	3.1%	148 out of 26623 genes, 0.6%	AT3G11670|AT3G02040|AT2G22240|AT1G32200
IV	01.06.06.05	sesquiterpenes metabolism	2	129	1,0E-02	1.6%	11 out of 26623 genes, 0%	AT5G67030|AT3G43600
IV	01.20.35.01.0	metabolism of stilbenes, flavonoids	3	129	1,0E-02	2.3%	42 out of 26623 genes, 0.2%	AT5G08640|AT3G51240|AT3G55120
IV	02.30	photosynthesis	7	129	1,5E-06	5.4%	63 out of 26623 genes, 0.2%	AT2G34430|AT2G05100|AT2G34420|AT2G21970|AT1G61520|AT2G05070|AT1G15820
IV	02.45	energy conversion and regeneration	5	129	4,0E-04	3.9%	69 out of 26623 genes, 0.3%	AT2G34430|AT2G05100|AT2G34420|AT2G05070|AT1G61520
IV	02.45.03	light absorption	5	129	3,2E-06	3.9%	20 out of 26623 genes, 0.1%	AT2G34430|AT1G61520|AT2G05070|AT2G05100|AT2G34420
IV	20.01.09	peptide transport	3	129	3,0E-02	2.3%	66 out of 26623 genes, 0.2%	AT2G40460|AT3G21670|AT1G32450
IV	32.01	stress response	24	129	2,2E-10	18.6%	795 out of 26623 genes, 3%	AT3G22840|AT5G52310|AT3G17790|AT2G25140|AT2G22240|AT2G19310|AT2G26690|AT4G34000|AT4G24190|AT1G12370|AT1G56340|AT1G7 5G15960|AT1G29390|AT2G27190|AT5G15970|AT1G22770|AT2G47180|AT1G06040|AT3G55120
IV	32.01.01	oxidative stress response	8	129	1,4E-04	6.2%	196 out of 26623 genes, 0.7%	AT1G75280|AT2G19310|AT1G22770|AT3G17790|AT2G47180|AT1G09210|AT2G22240|AT1G56340
IV	32.01.03	osmotic and salt stress response	6	129	5,8E-03	4.7%	201 out of 26623 genes, 0.8%	AT5G15970|AT5G52310|AT1G06040|AT4G34000|AT5G67030|AT5G15960
IV	32.01.05	heat shock response	5	129	9,5E-04	3.9%	85 out of 26623 genes, 0.3%	AT5G67030|AT2G19310|AT2G22240|AT2G25140|AT2G47180
IV	32.01.06	cold shock response	7	129	2,2E-04	5.4%	154 out of 26623 genes, 0.6%	AT3G22840|AT5G15970|AT1G22770|AT5G52310|AT2G15970|AT5G15960|AT4G24190
IV	32.01.13	electromagnetic waves stress response (e.g. UV, X-ray)	2	129	5,0E-02	1.6%	26 out of 26623 genes, 0.1%	AT1G12370|AT3G55120
IV	34.11	cellular sensing and response to external stimulus	26	129	4,9E-08	20.2%	1296 out of 26623 genes, 4.9%	AT3G22840|AT5G52310|AT2G22240|AT3G51240|AT2G40460|AT2G19310|AT2G26690|AT4G24190|AT5G67030|AT3G02050|AT5G15960|AT2G4 4G00880|AT2G46270|AT5G64840|AT4G34000|AT1G12370|AT2G46680|AT2G15970|AT1G13080|AT5G15970|AT1G06040
IV	34.11.01	photoperception and response	6	129	1,0E-02	4.7%	243 out of 26623 genes, 0.9%	AT2G19310|AT2G47180|AT3G55120|AT2G22240|AT3G51240|AT1G12370
IV	34.11.03	chemoperception and response	11	129	1,0E-02	8.5%	770 out of 26623 genes, 2.9%	AT5G52310|AT4G00880|AT2G46270|AT2G26690|AT4G34000|AT2G46680|AT2G15970|AT5G67030|AT5G15960|AT5G15970|AT1G06040
IV	34.11.03.12	water response	7	129	5,4E-05	5.4%	116 out of 26623 genes, 0.4%	AT4G34000|AT5G15970|AT5G67030|AT2G46680|AT5G52310|AT5G15960|AT2G15970
IV	34.11.03.13	osmosensing and response	6	129	5,8E-03	4.7%	202 out of 26623 genes, 0.8%	AT5G15970|AT5G52310|AT1G06040|AT4G34000|AT5G67030|AT5G15960
IV	34.11.09	temperature perception and response	14	129	3,5E-09	10.9%	248 out of 26623 genes, 0.9%	AT2G46790|AT3G22840|AT5G15970|AT2G19310|AT1G22770|AT5G52310|AT2G15970|AT2G25140|AT2G47180|AT1G13080|AT5G67030|AT5G1
IV	36.20	plant / fungal specific systemic sensing and response	9	129	2,0E-02	7%	599 out of 26623 genes, 2.2%	AT5G52310|AT4G00880|AT2G46270|AT2G26690|AT4G34000|AT2G46680|AT2G15970|AT5G15960|AT5G15970
IV	36.20.18	plant hormonal regulation	9	129	1,0E-02	7%	539 out of 26623 genes, 2%	AT5G52310|AT4G00880|AT2G46270|AT2G26690|AT4G34000|AT2G46680|AT2G15970|AT5G15960|AT5G15970
IV	36.20.18.05	abscisic acid response	7	129	2,2E-04	5.4%	157 out of 26623 genes, 0.6%	AT5G15970|AT2G46680|AT5G52310|AT2G15970|AT4G34000|AT5G15960|AT2G46270
VII	1	METABOLISM	446	1340	1,2E-35	33.3%	4823 out of 26623 genes, 18.1%	AT5G20050|AT1G05010|AT4G39980|AT1G24100|AT5G13280|AT4G27830|AT2G46620|AT1G20510|AT4G29810|AT1G02310|AT4G28490|AT5G2 5G04590|AT3G09830|AT1G57700|AT1G03905|AT1G07240|AT1G51760|AT1G72330|AT1G66880|AT4G32300|AT4G31500|AT5G07370|AT2G029 31800|AT4G23190|AT2G33580|AT4G11280|AT5G58940|AT2G30870|AT1G54100|AT1G02930|AT1G18480|AT1G08630|AT1G21130|AT1G77120 400|AT3G05050|AT2G05940|AT2G19800|AT1G37130|AT1G69960|AT2G14750|AT1G73730|AT4G18050|AT1G09940|AT4G21390|AT1G23730|A 0|AT1G79670|AT4G30490|AT1G56145|AT5G53990|AT1G06840|AT1G76470|AT1G48260|AT1G70740|AT5G02100|AT1G22280|AT3G60120|AT3 AT1G55020|AT4G16260|AT1G17170|AT3G08720|AT2G31020|AT2G34650|AT3G57760|AT2G02220|AT1G30620|AT4G30210|AT2G20850|AT3G2 1G67070|AT4G01700|AT3G19030|AT4G39270|AT3G10340|AT1G79680|AT5G22250|AT4G16760|AT1G21590|AT3G12500|AT4G23280|AT1G518 01850|AT5G57890|AT5G12890|AT2G31955|AT2G20900|AT4G15130|AT1G67000|AT5G07440|AT2G48010|AT1G59870|AT2G01180|AT3G47730 660|AT2G36970|AT3G26830|AT1G71697|AT5G14930|AT4G35600|AT3G23150|AT2G36790|AT4G21400|AT1G80820|AT3G16150|AT4G37670|A 0|AT3G17410|AT2G22250|AT2G30140|AT2G36800|AT1G21210|AT1G32170|AT3G62150|AT5G02290|AT4G25810|AT2G34180|AT2G36770|AT5 AT4G26270|AT5G27380|AT5G11520|AT5G20990|AT2G43570|AT5G34780|AT1G56120|AT3G19010|AT5G20830|AT4G23730|AT2G41850|AT5G6 5G01950|AT4G28350|AT3G46930|AT1G66690|AT1G18890|AT1G55850|AT2G29440|AT4G39950|AT5G05600|AT3G55270|AT5G67080|AT5G652 17510|AT2G24850|AT1G18500|AT5G25930|AT5G46080|AT1G27130|AT5G44290|AT1G67560|AT1G64160|AT1G51780|AT2G23770|AT1G01140 150|AT3G22890|AT3G44260|AT1G09480|AT3G15060|AT1G48320|AT4G04540|AT1G21230|AT1G48850|AT3G13100|AT3G02360|AT2G26560|A 0|AT5G05730|AT1G67880|AT1G43910|AT5G44070|AT3G28340|AT4G25390|AT3G48790|AT4G13920|AT1G35670|AT4G02520|AT2G31880|AT1 AT2G16500|AT5G49780|AT4G16820|AT5G17990|AT4G35630|AT1G61550|AT2G29420|AT2G07180|AT5G54810|AT5G22300|AT4G18250|AT2G2 2G37040|AT5G49760|AT4G27070|AT2G28940|AT3G25900|AT4G34230|AT3G25250|AT5G58540|AT1G53430|AT1G56140|AT2G39410|AT1G303 11480|AT1G75020|AT1G74710|AT2G44490|AT4G23270|AT4G27290|AT3G47780|AT4G28400|AT2G22330|AT3G28180|AT5G57500|AT5G16910 750|AT1G69740|AT1G02400|AT1G74310|AT4G11840|AT2G15480|AT2G40270|AT3G16030|AT3G28580|AT1G01480|AT2G41880|AT3G53810|A
VII	4	STORAGE PROTEIN	12	1340	2,1E-03	0.9%	65 out of 26623 genes, 0.2%	AT5G38910|AT5G39180|AT1G18970|AT5G39150|AT5G39110|AT3G05950|AT5G39130|AT5G39120|AT5G39160|AT2G26560|AT5G39100|AT5G3
VII	14	PROTEIN FATE (folding, modification, destination)	223	1340	2,0E-07	16.6%	3013 out of 26623 genes, 11.3%	AT5G20050|AT4G04540|AT1G21230|AT4G29810|AT3G26020|AT4G28490|AT3G02740|AT4G36550|AT5G15730|AT1G67880|AT4G14220|AT4G2 1G14370|AT1G57700|AT1G19530|AT1G07630|AT1G54960|AT1G51760|AT4G03510|AT1G66880|AT4G32300|AT5G49780|AT1G14260|AT2G261 31800|AT2G07180|AT1G32960|AT1G12360|AT4G23190|AT2G33580|AT4G18250|AT5G58940|AT2G17110|AT1G18480|AT5G42000|AT5G49760 210|AT3G05050|AT2G05940|AT1G08050|AT5G58540|AT5G60410|AT1G69960|AT1G53430|AT1G56140|AT1G24140|AT1G61370|AT4G11480|A 0|AT5G67250|AT5G51070|AT1G56145|AT1G06840|AT4G18950|AT1G53440|AT1G77500|AT1G48260|AT4G10520|AT1G17750|AT1G70740|AT1 AT3G16030|AT3G08720|AT2G34650|AT4G33910|AT3G53810|AT1G70170|AT3G57760|AT2G17530|AT5G60280|AT2G02220|AT3G05350|AT1G0 5G38900|AT5G39020|AT5G66210|AT3G47570|AT5G04870|AT1G16540|AT3G53180|AT3G08760|AT3G20410|AT4G33920|AT4G39270|AT1G697 12520|AT3G11840|AT1G21590|AT3G61390|AT5G64660|AT5G47070|AT1G51660|AT1G51890|AT3G12200|AT5G01850|AT1G11050|AT1G67000 050|AT3G59700|AT2G39660|AT4G35230|AT5G22060|AT3G02070|AT4G35600|AT3G23150|AT4G21400|AT1G73500|AT1G76040|AT3G16150|A 0|AT3G17410|AT1G61360|AT1G21210|AT5G02290|AT4G36410|AT2G34180|AT5G57190|AT1G11330|AT4G04570|AT4G34210|AT3G02750|AT5 AT1G25390|AT3G09010|AT3G07370|AT4G10530|AT5G14420|AT1G56120|AT2G30360|AT1G66480|AT3G55950|AT4G36880|AT3G59080|AT3G0 2G46500|AT4G35310|AT1G67800|AT3G14840|AT1G53570|AT5G01950|AT4G28350|AT3G46930|AT1G18890|AT5G03730|AT3G55270|AT4G013 67080|AT5G65240|AT5G59700|AT3G01650|AT3G22260|AT5G12480|AT3G20860|AT5G04540|AT5G38210|AT5G25930|AT5G46080|AT1G56130 670|AT1G01140|AT1G66160|AT5G35580|AT3G45640|AT5G60750|AT5G09800|AT4G22710|AT4G12120|AT4G34470|AT1G74360|AT1G01560|A
VII	16	PROTEIN WITH BINDING FUNCTION OR COFACTOR REQUIR	393	1340	2,0E-02	29.3%	6922 out of 26623 genes, 26%	AT1G27730|AT2G46620|ATMG00980|AT3G05200|AT4G31950|AT3G26020|AT4G28490|AT2G02820|AT2G31230|AT2G18350|AT4G11370|AT5G T4G17490|AT1G03905|AT5G41740|AT5G67310|AT4G36150|AT4G32300|AT3G18290|AT4G31500|AT4G11170|AT5G58720|AT2G13790|AT2G13 G04340|AT5G44870|AT3G47640|AT3G50950|AT2G47520|AT4G09100|AT3G47480|AT5G61210|AT1G37130|AT5G42940|AT2G14750|AT5G4773 4180|AT1G23730|AT3G61190|AT5G51070|AT1G53390|AT4G30490|AT1G43160|AT5G45110|AT2G30770|AT1G18570|AT1G06840|AT3G16530| 60|AT4G36140|AT2G30750|AT2G22690|AT2G41700|AT4G26400|AT2G32250|AT2G15760|AT5G07390|AT1G78820|AT5G27520|AT3G57760|AT |AT4G30210|AT4G30350|AT1G32530|AT3G28450|AT3G17700|AT4G37120|AT1G01260|AT1G02860|AT3G43890|AT2G31810|AT2G02010|AT4G T3G04720|AT1G18970|AT1G15890|AT5G27610|AT5G64660|AT1G72680|AT5G42650|AT1G19310|AT4G37370|AT2G46430|AT1G59870|AT5G54 G13195|AT3G26830|AT4G22690|AT5G15640|AT1G64280|AT1G33600|AT4G39540|AT5G59940|AT5G39130|AT1G06770|AT3G43430|AT1G7408 3250|AT3G62150|AT5G39660|AT1G11330|AT5G58430|AT4G34210|AT4G26270|AT1G70670|AT1G61560|AT5G20990|AT5G47220|AT5G46520| 80|AT1G66400|AT5G01950|AT5G59930|AT4G28350|AT5G27420|AT5G57580|AT5G39150|AT2G31180|AT1G08830|AT5G24760|AT2G37520|AT |AT5G59550|AT5G05600|AT5G67340|AT1G78860|AT1G76650|AT5G46470|AT5G39160|AT3G54640|AT3G17510|AT3G55730|AT1G13100|AT3G T4G24570|AT2G37150|AT1G66160|AT5G42010|AT4G39890|AT4G01010|AT1G11310|AT1G06180|AT5G09800|AT2G44070|AT5G01500|AT4G17 G29400|AT3G15060|AT5G35200|AT3G51920|AT1G72800|AT3G02360|AT3G13100|AT5G49620|AT4G12790|AT3G05950|AT1G09630|AT4G3655 4070|AT5G59820|AT5G48970|AT4G13920|AT2G31880|AT2G45170|AT3G22910|AT5G39180|AT4G36990|AT4G33450|AT3G04210|AT4G03510| 60|AT2G26190|AT1G61550|AT5G17770|AT3G14090|AT5G54810|AT1G28370|AT5G65790|AT1G15520|AT4G25940|AT1G73805|AT3G25600|AT |AT5G62460|AT1G08050|AT5G60410|AT1G56140|AT4G39940|AT1G24140|AT1G61370|ATMG00080|AT4G23270|AT4G26470|AT3G47780|AT5G AT2G34930|AT2G30600|AT3G18690|AT4G29900|AT4G17785|AT1G74310|AT1G76700|AT3G01990|AT3G06490|AT3G16030|AT3G28580|AT5G0 5G47960|AT4G26120|AT2G34500|AT3G13090|AT1G78830|AT3G14620|AT3G47570|AT4G25434|AT5G03380|AT1G01490|AT3G15430|AT5G526 11840|AT5G62020|AT2G15780|AT3G61390|AT2G46510|AT1G02530|AT3G07780|AT5G54500|AT4G12720|AT3G43210|AT5G39120|AT5G38910 380|AT1G78850|AT2G22300|AT4G32440|AT5G22060|AT2G44790|AT1G09090|AT2G35730|AT1G17310|AT3G57330|AT2G47190|AT3G11820|A 0|AT2G34660|AT1G61360|AT2G39480|AT3G46540|AT3G18930|AT3G44880|AT4G36410|AT2G17730|AT5G66070|AT5G28050|AT1G59910|AT5 AT5G20230|AT4G33940|AT5G13490|AT3G07370|AT5G14420|AT3G59080|AT1G24150|AT5G39670|AT3G08730|AT5G61560|AT3G63380|AT1G7 3G14840|AT1G63180|AT5G39190|AT2G01930|AT2G26650|AT5G03730|AT1G20700|AT3G48850|AT3G22600|AT5G07580|AT4G39830|AT3G200 G42380|AT3G26200|AT3G13430|AT3G06350|AT1G56130|AT4G08500|AT1G14480|AT5G40170|AT3G12040|AT3G45640|AT4G22710|AT1G5581 4470|AT5G56340|AT1G74360|AT2G32660|AT5G39100

VII	20	CELLULAR TRANSPORT, TRANSPORT FACILITATION AND TR/	172	1340	5,4E-05	12.8%	2404 out of 26623 genes, 9%	AT5G52450|AT5G25040|AT1G26380|AT4G31950|AT1G09630|AT2G38290|AT5G04590|AT5G48970|AT2G43400|AT1G15170|AT2G46440|AT5G5<br>4G15330|AT1G07000|AT3G25610|AT5G17770|AT3G48890|AT3G14090|AT1G12360|AT1G76970|AT1G15520|AT1G13180|AT5G41000|AT1G089<br>57220|AT5G61210|AT4G27970|AT5G47730|AT4G18050|AT3G06370|AT2G24180|AT3G15990|AT3G47780|AT1G63440|AT1G59820|AT2G29720<br>140|AT4G35180|AT1G30700|AT2G30750|AT2G41700|AT1G45145|AT3G60540|AT5G27520|AT1G01340|AT4G30210|AT5G47960|AT1G23020|A<br>0|AT5G13740|AT1G71880|AT5G03380|AT1G57980|AT1G01490|AT1G15140|AT4G37640|AT4G36670|AT5G52670|AT1G57990|AT5G40780|AT4<br>AT3G52400|AT4G37370|AT5G11210|AT2G46430|AT1G59870|AT1G26390|AT1G59660|AT3G08710|AT3G47730|AT4G31970|AT4G22690|AT2G4<br>3G11820|AT5G01490|AT2G45570|AT1G02520|AT1G26420|AT5G39040|AT3G26190|AT3G10640|AT1G75170|AT2G02860|AT3G19553|AT1G264<br>58430|AT1G80510|AT4G33520|AT2G40090|AT5G18290|AT5G20230|AT2G46740|AT5G13490|AT4G38540|AT5G47910|AT2G43020|ATMG0099<br>6790|AT5G54860|AT3G16270|AT2G33120|AT2G26650|AT3G48850|AT5G61010|AT1G33110|AT1G30720|AT3G22600|AT2G33110|AT5G52050| 00|AT2G46650|AT1G13100|AT1G19250|AT5G52760|AT2G16380|AT2G46750|AT4G24570|AT2G04040|AT1G62200|AT2G04050|AT1G28480|AT<br>|AT4G22710|AT5G25050|AT2G29990|AT4G21680|AT4G12120|AT3G56200|AT3G29400|AT3G15060|AT5G41610|AT2G07751
VII	30	CELLULAR COMMUNICATION/SIGNAL TRANSDUCTION MEC	117	1340	1,1E-08	8.7%	1235 out of 26623 genes, 4.6%	AT1G51660|AT5G63980|AT1G72920|AT5G01850|AT3G51920|AT1G21230|AT5G45350|AT1G31930|AT2G33130|AT2G48010|AT4G29810|AT3G2<br>2G06050|AT2G31230|AT5G47230|AT4G13920|AT1G35670|AT4G35380|AT1G73500|AT4G34390|AT2G31880|AT1G33600|AT1G76040|AT4G174<br>36150|AT1G66880|AT5G49780|AT5G07370|AT4G11170|AT2G13790|AT1G72900|AT2G13800|AT2G31800|AT1G28370|AT4G18250|AT1G66090<br>870|AT5G40540|AT1G33590|AT4G21380|AT4G11340|AT5G20990|AT5G47220|AT1G73730|AT5G46520|AT2G30360|AT3G45040|AT3G08730|A<br>0|AT5G01950|AT5G59930|AT1G06840|AT3G46930|AT4G18950|AT1G18890|AT1G48260|AT1G72910|AT1G17750|AT2G34930|AT5G03730|AT4<br>AT4G01370|AT5G07580|AT2G34650|AT5G46470|AT1G72940|AT1G72930|AT5G12480|AT1G13910|AT5G17650|AT2G02220|AT5G38210|AT5G4<br>1G01140|AT5G42010|AT3G45640|AT4G39890|AT5G66210|AT3G47570|AT5G04870|AT1G16540|AT5G41750|AT3G20410|AT1G03060|AT3G053<br>04220|AT1G74360|AT1G01560|AT2G32660
VII	32	CELL RESCUE, DEFENSE AND VIRULENCE	176	1340	9,0E-26	13.1%	1349 out of 26623 genes, 5.1%	AT1G51660|AT1G71695|AT4G12720|AT3G46230|AT3G52400|AT5G63980|AT1G72920|AT5G42650|AT4G39980|AT2G38870|AT1G27730|AT1G2<br>3G08710|AT5G44910|AT5G24810|AT5G51630|AT2G06050|AT5G05730|AT2G39660|AT2G16720|AT4G02200|AT1G71697|AT5G59820|AT5G472<br>61900|AT4G13920|AT3G53260|AT1G64280|AT4G02520|AT1G49570|AT1G80820|AT1G14550|AT2G47190|AT2G45170|AT3G11820|AT5G37850<br>150|AT2G29500|AT4G11170|AT5G58720|AT4G37530|AT2G02930|AT2G29420|AT2G02860|AT1G72900|AT5G27760|AT5G13320|AT4G23190|A<br>0|AT1G66090|AT2G29480|AT3G23250|AT2G29470|AT5G45090|AT1G57630|AT4G33420|AT1G68850|AT2G30490|AT2G37040|AT5G44870|AT1<br>AT2G40140|AT1G74590|AT1G02920|AT1G61560|AT5G20230|AT3G07370|AT5G06730|AT4G11340|AT4G08770|AT2G18150|AT1G73730|AT5G4<br>3G61190|AT1G69930|AT3G08730|AT1G69880|AT3G23240|AT2G38470|AT4G20830|AT3G49620|AT1G18570|AT1G54050|AT2G31180|AT1G729<br>74310|AT5G54110|AT4G34150|AT4G36140|AT2G29440|AT3G06490|AT2G32140|AT2G35980|AT5G45250|AT3G56400|AT1G55020|AT3G55270<br>540|AT1G45145|AT5G46470|AT1G72940|AT1G72930|AT3G17510|AT2G24850|AT2G29460|AT3G55730|AT1G57650|AT5G40990|AT4G36430|A<br>0|AT1G64160|AT1G65970|AT3G12040|AT3G45640|AT1G28480|AT1G11310|AT1G06180|AT4G31800|AT1G07400|AT4G23100|AT1G16540|AT5<br>AT5G45260|AT1G71400|AT3G16720|AT4G37520|AT2G15220|AT1G15890|AT3G04220|AT5G06320|AT3G28850|AT1G12520|AT1G21590|AT3G1
VII	34	INTERACTION WITH THE ENVIRONMENT	140	1340	6,1E-11	10.4%	1451 out of 26623 genes, 5.5%	AT1G51660|AT5G54500|AT5G52450|AT4G12720|AT3G46230|AT3G52400|AT5G63980|AT5G42650|AT4G39980|AT3G51920|AT5G57890|AT3G2<br>5G49620|AT4G29810|AT1G59870|AT5G54490|AT5G24810|AT2G06050|AT5G05730|AT2G39660|AT3G26830|AT2G16720|AT2G38290|AT5G551<br>47230|AT1G09090|AT2G43400|AT1G80840|AT5G61900|AT1G64280|AT2G46440|AT4G02520|AT1G80820|AT3G28210|AT2G47190|AT3G11820<br>500|AT3G48450|AT2G29420|AT5G13320|AT4G23190|AT4G11280|AT4G18250|AT2G30870|AT5G65790|AT3G23250|AT3G44880|AT1G15520|A<br>0|AT1G61560|AT5G61210|AT5G20230|AT3G07370|AT1G37130|AT2G18150|AT1G73730|AT5G20830|AT5G47910|AT2G41100|AT1G74710|AT2<br>AT1G79670|AT3G25070|AT2G44080|AT2G38470|AT3G49620|AT1G18570|AT1G54050|AT5G27420|AT2G31180|AT3G16530|AT2G26650|AT1G0<br>4G34150|AT3G06490|AT4G37790|AT1G04100|AT3G28580|AT5G45250|AT3G56400|AT1G55020|AT3G55270|AT4G01370|AT3G08720|AT2G346<br>40990|AT4G21410|AT1G59860|AT1G23020|AT4G08500|AT2G04040|AT3G17700|AT4G11850|AT3G45640|AT1G11310|AT5G13740|AT1G06180<br>880|AT1G16540|AT1G67070|AT4G02380|AT3G04720|AT3G21780|AT5G45260|AT5G06320|AT1G12520|AT1G01560|AT3G12500
VII	36	SYSTEMIC INTERACTION WITH THE ENVIRONMENT	83	1340	3,5E-11	6.2%	664 out of 26623 genes, 2.5%	AT3G61190|AT5G54500|AT4G12720|AT5G42650|AT4G39980|AT1G73080|AT3G22060|AT2G22330|AT2G38870|AT3G25070|AT1G27730|AT2G4<br>5G54490|AT5G27420|AT2G31180|AT2G06050|AT5G05730|AT3G26830|AT2G16720|AT3G18690|AT5G55120|AT1G71697|AT5G03730|AT5G598<br>64280|AT3G06490|AT4G39950|AT4G37790|AT3G28210|AT2G47190|AT1G04100|AT3G28580|AT3G51660|AT3G56400|AT1G55020|AT4G01370<br>250|AT2G02860|AT2G29420|AT5G40990|AT4G21410|AT5G13320|AT4G23190|AT4G11280|AT4G08500|AT5G65790|AT3G23250|AT3G44880|A<br>0|AT1G11310|AT1G06180|AT4G31800|AT1G25220|AT4G23100|AT5G27380|AT1G61560|AT5G20230|AT5G60410|AT3G07370|AT3G04720|AT3<br>AT1G74710|AT2G44490
VII	01.01	amino acid metabolism	47	1340	9,3E-08	3.5%	335 out of 26623 genes, 1.3%	AT4G35630|AT1G65520|AT1G18500|AT1G05010|AT5G57890|AT3G06350|AT5G13280|AT2G22330|AT1G03090|AT1G48850|AT2G22250|AT4G1<br>1G50090|AT2G37040|AT5G05730|AT4G27070|AT1G08630|AT5G66120|AT3G44720|AT1G25220|AT1G77670|AT3G25900|AT2G31810|AT2G020<br>19030|AT3G16150|AT1G01480|AT4G37670|AT4G39540|AT3G10340|AT2G16500|AT1G17290|AT1G72330|AT4G31500|AT5G17990|AT3G54640
VII	01.01.03	assimilation of ammonia, metabolism of the glutamate gro	9	1340	2,0E-02	0.7%	57 out of 26623 genes, 0.2%	AT4G37670|AT2G22250|AT2G16500|AT2G02010|AT3G53180|AT1G17290|AT2G02000|AT1G72330|AT5G11520
VII	01.01.03.02	metabolism of glutamate	7	1340	1,0E-02	0.5%	34 out of 26623 genes, 0.1%	AT2G22250|AT2G02010|AT3G53180|AT1G17290|AT2G02000|AT1G72330|AT5G11520
VII	01.01.06	metabolism of the aspartate family	11	1340	2,0E-02	0.8%	84 out of 26623 genes, 0.3%	AT3G25900|AT1G65520|AT1G05010|AT5G11520|AT5G13280|AT1G08630|AT2G22250|AT4G11280|AT3G16150|AT1G01480|AT1G77670
VII	01.01.09	metabolism of the cysteine - aromatic group	23	1340	1,8E-05	1.7%	132 out of 26623 genes, 0.5%	AT4G35630|AT3G53260|AT5G57890|AT3G06350|AT2G22330|AT4G39950|AT1G48850|AT3G19030|AT5G54810|AT4G39540|AT3G10340|AT2G0<br>5G66120|AT4G31500|AT3G44720|AT5G17990|AT3G54640|AT1G25220|AT2G24850
VII	01.01.09.04	metabolism of phenylalanine	9	1340	1,1E-03	0.7%	33 out of 26623 genes, 0.1%	AT2G37040|AT1G48850|AT4G39540|AT3G10340|AT5G66120|AT3G44720|AT3G53260|AT2G24850|AT3G06350
VII	01.01.09.05	metabolism of tyrosine	5	1340	5,0E-02	0.4%	27 out of 26623 genes, 0.1%	AT1G48850|AT4G39540|AT5G66120|AT2G24850|AT3G06350
VII	01.01.09.06	metabolism of tryptophan	15	1340	1,4E-06	1.1%	44 out of 26623 genes, 0.2%	AT2G22330|AT4G39950|AT5G05730|AT1G48850|AT4G39540|AT4G27070|AT5G66120|AT4G31500|AT5G17990|AT5G54810|AT3G54640|AT5G5
VII	01.01.09.06.0	biosynthesis of tryptophan	10	1340	7,5E-05	0.7%	27 out of 26623 genes, 0.1%	AT5G17990|AT5G05730|AT3G54640|AT5G54810|AT4G27070|AT5G57890|AT5G66120|AT1G25220|AT4G31500|AT2G04400
VII	01.01.11	metabolism of the pyruvate family (alanine, isoleucine, leu	8	1340	1,0E-02	0.6%	42 out of 26623 genes, 0.2%	AT1G03090|AT1G55510|AT1G18500|AT2G31810|AT1G17290|AT1G21400|AT1G72330|AT1G50090
VII	01.01.11.04	metabolism of leucine	5	1340	4,0E-02	0.4%	23 out of 26623 genes, 0.1%	AT1G03090|AT1G55510|AT1G21400|AT1G18500|AT1G50090
VII	01.01.11.04.0	degradation of leucine	3	1340	5,0E-02	0.2%	9 out of 26623 genes, 0%	AT1G03090|AT1G55510|AT1G21400
VII	01.02	nitrogen, sulfur and selenium metabolism	22	1340	5,6E-04	1.6%	159 out of 26623 genes, 0.6%	AT5G04590|AT1G16540|AT5G63980|AT3G53180|AT5G11520|AT3G22890|AT3G16150|AT1G37130|AT5G22300|AT3G10340|AT2G14750|AT1G7<br>5G07010|AT4G04610|AT5G28050|AT2G43020|AT2G24850|AT1G77670
VII	01.02.02	nitrogen metabolism	10	1340	5,0E-02	0.7%	83 out of 26623 genes, 0.3%	AT3G10340|AT3G53180|AT5G11520|AT2G20610|AT3G16150|AT2G43020|AT1G37130|AT5G22300|AT2G24850|AT1G77670
VII	01.02.03	sulfur metabolism	11	1340	1,0E-02	0.8%	72 out of 26623 genes, 0.3%	AT5G04590|AT2G14750|AT5G63980|AT1G74100|AT4G39940|AT1G73730|AT1G18590|AT3G22890|AT5G07010|AT4G04610|AT5G28050
VII	01.04	phosphate metabolism	207	1340	3,6E-23	15.4%	1845 out of 26623 genes, 6.9%	AT5G47070|AT1G51660|AT1G51890|AT5G20050|AT4G04540|AT5G63980|AT3G12200|AT5G01850|AT1G11050|AT1G21230|AT5G13280|AT3G1<br>1G59870|AT2G01180|AT3G47730|AT4G28490|AT4G29220|AT3G21630|AT5G24430|AT3G56050|AT3G59700|AT2G39660|AT5G15730|AT5G661<br>35600|AT3G23150|AT4G25390|AT4G13920|AT1G35670|AT4G21400|AT1G73500|AT2G31880|AT3G09830|AT1G14370|AT1G76040|AT1G57700<br>960|AT1G02520|AT2G35390|AT1G66880|AT3G57750|AT4G32300|AT1G18390|AT5G49780|AT5G07370|AT5G39040|AT1G61550|AT2G13790|A<br>0|AT3G17410|AT4G23190|AT2G33580|AT1G61360|AT2G39480|AT4G18250|AT5G58940|AT1G21210|AT1G32380|AT1G15520|AT3G62150|AT5<br>AT4G04570|AT3G02750|AT5G40540|AT2G23450|AT2G11520|AT3G59350|AT2G28940|AT4G26270|AT5G51260|AT4G21380|AT1G25390|AT3G2<br>1G69960|AT1G53430|AT1G56140|AT2G14750|AT1G56120|AT4G39940|AT2G30360|AT4G18050|AT1G61370|AT4G11480|AT3G55950|AT4G213<br>08730|AT5G61560|AT3G50930|AT1G73080|AT5G51070|AT5G58350|AT2G46500|AT1G53390|AT1G79670|AT4G30490|AT4G35310|AT1G56145<br>350|AT1G06840|AT3G46930|AT4G18950|AT1G53440|AT1G18890|AT1G48260|AT1G17750|AT1G70740|AT5G03730|AT1G22280|AT1G74310|A<br>0|AT3G55270|AT4G01370|AT1G03740|AT1G10700|AT4G23180|AT3G08720|AT2G41880|AT5G67080|AT5G65240|AT3G20040|AT2G34650|AT3<br>AT3G17510|AT2G17530|AT3G20860|AT5G60280|AT2G02220|AT4G21410|AT5G04540|AT5G38210|AT4G11470|AT2G20850|AT5G25930|AT5G4<br>5G40170|AT2G23770|AT1G16670|AT1G01140|AT3G13090|AT5G35580|AT3G45640|AT5G39020|AT5G66210|AT3G47570|AT1G68690|AT4G225<br>20410|AT4G33920|AT1G55810|AT3G05360|AT4G39270|AT1G69790|AT1G79680|AT1G74360|AT1G01560|AT1G21590|AT1G02530|AT2G32660
VII	01.05	C-compound and carbohydrate metabolism	111	1340	6,4E-03	8.3%	1625 out of 26623 genes, 6.1%	AT5G03490|AT4G39980|AT1G24100|AT5G12890|AT4G27830|AT1G53990|AT3G02360|AT3G06500|AT1G02310|AT5G26000|AT2G38180|AT4G2<br>2G36970|AT1G67880|AT3G49160|AT3G07970|AT5G14930|AT3G28340|AT2G36790|AT4G19460|AT1G02850|AT1G07240|AT5G44480|AT1G711<br>43870|AT1G33030|AT1G80160|AT4G11280|AT2G15390|AT2G30140|AT2G36800|AT1G32170|AT1G54100|AT4G25810|AT2G36770|AT5G56300<br>270|AT5G51260|AT1G18270|AT1G09490|AT2G19800|AT2G43570|AT4G24380|AT2G39410|AT1G61820|AT1G22410|AT5G20830|AT4G23730|A<br>0|AT1G23730|AT2G39030|AT1G66700|AT5G41080|AT3G28180|AT5G57500|AT1G63180|AT5G16910|AT1G22400|AT5G53990|AT1G66690|AT1<br>AT1G01480|AT4G16260|AT2G28080|AT3G20040|AT5G11650|AT5G40850|AT5G40990|AT1G30620|AT3G06350|AT5G53050|AT2G44460|AT2G1<br>5G25980|AT2G31810|AT2G02010|AT4G24160|AT1G23870|AT1G67070|AT1G61810|AT4G01700|AT4G12250|AT3G21780|AT1G09480|AT2G394
VII	01.05.02	sugar, glucoside, polyol and carboxylate metabolism	59	1340	4,0E-02	4.4%	846 out of 26623 genes, 3.2%	AT5G03490|AT1G24100|AT5G12890|AT4G27830|AT3G02360|AT3G28180|AT5G57500|AT5G16910|AT1G63180|AT1G22400|AT5G53990|AT1G0<br>2G36970|AT3G49160|AT3G60120|AT3G28340|AT1G55850|AT2G36790|AT4G19460|AT3G09020|AT2G15480|AT1G02850|AT4G16260|AT1G072<br>72330|AT2G28080|AT3G20040|AT1G30620|AT2G15390|AT2G30140|AT2G36800|AT2G36770|AT2G44460|AT2G15490|AT4G25900|AT3G53150<br>070|AT1G18270|AT1G61810|AT4G12250|AT2G19800|AT3G21780|AT1G61820|AT5G20830|AT4G23730|AT2G44480|AT2G44490
VII	01.05.02.01	nucleotide-sugar metabolism	22	1340	2,8E-03	1.6%	185 out of 26623 genes, 0.7%	AT3G53150|AT2G36790|AT5G03490|AT1G24100|AT1G30620|AT2G15480|AT5G12890|AT4G12250|AT3G21780|AT1G63180|AT2G30140|AT2G3<br>5G20830|AT2G36780|AT2G15490|AT3G53160|AT2G36970|AT1G67880
VII	01.05.03.03	chitin metabolism	6	1340	2,0E-03	0.4%	14 out of 26623 genes, 0.1%	AT3G47540|AT2G43590|AT2G43620|AT3G12500|AT2G43570|AT4G01700
VII	01.05.03.03.0	chitin catabolism	6	1340	2,0E-03	0.4%	14 out of 26623 genes, 0.1%	AT3G47540|AT2G43590|AT2G43620|AT3G12500|AT2G43570|AT4G01700
VII	01.05.11	aromate metabolism	12	1340	8,1E-04	0.9%	57 out of 26623 genes, 0.2%	AT1G48860|AT5G11650|AT5G66120|AT2G39410|AT4G24160|AT1G22410|AT4G39980|AT2G39400|AT2G45300|AT5G53050|AT3G06350|AT1G7
VII	01.05.11.04	aromate anabolism	6	1340	1,7E-04	0.4%	7 out of 26623 genes, 0%	AT2G45300|AT3G06350|AT1G22410|AT4G39980|AT1G48860|AT5G66120
VII	01.06.02.03	sphingolipid metabolism	3	1340	3,0E-02	0.2%	6 out of 26623 genes, 0%	AT3G48780|AT3G48790|AT4G36480
VII	01.20	secondary metabolism	80	1340	4,6E-20	6%	410 out of 26623 genes, 1.5%	AT5G63570|AT1G72680|AT1G69930|AT1G33030|AT2G02930|AT5G42650|AT1G05010|AT2G29420|AT4G39980|AT1G24100|AT4G30210|AT3G0<br>1G20510|AT5G54810|AT1G27130|AT2G30870|AT2G29480|AT2G36800|AT2G29470|AT1G64160|AT1G51780|AT5G26000|AT4G10500|AT5G248<br>37040|AT1G02930|AT1G75450|AT2G06050|AT5G05730|AT1G08630|AT5G66120|AT1G69740|AT3G26830|AT1G25220|AT2G45300|AT1G74590<br>380|AT1G16540|AT4G34230|AT5G25980|AT3G53260|AT2G29440|AT1G09490|AT4G39950|AT4G02520|AT1G80820|AT1G03475|AT4G39640|A<br>0|AT4G39540|AT1G17170|AT2G16500|AT5G35735|AT3G19010|AT1G09480|AT1G22410|AT1G51760|AT2G20610|AT1G09940|AT4G31500|AT1

VII	01.20.15	metabolism of derivatives of dehydroquinic acid, shikimic a	8	1340	2,7E-04	0.6%	19 out of 26623 genes, 0.1%	AT1G22410|AT1G48860|AT4G39980|AT1G48850|AT4G39540|AT5G66120|AT2G45300|AT3G06350
VII	01.20.19	metabolism of secondary products derived from glycine, L-	9	1340	2,0E-02	0.7%	58 out of 26623 genes, 0.2%	AT1G09940|AT5G44070|AT1G08630|AT5G63570|AT1G69740|AT1G16540|AT5G40850|AT1G03475|AT5G57190
VII	01.20.33	metabolism of secondary products derived from L-tryptopl	10	1340	5,0E-04	0.7%	36 out of 26623 genes, 0.1%	AT2G22330|AT4G39950|AT5G05730|AT4G23100|AT4G31500|AT3G26830|AT5G54810|AT1G25220|AT3G56400|AT2G20610
VII	01.20.35	metabolism of secondary products derived from L-phenylal	17	1340	2,9E-03	1.3%	124 out of 26623 genes, 0.5%	AT5G35735|AT1G64160|AT3G19010|AT4G34230|AT1G72680|AT1G33030|AT1G09480|AT3G53260|AT1G76470|AT4G30210|AT1G09490|AT2G3<br>1G20510
VII	01.20.35.01	metabolism of phenylpropanoids	15	1340	6,4E-03	1.1%	112 out of 26623 genes, 0.4%	AT1G64160|AT3G19010|AT4G34230|AT1G72680|AT1G33030|AT1G09480|AT3G53260|AT1G76470|AT4G30210|AT1G09490|AT2G33600|AT2G3
VII	01.20.35.01.0	metabolism of lignins	9	1340	6,4E-03	0.7%	46 out of 26623 genes, 0.2%	AT1G80820|AT1G64160|AT1G72680|AT4G34230|AT1G33030|AT1G09480|AT1G76470|AT1G09490|AT2G33600
VII	01.20.37	metabolism of peptide derived compounds	5	1340	2,0E-02	0.4%	17 out of 26623 genes, 0.1%	AT4G23100|AT5G24810|AT4G39640|AT5G05600|AT5G27380
VII	01.20.37.01	metabolism of thioredoxin, glutaredoxin, glutathion	3	1340	5,0E-02	0.2%	8 out of 26623 genes, 0%	AT5G27380|AT4G23100|AT4G39640
VII	01.20.38	metabolism of toxins/drugs	15	1340	2,4E-06	1.1%	47 out of 26623 genes, 0.2%	AT2G29480|AT1G74590|AT1G02930|AT1G02920|AT4G02520|AT1G17170|AT2G29470|AT1G69930|AT2G02930|AT1G69920|AT2G29420|AT2G2
VII	04.01	storage facilitating proteins	12	1340	2,1E-03	0.9%	65 out of 26623 genes, 0.2%	AT5G38910|AT5G39180|AT1G18970|AT5G39150|AT5G39110|AT3G05950|AT5G39130|AT5G39120|AT5G39160|AT2G26560|AT5G39100|AT5G3
VII	14.07	protein modification	196	1340	2,2E-13	14.6%	2142 out of 26623 genes, 8%	AT5G20050|AT4G04540|AT1G21230|AT4G29810|AT3G26020|AT4G28490|AT4G36550|AT5G15730|AT1G67880|AT4G14220|AT4G25390|AT1G3<br>1G57700|AT1G19530|AT1G07630|AT1G54960|AT4G03510|AT1G66880|AT4G32300|AT5G49780|AT1G14260|AT2G26190|AT1G61550|AT2G137<br>23190|AT2G33580|AT4G18250|AT5G58940|AT2G17110|AT1G18480|AT5G49760|AT2G28940|AT4G21380|AT3G25250|AT3G05050|AT2G05940<br>430|AT1G56140|AT1G61370|AT4G11480|AT4G21390|AT4G23270|AT4G26470|AT4G27290|AT4G28400|AT5G67250|AT5G51070|AT1G56145|A<br>0|AT1G17750|AT1G70740|AT1G22280|AT5G65600|AT2G40270|AT3G16030|AT3G08720|AT2G34650|AT4G33910|AT3G53810|AT3G57760|AT2<br>AT2G20850|AT3G28450|AT5G39020|AT5G66210|AT3G47570|AT5G04870|AT1G16540|AT3G53180|AT3G08760|AT3G20410|AT4G33920|AT4G3<br>3G28850|AT3G11840|AT1G21590|AT3G61390|AT5G64660|AT5G47070|AT1G51660|AT1G51890|AT3G12200|AT5G01850|AT1G11050|AT1G670<br>56050|AT3G59700|AT2G39660|AT4G35230|AT4G35600|AT3G23150|AT1G73500|AT4G21400|AT1G76040|AT3G57750|AT1G18390|AT5G60710<br>290|AT4G36410|AT2G34180|AT5G57190|AT1G11330|AT4G04570|AT4G34210|AT3G02750|AT5G40540|AT2G23450|AT2G11520|AT3G59350|A<br>0|AT1G56120|AT2G30360|AT1G66480|AT3G55950|AT3G08730|AT1G69880|AT5G61560|AT1G73080|AT5G58350|AT2G46500|AT4G35310|AT1<br>AT3G46930|AT1G18890|AT5G03730|AT3G55270|AT5G67340|AT4G01370|AT1G03740|AT4G23180|AT5G17350|AT5G67080|AT5G65240|AT5G5<br>5G38210|AT5G25930|AT5G46080|AT1G56130|AT4G08500|AT5G44290|AT2G23770|AT1G16670|AT1G01140|AT1G66160|AT5G35580|AT3G456<br>01560|AT3G26980
VII	14.07.01	modification with fatty acids (e.g. myristylation, palmitylati	40	1340	4,6E-03	3%	449 out of 26623 genes, 1.7%	AT4G31020|AT2G26190|AT1G69880|AT5G67250|AT2G07180|AT4G35310|AT3G17410|AT1G67800|AT5G44290|AT5G02290|AT3G08710|AT2G1<br>2G39660|AT4G35230|AT3G02750|AT5G04870|AT4G22710|AT5G27380|AT4G35600|AT3G53180|AT3G20410|AT3G05050|AT5G14420|AT1G577<br>17350|AT1G08800|AT3G28850|AT1G66480|AT5G60710|AT3G01650|AT5G12480|AT4G26470
VII	14.07.03	modification by phosphorylation, dephosphorylation, autoj	150	1340	2,6E-19	11.2%	1235 out of 26623 genes, 4.6%	AT5G47070|AT1G51660|AT1G51890|AT5G20050|AT4G04540|AT3G12200|AT5G01850|AT1G11050|AT1G21230|AT1G67000|AT2G48010|AT4G2<br>3G56050|AT3G59700|AT2G39660|AT5G15730|AT4G35230|AT4G35600|AT3G23150|AT4G25390|AT1G35670|AT4G21400|AT1G73500|AT2G318<br>07630|AT1G54960|AT1G66880|AT3G57750|AT4G32300|AT1G18390|AT5G49780|AT1G61550|AT2G13790|AT2G13800|AT5G06740|AT2G31800<br>360|AT4G18250|AT5G58940|AT1G21210|AT5G02290|AT2G34180|AT1G18480|AT5G49760|AT1G11330|AT4G04570|AT3G02750|AT5G40540|A<br>0|AT1G25390|AT3G25250|AT3G09010|AT3G05050|AT2G05940|AT5G58540|AT1G69960|AT1G53430|AT1G56140|AT1G56120|AT2G30360|AT1<br>AT4G27290|AT4G28400|AT3G08730|AT5G61560|AT1G73080|AT5G58350|AT4G35310|AT1G56145|AT3G14840|AT1G53570|AT5G01950|AT4G2<br>1G48260|AT1G18890|AT1G17750|AT1G70740|AT5G03730|AT1G22280|AT5G65600|AT2G40270|AT3G16030|AT3G55270|AT4G01370|AT1G037<br>34650|AT3G53810|AT5G59700|AT3G57760|AT5G12480|AT2G17530|AT3G20860|AT5G60280|AT2G02220|AT5G04540|AT5G38210|AT4G11470<br>500|AT5G44290|AT3G28450|AT2G23770|AT1G16670|AT1G01140|AT5G35580|AT3G45640|AT5G39020|AT5G66210|AT3G47570|AT5G04870|A<br>0|AT1G79680|AT1G74360|AT1G01560|AT1G21590
VII	16.13	C-compound binding	15	1340	3,0E-04	1.1%	77 out of 26623 genes, 0.3%	AT4G28350|AT1G78860|AT1G61550|AT5G18470|AT3G16530|AT1G61370|AT5G06740|AT1G78820|AT1G11330|AT4G32300|AT3G16030|AT4G2
VII	16.13.01	sugar binding	6	1340	7,9E-03	0.4%	21 out of 26623 genes, 0.1%	AT1G78850|AT5G18470|AT1G78820|AT1G78860|AT4G21390|AT1G78830
VII	16.17	metal binding	100	1340	2,7E-04	7.5%	1281 out of 26623 genes, 4.8%	AT1G72680|AT3G51920|AT1G19310|AT5G39120|AT1G27730|AT3G05200|AT5G38910|AT5G54490|AT5G39110|AT5G24430|AT3G05950|AT1G1<br>5G59820|AT4G11370|AT1G09090|AT2G35730|AT5G52750|AT3G28210|AT5G39180|AT5G59940|AT4G03510|AT5G39130|AT3G18290|AT5G607<br>17145|AT3G18930|AT2G17730|AT3G25600|AT5G66070|AT5G04340|AT5G28050|AT5G04170|AT4G09100|AT3G47480|AT1G70670|AT1G18210<br>050|AT5G20990|AT5G42940|AT1G24140|AT2G22010|AT2G41100|AT4G26470|AT1G23730|AT5G39670|AT1G70680|AT1G66400|AT5G39190|A<br>0|AT1G22510|AT5G59550|AT2G22690|AT5G05600|AT3G53600|AT4G26400|AT2G32250|AT5G07390|AT1G76650|AT4G39830|AT2G36950|AT1<br>AT1G21550|AT3G47550|AT2G18670|AT1G32530|AT2G37150|AT1G02860|AT3G43890|AT5G03380|AT1G01490|AT5G44090|AT5G52670|AT3G1<br>2G15780|AT3G07780|AT5G39100
VII	16.17.01	calcium binding	19	1340	1,0E-02	1.4%	172 out of 26623 genes, 0.6%	AT5G39670|AT5G42380|AT1G70670|AT3G47480|AT1G09090|AT1G18210|AT3G51920|AT1G70680|AT1G66400|AT1G21550|AT5G44090|AT5G5<br>2G41100|AT5G04170|AT4G26470
VII	16.19.01	cyclic nucleotide binding (cAMP, cGMP, etc.)	6	1340	2,0E-02	0.4%	29 out of 26623 genes, 0.1%	AT1G01340|AT2G46430|AT2G26650|AT2G46440|AT4G01010|AT3G17700
VII	16.19.03	ATP binding	82	1340	2,0E-02	6.1%	1209 out of 26623 genes, 4.5%	AT3G13100|AT2G46620|AT1G59870|AT3G47730|AT4G28490|AT4G12790|AT5G51630|AT4G36550|AT1G43910|AT4G14220|AT2G31880|AT1G0<br>1G02520|AT4G03510|AT5G49780|AT5G60710|AT4G11170|AT1G14260|AT5G39040|AT5G58720|AT2G13800|AT2G34660|AT2G39480|AT1G660<br>50950|AT4G34210|AT1G08050|AT3G07370|AT5G14420|AT2G14750|AT4G39940|AT5G46520|AT4G18050|AT4G23270|AT3G47780|AT5G67250<br>490|AT3G14840|AT5G01950|AT1G06840|AT1G11100|AT1G17750|AT1G74310|AT4G36140|AT3G28580|AT2G41700|AT5G67340|AT3G20040|A<br>0|AT3G28450|AT1G66160|AT3G13090|AT3G47570|AT5G09800|AT5G41750|AT3G14470|AT1G55810|AT4G39270|AT4G34470|AT1G15890|AT3<br>AT5G64660
VII	16.25	oxygen binding	24	1340	7,1E-03	1.8%	231 out of 26623 genes, 0.9%	AT4G22710|AT3G26200|AT5G42650|AT5G57220|AT1G13100|AT5G36220|AT2G30750|AT2G22330|AT4G39950|AT4G37370|AT2G30770|AT5G6<br>4G15330|AT4G31500|AT3G26830|AT4G22690|AT2G24180|AT3G14620|AT2G27690|AT3G26190
VII	18.01	regulation by	35	1340	1,0E-02	2.6%	402 out of 26623 genes, 1.5%	AT4G27290|AT4G28400|AT3G08730|AT2G13790|AT5G58350|AT5G04540|AT2G13800|AT4G35310|AT4G11470|AT1G56145|AT4G23190|AT3G1<br>3G02750|AT1G22280|AT5G04870|AT3G23150|AT1G35670|AT3G20410|AT4G33920|AT1G76040|AT1G69960|AT1G07630|AT4G01370|AT1G561<br>57760|AT5G12480|AT4G23270
VII	18.01.01	regulation by modification	35	1340	4,6E-03	2.6%	376 out of 26623 genes, 1.4%	AT4G27290|AT4G28400|AT3G08730|AT2G13790|AT5G58350|AT5G04540|AT2G13800|AT4G35310|AT4G11470|AT1G56145|AT4G23190|AT3G1<br>3G02750|AT1G22280|AT5G04870|AT3G23150|AT1G35670|AT3G20410|AT4G33920|AT1G76040|AT1G69960|AT1G07630|AT4G01370|AT1G561<br>57760|AT5G12480|AT4G23270
VII	18.02	regulation of protein activity	43	1340	5,0E-02	3.2%	598 out of 26623 genes, 2.2%	AT4G27290|AT4G28400|AT3G08730|AT5G58350|AT2G20900|AT2G38870|AT4G35310|AT1G56145|AT3G14840|AT3G26020|AT1G18890|AT4G3<br>1G76040|AT1G07630|AT4G01370|AT3G08720|AT5G38640|AT2G34650|AT3G57760|AT5G12480|AT2G13790|AT5G04540|AT2G13800|AT1G732<br>44070|AT3G02750|AT5G04870|AT4G16500|AT3G20410|AT4G33920|AT1G69960|AT1G56140|AT1G01560|AT4G21390|AT4G23270
VII	20.01	transported compounds (substrates)	133	1340	8,1E-04	9.9%	1881 out of 26623 genes, 7.1%	AT5G54500|AT3G52400|AT4G37370|AT2G46430|AT1G26380|AT1G59870|AT1G26390|AT3G08710|AT4G31950|AT4G31970|AT1G09630|AT4G2<br>2G43400|AT1G15170|AT2G35730|AT2G46440|AT3G57330|AT5G52750|AT3G11820|AT3G22910|AT5G04930|AT5G01490|AT5G67310|AT2G455<br>25610|AT2G02860|AT5G17770|AT3G19553|AT1G26410|AT3G48890|AT1G12360|AT1G76970|AT2G35660|AT1G15520|AT5G41000|AT1G08930<br>510|AT4G33520|AT5G57220|AT5G61210|AT5G20230|AT2G46740|AT5G13490|AT4G38540|AT5G47910|AT3G06370|AT2G24180|AT3G15990|A<br>80|AT1G59820|AT3G63380|AT5G36220|AT4G36790|AT2G29720|AT4G20830|AT3G16270|AT2G30770|AT2G26650|AT5G59540|AT4G29900|AT<br>|AT1G30720|AT3G22600|AT2G41700|AT5G52050|AT1G45145|AT3G60540|AT1G34200|AT1G13210|AT2G36950|AT3G26200|AT2G46650|AT1G<br>T5G52760|AT1G23020|AT2G46750|AT2G04040|AT1G62200|AT2G04050|AT2G34500|AT1G28480|AT4G39890|AT4G01010|AT1G12200|AT3G14<br>G22710|AT5G03380|AT1G57980|AT1G15140|AT1G01490|AT2G29990|AT4G37640|AT4G36670|AT4G21680|AT5G52670|AT1G57990|AT5G4078<br>8850|AT5G41610|AT2G15780|AT2G07751
VII	20.01.15	electron transport	62	1340	6,2E-04	4.6%	717 out of 26623 genes, 2.7%	AT1G32350|AT5G54500|AT1G69880|AT3G63380|AT5G36220|AT4G37370|AT2G29720|AT1G26380|AT4G20830|AT1G26390|AT2G30770|AT3G0<br>2G44790|AT5G04590|AT2G43400|AT1G30700|AT2G30750|AT1G30720|AT3G57330|AT3G22910|AT5G67310|AT2G45570|AT1G45145|AT1G264<br>46650|AT1G13100|AT1G19250|AT5G17770|AT1G26410|AT3G48890|AT4G30210|AT2G35660|AT1G23020|AT2G46750|AT2G34500|AT1G28480<br>710|AT5G57220|AT1G15140|AT2G29990|AT5G20230|AT2G46740|AT4G38540|AT5G47910|AT3G28850|AT2G15780|AT2G24180|AT2G43020|A
VII	20.01.27	drug/toxin transport	9	1340	3,0E-02	0.7%	64 out of 26623 genes, 0.2%	AT1G15520|AT2G04040|AT5G52050|AT4G23030|AT1G15170|AT1G71140|AT2G04050|AT1G33110|AT1G59870
VII	20.03	transport facilities	51	1340	2,6E-03	3.8%	596 out of 26623 genes, 2.2%	AT5G25040|AT5G52450|AT3G47780|AT1G63440|AT1G59820|AT3G63380|AT5G54860|AT5G11210|AT2G46430|AT1G59660|AT3G47730|AT2G2<br>2G46440|AT1G33110|AT3G57330|AT3G22910|AT5G04930|AT5G01490|AT5G52050|AT1G13210|AT5G39040|AT1G75170|AT3G25610|AT2G028<br>62200|AT2G04050|AT4G01010|AT3G18830|AT5G33320|AT1G71880|AT5G01500|AT4G33520|AT5G25050|AT2G40090|AT5G18290|AT4G37640<br>030|AT5G41610|AT3G06370
VII	20.03.22	transport ATPases	11	1340	8,5E-03	0.8%	70 out of 26623 genes, 0.3%	AT3G22910|AT5G04930|AT3G25610|AT4G33520|AT1G63440|AT1G59820|AT4G37640|AT3G63380|AT3G57330|AT1G13210|AT4G29900
VII	20.09.07.27	vesicle fusion	9	1340	1,0E-02	0.7%	53 out of 26623 genes, 0.2%	AT5G61210|AT3G14090|AT1G07000|AT3G29400|AT5G58430|AT3G52400|AT5G16830|AT5G61010|AT3G11820
VII	20.09.16	cellular export and secretion	10	1340	1,0E-02	0.7%	61 out of 26623 genes, 0.2%	AT3G14090|AT1G07000|AT1G12360|AT2G04040|AT2G29410|AT3G29400|AT5G58430|AT4G12120|AT5G61010|AT4G03510
VII	20.09.16.09	vesicular cellular export	5	1340	5,0E-02	0.4%	26 out of 26623 genes, 0.1%	AT3G14090|AT1G07000|AT3G29400|AT5G61010|AT5G58430
VII	20.09.16.09.0	exocytosis	5	1340	5,0E-02	0.4%	26 out of 26623 genes, 0.1%	AT3G14090|AT1G07000|AT3G29400|AT5G61010|AT5G58430
VII	30.01	cellular signalling	70	1340	5,7E-04	5.2%	836 out of 26623 genes, 3.1%	AT1G51660|AT5G63980|AT3G08730|AT5G01850|AT3G51920|AT1G21230|AT1G73080|AT3G23240|AT2G46500|AT4G35310|AT2G44080|AT2G3<br>3G46930|AT4G18950|AT4G28490|AT1G18890|AT1G09630|AT2G06050|AT1G17750|AT2G31230|AT5G03730|AT5G47230|AT1G35670|AT4G353<br>17490|AT3G55270|AT4G01370|AT1G54960|AT5G07580|AT5G59940|AT1G66880|AT2G34650|AT5G49780|AT5G07370|AT5G12480|AT2G02220<br>500|AT3G28450|AT1G21210|AT4G11850|AT5G42010|AT3G45640|AT4G39890|AT5G66210|AT3G47570|AT5G40540|AT5G04870|AT1G16540|A<br>0|AT3G45040|AT1G64610|AT3G15060|AT1G74360|AT1G01560
VII	30.01.05	enzyme mediated signal transduction	40	1340	3,6E-03	3%	442 out of 26623 genes, 1.7%	AT1G51660|AT2G02220|AT3G08730|AT5G01850|AT1G21230|AT1G73080|AT5G38210|AT5G47960|AT2G31800|AT5G01950|AT4G08500|AT4G2<br>4G18950|AT4G28490|AT5G42010|AT1G09630|AT3G45640|AT1G17750|AT4G39890|AT3G47570|AT5G40540|AT5G03730|AT4G35380|AT1G735<br>54960|AT3G15060|AT1G64610|AT1G74360|AT1G66880|AT1G01560|AT2G34650|AT5G49780
VII	30.01.05.01	protein kinase	33	1340	1,7E-04	2.5%	276 out of 26623 genes, 1%	AT1G51660|AT2G02220|AT3G08730|AT5G01850|AT1G21230|AT1G73080|AT5G38210|AT2G31800|AT5G01950|AT4G08500|AT4G29810|AT1G2<br>4G28490|AT3G45640|AT1G17750|AT3G47570|AT5G40540|AT5G03730|AT1G73500|AT2G31880|AT4G39270|AT3G55270|AT4G01370|AT1G549<br>49780
VII	30.01.05.01.0	MAPKKK cascade	9	1340	2,1E-03	0.7%	37 out of 26623 genes, 0.1%	AT1G51660|AT1G01560|AT3G55270|AT4G01370|AT3G45640|AT1G54960|AT1G73500|AT4G08500|AT4G29810
VII	30.01.05.01.0	serine/threonine kinase	19	1340	2,0E-02	1.4%	182 out of 26623 genes, 0.7%	AT2G02220|AT3G08730|AT1G21230|AT1G73080|AT5G38210|AT2G31880|AT4G39270|AT5G01950|AT3G28450|AT1G21210|AT1G06840|AT4G2<br>5G49780|AT3G47570|AT5G03730
VII	30.01.09	second messenger mediated signal transduction	29	1340	2,0E-02	2.2%	328 out of 26623 genes, 1.2%	AT5G63980|AT3G51920|AT3G23240|AT2G46500|AT4G35310|AT2G44080|AT1G28370|AT4G11850|AT1G18890|AT2G06050|AT3G45640|AT5G6<br>1G35670|AT4G11840|AT3G20410|AT1G76040|AT4G17490|AT5G20990|AT5G47220|AT1G73730|AT3G45040|AT5G07580|AT2G34650|AT5G073
VII	30.01.09.03	Ca2+ mediated signal transduction	9	1340	7,0E-03	0.7%	47 out of 26623 genes, 0.2%	AT5G04870|AT4G35310|AT5G66210|AT1G76040|AT5G12480|AT3G51920|AT1G35670|AT3G20410|AT1G18890
VII	30.05	transmembrane signal transduction	44	1340	4,7E-07	3.3%	324 out of 26623 genes, 1.2%	AT5G17650|AT2G02220|AT1G72920|AT2G13790|AT1G72900|AT1G73080|AT2G13800|AT5G45350|AT1G31930|AT5G01950|AT4G18250|AT2G4<br>4G28490|AT1G57630|AT5G44910|AT5G51630|AT5G44870|AT1G72910|AT1G17750|AT3G47570|AT4G21380|AT4G34150|AT4G36140|AT5G417<br>11340|AT5G41740|AT5G46520|AT3G04220|AT3G04210|AT4G36150|AT1G74360|AT5G46470|AT5G49780|AT4G11170|AT1G72940|AT1G72930
VII	30.05.01	receptor enzyme mediated signalling	39	1340	1,5E-06	2.9%	281 out of 26623 genes, 1.1%	AT2G02220|AT1G72920|AT2G13790|AT1G72900|AT1G73080|AT2G13800|AT5G01950|AT4G18250|AT2G48010|AT1G66090|AT3G28450|AT1G0<br>5G51630|AT5G44870|AT1G72910|AT1G17750|AT3G47570|AT4G21380|AT4G36140|AT5G41750|AT2G31880|AT2G32140|AT4G39270|AT4G113<br>36150|AT1G74360|AT5G46470|AT5G49780|AT4G11170|AT1G72940|AT1G72930
VII	30.05.01.18	transmembrane receptor protein serine/threonine kinase s	5	1340	2,0E-02	0.4%	17 out of 26623 genes, 0.1%	AT2G13790|AT4G21380|AT4G18250|AT2G48010|AT2G13800
VII	32.01	stress response	91	1340	2,0E-10	6.8%	795 out of 26623 genes, 3%	AT1G71695|AT3G61190|AT4G12720|AT3G46230|AT5G63980|AT3G08730|AT5G42650|AT4G39980|AT2G38870|AT1G27730|AT5G49620|AT4G2<br>1G54050|AT2G31180|AT2G06050|AT5G05730|AT1G42990|AT2G26650|AT1G08830|AT2G16720|AT4G02200|AT1G71697|AT5G59820|AT5G472<br>18140|AT4G34150|AT3G53260|AT1G64280|AT3G06490|AT4G02520|AT1G49570|AT1G80820|AT1G14550|AT2G47190|AT2G45170|AT1G55020<br>990|AT3G08720|AT1G14540|AT1G45145|AT2G29500|AT3G17510|AT2G24850|AT5G58720|AT4G37530|AT3G55730|AT2G02860|AT5G27760|A<br>0|AT4G08500|AT3G23250|AT4G33420|AT1G68850|AT3G12040|AT2G30490|AT2G37040|AT1G02930|AT3G45640|AT1G06180|AT1G77120|AT1<br>AT4G02380|AT3G07370|AT5G06730|AT3G21780|AT4G37520|AT4G08770|AT1G73730|AT5G20830|AT5G47910|AT1G21590
VII	32.01.01	oxidative stress response	31	1340	2,4E-06	2.3%	196 out of 26623 genes, 0.7%	AT1G71695|AT4G12720|AT5G59820|AT1G74310|AT5G05340|AT2G18140|AT3G53260|AT5G20230|AT1G49570|AT4G23190|AT1G14550|AT4G0<br>4G08770|AT1G54050|AT2G16500|AT1G14540|AT4G33420|AT1G68850|AT5G47910|AT1G45145|AT2G37040|AT1G02930|AT2G29500|AT3G456
VII	32.01.03	osmotic and salt stress response	30	1340	1,0E-05	2.2%	201 out of 26623 genes, 0.8%	AT3G55730|AT4G12720|AT5G59820|AT1G16540|AT5G54110|AT5G63980|AT3G08730|AT3G06490|AT1G27730|AT2G47190|AT3G07370|AT5G4<br>3G55270|AT3G23250|AT1G18570|AT4G01370|AT2G16500|AT3G08720|AT2G31180|AT5G20830|AT3G45640|AT2G26650|AT2G16720|AT1G061
VII	32.01.05	heat shock response	15	1340	7,1E-04	1.1%	85 out of 26623 genes, 0.3%	AT3G61190|AT4G23100|AT1G54050|AT5G59820|AT3G46230|AT4G36990|AT1G74310|AT3G08720|AT1G16540|AT1G09090|AT5G47910|AT1G6
VII	32.01.06	cold shock response	18	1340	8,3E-03	1.3%	154 out of 26623 genes, 0.6%	AT2G40140|AT3G61190|AT5G59820|AT5G47230|AT1G16540|AT5G63980|AT3G08730|AT4G34150|AT4G02520|AT1G80820|AT1G27730|AT4G0<br>5G20830|AT3G45640
VII	32.05	disease, virulence and defense	72	1340	2,9E-15	5.4%	424 out of 26623 genes, 1.6%	AT1G51660|AT3G52400|AT1G72920|AT5G42650|AT1G57650|AT1G72900|AT5G40990|AT3G23240|AT2G38870|AT5G13320|AT2G38470|AT4G1<br>5G45090|AT1G57630|AT5G44910|AT5G24810|AT5G51630|AT2G37040|AT5G44870|AT1G72910|AT3G50950|AT2G39660|AT2G34930|AT1G113<br>16540|AT1G09090|AT1G61560|AT1G80840|AT5G61900|AT4G13920|AT3G53260|AT4G36140|AT5G41750|AT3G14470|AT2G32140|AT3G05360<br>400|AT4G11340|AT3G16720|AT3G11820|AT1G55020|AT2G15220|AT5G41740|AT5G07390|AT1G15890|AT5G46520|AT3G04220|AT5G47910|A<br>0|AT1G72940|AT3G12500|AT1G72930|AT1G74710|AT2G32660|AT4G23280|AT2G44490
VII	32.07	detoxification	40	1340	1,4E-07	3%	261 out of 26623 genes, 1%	AT1G71695|AT1G69930|AT1G69880|AT2G02930|AT2G29420|AT4G36430|AT1G69920|AT1G20630|AT5G22300|AT1G27130|AT2G30870|AT2G2<br>1G68850|AT1G02930|AT1G60740|AT1G28480|AT1G08830|AT1G74590|AT1G02920|AT5G05340|AT2G18140|AT2G29440|AT4G02520|AT1G495<br>17170|AT1G14540|AT2G18150|AT1G45145|AT3G28850|AT1G12520|AT4G37530|AT2G29460
VII	32.07.07	oxygen and radical detoxification	39	1340	2,0E-07	2.9%	256 out of 26623 genes, 1%	AT1G71695|AT1G69930|AT1G69880|AT2G02930|AT2G29420|AT4G36430|AT1G69920|AT1G20630|AT1G27130|AT2G30870|AT2G29480|AT2G2<br>1G02930|AT1G60740|AT1G28480|AT1G08830|AT1G74590|AT1G02920|AT5G05340|AT2G18140|AT2G29440|AT4G02520|AT1G49570|AT1G145<br>14540|AT2G18150|AT1G45145|AT3G28850|AT1G12520|AT4G37530|AT2G29460

VII	32.07.07.03	glutathione conjugation reaction	15	1340	1,2E-05	1.1%	55 out of 26623 genes, 0.2%	AT2G29480|AT1G74590|AT1G02930|AT1G02920|AT4G02520|AT1G17170|AT2G29470|AT1G69930|AT2G02930|AT1G69920|AT2G29420|AT2G2
VII	32.07.07.05	peroxidase reaction	14	1340	3,2E-03	1%	91 out of 26623 genes, 0.3%	AT4G08770|AT1G71695|AT5G05340|AT1G14540|AT2G18150|AT2G18140|AT4G33420|AT1G68850|AT1G49570|AT4G36430|AT1G14550|AT5G0
VII	34.11	cellular sensing and response to external stimulus	135	1340	1,3E-12	10.1%	1296 out of 26623 genes, 4.9%	AT1G51660|AT5G54500|AT5G52450|AT4G12720|AT3G46230|AT3G52400|AT5G63980|AT5G42650|AT4G39980|AT3G51920|AT5G57890|AT3G2<br>4G29810|AT1G59870|AT5G54490|AT5G24810|AT2G06050|AT5G05730|AT2G39660|AT3G26830|AT2G16720|AT2G38290|AT5G55120|AT4G022<br>09090|AT2G43400|AT1G80840|AT5G61900|AT1G64280|AT2G46440|AT4G02520|AT1G80820|AT3G28210|AT2G47190|AT3G11820|AT5G37850<br>450|AT2G29420|AT5G13320|AT4G23190|AT4G11280|AT4G18250|AT2G30870|AT5G65790|AT3G23250|AT3G44880|AT1G15520|AT2G30490|A<br>0|AT5G61210|AT5G20230|AT3G07370|AT1G37130|AT2G18150|AT1G73730|AT5G20830|AT5G47910|AT2G41100|AT1G74710|AT2G44490|AT3<br>AT3G25070|AT2G44080|AT2G38470|AT3G49620|AT1G18570|AT1G54050|AT5G27420|AT2G31180|AT3G16530|AT2G26650|AT1G08830|AT2G3<br>3G06490|AT4G37790|AT1G04100|AT3G28580|AT5G45250|AT3G56400|AT1G55020|AT3G55270|AT4G01370|AT3G08720|AT2G34650|AT3G175<br>59860|AT4G08500|AT3G17700|AT4G11850|AT3G45640|AT1G11310|AT1G06180|AT5G13740|AT4G31800|AT1G25220|AT1G07400|AT4G23100<br>720|AT3G21780|AT5G45260|AT5G06320|AT1G01560|AT3G12500
VII	34.11.03	chemoperception and response	77	1340	1,0E-06	5.7%	770 out of 26623 genes, 2.9%	AT3G61190|AT5G54500|AT4G12720|AT1G63440|AT5G63980|AT3G08730|AT5G42650|AT3G51920|AT3G22060|AT1G27730|AT2G44080|AT5G4<br>5G27420|AT2G31180|AT5G24810|AT3G16530|AT5G05730|AT2G26650|AT1G08830|AT3G26830|AT2G16720|AT5G55120|AT4G02200|AT5G037<br>06490|AT4G37790|AT3G28210|AT2G47190|AT1G04100|AT3G28580|AT3G56400|AT1G55020|AT5G37850|AT3G55270|AT4G01370|AT2G16500<br>850|AT3G55730|AT2G29420|AT5G40990|AT4G21410|AT5G13320|AT4G11280|AT2G30870|AT4G08500|AT5G65790|AT3G23250|AT1G15520|A<br>0|AT1G25220|AT4G23100|AT5G27380|AT1G16540|AT1G67070|AT4G02380|AT3G07370|AT3G04720|AT3G21780|AT1G73730|AT5G20830|AT1
VII	34.11.03.13	osmosensing and response	30	1340	1,1E-05	2.2%	202 out of 26623 genes, 0.8%	AT3G55730|AT4G12720|AT5G59820|AT1G16540|AT5G54110|AT5G63980|AT3G08730|AT3G06490|AT1G27730|AT2G47190|AT3G07370|AT5G4<br>3G55270|AT3G23250|AT1G18570|AT4G01370|AT2G16500|AT3G08720|AT2G31180|AT5G20830|AT3G45640|AT2G26650|AT2G16720|AT1G061
VII	34.11.05	mechanical stimulus perception and response	3	1340	5,0E-02	0.2%	8 out of 26623 genes, 0%	AT5G61210|AT2G41100|AT4G11280
VII	34.11.09	temperature perception and response	32	1340	6,1E-05	2.4%	248 out of 26623 genes, 0.9%	AT2G40140|AT3G61190|AT4G23100|AT5G59820|AT5G47230|AT3G46230|AT1G74310|AT1G16540|AT1G09090|AT5G63980|AT3G08730|AT5G6<br>1G27730|AT1G59860|AT4G02380|AT3G07370|AT4G08500|AT4G29810|AT4G01370|AT1G54050|AT4G36990|AT3G08720|AT5G20830|AT5G479
VII	34.11.10	response to biotic stimulus	48	1340	1,4E-13	3.6%	220 out of 26623 genes, 0.8%	AT2G40140|AT1G51660|AT4G23100|AT1G71880|AT5G52450|AT1G16540|AT3G52400|AT1G61560|AT1G80840|AT4G39980|AT5G40990|AT5G5<br>1G79670|AT3G25070|AT5G13320|AT4G23190|AT3G04720|AT5G45250|AT5G45260|AT4G18250|AT2G38470|AT3G56400|AT1G59870|AT3G118<br>17700|AT4G11850|AT2G18150|AT5G06320|AT5G05730|AT2G39660|AT2G34930|AT1G11310|AT3G12500|AT1G74710|AT5G13740|AT2G38290
VII	36.20	plant / fungal specific systemic sensing and response	67	1340	2,0E-07	5%	599 out of 26623 genes, 2.2%	AT3G61190|AT5G54500|AT4G12720|AT5G42650|AT3G22060|AT2G22330|AT3G25070|AT1G27730|AT2G44080|AT5G49620|AT1G59870|AT1G1<br>3G26830|AT2G16720|AT3G18690|AT5G55120|AT5G03730|AT3G23150|AT1G80840|AT1G64280|AT3G06490|AT4G39950|AT4G37790|AT3G282<br>55020|AT4G01370|AT2G34650|AT3G17510|AT2G24850|AT3G55730|AT1G19250|AT2G29420|AT5G40990|AT4G21410|AT5G13320|AT4G23190<br>520|AT4G11850|AT1G11310|AT1G06180|AT4G31800|AT1G25220|AT4G23100|AT5G27380|AT1G61560|AT5G60410|AT3G07370|AT3G04720|A<br>0|AT1G74710|AT2G44490
VII	36.20.16	plant defense response	27	1340	3,5E-11	2%	81 out of 26623 genes, 0.3%	AT1G55020|AT4G23100|AT3G44880|AT4G01370|AT4G12720|AT3G23150|AT5G47220|AT1G61560|AT4G11850|AT1G19250|AT5G40990|AT1G6<br>4G23190|AT3G12500|AT5G60410|AT3G26830|AT1G74710|AT3G18690|AT5G45260|AT2G44490|AT5G03730|AT3G56400|AT1G59870
VII	36.20.16.01	disease resistance (R-gene) specific	4	1340	1,0E-02	0.3%	9 out of 26623 genes, 0%	AT1G19250|AT3G18690|AT5G60410|AT4G12720
VII	36.20.16.03	jasmonic acid/ethylene dependent systemic resistance	7	1340	2,5E-03	0.5%	22 out of 26623 genes, 0.1%	AT4G01370|AT5G40990|AT5G03730|AT3G23150|AT5G47220|AT3G56400|AT3G12500
VII	36.20.16.05	systemic acquired resistance	5	1340	3,0E-02	0.4%	21 out of 26623 genes, 0.1%	AT1G74710|AT4G01370|AT1G64280|AT3G56400|AT1G59870
VII	36.20.18	plant hormonal regulation	46	1340	4,5E-03	3.4%	539 out of 26623 genes, 2%	AT3G61190|AT5G54500|AT5G42650|AT3G22060|AT1G27730|AT2G44080|AT5G49620|AT1G18570|AT5G54490|AT5G27420|AT2G31180|AT5G0<br>1G80840|AT3G06490|AT4G37790|AT3G28210|AT2G47190|AT1G04100|AT3G28580|AT3G56400|AT1G55020|AT2G34650|AT3G17510|AT2G248<br>13320|AT4G11280|AT5G65790|AT3G23250|AT1G15520|AT1G06180|AT4G31800|AT1G25220|AT4G23100|AT5G27380|AT3G07370|AT3G04720
VII	36.20.18.02	ethylen response	14	1340	4,5E-03	1%	95 out of 26623 genes, 0.4%	AT3G23250|AT1G18570|AT3G55730|AT1G15520|AT2G31180|AT3G06490|AT5G05730|AT4G11280|AT2G16720|AT2G47190|AT1G06180|AT3G0
VII	36.20.18.05	abscisic acid response	19	1340	4,8E-03	1.4%	157 out of 26623 genes, 0.6%	AT3G55730|AT4G21410|AT3G22060|AT3G06490|AT1G27730|AT3G28210|AT2G47190|AT3G07370|AT3G28580|AT5G49620|AT3G21780|AT1G5<br>2G16720|AT1G06180|AT3G17510
VII	36.20.18.99	other plant signalling molecules response (jasmonic acid, sa	23	1340	5,9E-04	1.7%	172 out of 26623 genes, 0.6%	AT3G61190|AT4G23100|AT3G55730|AT5G27380|AT5G42650|AT1G80840|AT2G29420|AT5G40990|AT3G06490|AT4G11280|AT2G47190|AT3G5<br>1G15520|AT2G31180|AT2G16720|AT1G06180|AT4G31800|AT5G55120|AT2G24850
VII	36.25	animal specific systemic sensing and response	24	1340	2,0E-07	1.8%	105 out of 26623 genes, 0.4%	AT1G55020|AT3G61190|AT5G59820|AT5G42650|AT5G61900|AT2G02860|AT4G39980|AT3G53260|AT1G73080|AT1G64280|AT2G30490|AT2G3<br>1G29690|AT1G27730|AT4G11280|AT3G51660|AT1G71697|AT4G08500|AT2G24850|AT2G44770
VII	36.25.16	immune response	24	1340	9,6E-08	1.8%	99 out of 26623 genes, 0.4%	AT1G55020|AT3G61190|AT5G59820|AT5G42650|AT5G61900|AT2G02860|AT4G39980|AT3G53260|AT1G73080|AT1G64280|AT2G30490|AT2G3<br>1G29690|AT1G27730|AT4G11280|AT3G51660|AT1G71697|AT4G08500|AT2G24850|AT2G44770
VII	36.25.16.08	response to wounding	19	1340	2,5E-06	1.4%	79 out of 26623 genes, 0.3%	AT1G55020|AT3G61190|AT5G59820|AT5G42650|AT2G02860|AT4G39980|AT3G53260|AT1G64280|AT2G30490|AT2G37040|AT2G38870|AT2G0<br>1G71697|AT2G24850|AT4G08500
VII	40.10	cell death	14	1340	3,5E-03	1%	92 out of 26623 genes, 0.3%	AT3G44880|AT4G12720|AT1G61560|AT1G19250|AT5G61900|AT1G02170|AT5G47910|AT1G64280|AT1G29690|AT3G25070|AT1G11310|AT4G1
VII	40.10.02.01	anti-apoptosis	5	1340	5,0E-02	0.4%	27 out of 26623 genes, 0.1%	AT1G19250|AT5G61900|AT2G26560|AT4G12720|AT3G25070
VII	42.34	prokaryotic cell envelope structures	7	1340	5,7E-03	0.5%	27 out of 26623 genes, 0.1%	AT2G23770|AT2G43570|AT4G01700|AT5G62150|AT3G47540|AT2G43590|AT2G33580
VII	42.34.07	peptidoglycan layer or other prokaryotic cell wall	7	1340	2,1E-03	0.5%	21 out of 26623 genes, 0.1%	AT2G23770|AT2G43570|AT4G01700|AT5G62150|AT3G47540|AT2G43590|AT2G33580
VII	70.02	eukaryotic plasma membrane / membrane attached	45	1340	3,6E-03	3.4%	516 out of 26623 genes, 1.9%	AT3G52400|AT1G21230|AT3G25070|AT1G59870|AT2G01180|AT3G02740|AT2G33120|AT2G26650|AT2G39660|AT2G38290|AT2G44790|AT5G5<br>4G02520|AT3G22600|AT3G11820|AT1G07000|AT3G02600|AT5G07370|AT1G70170|AT3G17510|AT4G28085|AT2G02860|AT3G14090|AT5G409<br>46330|AT5G58430|AT1G11310|AT3G18830|AT4G09030|AT1G71880|AT1G61560|AT5G61210|AT5G20230|AT3G29400|AT1G64610|AT5G06320
VIII	1	METABOLISM	353	1286	3,4E-13	27.4%	4823 out of 26623 genes, 18.1%	AT2G27960|AT2G25530|AT3G08510|AT1G15210|AT1G20650|AT1G76540|AT1G80870|AT1G77130|AT1G79470|AT1G77520|AT4G21470|AT4G3<br>2G45910|AT2G31360|AT4G33010|AT2G01830|AT2G26640|AT2G46700|AT1G11545|AT1G11190|AT4G34350|AT5G38420|AT3G46700|AT1G324<br>02130|AT1G70710|AT4G02010|AT3G49360|AT2G26830|AT2G42690|AT5G64570|AT5G14060|AT5G05270|AT5G55910|AT2G16750|AT1G18460<br>190|AT4G29120|AT2G17820|AT1G67720|AT4G30190|AT3G43220|AT1G18870|AT2G19880|AT2G35020|AT5G16000|AT2G16430|AT4G34720|A<br>0|AT1G53090|AT2G32440|AT3G23640|AT2G43360|AT1G53000|AT4G37470|AT1G01290|AT1G55740|AT5G40870|AT2G22590|AT1G76790|AT1<br>AT3G62220|AT2G26080|AT1G11130|AT5G57330|AT4G33030|AT1G63970|AT1G64390|AT1G11840|AT2G03890|AT4G16360|AT3G25585|AT1G8<br>5G10870|AT3G01180|AT2G39930|AT1G11860|AT1G06430|AT2G36390|AT2G16890|AT4G15560|AT2G18960|AT2G05790|AT3G48750|AT4G014<br>42970|AT5G51350|AT5G47780|AT4G29890|AT1G27690|AT1G34430|AT1G79560|AT3G09270|AT1G32440|AT1G28570|AT2G42600|AT5G50375<br>490|AT2G31750|AT3G25820|AT1G31420|AT5G37600|AT3G02020|AT3G17940|AT2G20050|AT5G53140|AT4G36940|AT4G14070|AT1G24280|A<br>0|AT1G43710|AT1G66270|AT3G57030|AT4G31990|AT3G10850|AT2G29980|AT3G57050|AT5G42810|AT1G01050|AT1G50250|AT1G09100|AT2<br>AT2G29750|AT3G17310|AT2G34470|AT2G38700|AT1G15550|AT2G23950|AT1G70370|AT2G39360|AT2G36380|AT1G26640|AT4G38920|AT4G3<br>5G35630|AT1G68560|AT2G23910|AT3G07400|AT1G78040|AT1G79550|AT1G68760|AT1G50110|AT2G45790|AT2G46370|AT1G80640|AT5G012<br>25230|AT2G46930|AT5G38430|AT1G62430|AT4G18700|AT4G33770|AT1G53500|AT5G43330|AT1G30110|AT5G48010|AT1G63000|AT5G59520<br>640|AT1G53240|AT2G18570|AT2G32260|AT2G18790|AT1G20050|AT1G66280|AT2G01890|AT1G54220|AT1G78290|AT2G29390|AT1G19600|A<br>0|AT5G23580|AT4G00710|AT1G67090|AT2G32990|AT3G48190|AT3G57650|AT2G05990|AT1G05810|AT2G30200|AT1G04220|AT2G35120|AT3
VIII	2	ENERGY	35	1286	5,0E-02	2.7%	451 out of 26623 genes, 1.7%	AT4G30950|AT1G09780|AT1G78040|AT1G79550|AT4G04040|AT1G10760|AT1G24280|AT1G05570|AT1G42970|AT5G52920|AT5G43330|AT3G6<br>1G12230|AT3G10850|AT3G01390|AT1G34430|AT4G29120|AT3G01180|AT3G25530|AT2G39930|AT1G32440|AT1G21440|AT2G42600|AT4G301<br>18960|AT1G54220|AT3G22960
VIII	20	CELLULAR TRANSPORT, TRANSPORT FACILITATION AND TRA	180	1286	6,8E-07	14%	2404 out of 26623 genes, 9%	AT2G26900|AT3G53420|AT2G40300|AT5G48000|AT1G15210|AT5G42580|AT1G10130|AT4G17730|AT4G04950|AT4G27180|AT1G62510|AT2G3<br>5G24030|AT4G15680|AT4G37410|AT1G20840|AT5G27950|AT2G39890|AT3G08040|AT1G64200|AT4G29720|AT1G80500|AT3G01390|AT5G595<br>35335|AT2G38170|AT1G73640|AT1G12110|AT1G72140|AT5G10470|AT1G63940|AT4G35100|AT1G19450|AT5G49990|AT3G03180|AT5G41760<br>430|AT1G05810|AT2G16980|AT2G45960|AT4G30190|AT2G45510|AT3G54700|AT2G35060|AT4G39460|AT2G45180|AT1G72150|AT3G61430|A<br>0|AT1G14730|AT3G26290|AT1G76100|AT3G19490|AT2G47600|AT2G32300|AT2G30540|AT2G25810|AT1G30840|AT1G01620|AT2G38120|AT3<br>AT5G03280|AT5G12860|AT5G01600|AT1G72160|AT2G42850|AT2G39380|AT3G16060|AT1G80050|AT1G60660|AT5G13300|AT1G75130|AT1G2<br>1G16310|AT1G70330|AT5G65460|AT5G33280|AT3G05160|AT3G46900|AT2G18960|AT1G03680|AT3G27820|AT3G09800|AT2G24940|AT5G019<br>47160|AT1G64780|AT4G29890|AT2G44890|AT3G26520|AT2G14100|AT2G16850|AT5G58590|AT4G23400|AT3G52190|AT1G10950|AT1G77690<br>160|AT5G05000|AT5G64290|AT1G22530|AT2G16800|AT1G08560|AT5G40890|AT2G40540|AT5G37310|AT4G12520|AT3G63130|AT1G60960|A<br>0|AT4G12650|AT5G63450|AT3G24290|AT1G12090|AT2G36830|AT1G05300|AT4G35300|AT1G35620|AT4G08520|AT1G70300|AT4G39170|AT1<br>AT5G43370|AT5G58970|AT4G38920|AT1G60070|AT4G35060|AT1G12160|AT1G71090|AT2G32270|AT5G64410|AT1G47670|AT1G79990|AT1G7<br>4G12550|AT3G05165
VIII	32	CELL RESCUE, DEFENSE AND VIRULENCE	105	1286	1,1E-04	8.2%	1349 out of 26623 genes, 5.1%	AT1G27170|AT1G09780|AT4G33030|AT5G03280|AT5G01600|AT3G03250|AT3G53420|AT3G11630|AT1G05250|AT2G46370|AT1G19570|AT1G2<br>4G17340|AT2G43710|AT1G70000|AT4G15680|AT3G05880|AT1G05260|AT1G30870|AT1G03680|AT3G27820|AT3G48750|AT3G16460|AT2G441<br>09590|AT1G18330|AT3G26520|AT1G34510|AT3G44320|AT5G14680|AT1G35720|AT1G76180|AT3G09270|AT3G16350|AT4G23400|AT3G43800<br>150|AT1G12110|AT1G53240|AT3G52960|AT2G18790|AT1G53310|AT1G05240|AT5G63660|AT1G05850|AT2G04030|AT3G05630|AT1G78290|A<br>0|AT2G24040|AT1G20440|AT2G43535|AT1G66270|AT1G14980|AT2G17820|AT4G00430|AT1G27140|AT4G02600|AT2G45960|AT5G18100|AT1<br>AT2G43550|AT3G61430|AT4G35300|AT1G35620|AT1G10760|AT3G11410|AT2G41560|AT3G16450|AT2G18980|AT1G74840|AT1G17190|AT2G3<br>1G01620|AT1G10470|AT3G16110|AT2G37170|AT5G50340|AT2G28190|AT5G49230|AT5G08590
VIII	34	INTERACTION WITH THE ENVIRONMENT	127	1286	6,3E-08	9.9%	1451 out of 26623 genes, 5.5%	AT4G13520|AT1G09780|AT5G03280|AT5G12860|AT5G01600|AT3G53420|AT2G40300|AT2G23430|AT3G16060|AT1G28130|AT2G46370|AT1G1<br>2G43710|AT1G70000|AT2G32950|AT3G54720|AT5G65460|AT1G07610|AT1G20840|AT3G05880|AT1G05260|AT4G34350|AT3G48750|AT3G164<br>48800|AT1G30440|AT3G08040|AT1G64780|AT5G64330|AT2G38750|AT5G09590|AT4G15930|AT1G18330|AT3G26520|AT3G44320|AT1G35720<br>910|AT2G42600|AT2G38170|AT1G14920|AT2G34680|AT1G01470|AT4G21150|AT1G12110|AT1G53240|AT2G24150|AT1G72140|AT5G10470|A<br>0|AT2G46340|AT1G78290|AT1G04250|AT4G35100|AT5G64290|AT5G37190|AT4G37680|AT5G40890|AT1G67090|AT2G30520|AT2G24040|AT4<br>AT4G32650|AT3G48190|AT1G14980|AT2G17820|AT4G00430|AT4G30080|AT5G42810|AT2G45960|AT2G04780|AT1G20450|AT3G16470|AT1G7<br>3G61430|AT4G35300|AT1G34000|AT2G22670|AT1G10760|AT3G11410|AT1G15550|AT1G02340|AT2G41560|AT1G04240|AT2G06850|AT3G164<br>35860|AT2G38120|AT1G10470|AT1G76670|AT1G04550|AT2G37170|AT2G28190|AT4G00630|AT5G49230|AT1G72430|AT5G08590|AT5G38030
VIII	36	SYSTEMIC INTERACTION WITH THE ENVIRONMENT	49	1286	3,0E-02	3.8%	664 out of 26623 genes, 2.5%	AT4G13520|AT1G04250|AT5G03280|AT2G23430|AT4G37680|AT4G38320|AT3G57040|AT1G28130|AT1G20440|AT2G46370|AT1G19570|AT5G1<br>1G22070|AT3G16470|AT5G47370|AT2G01830|AT2G01570|AT3G05880|AT2G04160|AT2G22670|AT3G11410|AT1G15550|AT1G04240|AT2G068<br>38760|AT1G18330|AT1G35720|AT2G38120|AT1G10470|AT1G04550|AT1G76180|AT3G16350|AT2G36910|AT1G14920|AT2G34680|AT1G01470<br>550

VIII	40	CELL FATE	35	1286	4,9E-03	2.7%	378 out of 26623 genes, 1.4%	AT5G03280|AT3G45600|AT1G79320|AT2G19580|AT2G44110|AT5G19530|AT2G06850|AT1G71270|AT3G57040|AT1G75750|AT1G75840|AT1G7<br>2G21045|AT4G02600|AT2G36910|AT1G70710|AT1G62980|AT2G03680|AT5G04200|AT1G16060|AT1G05850|AT5G47370|AT2G03090|AT5G35<br>38430|AT1G11130|AT2G19760
VIII	70	SUBCELLULAR LOCALIZATION	553	1286	1,0E-02	43%	10308 out of 26623 genes, 38.7%	AT4G34980|AT3G08510|AT1G15210|AT1G80870|AT1G27180|AT1G69295|AT2G17630|AT1G32050|AT4G35020|AT2G32950|AT1G22070|AT2G3<br>4G21350|AT5G42250|AT1G78080|AT1G32470|AT1G20696|AT2G24980|AT3G03780|AT5G19770|AT1G19740|AT3G52960|AT1G44000|AT2G337<br>73470|AT4G35100|AT4G24780|AT1G01300|AT1G17370|AT3G04010|AT1G04820|AT2G01950|AT1G75780|AT4G30080|AT4G38130|AT1G56110<br>670|AT2G16430|AT3G22960|AT3G19490|AT5G19530|AT2G32300|AT5G53860|AT1G53090|AT2G43360|AT1G53000|AT5G18690|AT5G40870|A<br>0|AT2G40070|AT1G68490|AT1G72430|AT4G31460|AT2G46820|AT2G26080|AT1G26740|AT4G33030|AT3G62360|AT2G39380|AT1G63970|AT1<br>AT1G28130|AT4G17880|AT1G13560|AT1G60660|AT5G10870|AT3G22300|AT4G13850|AT1G11860|AT1G20010|AT5G65460|AT1G07610|AT1G1<br>3G09800|AT1G30510|AT5G59690|AT2G47160|AT2G42840|AT4G29890|AT5G09590|AT1G27690|AT1G34430|AT3G09270|AT1G32440|AT2G036<br>66950|AT5G37600|AT1G50010|AT5G64290|AT5G45760|AT1G18080|AT1G24280|AT5G37310|AT1G73760|AT2G27860|AT1G51140|AT5G62340<br>980|AT1G50250|AT5G55230|AT4G02600|AT1G29280|AT2G04780|ATCG00490|AT3G25660|AT1G35620|AT2G39670|AT1G15550|AT1G02340|A<br>0|AT2G19520|AT3G07360|AT5G59970|AT5G35630|AT1G68560|AT1G59990|AT3G07400|AT2G45050|AT2G29550|AT2G45790|AT2G46370|AT1<br>AT1G30610|AT2G19720|AT5G25460|AT4G30330|AT3G63170|AT2G45430|AT4G38900|AT5G27950|AT3G61650|AT1G64200|AT1G35720|AT2G2<br>5G10470|AT1G20050|AT1G21880|AT5G18590|AT3G01860|AT2G40150|AT3G01090|AT3G55010|AT5G41760|AT2G47320|AT1G19700|AT5G461<br>20450|AT2G35120|AT4G39460|AT5G02740|AT5G64670|AT3G22970|AT5G53480|AT1G23410|AT2G29670|AT3G16290|AT5G09450|AT1G54120<br>830|AT1G70990|AT3G05100|AT5G39800|AT1G29900|AT1G09780|AT5G40080|AT5G12860|AT1G04680|AT3G11490|AT3G24190|AT5G27670|A<br>0|AT1G71130|AT5G51660|AT1G23190|AT1G74210|AT1G76090|AT1G05570|AT2G04240|AT1G65960|AT1G77530|AT3G43800|AT3G25830|AT2<br>AT1G29980|AT2G43970|AT1G16340|AT4G38660|AT4G29730|AT5G57150|AT3G63130|AT1G54690|AT2G29660|AT5G17230|AT5G05760|AT3G0<br>5G06280|AT1G53280|AT1G17190|AT1G74840|AT1G75840|AT3G62680|AT5G58970|AT2G11810|AT3G10190|AT2G28190|AT5G15980|AT3G534<br>23720|AT1G72730|AT2G41380|AT5G65360|AT1G73940|AT1G77520|AT1G75390|AT4G33580|AT1G76490|AT2G18440|AT1G08200|AT3G13040<br>960|AT1G68010|AT3G46700|AT5G19780|AT2G18050|AT3G16350|AT5G11260|AT1G27400|AT1G24530|AT1G31940|AT2G04030|AT1G07300|A<br>0|AT3G07770|AT2G28070|AT3G28700|AT1G54740|AT4G29120|AT3G44100|AT5G12150|AT2G45960|AT4G30190|AT4G05590|AT2G01570|AT2<br>AT1G42960|AT1G01620|AT1G01290|AT2G38120|AT4G27230|AT1G76790|AT2G37170|AT2G29570|AT3G53730|AT2G38140|AT3G53750|AT3G1<br>1G71270|AT2G44050|AT2G34050|AT3G01180|AT1G06430|AT1G70000|AT2G36390|AT3G15000|AT1G12560|AT1G22170|AT5G60690|AT2G370<br>63120|AT1G18330|AT1G79560|AT5G42480|AT2G42590|AT2G41490|AT3G25820|AT2G46340|AT2G35605|AT5G66160|AT5G05000|AT1G04250<br>400|AT2G44640|AT1G32070|AT2G30520|AT1G55330|AT3G57030|AT1G26460|AT2G44150|AT1G14980|AT3G57050|AT1G01050|AT4G37190|A<br>0|AT2G13690|AT2G47270|AT1G27950|AT3G13860|AT4G08520|AT2G28720|AT1G04240|AT2G36380|AT1G19000|AT4G36380|AT1G29950|AT5<br>AT1G69310|AT3G49760|AT1G79550|AT2G14890|AT2G40300|AT3G11630|AT4G18610|AT1G68760|AT2G24060|AT5G41600|AT3G50685|AT2G2<br>4G28440|AT1G65010|AT5G01220|AT1G53680|AT5G58930|AT1G15810|AT2G35940|AT2G44940|AT2G01910|AT5G38430|AT2G03090|AT1G206
VIII	01.01	amino acid metabolism	30	1286	2,0E-02	2.3%	335 out of 26623 genes, 1.3%	AT5G14060|AT5G37600|AT2G24580|AT3G02020|AT1G51570|AT1G70310|AT5G19530|AT1G50110|AT5G17920|AT1G65960|AT1G32470|AT2G4<br>2G05990|AT3G03780|AT3G57050|AT3G25530|AT1G11860|AT2G17630|AT3G04940|AT2G35120|AT2G27820|AT5G35630|AT4G33010|AT1G312
VIII	01.01.09.01	metabolism of glycine	6	1286	2,0E-02	0.5%	25 out of 26623 genes, 0.1%	AT2G26080|AT2G24580|AT4G33010|AT1G11860|AT1G32470|AT2G35120
VIII	01.01.09.01.0	degradation of glycine	5	1286	4,9E-03	0.4%	10 out of 26623 genes, 0%	AT2G26080|AT1G11860|AT1G32470|AT4G33010|AT2G35120
VIII	01.02	nitrogen, sulfur and selenium metabolism	16	1286	5,0E-02	1.2%	159 out of 26623 genes, 0.6%	AT5G37600|AT3G57050|AT4G33030|AT1G11860|AT2G17630|AT3G04940|AT1G14920|AT1G08980|AT1G74090|AT1G65960|AT5G35630|AT3G0
VIII	01.02.02	nitrogen metabolism	11	1286	3,0E-02	0.9%	83 out of 26623 genes, 0.3%	AT5G37600|AT3G57050|AT1G08980|AT1G65960|AT5G35630|AT1G11860|AT2G17630|AT4G29720|AT1G29900|AT2G34470|AT3G44320
VIII	01.03.01	purin nucleotide/nucleoside/nucleobase metabolism	17	1286	3,0E-02	1.3%	159 out of 26623 genes, 0.6%	AT1G11190|AT1G79550|AT1G10760|AT1G32440|AT3G19720|AT4G35020|AT3G55010|AT5G52920|AT2G19860|AT5G48300|AT1G75840|AT1G8<br>3G22960
VIII	01.05	C-compound and carbohydrate metabolism	144	1286	3,4E-09	11.2%	1625 out of 26623 genes, 6.1%	AT1G78040|AT5G57330|AT1G79550|AT3G03250|AT2G23540|AT1G64390|AT1G11840|AT1G48600|AT2G45790|AT1G77130|AT1G77520|AT1G1<br>1G62660|AT2G39930|AT1G11860|AT5G66530|AT1G08200|AT2G46930|AT2G36390|AT2G16890|AT5G38430|AT4G33010|AT5G66680|AT1G209<br>38420|AT2G01880|AT1G23190|AT1G78570|AT1G74210|AT1G76090|AT1G05570|AT1G42970|AT5G47780|AT3G46700|AT5G17920|AT1G53500<br>980|AT1G75680|AT4G29720|AT1G27690|AT1G63000|AT1G77530|AT5G59520|AT1G34430|AT3G03780|AT1G16900|AT1G19710|AT1G32440|A<br>0|AT2G18570|AT1G53310|AT1G60810|AT2G19860|AT1G05850|AT1G47600|AT3G49360|AT1G17430|AT2G20370|AT2G41490|AT2G31750|AT1<br>AT4G04040|AT1G19600|AT1G18460|AT1G24280|AT1G14720|AT3G04010|AT1G76400|AT1G67090|AT1G51470|AT2G27860|AT1G32070|AT2G3<br>3G10850|AT4G29120|AT5G63800|ATCG00490|AT1G45130|AT2G35120|AT2G29750|AT1G54010|AT1G24170|AT3G17310|AT1G71070|AT2G164<br>70370|AT5G52920|AT5G38410|AT2G06850|AT1G08110|AT2G18560|AT5G58090|AT5G20280|AT2G32810|AT3G23640|AT1G53000|AT1G09010<br>530|AT2G22590|AT1G34130|AT2G03220|AT1G76790|AT5G66690|AT2G28470|AT4G31140|AT2G38650|AT1G68560|AT1G14080|AT2G22900|A
VIII	01.05.02	sugar, glucoside, polyol and carboxylate metabolism	87	1286	5,6E-08	6.8%	846 out of 26623 genes, 3.2%	AT1G78040|AT5G57330|AT1G79550|AT3G17940|AT3G03250|AT4G04040|AT1G19600|AT1G24280|AT3G04010|AT1G67090|AT1G64390|AT2G2<br>1G77130|AT1G66270|AT3G60750|AT1G12230|AT5G01220|AT1G64440|AT4G29120|AT3G01180|AT5G63800|AT1G62660|AT5G66530|AT1G082<br>29750|AT1G24170|AT1G71070|AT1G20950|AT2G05790|AT3G22960|AT5G38420|AT1G23190|AT1G78570|AT1G08660|AT1G05570|AT1G42970<br>560|AT5G58090|AT5G20280|AT5G13980|AT2G32810|AT3G23640|AT1G75680|AT1G63000|AT5G59520|AT1G09010|AT1G67490|AT5G51820|A<br>0|AT1G32440|AT2G03220|AT2G42600|AT1G70710|AT5G66690|AT2G28470|AT2G18570|AT1G53310|AT4G31140|AT1G60810|AT2G38650|AT2<br>AT2G20370|AT2G31750|AT1G48930|AT1G66280|AT1G54220|AT5G64570
VIII	01.05.02.07	sugar, glucoside, polyol and carboxylate catabolism	14	1286	4,9E-03	1.1%	93 out of 26623 genes, 0.3%	AT1G78040|AT1G53310|AT4G29120|AT3G25530|AT1G79550|AT4G04040|AT1G19600|AT1G32440|AT2G42600|AT1G20950|AT1G42970|AT5G5
VIII	01.05.03	polysaccharide metabolism	22	1286	3,0E-02	1.7%	231 out of 26623 genes, 0.9%	AT1G67490|AT5G51820|AT1G64440|AT3G01180|AT2G39930|AT1G10760|AT1G34130|AT2G03220|AT1G70710|AT2G36390|AT1G05570|AT1G7<br>1G68560|AT2G20370|AT2G41490|AT1G53000|AT2G01720|AT5G64570
VIII	01.05.03.04.0	starch anabolism	4	1286	5,0E-02	0.3%	13 out of 26623 genes, 0%	AT5G51820|AT2G39930|AT5G48300|AT2G36390
VIII	01.05.05	C-1 compound metabolism	23	1286	1,4E-03	1.8%	183 out of 26623 genes, 0.7%	AT4G33580|AT1G34430|AT1G54000|AT5G38420|AT1G08200|AT1G28570|AT2G42600|ATCG00490|AT2G46930|AT1G76090|AT5G38410|AT1G6<br>5G38430|AT1G54010|AT4G33010|AT1G32470|AT3G09410|AT2G26080|AT1G54220
VIII	01.05.07	C-3 compound metabolism	15	1286	1,4E-03	1.2%	89 out of 26623 genes, 0.3%	AT5G38430|AT1G53310|AT1G79550|AT5G38420|AT1G32440|AT1G74210|AT2G42600|AT1G18460|ATCG00490|AT5G52920|AT3G10850|AT5G3
VIII	01.05.12	autotrophic CO2-fixation	6	1286	6,7E-03	0.5%	18 out of 26623 genes, 0.1%	AT5G38420|AT1G42970|AT5G38430|ATCG00490|AT5G38410|AT1G67090
VIII	01.06	lipid, fatty acid and isoprenoid metabolism	68	1286	2,7E-04	5.3%	790 out of 26623 genes, 3%	AT2G29390|AT4G22756|AT4G33030|AT3G02630|AT3G57020|AT3G08510|AT1G18460|AT4G14070|AT1G07420|AT1G63970|AT2G23540|AT3G2<br>5G17230|AT3G48190|AT3G57030|AT3G57650|AT5G01220|AT2G29980|AT4G17190|AT2G05990|AT1G76490|AT2G30200|AT2G43710|AT3G515<br>54010|AT2G31360|AT2G19880|AT1G06090|AT4G15560|AT2G26640|AT4G30950|AT4G34350|AT2G07050|AT2G38040|AT1G54000|AT1G74210<br>440|AT5G48010|AT1G53000|AT2G11810|AT1G06120|AT4G36380|AT1G62640|AT2G22230|AT3G25830|AT1G28570|AT5G58560|AT2G32260|A<br>0|AT2G41490|AT5G16390|AT3G25820
VIII	01.06.02.02	glycolipid metabolism	6	1286	3,0E-03	0.5%	14 out of 26623 genes, 0.1%	AT1G53000|AT2G11810|AT4G33030|AT5G01220|AT3G05630|AT2G19880
VIII	01.06.05	fatty acid metabolism	14	1286	1,0E-02	1.1%	107 out of 26623 genes, 0.4%	AT4G30950|AT2G05990|AT2G30200|AT2G43710|AT2G38040|AT2G31360|AT1G62640|AT1G06090|AT2G22230|AT3G61200|AT3G02630|AT5G1
VIII	01.06.06	isoprenoid metabolism	23	1286	9,6E-04	1.8%	174 out of 26623 genes, 0.7%	AT4G36380|AT4G34350|AT2G29390|AT4G22756|AT2G07050|AT1G76490|AT3G25830|AT2G38700|AT3G57020|AT1G76090|AT1G15550|AT1G0<br>3G54250|AT5G48010|AT4G15560|AT5G17230|AT3G57030|AT3G25820|AT4G17190
VIII	01.06.06.11	tetracyclic and pentacyclic triterpenes (cholesterin, steroid	11	1286	4,9E-04	0.9%	41 out of 26623 genes, 0.2%	AT4G36380|AT2G29390|AT3G54250|AT4G22756|AT2G38700|AT5G50375|AT1G76090|AT2G07050|AT1G07420|AT1G20050|AT4G17190
VIII	02.01	glycolysis and gluconeogenesis	11	1286	2,0E-02	0.9%	73 out of 26623 genes, 0.3%	AT1G09780|AT1G78040|AT1G79550|AT4G04040|AT1G32440|AT1G20950|AT1G42970|AT5G52920|AT3G10850|AT1G54220|AT3G22960
VIII	10.01.11	regulation of DNA processing	3	1286	2,0E-02	0.2%	3 out of 26623 genes, 0%	AT2G29570|AT2G23430|AT1G07370
VIII	10.03.03	cytokinesis (cell division) /septum formation and hydrolysis	8	1286	2,0E-02	0.6%	42 out of 26623 genes, 0.2%	AT5G55230|AT2G30410|AT2G35190|AT3G48750|AT2G28350|AT3G61650|AT5G05620|AT4G30080
VIII	14.01	protein folding and stabilization	23	1286	3,0E-02	1.8%	247 out of 26623 genes, 0.9%	AT5G58710|AT5G56010|AT1G73655|AT2G41000|AT2G28000|AT2G18040|AT2G30410|AT5G42480|AT3G07770|AT5G56000|AT5G26360|AT3G1<br>4G04950|AT2G04030|AT5G20890|AT2G36130|AT5G09590|AT1G21750|AT1G14980
VIII	20.01	transported compounds (substrates)	136	1286	1,4E-04	10.6%	1881 out of 26623 genes, 7.1%	AT5G12860|AT5G01600|AT2G26900|AT2G40300|AT5G48000|AT1G15210|AT2G42850|AT5G42580|AT1G10130|AT1G80050|AT4G17730|AT4G0<br>1G22550|AT1G03000|AT1G22740|AT2G27000|AT1G76490|AT1G16310|AT1G70330|AT4G35020|AT5G33280|AT4G15680|AT3G05160|AT4G374<br>27820|AT3G09800|AT2G24940|AT5G01990|AT1G30510|AT1G51610|AT2G13820|AT2G39890|AT4G02050|AT2G47160|AT1G64780|AT4G29890<br>390|AT5G59520|AT1G54730|AT1G48370|AT4G35335|AT2G38170|AT1G73640|AT1G12110|AT1G72140|AT3G52190|AT1G77690|AT2G35190|A<br>0|AT1G19450|AT2G16800|AT1G08560|AT5G40890|AT2G40540|AT5G41760|AT3G24300|AT4G12520|AT1G60960|AT2G16990|AT1G22990|AT4<br>AT2G16980|AT5G05760|AT4G30190|AT2G45510|AT3G54700|AT2G35060|AT5G63450|AT3G24290|AT1G12090|AT1G05300|AT4G39460|AT2G4<br>1G30760|AT1G20860|AT1G14730|AT3G26290|AT1G76100|AT3G19490|AT1G35620|AT4G08520|AT1G70300|AT2G47600|AT4G39170|AT2G360<br>62270|AT5G43370|AT4G38920|AT1G60070|AT4G35060|AT1G30840|AT1G12160|AT1G71090|AT2G38120|AT2G32270|AT5G64410|AT3G16110<br>610|AT4G00630|AT1G48230|AT1G80310|AT1G04690|AT4G12550|AT3G05165
VIII	20.01.01	ion transport	50	1286	1,1E-05	3.9%	450 out of 26623 genes, 1.7%	AT1G20860|AT3G19490|AT1G70300|AT5G01600|AT2G26900|AT2G47600|AT2G40300|AT2G41560|AT2G16800|AT5G40890|AT1G51610|AT2G4<br>1G80050|AT3G62270|AT1G60960|AT5G43370|AT1G22990|AT4G32650|AT1G64200|AT4G38920|AT2G38940|AT3G01390|AT4G35060|AT5G595<br>30190|AT2G38170|AT1G78560|AT1G77610|AT1G12110|AT3G54700|AT4G00630|AT3G52190|AT2G35060|AT1G48230|AT5G33280|AT3G24290<br>960|AT4G34720
VIII	20.01.01.01	cation transport (H+, Na+, K+, Ca2+ , NH4+, etc.)	34	1286	3,2E-03	2.6%	351 out of 26623 genes, 1.3%	AT3G19490|AT1G70300|AT5G01600|AT2G26900|AT2G47600|AT2G40300|AT2G41560|AT2G16800|AT1G51610|AT2G40540|AT1G10130|AT3G2<br>1G64200|AT3G01390|AT4G35060|AT5G59520|AT2G32270|AT1G16310|AT4G19960|AT4G30190|AT2G38170|AT1G78560|AT4G00630|AT2G350<br>18960|AT4G34720
VIII	20.01.01.07	anion transport	15	1286	3,2E-03	1.2%	99 out of 26623 genes, 0.4%	AT3G54700|AT1G20860|AT3G52190|AT5G43350|AT1G48230|AT5G33280|AT2G47160|AT1G80050|AT3G62270|AT1G80310|AT5G43370|AT1G7
VIII	20.01.01.07.0	phosphate transport	7	1286	1,1E-03	0.5%	16 out of 26623 genes, 0.1%	AT3G54700|AT1G20860|AT3G52190|AT5G43350|AT2G38940|AT5G43370|AT1G80050
VIII	20.01.03	C-compound and carbohydrate transport	19	1286	1,1E-03	1.5%	129 out of 26623 genes, 0.5%	AT1G20860|AT5G43350|AT1G54730|AT5G64290|AT5G12860|AT2G26900|AT1G19450|AT4G35335|AT1G78560|AT3G54700|AT5G41760|AT4G0<br>2G38940|AT3G05165|AT4G35300
VIII	20.01.03.01	sugar transport	14	1286	2,0E-02	1.1%	109 out of 26623 genes, 0.4%	AT3G54700|AT1G20860|AT5G43350|AT1G54730|AT5G41760|AT4G02050|AT3G05160|AT1G20840|AT5G43370|AT1G19450|AT4G35335|AT2G3
VIII	20.03	transport facilities	56	1286	1,8E-04	4.4%	596 out of 26623 genes, 2.2%	AT5G64290|AT4G35100|AT5G03280|AT5G12860|AT1G22530|AT2G26900|AT3G53420|AT1G72160|AT5G49990|AT5G40890|AT1G10130|AT5G3<br>4G17340|AT4G00430|AT4G12650|AT2G45960|AT4G30190|AT1G32050|AT5G24030|AT5G33280|AT2G36830|AT1G72150|AT2G18960|AT3G614<br>36070|AT2G41560|AT1G51610|AT2G47160|AT3G08040|AT3G62270|AT3G26520|AT3G01390|AT2G25810|AT1G01620|AT2G16850|AT1G71090<br>110|AT2G37170|AT1G72140|AT4G00630|AT1G10950|AT1G77690|AT1G04690|AT5G38030
VIII	20.03.01	channel / pore class transport	18	1286	1,4E-03	1.4%	122 out of 26623 genes, 0.5%	AT2G25810|AT2G37180|AT1G01620|AT4G17340|AT4G00430|AT4G35100|AT5G33280|AT2G16850|AT2G36830|AT3G53420|AT1G04690|AT4G2<br>5G40890|AT2G37170
VIII	32.01	stress response	77	1286	2,3E-06	6%	795 out of 26623 genes, 3%	AT1G78290|AT1G09780|AT4G33030|AT4G35100|AT5G03280|AT5G01600|AT3G03250|AT3G53420|AT1G67090|AT3G01190|AT1G05250|AT2G2<br>5G01220|AT2G37180|AT2G17820|AT4G17340|AT4G00430|AT2G45960|AT1G70000|AT5G18100|AT1G20450|AT3G16470|AT1G73330|AT2G015<br>61430|AT4G35300|AT3G48750|AT1G10760|AT3G11410|AT3G16460|AT2G27710|AT2G41560|AT3G16450|AT2G04240|AT2G18980|AT1G74840<br>520|AT1G34510|AT3G44320|AT1G44970|AT5G14680|AT1G01620|AT1G35720|AT1G10470|AT1G76180|AT3G16350|AT4G23400|AT2G42600|A<br>0|AT1G53240|AT2G37170|AT5G50340|AT2G28190|AT5G49230|AT1G53310|AT2G18790|AT1G05240|AT1G05850|AT2G04030|AT3G05630|AT5
VIII	32.01.03	osmotic and salt stress response	31	1286	5,6E-06	2.4%	201 out of 26623 genes, 0.8%	AT1G78290|AT2G37180|AT1G01620|AT4G17340|AT2G17820|AT1G35720|AT4G35100|AT3G53420|AT3G16350|AT4G23400|AT2G45960|AT2G4<br>2G37170|AT2G04240|AT1G74840|AT1G05850|AT2G24040|AT2G01570|AT2G36830|AT2G38750|AT1G20440|AT3G05880|AT1G05260|AT5G085
VIII	32.01.06	cold shock response	21	1286	1,2E-03	1.6%	154 out of 26623 genes, 0.6%	AT1G09780|AT3G48750|AT5G01600|AT1G10760|AT3G11410|AT1G76180|AT3G16460|AT2G27710|AT2G38170|AT4G21150|AT1G53240|AT1G6<br>2G24040|AT1G20440|AT3G05880|AT1G05260|AT4G35300
VIII	32.01.11	nutrient starvation response	7	1286	6,0E-03	0.5%	25 out of 26623 genes, 0.1%	AT2G11810|AT3G05630|AT3G03250|AT1G66270|AT4G33030|AT5G01220|AT3G44320
VIII	32.07	detoxification	28	1286	2,7E-03	2.2%	261 out of 26623 genes, 1%	AT1G35620|AT4G35090|AT3G11630|AT2G18980|AT3G01190|AT1G19550|AT1G17190|AT2G30540|AT1G05250|AT1G19570|AT2G18030|AT1G3<br>3G16110|AT3G09270|AT3G43800|AT5G18100|AT3G52960|AT2G28190|AT1G05240|AT4G15680|AT1G30870|AT1G05260|AT1G03680|AT3G278
VIII	32.07.07	oxygen and radical detoxification	28	1286	2,1E-03	2.2%	256 out of 26623 genes, 1%	AT1G35620|AT4G35090|AT3G11630|AT2G18980|AT3G01190|AT1G19550|AT1G17190|AT2G30540|AT1G05250|AT1G19570|AT2G18030|AT1G3<br>3G16110|AT3G09270|AT3G43800|AT5G18100|AT3G52960|AT2G28190|AT1G05240|AT4G15680|AT1G30870|AT1G05260|AT1G03680|AT3G278
VIII	32.07.07.05	peroxidase reaction	11	1286	5,0E-02	0.9%	91 out of 26623 genes, 0.3%	AT1G44970|AT2G18980|AT3G01190|AT1G05250|AT1G05240|AT1G35720|AT4G35090|AT1G05260|AT1G30870|AT1G34510|AT3G27820
VIII	34.11	cellular sensing and response to external stimulus	115	1286	1,7E-07	8.9%	1296 out of 26623 genes, 4.9%	AT4G13520|AT1G09780|AT5G03280|AT5G12860|AT5G01600|AT3G53420|AT2G40300|AT2G23430|AT1G28130|AT2G46370|AT1G19570|AT5G1<br>2G32950|AT3G54720|AT1G07610|AT1G20840|AT3G05880|AT1G05260|AT4G34350|AT3G48750|AT3G16460|AT2G27710|AT1G42970|AT2G042<br>38750|AT5G09590|AT1G18330|AT3G26520|AT3G44320|AT1G35720|AT1G76180|AT3G16350|AT5G11260|AT4G23400|AT2G36910|AT2G42600<br>150|AT1G12110|AT1G53240|AT2G24150|AT1G72140|AT2G18790|AT1G53310|AT1G05850|AT2G04030|AT3G05630|AT2G46340|AT1G78290|A<br>0|AT5G40890|AT1G67090|AT2G30520|AT2G24040|AT4G38320|AT3G57040|AT1G75780|AT1G60960|AT1G20440|AT4G32650|AT3G48190|AT1<br>AT1G20450|AT3G16470|AT1G73330|AT5G47370|AT2G01570|AT2G36830|AT2G04160|AT3G61430|AT4G35300|AT1G34000|AT2G22670|AT1G1<br>1G04240|AT2G06850|AT3G16450|AT1G74840|AT1G75750|AT5G51820|AT1G01620|AT2G38120|AT1G10470|AT1G76670|AT1G04550|AT2G371<br>38030|AT4G12550
VIII	34.11.03	chemoperception and response	79	1286	2,0E-07	6.1%	770 out of 26623 genes, 2.9%	AT1G78290|AT4G13520|AT1G04250|AT4G35100|AT5G03280|AT5G01600|AT3G53420|AT2G40300|AT2G23430|AT4G37680|AT5G40890|AT2G2<br>2G46370|AT1G19570|AT5G13300|AT2G37180|AT2G17820|AT4G17340|AT4G00430|AT4G30080|AT2G45960|AT1G70000|AT1G20450|AT1G076<br>36830|AT3G05880|AT2G04160|AT1G05260|AT3G61430|AT4G35300|AT4G34350|AT2G22670|AT3G11410|AT1G15550|AT2G41560|AT1G42970<br>750|AT2G38750|AT1G18330|AT3G26520|AT3G44320|AT1G01620|AT1G35720|AT2G38120|AT1G10470|AT1G04550|AT1G76180|AT3G16350|A<br>0|AT2G34680|AT1G01470|AT1G12110|AT2G37170|AT2G28190|AT2G24150|AT5G49230|AT1G53310|AT1G72430|AT1G05850|AT2G04030|AT3
VIII	34.11.03.12	water response	21	1286	5,0E-05	1.6%	116 out of 26623 genes, 0.4%	AT1G78290|AT5G49230|AT2G37180|AT1G53310|AT1G01620|AT4G00430|AT1G73330|AT1G05850|AT3G53420|AT1G76180|AT3G11410|AT1G2<br>1G01470|AT1G12110|AT2G37170|AT4G35300|AT1G20450

VIII	34.11.03.13	osmosensing and response	31	1286	5,6E-06	2.4%	202 out of 26623 genes, 0.8%	AT1G78290|AT2G37180|AT1G01620|AT4G17340|AT2G17820|AT1G35720|AT4G35100|AT3G53420|AT3G16350|AT4G23400|AT2G45960|AT2G4<br>2G37170|AT2G04240|AT1G74840|AT1G05850|AT2G24040|AT2G01570|AT2G36830|AT2G38750|AT1G20440|AT3G05880|AT1G05260|AT5G085
VIII	34.11.09	temperature perception and response	26	1286	4,9E-03	2%	248 out of 26623 genes, 0.9%	AT1G09780|AT3G48750|AT5G03280|AT5G01600|AT1G10760|AT3G11410|AT1G76180|AT3G16460|AT2G27710|AT2G38170|AT4G21150|AT1G5<br>3G16470|AT1G05850|AT2G24040|AT1G20440|AT3G05880|AT2G04030|AT5G09590|AT1G05260|AT1G14980|AT4G35300
VIII	36.20	plant / fungal specific systemic sensing and response	47	1286	1,0E-02	3.7%	599 out of 26623 genes, 2.2%	AT4G13520|AT1G04250|AT5G03280|AT2G23430|AT4G37680|AT4G38320|AT3G57040|AT1G28130|AT1G20440|AT2G46370|AT1G19570|AT5G1<br>1G22070|AT3G16470|AT5G47370|AT2G01830|AT2G01570|AT3G05880|AT2G04160|AT2G22670|AT3G11410|AT1G15550|AT1G04240|AT2G068<br>18330|AT1G35720|AT2G38120|AT1G10470|AT1G04550|AT1G76180|AT3G16350|AT2G36910|AT1G14920|AT2G34680|AT2G24150|AT1G72430
VIII	36.20.18	plant hormonal regulation	44	1286	1,0E-02	3.4%	539 out of 26623 genes, 2%	AT4G13520|AT1G04250|AT5G03280|AT2G23430|AT4G37680|AT4G38320|AT3G57040|AT1G28130|AT1G20440|AT2G46370|AT1G19570|AT5G1<br>5G47370|AT2G01830|AT2G01570|AT3G05880|AT2G04160|AT2G22670|AT3G11410|AT1G15550|AT1G04240|AT2G06850|AT1G74840|AT1G757<br>10470|AT1G04550|AT1G76180|AT3G16350|AT2G36910|AT1G14920|AT2G34680|AT2G24150|AT1G72430|AT1G05850|AT3G05630|AT4G12550
VIII	36.20.18.01	auxin response	21	1286	5,0E-02	1.6%	236 out of 26623 genes, 0.9%	AT4G13520|AT2G22670|AT1G04250|AT4G30080|AT2G38120|AT1G04550|AT3G16350|AT2G36910|AT1G70000|AT2G34680|AT1G04240|AT2G0<br>2G46370|AT2G04160|AT3G05630|AT5G13300|AT4G12550
VIII	36.25.07	animal hormonal regulation	4	1286	8,0E-03	0.3%	6 out of 26623 genes, 0%	AT5G03280|AT4G38320|AT2G24150|AT4G37680
VIII	40.01	cell growth / morphogenesis	23	1286	4,9E-03	1.8%	205 out of 26623 genes, 0.8%	AT4G36380|AT2G19520|AT2G36910|AT1G70710|AT1G62980|AT5G19530|AT2G03680|AT2G06850|AT1G16060|AT1G71270|AT1G05850|AT5G4<br>1G12560|AT2G20370|AT2G28550|AT5G66680|AT4G38430|AT5G13300|AT2G19760
VIII	40.01.03	directional cell growth (morphogenesis)	17	1286	6,3E-03	1.3%	133 out of 26623 genes, 0.5%	AT4G36380|AT2G19520|AT1G62980|AT5G19530|AT2G03680|AT2G06850|AT1G71270|AT5G47370|AT2G03090|AT1G75750|AT1G75780|AT1G1<br>2G19760
VIII	42.04	cytoskeleton/structural proteins	19	1286	2,0E-03	1.5%	139 out of 26623 genes, 0.5%	AT5G56600|AT1G50010|AT5G19780|AT5G03280|AT5G19770|AT2G38120|AT1G20010|AT5G55230|AT2G29550|AT5G05620|AT2G37620|AT1G0<br>3G61650|AT4G15930|AT2G19760
VIII	42.04.05	microtubule cytoskeleton	10	1286	1,2E-03	0.8%	39 out of 26623 genes, 0.1%	AT5G55230|AT1G50010|AT2G29550|AT5G19780|AT3G61650|AT5G05620|AT3G63130|AT4G15930|AT1G20010|AT1G04820
VIII	43.02.05.03	root hair	4	1286	4,0E-02	0.3%	12 out of 26623 genes, 0%	AT1G64440|AT3G62680|AT1G05850|AT1G11130
VIII	70.01	cell wall	36	1286	2,0E-02	2.8%	428 out of 26623 genes, 1.6%	AT1G35620|AT5G45760|AT4G24780|AT1G01300|AT1G18080|AT3G62360|AT1G04680|AT5G05620|AT5G37310|AT2G06850|AT1G23720|AT2G2<br>1G02810|AT5G62340|AT2G24980|AT3G57030|AT2G02100|AT1G62980|AT4G21150|AT5G25460|AT1G05240|AT2G03090|AT5G51550|AT1G418<br>16430|AT2G01720|AT2G19760|AT2G46340
VIII	70.02	eukaryotic plasma membrane / membrane attached	46	1286	2,0E-03	3.6%	516 out of 26623 genes, 1.9%	AT4G35100|AT5G45760|AT2G14890|AT1G18080|AT3G53420|AT2G39380|AT3G04010|AT4G38660|AT3G24300|AT1G55330|AT2G29660|AT4G3<br>2G45960|AT2G04780|AT4G30190|AT4G35020|AT3G03530|AT5G56540|AT3G61430|AT1G27950|AT2G32300|AT1G05570|AT2G39890|AT1G530<br>18690|AT1G01620|AT2G38120|AT2G36910|AT2G37170|AT4G31140|AT1G21880|AT1G24530|AT1G70990|AT1G29980|AT4G38430|AT1G03870
VIII	70.03	cytoplasm	82	1286	9,6E-04	6.4%	1056 out of 26623 genes, 4%	AT1G79550|AT5G56940|AT1G79850|AT5G52510|AT1G68760|AT3G16420|AT2G45790|AT1G80050|AT1G28130|AT2G46370|AT1G77520|AT5G1<br>1G08200|AT4G35020|AT2G45910|AT2G19720|AT4G33010|AT2G19760|AT5G56600|AT1G23190|AT4G21350|AT2G27710|AT5G17920|AT2G471<br>35720|AT3G09270|AT3G43800|AT1G27400|AT2G42590|AT2G43970|AT5G37600|AT1G04250|AT1G16340|AT5G05620|AT3G55010|AT2G27860<br>370|AT2G05990|AT1G01050|AT1G64880|AT1G27140|AT3G51260|AT5G55230|AT1G09100|AT2G21790|AT1G74560|AT1G20450|AT2G35120|A<br>0|AT1G15550|AT5G19530|AT5G44200|AT1G23410|AT2G06850|AT1G17190|AT1G75840|AT5G06950|AT1G10470|AT2G19520|AT1G76790|AT3<br>AT1G29900
VIII	70.04	cytoskeleton	23	1286	1,6E-04	1.8%	151 out of 26623 genes, 0.6%	AT5G56600|AT1G50010|AT5G19780|AT5G19770|AT1G20010|AT3G51260|AT5G55230|AT2G29550|AT4G35020|AT5G27950|AT5G05620|AT2G3<br>3G16060|AT1G75780|AT3G53750|AT4G27180|AT3G61650|AT4G15930|AT2G19760
VIII	70.04.05	microtubule cytoskeleton	20	1286	6,0E-05	1.6%	108 out of 26623 genes, 0.4%	AT5G65460|AT5G10470|AT1G50010|AT5G19780|AT3G16060|AT5G19770|AT1G75780|AT1G20010|AT3G51260|AT5G55230|AT2G29550|AT4G3<br>4G15930|AT2G03680|AT2G19760|AT1G04820
VIII	70.07	endoplasmic reticulum	20	1286	2,0E-02	1.6%	193 out of 26623 genes, 0.7%	AT1G67490|AT1G76490|AT1G04250|AT5G05760|AT3G51260|AT1G76090|AT4G21150|AT1G76400|AT5G41600|AT3G52190|AT1G20050|AT2G3<br>5G66680|AT3G57650|AT1G06120|AT2G29980
VIII	70.10.03	chromosome	15	1286	4,0E-02	1.2%	140 out of 26623 genes, 0.5%	AT1G20693|AT4G27230|AT2G18050|AT1G09200|AT2G28720|AT5G50340|AT4G40030|AT5G59970|AT3G53730|AT5G59690|AT5G65360|AT1G5
