# Supplementary material for: Transcriptome dynamic of Arabidopsis roots infected with Phytophthora parasitica identifies VQ29, a gene induced during the penetration and involved in the restriction of infection
Source: PLoS One. 2017 Dec 27;12(12):e0190341. doi: 10.1371/journal.pone.0190341 (PMC5744986; doi:10.1371/journal.pone.0190341)
Supplement: S7 Table — FC, Fold Change. (PDF) [file pone.0190341.s011.pdf]

S7 Table: Microarray data for all the genes described.

FC, Fold Change.

				Genes expression signals during *A. thaliana* / *P. parasitica* interaction										Genes expression fold changes compared to NI control							
				NI		2.5-hai		6-hai		10.5-hai		30-hai		2.5-hai / Ctrl		6-hai / Ctrl		10.5-hai / Ctrl		30-hai / Ctrl	
Affy ID	AGI code	Gene description	Clusters	rep. 1	rep. 2	rep. 1	rep. 2	rep. 1	rep. 2	rep. 1	rep. 2	rep. 1	rep. 2	FC	direction	FC	direction	FC	direction	FC	direction
**Genes involved in triptophan biosynthesis**																					
253898_s_at	AT5G54810;AT4G27070	TSB1 (TRYPTOPHAN SYNTHASE BETA-SUBUNIT)];[AT4G27070, TSB2 (TRYPTOPHAN SYNTHASE BETA-SUBUNIT); tryptophan synthase]	VII (Interaction Up)	461,9	521,3	2719,2	5841,8	8773,5	8181,7	6238,3	6848,0	7257,1	5883,7	8,7	up	17,2	up	13,3	up	13,4	up
250014_at	AT5G17990	TRP1 (TRYPTOPHAN BIOSYNTHESIS 1)	VII (Interaction Up)	129,5	89,9	623,6	2386,4	2818,7	3934,4	3288,1	2994,2	2517,0	2104,7	13,7	up	30,8	up	28,6	up	21,1	up
259770_s_at	AT1G07780; AT1G29410; AT5G05590	PAI1 (PHOSPHORIBOSYLANTHRANILATE ISOMERASE 1); phosphoribosylanthranilate isomerase];[AT1G29410, PAI3 (PHOSPHORIBOSYLANTHRANILATE ISOMERASE 3); phosphoribosylanthranilate isomerase];[AT5G05590, PAI2 (PHOSPHORIBOSYLANTHRANILATE ISOMERASE 2); phosphoribosylanthranilate isomerase]		87,3	61,9	76,1	46,5	98,9	73,5	113,1	92,4	65,7	54,7								
266671_at	AT2G29690	ASA2 (anthranilate synthase 2); anthranilate synthase		23,0	26,4	20,3	14,3	23,6	20,6	20,7	17,6	20,3	19,2								
250738_at	AT5G05730	ASA1 (ANTHRANILATE SYNTHASE ALPHA SUBUNIT 1); anthranilate synthase	VII (Interaction Up)	134,8	142,9	2975,2	6246,1	10337,4	10377,7	10429,5	7903,7	11339,7	8925,1	33,2	up	74,6	up	66,0	up	73,0	up
251847_at	AT3G54640	TSA1 (TRYPTOPHAN SYNTHASE ALPHA CHAIN); tryptophan synthase	VII (Interaction Up)	314,3	280,2	2811,6	6991,7	5976,2	7534,3	5441,2	5417,0	5207,9	4407,5	16,5	up	22,7	up	18,3	up	16,2	up
247864_s_at	AT1G25155; AT5G57890; AT1G25083; AT1G24909; AT1G25220; AT1G24807	[AT1G25155, anthranilate synthase beta subunit, putative];[AT5G57890, anthranilate synthase beta subunit, putative];[AT1G25083, anthranilate synthase beta subunit, putative];[AT1G24909, anthranilate synthase beta subunit, putative];[AT1G25220, ASB1 (ANTHRANILATE SYNTHASE BETA SUBUNIT 1); anthranilate synthase];[AT1G24807, anthranilate synthase beta subunit, putative]	VII (Interaction Up)	149,0	132,8	1387,1	2248,3	2925,7	3003,4	2769,7	2188,3	2404,5	1860,0	12,9	up	21,0	up	17,6	up	15,1	up
248688_at	AT5G48220	indole-3-glycerol phosphate synthase		24,8	56,2	25,9	41,1	24,8	36,5	34,9	29,9	43,9	32,1								
249515_at	AT5G38530	tryptophan synthase beta type 2	I (penetration up)	211,8	170,1	455,0	323,0	365,9	315,7	446,2	303,1	541,4	292,3	2,0	up	1,8	up	2,0	up	2,2	up
251563_at	AT3G57880	C2 domain-containing protein		75,9	92,0	125,0	230,8	145,8	90,0	86,8	142,7	115,8	110,5								
**Genes involved in camalexin biosynthesis or related**																					
258277_at	AT3G26830	PAD3 (PHYTOALEXIN DEFICIENT 3)	VII (Interaction Up)	30,2	12,9	601,6	253,9	4659,8	2544,8	4361,9	3416,9	5739,9	4490,8	19,8	up	166,8	up	180,1	up	236,9	up
252827_at	AT4G39950	CYP79B2 (cytochrome P450, family 79, subfamily B, polypeptide 2)	VII (Interaction Up)	112,6	65,2	1333,9	3159,1	4490,0	7894,1	5010,5	6055,3	5627,4	5078,4	25,3	up	69,6	up	62,2	up	60,2	up
267567_at	AT2G30770	CYP71A13 (CYTOCHROME P450, FAMILY 71, SUBFAMILY A, POLYPEPTIDE 13)	VII (Interaction Up)	5,9	5,9	7,9	18,4	232,2	76,0	231,4	118,5	1355,1	507,7	2,2	up	26,3	up	29,9	up	159,0	up
264052_at	AT2G22330	CYP79B3 (cytochrome P450, family 79, subfamily B, polypeptide 3)	VII (Interaction Up)	63,7	59,5	505,7	548,9	2189,0	2105,4	1309,0	1843,8	2562,1	1827,3	8,6	up	34,8	up	25,6	up	35,6	up
262119_s_at	AT1G02930; AT1G02920	ATGSTF6 (EARLY RESPONSIVE TO DEHYDRATION 11); ATGSTF7 (GLUTATHIONE S-TRANSFERASE 11)	VII (Interaction Up)	915,3	2082,9	11896,4	15318,2	13629,4	14618,4	15545,8	14512,5	14858,5	15132,3	9,1	up	9,4	up	10,0	up	10,0	up
249078_at	AT5G44070	CAD1 (CADMIUM SENSITIVE 1)	VII (Interaction Up)	208,9	159,1	1915,2	800,3	720,0	850,8	680,9	616,0	356,8	443,2	7,4	up	4,3	up	3,5	up	2,2	up
**Expansin genes**																					
, 251791_at	AT3G55500	ATEXPA16 (ARABIDOPSIS THALIANA EXPANSIN A16)		4,6	4,6	4,6	4,6	4,6	6,8	4,6	5,0	4,6	4,6								
266770_at	AT2G03090	ATEXPA15 (ARABIDOPSIS THALIANA EXPANSIN A15)	VIII (Interaction Down)	231,3	55,0	46,5	33,9	17,3	16,3	22,5	17,3	20,2	23,5	3,6	down	8,5	down	7,2	down	6,6	down
258003_at	AT3G29030	ATEXPA5 (ARABIDOPSIS THALIANA EXPANSIN A5)		4,8	4,6	4,6	4,6	4,6	4,6	4,6	4,6	5,5	4,6								
249500_at	AT5G39260	ATEXPA21 (ARABIDOPSIS THALIANA EXPANSIN A21)		4,8	4,8	4,8	8,2	4,8	4,8	4,8	4,8	4,8	4,8								
249501_s_at	AT5G39270, AT5G39290	[AT5G39270, ATEXPA22 (ARABIDOPSIS THALIANA EXPANSIN A22)];[AT5G39290, ATEXP26 (ARABIDOPSIS THALIANA EXPANSIN A26)]		4,6	4,6	4,6	6,8	4,6	4,7	4,6	4,6	4,6	4,6								
256299_at	AT1G69530	ATEXPA1 (ARABIDOPSIS THALIANA EXPANSIN A1)		292,4	83,5	325,5	108,2	587,4	207,5	455,4	175,9	480,0	501,2								
252557_at	AT3G45960	ATEXLA3 (ARABIDOPSIS THALIANA EXPANSIN-LIKE A3)		19,1	11,3	19,3	19,0	12,8	23,2	22,3	31,2	19,3	77,8								
252563_at	AT3G45970	ATEXLA1 (ARABIDOPSIS THALIANA EXPANSIN-LIKE A1)		238,0	267,4	123,9	226,1	56,2	227,8	79,4	194,1	57,1	199,6								
247991_at	AT5G56320	ATEXPA14 (ARABIDOPSIS THALIANA EXPANSIN A14)		5,9	5,5	4,6	4,6	4,6	4,6	4,6	4,6	4,6	4,6								
250992_at	AT5G02260	ATEXPA9 (ARABIDOPSIS THALIANA EXPANSIN A9)		127,8	63,4	71,4	27,7	20,6	17,8	33,9	18,7	33,5	9,1								
266134_s_at	AT2G45110, AT1G65681	[AT2G45110, ATEXPB4 (ARABIDOPSIS THALIANA EXPANSIN B4)];[AT1G65681, similar to ATEXPB4 (ARABIDOPSIS THALIANA EXPANSIN B4)		4,6	4,6	4,6	4,6	4,6	5,3	4,6	5,9	4,6	4,6								
255591_at	AT4G01630	ATEXPA17 (ARABIDOPSIS THALIANA EXPANSIN A17)		5,2	7,1	5,5	6,5	5,1	5,4	5,4	5,4	5,4	5,4								
245463_at	AT4G17030	ATEXLB1 (ARABIDOPSIS THALIANA EXPANSIN-LIKE B1)		706,7	306,4	885,3	293,1	1066,1	681,0	3453,6	1002,5	2247,1	1367,5								
253008_at	AT4G38210	ATEXPA20 (ARABIDOPSIS THALIANA EXPANSIN A20)		8,5	5,2	6,8	5,2	5,2	5,2	5,2	4,6	5,2	5,2								
265443_at	AT2G20750	ATEXPB1 (ARABIDOPSIS THALIANA EXPANSIN B1)		13,6	6,3	4,9	5,0	4,9	4,8	4,9	4,9	4,8	4,9								
253815_at	AT4G28250	ATEXPB3 (ARABIDOPSIS THALIANA EXPANSIN B3)		911,0	184,1	383,4	75,5	51,8	85,1	24,2	23,6	31,4	69,6								
261266_at	AT1G26770	ATEXPA10 (ARABIDOPSIS THALIANA EXPANSIN A10)		10,0	7,3	14,3	10,8	30,4	10,4	13,6	10,3	10,3	19,1								
251397_at	AT3G60570	ATEXPB5 (ARABIDOPSIS THALIANA EXPANSIN B5)		4,6	4,6	4,6	4,6	4,6	4,6	4,6	6,5	4,6	4,6								
267590_at	AT2G39700	ATEXPA4 (ARABIDOPSIS THALIANA EXPANSIN A4)	VIII (Interaction Down)	1226,6	319,9	443,9	186,5	69,5	85,8	62,3	21,6	64,4	123,7	2,5	down	10,0	down	18,4	down	8,2	down
252997_at	AT4G38400	ATEXLA2 (ARABIDOPSIS THALIANA EXPANSIN-LIKE A2)		16,0	4,6	4,6	4,6	4,6	4,6	4,6	4,6	4,6	4,6								
249502_s_at	AT5G39280, AT5G39300	[AT5G39280, ATEXPA23 (ARABIDOPSIS THALIANA EXPANSIN A23)];[AT5G39300, ATEXPA25 (ARABIDOPSIS THALIANA EXPANSIN A25)]		4,6	4,6	4,9	4,6	4,6	4,6	4,6	4,6	4,6	4,6								
264640_at	AT1G65680	ATEXPB2 (ARABIDOPSIS THALIANA EXPANSIN B2)		5,9	4,6	5,0	5,0	4,6	5,3	4,8	8,0	5,0	6,3								
259525_at	AT1G12560	ATEXPA7 (ARABIDOPSIS THALIANA EXPANSIN A7)	VIII (Interaction Down)	168,0	67,7	84,3	58,9	23,8	17,7	81,0	15,3	11,9	11,5	1,6	down	5,7	down	2,4	down	10,1	down
258388_at	AT3G15370	ATEXPA12 (ARABIDOPSIS THALIANA EXPANSIN 12)		4,6	4,9	5,9	4,9	4,9	4,9	4,9	4,9	5,3	4,9								
261099_at	AT1G62980	ATEXPA18 (ARABIDOPSIS THALIANA EXPANSIN A18)	VIII (Interaction Down)	235,1	51,2	87,7	46,8	13,6	9,4	41,6	7,4	6,1	7,5	2,1	down	12,4	down	5,8	down	21,0	down
249503_at	AT5G39310	ATEXPA24 (ARABIDOPSIS THALIANA EXPANSIN A24)		6,0	6,0	6,0	6,3	6,0	6,0	6,0	9,4	6,0	6,0								
266790_at	AT2G28950	ATEXPA6 (ARABIDOPSIS THALIANA EXPANSIN A6)		43,5	14,5	10,0	6,4	5,1	4,8	6,4	5,1	6,2	6,3								
250780_at	AT5G05290	ATEXPA2 (ARABIDOPSIS THALIANA EXPANSIN A2)		4,6	4,6	4,6	4,6	4,6	4,6	4,6	4,6	4,6	4,6								
255822_at	AT2G40610	ATEXPA8 (ARABIDOPSIS THALIANA EXPANSIN A8)		12,0	4,6	4,6	4,6	4,6	4,6	4,6	4,6	4,6	4,6								
267158_at	AT2G37640	ATEXPA3 (ARABIDOPSIS THALIANA EXPANSIN A3)		9,8	6,0	4,6	4,7	5,2	4,6	4,6	4,6	4,6	4,6								
261226_at	AT1G20190	ATEXPA11 (ARABIDOPSIS THALIANA EXPANSIN A11)		6,1	5,5	4,6	4,6	4,6	4,6	4,6	4,6	4,6	4,6								
**WRKY genes**																					
263626_at	AT2G04880	ZAP1 (WRKY FAMILY TRANSCRIPTION FACTOR 1); transcription activator/ transcription factor		296,5	278,2	199,4	248,4	226,8	226,8	282,9	363,5	424,1	374,2								
260337_at	AT1G69310	WRKY57 (WRKY DNA-binding protein 57); transcription factor	VIII (Interaction Down)	155,2	173,3	40,9	121,5	34,6	66,7	55,7	60,9	78,9	74,4	2,0	down	3,2	down	2,8	down	2,1	down
253485_at	AT4G31800	WRKY18 (WRKY DNA-binding protein 18); transcription factor	VII (Interaction Up)	18,4	63,5	327,3	178,3	216,1	249,2	295,8	207,3	330,6	309,4	6,2	up	5,7	up	6,1	up	7,8	up
264538_at	AT1G55600	WRKY10 (MINISEED3); transcription factor		4,6	4,6	4,6	4,6	4,6	4,6	4,6	4,6	4,6	4,6								
260278_at	AT1G80590	WRKY66 (WRKY DNA-binding protein 66); transcription factor		4,6	4,6	6,0	6,2	9,2	5,9	25,8	5,9	23,9	11,0								
260403_at	AT1G69810	WRKY36 (WRKY DNA-binding protein 36); transcription factor	VII (Interaction Up)	72,9	86,1	281,4	212,1	253,8	261,7	264,8	231,9	189,2	131,2	3,1	up	3,2	up	3,1	up	2,0	up
254159_at	AT4G24240	WRKY7 (WRKY DNA-binding protein 7); transcription factor		105,5	199,4	147,6	125,7	111,2	112,2	62,2	99,6	60,7	79,7								
265954_at	AT2G37260	TTG2 (TRANSPARENT TESTA GLABRA 2); transcription factor		4,6	4,6	4,6	4,6	4,6	4,6	4,6	4,6	4,6	4,6								
255999_at	AT1G29860	WRKY71 (WRKY DNA-binding protein 71); transcription factor		8,3	7,9	8,3	20,0	8,1	13,9	9,3	11,1	10,7	37,6								
266886_at	AT2G44745	WRKY family transcription factor		4,6	4,6	4,6	4,7	4,6	4,7	4,6	4,6	4,6	4,6								
262613_at	AT1G13960	WRKY4 (WRKY DNA-binding protein 4); transcription factor		196,4	382,9	112,8	224,4	200,8	208,6	217,8	291,3	208,5	297,2								
255337_at	AT4G04450	WRKY42 (WRKY DNA-binding protein 42); transcription factor		4,6	4,6	4,6	4,6	5,5	4,6	4,6	4,6	4,6	4,9								
251705_at	AT3G56400	WRKY70 (WRKY DNA-binding protein 70); transcription factor	VII (Interaction Up)	9,5	14,8	989,9	145,7	975,2	334,9	1083,3	347,4	2092,4	1150,6	46,8	up	54,0	up	58,9	up	133,6	up
258975_at	AT3G01970	WRKY45 (WRKY DNA-binding protein 45); transcription factor	VII (Interaction Up)	179,9	529,8	728,5	1303,7	2456,5	2051,3	1709,3	2167,9	2150,6	2151,1	2,9	up	6,4	up	5,5	up	6,1	up
259320_at	AT3G01080	WRKY58 (WRKY DNA-binding protein 58); transcription factor		4,6	4,6	4,6	4,6	4,6	4,6	4,6	4,6	4,6	4,6								
267246_at	AT2G30250	WRKY25 (WRKY DNA-binding protein 25); transcription factor		135,0	394,4	392,6	481,0	350,8	498,2	293,0	622,7	422,1	550,6								
254652_at	AT4G18170	WRKY28 (WRKY DNA-binding protein 28); transcription factor		7,6	26,2	50,4	62,9	33,0	111,3	108,2	195,9	104,1	332,1								
265718_at	AT2G03340	WRKY3 (WRKY DNA-binding protein 3); transcription factor	II (2,5 down)	89,1	107,4	45,1	51,1	75,4	77,2	70,8	79,0	115,5	108,1	2,0	down	1,3	down	1,3	down	1,1	up
246104_at	AT5G28650	WRKY74 (WRKY DNA-binding protein 74); transcription factor		45,8	89,1	66,4	101,6	33,9	80,7	56,9	87,0	52,5	43,8								
254231_at	AT4G23810	WRKY53 (WRKY DNA-binding protein 53); DNA binding / protein binding / transcription activator/ transcription factor	VII (Interaction Up)	11,8	10,3	1004,4	311,8	220,6	312,7	163,0	233,7	177,5	444,4	59,5	up	24,1	up	17,9	up	28,1	up
251553_at	AT3G58710	WRKY69 (WRKY DNA-binding protein 69); transcription factor		309,6	225,1	321,0	209,6	321,3	242,1	342,5	249,4	242,4	179,1								
257382_at	AT2G40750	WRKY54 (WRKY DNA-binding protein 54); transcription factor	VII (Interaction Up)	53,0	11,3	959,8	306,7	509,8	149,3	558,3	54,3	367,8	321,8	19,7	up	10,2	up	9,5	up	10,7	up
267469_at	AT2G30590	WRKY21 (WRKY DNA-binding protein 21); transcription factor		57,7	56,3	79,2	70,6	65,8	56,0	44,6	83,1	73,1	72,4								
245051_at	AT2G23320	WRKY15 (WRKY DNA-binding protein 15); transcription factor	VII (Interaction Up)	95,4	112,5	556,2	506,4	1034,4	1034,5	787,5	1334,9	678,9	1220,3	5,1	up	10,0	up	10,2	up	9,1	up
262339_at	AT1G64000	WRKY56 (WRKY DNA-binding protein 56); transcription factor		5,8	5,0	5,7	4,9	5,0	5,0	5,0	5,0	4,9	5,0								
254347_at	AT4G22070	WRKY31 (WRKY DNA-binding protein 31); transcription factor	I (penetration up)	52,1	69,5	159,2	133,9	105,3	118,7	83,2	79,3	66,0	47,4	2,4	up	1,8	up	1,3	up	1,1	down
252891_at	AT4G39410	WRKY13 (WRKY DNA-binding protein 13); transcription factor		7,5	6,4	6,4	9,4	6,4	7,9	6,4	7,1	6,4	6,0								
256364_at	AT1G66550	WRKY67 (WRKY DNA-binding protein 67); transcription factor		5,3	5,0	5,3	6,8	4,8	5,3	5,0	5,9	5,8	8,0								
267427_at	AT2G34830	WRKY35 (WRKY DNA-BINDING PROTEIN 35); transcription factor		20,8	14,6	19,3	5,8	13,6	7,9	22,5	11,1	11,6	11,6								
253603_at	AT4G30935	WRKY32 (WRKY DNA-binding protein 32); transcription factor		53,7	73,5	41,4	64,2	48,9	83,2	70,6	87,2	90,8	71,2								
260528_at	AT2G47260	WRKY23 (WRKY DNA-binding protein 23); transcription factor	VIII (Interaction Down)	122,9	243,0	76,7	112,3	56,3	82,7	83,8	82,9	63,3	84,8	1,9	down	2,6	down	2,2	down	2,5	down
255596_at	AT4G01720	WRKY47 (WRKY DNA-binding protein 47); transcription factor		74,2	70,5	69,7	73,7	57,0	66,1	74,7	80,4	38,2	37,7								
264746_at	AT1G62300	WRKY6 (WRKY DNA-binding protein 6); transcription factor	VII (Interaction Up)	96,3	175,6	552,1	301,8	681,0	724,1	672,0	978,1	634,3	660,7	3,1	up	5,2	up	6,1	up	4,8	up
260882_at	AT1G29280	WRKY65 (WRKY DNA-binding protein 65); transcription factor	VIII (Interaction Down)	1090,1	1942,3	1022,5	776,4	765,7	581,8	960,2	795,7	459,5	531,0	1,7	down	2,3	down	1,7	down	3,1	down
263225_at	AT1G30650	WRKY14 (WRKY DNA-binding protein 14); transcription factor	VIII (Interaction Down)	96,3	249,5	81,1	52,3	71,6	72,7	80,8	92,7	47,3	94,1	2,6	down	2,4	down	2,0	down	2,4	down
260432_at	AT1G68150	WRKY9 (WRKY DNA-binding protein 9); transcription factor		90,4	201,9	193,4	250,4	114,0	350,6	107,3	292,5	43,3	137,9								
261429_at	AT1G18860	WRKY61 (WRKY DNA-binding protein 61); transcription factor	VII (Interaction Up)	39,5	105,2	121,0	174,0	128,6	220,8	174,8	268,9	132,6	163,1	2,0	up	2,4	up	3,1	up	2,0	up
263536_at	AT2G25000	WRKY60 (WRKY DNA-BINDING PROTEIN 60); transcription factor		10,4	4,9	5,8	4,9	4,6	4,9	5,3	4,8	6,0	4,9								
249890_at	AT5G22570	WRKY38 (WRKY DNA-binding protein 38); transcription factor	VII (Interaction Up)	6,9	6,6	249,1	13,1	372,7	24,7	259,4	7,7	183,7	88,9	19,5	up	29,6	up	19,9	up	20,3	up
245976_at	AT5G13080	WRKY75 (WRKY DNA-BINDING PROTEIN 75); transcription factor	VII (Interaction Up)	232,0	836,0	2052,8	2339,7	4844,1	4214,5	3882,6	4107,3	5211,0	4597,0	4,1	up	8,5	up	7,5	up	9,2	up
255568_at	AT4G01250	WRKY22 (WRKY DNA-binding protein 22); transcription factor	VII (Interaction Up)	11,4	37,1	431,1	632,5	216,8	283,8	75,7	429,4	66,0	224,1	22,0	up	10,3	up	10,4	up	6,0	up
253535_at	AT4G31550	WRKY11 (WRKY DNA-binding protein 11); transcription factor	I (penetration up)	156,9	141,2	648,9	646,2	260,9	315,3	148,8	221,0	76,9	128,2	4,3	up	1,9	up	1,2	up	1,5	down
254852_at	AT4G12020	WRKY19 (WRKY DNA-binding protein 19)		63,2	77,8	82,0	76,2	65,7	66,8	67,6	56,3	84,4	72,8								
249770_at	AT5G24110	WRKY30 (WRKY DNA-binding protein 30); transcription factor		5,9	5,3	40,4	9,1	17,1	34,3	22,2	24,4	12,3	42,2								
261892_at	AT1G80840	WRKY40 (WRKY DNA-binding protein 40); transcription factor	VII (Interaction Up)	22,0	25,1	2360,1	1591,7	2770,8	1642,1	1580,1	1374,0	700,0	1185,9	83,9	up	93,7	up	62,8	up	40,1	up
248306_at	AT5G52830	WRKY27 (WRKY DNA-binding protein 27); transcription factor	I (penetration up)	33,2	46,9	179,3	133,3	57,8	60,3	54,7	42,3	26,7	36,3	3,9	up	1,5	up	1,2	up	1,3	down
258803_at	AT3G04670	WRKY39 (WRKY DNA-binding protein 39); transcription factor	V (Switch up)	290,3	444,5	338,9	377,4	572,9	448,7	547,3	855,7	899,3	649,4	1,0	down	1,4	up	1,9	up	2,1	up
253983_at	AT4G26640	WRKY20 (WRKY DNA-binding protein 20); transcription factor		191,7	173,1	219,6	166,5	141,6	102,1	197,9	128,0	319,4	200,3								
266597_at	AT2G46130	WRKY43 (WRKY DNA-binding protein 43); transcription factor		8,6	4,6	8,6	12,4	8,4	10,3	8,5	15,6	9,7	8,6								
248896_at	AT5G46350	WRKY8 (WRKY DNA-binding protein 8); transcription factor		4,9	5,1	4,9	7,5	5,7	19,6	8,3	18,6	29,3	45,9								
248973_at	AT5G45050	TTR1 (WRKY domain family protein 16); transcription factor		53,9	61,2	54,3	62,8	55,8	61,5	59,0	62,0	75,8	82,8								
267028_at	AT2G38470	WRKY33 (WRKY DNA-binding protein 33); transcription factor	VII (Interaction Up)	143,1	246,8	4005,7	3113,0	2752,8	3915,8	2304,8	3545,7	1781,2	2089,9	18,3	up	17,1	up	15,0	up	9,9	up
263797_at	AT2G24570	WRKY17 (WRKY DNA-binding protein 17); transcription factor		160,2	126,8	215,6	201,1	175,4	141,1	174,9	205,5	101,1	210,5								
266052_at	AT2G40740	WRKY55 (WRKY DNA-binding protein 55); transcription factor	III (biotrophy up)	9,1	7,8	40,8	103,7	79,8	211,1	68,9	234,2	40,2	107,8	8,6	up	17,2	up	17,9	up	8,8	up
248008_at	AT5G56270	WRKY2 (WRKY DNA-binding protein 2); transcription factor	V (Switch up)	15,2	31,6	16,5	27,9	16,7	31,6	29,0	43,6	45,0	64,7	1,1	down	1,0	up	1,6	up	2,3	up
250666_at	AT5G07100	WRKY26 (WRKY DNA-binding protein 26); transcription factor	VII (Interaction Up)	94,0	304,6	288,6	382,5	401,3	482,8	451,4	669,4	308,9	549,1	1,7	up	2,2	up	2,8	up	2,2	up
250153_at	AT5G15130	WRKY72 (WRKY DNA-binding protein 72); transcription factor	I (penetration up)	81,8	111,4	1931,7	1583,3	450,9	671,6	463,8	357,3	168,2	159,3	18,2	up	5,8	up	4,3	up	1,7	up
253961_at	AT4G26440	WRKY34 (MICROSPORE-SPECIFIC PROMOTER 3); transcription factor		4,6	4,6	4,6	4,6	4,6	4,6	4,6	4,6	4,6	4,6								
263783_at	AT2G46400	WRKY46 (WRKY DNA-binding protein 46); transcription factor	VII (Interaction Up)	6,2	5,3	204,9	22,0	198,5	126,5	168,6	162,5	230,5	142,5	19,6	up	28,1	up	28,6	up	32,2	up
248611_at	AT5G49520	WRKY48 (WRKY DNA-binding protein 48); transcription factor	VII (Interaction Up)	31,7	72,2	82,5	137,9	58,1	128,1	82,1	151,2	123,3	204,7	2,1	up	1,8	up	2,2	up	3,2	up

No probe corresponded to : AT1G66560; AT1G66600; AT2G21900; AT3G62340; AT4G11070; AT4G23550; AT5G01900; AT5G26170; AT5G41570; AT5G43290; AT5G45270; AT5G64810

Genes transiently modulated during penetration and involved in perception of stimuli and in resulting responses (Cluster I and FunCatDB 32.09 and 34.11 from Table S6)

258566_at	AT3G04110	GLR1 (GLUTAMATE RECEPTOR 1)	I (2,5up)	28,8	20,5	57,1	57,0	30,0	49,9	35,2	32,5	10,2	16,9	2,3	up	1,6	up	1,4	up	1,8	down
253377_at	AT4G33300	ADR1-L1 (ADR1-LIKE 1)	I (2,5up)	657,6	735,1	1681,5	1140,7	919,3	1305,5	1254,1	1251,1	1630,6	1342,9	2,0	up	1,6	up	1,8	up	2,1	up
247326_at	AT5G64110	peroxidase, putative	I (2,5up)	22,2	14,9	659,6	148,0	113,3	148,8	218,7	35,8	37,5	31,6	21,8	up	7,1	up	6,9	up	1,9	up
253993_at	AT4G26070	MEK1 (mitogen-activated protein kinase kinase 1)	I (2,5up)	62,9	46,8	147,3	103,1	73,6	90,9	89,9	86,3	127,2	177,5	2,3	up	1,5	up	1,6	up	2,8	up
245487_at	AT4G16250	PHYD (PHYTOCHROME DEFECTIVE D)	I (2,5up)	99,8	144,7	264,4	258,2	219,6	258,2	162,6	213,2	121,1	151,9	2,1	up	2,0	up	1,5	up	1,1	up
262525_at	AT1G17060	CYP72C1 (cytochrome P450, family 72, subfamily C, polypeptide 1)	I (2,5up)	42,6	18,5	80,8	27,0	91,6	33,1	38,5	16,8	11,7	12,7	1,8	up	2,0	up	1,1	down	2,5	down
264042_at	AT2G03760	ST (steroid sulfotransferase)	I (2,5up)	137,4	20,9	287,9	82,6	221,4	66,3	86,3	40,8	29,6	41,2	2,3	up	1,8	up	1,2	down	2,2	down
256245_at	AT3G12580	HSP70 (heat shock protein 70)	I (2,5up)	1394,3	1180,0	1775,2	6734,3	914,2	812,9	180,7	536,4	2206,1	886,8	3,3	up	1,5	down	3,6	down	1,2	up
259937_s_at	AT3G13080; AT1G71330	ATMRP3 (Arabidopsis thaliana multidrug resistance-associated protein 3); ATNAP5 (Arabidopsis thaliana non-intrinsic ABC protein 5)	I (2,5up)	59,0	71,0	147,9	194,4	96,7	135,8	107,8	87,3	184,5	108,8	2,6	up	1,8	up	1,5	up	2,3	up
248978_at	AT5G45070	ATPP2-A8 (Phloem protein 2-A8)	I (2,5up)	478,6	291,6	2784,0	1733,8	272,9	407,2	467,5	355,9	326,8	241,7	5,9	up	1,1	down	1,1	up	1,4	down
254586_at	AT4G19510	disease resistance protein (TIR-NBS-LRR class), putative	I (2,5up)	24,6	35,5	50,2	60,4	58,0	67,9	34,6	55,3	38,5	35,6	1,8	up	2,1	up	1,5	up	1,2	up
248607_at	AT5G49480	ATCP1 (CA2+-BINDING PROTEIN 1)	I (2,5up)	784,2	954,2	1979,2	2765,1	1059,3	1128,2	1003,5	853,9	1110,9	832,1	2,7	up	1,3	up	1,1	up	1,1	up
266992_at	AT2G39200	MLO12 (MILDEW RESISTANCE LOCUS O 12)	I (2,5up)	119,3	180,8	632,1	730,7	65,6	178,3	110,0	106,9	20,0	60,3	4,5	up	1,2	down	1,4	down	3,7	down
267064_at	AT2G41110	CAM2 (CALMODULIN-2)	I (2,5up)	855,0	533,8	1311,9	1693,9	1252,9	1176,9	1068,7	968,8	644,9	985,0	2,2	up	1,7	up	1,5	up	1,2	up
254384_at	AT4G21870	26.5 kDa class P-related heat shock protein (HSP26.5-P)	I (2,5up)	18,0	15,3	1206,0	129,6	56,4	50,7	99,9	37,0	29,2	16,7	40,2	up	3,2	up	4,1	up	1,4	up
245010_at	ATCG00420	NADH dehydrogenase subunit J	I (2,5up)	20,8	11,6	225,3	427,2	30,5	188,5	46,5	134,4	11,9	32,0	20,1	up	6,8	up	5,6	up	1,4	up
259466_at	AT1G19050	ARR7 (RESPONSE REGULATOR 7)	I (2,5up)	93,9	58,9	157,9	237,5	64,6	72,7	41,2	52,3	33,6	35,2	2,6	up	1,1	down	1,6	down	2,2	down
265668_at	AT2G32020	GCN5-related N-acetyltransferase (GNAT) family protein	I (2,5up)	101,8	15,9	1322,2	414,8	510,5	416,3	485,7	293,0	236,6	171,4	14,8	up	7,9	up	6,6	up	3,5	up
253535_at	AT4G31550	WRKY11 (WRKY DNA-binding protein 11)	I (2,5up)	156,9	141,2	648,9	646,2	260,9	315,3	148,8	221,0	76,9	128,2	4,3	up	1,9	up	1,2	up	1,5	down
256417_s_at	AT3G11170; AT5G05580	FAD7 (FATTY ACID DESATURASE 7);FAD8 (FATTY ACID DESATURASE 8)	I (2,5up)	78,4	83,4	273,8	196,5	216,4	207,3	139,8	171,1	97,3	97,4	2,9	up	2,6	up	1,9	up	1,2	up
255059_at	AT4G09420	disease resistance protein (TIR-NBS class), putative	I (2,5up)	23,2	50,7	104,4	68,7	97,6	88,9	55,6	71,3	8,5	14,7	2,3	up	2,5	up	1,7	up	3,2	down
266461_at	AT2G47730	ATGSTF8 (GLUTATHIONE S-TRANSFERASE 8)	I (2,5up)	3357,3	1810,4	7162,1	6673,1	3966,1	3209,7	2804,7	2368,4	1824,7	1764,8	2,7	up	1,4	up	1,0	up	1,4	down
267436_at	AT2G19190	FRK1 (FLG22-INDUCED RECEPTOR-LIKE KINASE 1)	I (2,5up)	6,1	4,6	20,8	80,2	4,9	37,8	9,6	13,2	8,4	42,0	9,4	up	4,0	up	2,1	up	4,7	up
266719_at	AT2G46830	CCA1 (CIRCADIAN CLOCK ASSOCIATED 1)	I (2,5up)	106,9	23,1	66,7	214,3	11,5	9,4	6,4	7,7	25,6	10,6	2,2	up	6,2	down	9,2	down	3,6	down
266841_at	AT2G26150	ATHSFA2 (Arabidopsis thaliana heat shock transcription factor A2)	I (2,5up)	13,3	26,4	40,6	113,9	36,8	30,5	13,3	22,6	76,2	32,2	3,9	up	1,7	up	1,1	down	2,7	up
257479_at	AT1G16150	WAKL4 (WALL ASSOCIATED KINASE-LIKE 4)	I (2,5up)	10,2	18,0	96,6	77,4	9,8	35,8	14,9	47,6	11,2	22,3	6,2	up	1,6	up	2,2	up	1,2	up
267069_at	AT2G41010	ATCAMBP25 (ARABIDOPSIS THALIANA CALMODULIN (CAM)-BINDING PROTEIN OF 25 KDA)	I (2,5up)	48,9	147,5	356,1	290,8	190,2	198,8	106,3	130,1	62,1	174,1	3,3	up	2,0	up	1,2	up	1,2	up
262703_at	AT1G16510	auxin-responsive family protein	I (2,5up)	25,9	26,4	90,1	142,1	58,6	47,2	37,1	29,7	35,4	38,5	4,4	up	2,0	up	1,3	up	1,4	up
245457_s_at	AT4G16940; AT4G16960	disease resistance protein (TIR-NBS-LRR class), putative	I (2,5up)	30,2	23,5	71,1	64,5	36,1	52,5	36,4	45,6	35,6	31,8	2,5	up	1,6	up	1,5	up	1,3	up
248964_at	AT5G45340	CYP707A3 (cytochrome P450, family 707, subfamily A, polypeptide 3)	I (2,5up)	171,2	32,9	1215,6	1513,6	292,8	400,4	212,6	341,3	93,0	97,9	13,4	up	3,4	up	2,7	up	1,1	down
254211_at	AT4G23570	SGT1A (Suppressor of G2 (Two) 1A)	I (2,5up)	105,7	164,2	674,5	484,3	218,7	274,0	94,0	176,3	155,3	213,4	4,3	up	1,8	up	1,0	up	1,4	up
262905_at	AT1G59730	ATH7 (thioredoxin H-type 7)	I (2,5up)	175,8	31,4	199,1	251,9	77,7	21,4	77,0	19,7	192,0	24,4	2,2	up	2,1	down	2,1	down	1,0	up
248979_at	AT5G45080	ATPP2-A6 (Phloem protein 2-A6)	I (2,5up)	92,1	88,3	762,1	307,4	124,5	180,7	145,9	129,2	65,3	95,2	5,9	up	1,7	up	1,5	up	1,1	down
247297_at	AT5G64100	peroxidase, putative	I (2,5up)	5912,4	5064,7	14403,9	10577,6	3185,1	7341,3	3141,5	3831,3	372,4	1169,4	2,3	up	1,0	down	1,6	down	7,1	down
247327_at	AT5G64120	peroxidase, putative	I (2,5up)	807,7	510,3	3803,4	3997,9	647,3	2569,6	1064,4	1029,8	96,1	337,0	5,9	up	2,4	up	1,6	up	3,0	down
262517_at	AT1G17180	ATGSTU25 (Arabidopsis thaliana Glutathione S-transferase (class tau) 25)	I (2,5up)	817,8	140,9	1489,8	606,1	594,8	335,1	850,9	261,8	424,4	266,7	2,2	up	1,0	down	1,2	up	1,4	down
266561_at	AT2G23960	defense-related protein, putative	I (2,5up)	18,0	10,8	107,5	13,8	22,0	13,5	15,1	9,1	13,6	11,2	4,2	up	1,2	up	1,2	down	1,2	down

Genes transiently modulated during biotrophy and involved in perception of stimuli and in resulting responses (Cluster IV, FunCatDB 32.01, 34.11 and 36.20 from Table S6)

264402_at	AT2G25140	CLPB-M/CLPB4/HSP98.7 (HEAT SHOCK PROTEIN 98.7)	IV (biotrophy down)	80,4	62,8	73,7	101,1	41,6	36,6	23,7	22,9	108,2	55,6	1,2	up	1,8	down	3,1	down	1,1	up
259537_at	AT1G12370	PHR1 (PHOTOLYASE 1)	IV (biotrophy down)	126,0	43,4	61,4	94,4	33,2	25,5	17,1	14,1	32,2	30,9	1,1	down	2,9	down	5,4	down	2,7	down
266555_at	AT2G46270	GBF3 (G-BOX BINDING FACTOR 3)	IV (biotrophy down)	188,0	150,5	111,8	181,6	90,7	62,3	34,5	31,5	58,7	105,8	1,2	down	2,2	down	5,1	down	2,1	down
251827_at	AT3G55120	A11/CFI/TT5 (TRANSPARENT TESTA 5)	IV (biotrophy down)	451,3	519,4	107,0	1004,7	67,6	119,4	91,4	42,2	78,0	298,2	1,1	up	5,2	down	7,3	down	2,6	down
262826_at	AT1G13080	CYP71B2 (CYTOCHROME P450 71B2)	IV (biotrophy down)	268,9	514,6	126,2	90,7	33,6	58,2	16,2	34,2	28,5	90,0	3,6	down	8,5	down	15,6	down	6,6	down
252123_at	AT3G51240	F3H (TRANSPARENT TESTA 6)	IV (biotrophy down)	1543,2	834,2	262,7	1170,1	71,8	131,4	59,5	62,0	92,7	209,4	1,7	down	11,7	down	19,6	down	7,9	down
260956_at	AT1G06040	STO (SALT TOLERANCE)	IV (biotrophy down)	2910,5	2224,8	1903,5	2954,2	779,9	1131,0	168,3	290,5	723,1	979,0	1,1	down	2,7	down	11,2	down	3,0	down
247025_at	AT5G67030	ABA1 (ABA DEFICIENT 1)	IV (biotrophy down)	223,7	282,4	82,6	229,6	45,5	90,2	11,2	15,8	110,5	107,0	1,6	down	3,7	down	18,8	down	2,3	down
263083_at	AT2G27190	PAP1 (PURPLE ACID PHOSPHATASE 1)	IV (biotrophy down)	1547,3	1307,0	574,5	374,3	526,9	278,9	510,6	242,4	799,1	713,0	3,0	down	3,5	down	3,8	down	1,9	down
258321_at	AT3G22840	ELIP1 (EARLY LIGHT-INDUCABLE PROTEIN)	IV (biotrophy down)	2463,0	1124,6	1493,0	5031,6	231,9	319,6	30,3	20,9	366,1	381,8	1,8	up	6,5	down	70,1	down	4,8	down
248337_at	AT5G52310	COR78 (COLD REGULATED 78)	IV (biotrophy down)	173,7	1231,5	91,4	158,5	46,1	400,0	37,5	21,5	49,0	603,2	5,6	down	3,1	down	23,8	down	2,2	down
264211_at	AT1G22770	GI (GIGANTEA)	IV (biotrophy down)	989,4	1992,7	622,1	1030,5	652,2	897,7	105,8	112,0	705,2	811,1	1,8	down	1,9	down	13,7	down	2,0	down
247222_at	AT5G64840	ATGCN5 (Arabidopsis thaliana general control non-repressible 5)	IV (biotrophy down)	271,5	350,9	125,1	356,0	125,0	156,4	61,0	94,8	207,6	145,6	1,3	down	2,2	down	4,0	down	1,8	down
253263_at	AT4G34000	ABF3/DPBF5 (ABSCISIC ACID RESPONSIVE ELEMENTS-BINDING FACTOR 3)	IV (biotrophy down)	259,0	149,3	85,0	94,6	81,3	51,1	32,9	33,9	99,0	80,2	2,3	down	3,1	down	6,1	down	2,3	down
263433_at	AT2G22240	inositol-3-phosphate synthase isozyme 2 / myo-inositol-1-phosphate synthase 2 / MI-1-P synthase 2 / IPS 2	IV (biotrophy down)	269,1	77,4	112,8	136,4	34,1	47,1	29,3	15,7	48,3	68,8	1,4	down	4,3	down	7,7	down	3,0	down
255877_at	AT2G40460	proton-dependent oligopeptide transport (POT) family protein	IV (biotrophy down)	185,0	287,6	46,2	262,5	17,1	75,3	16,7	17,7	74,3	127,1	1,5	down	5,1	down	13,7	down	2,3	down
259768_at	AT1G29390	COR314-TM2 (cold regulated 314 thylakoid membrane 2)	IV (biotrophy down)	39,5	51,5	27,4	81,1	17,2	46,1	20,4	20,9	41,7	47,7	1,2	up	1,4	down	2,2	down	1,0	down
255645_at	AT4G00880	auxin-responsive family protein	IV (biotrophy down)	732,4	548,6	391,5	302,0	142,8	176,0	152,3	102,0	306,6	311,9	1,8	down	4,0	down	5,0	down	2,1	down
266720_s_at	AT2G46670; AT2G46790	pseudo-response regulator ; APRR9 (PSEUDO-RESPONSE REGULATOR 9)	IV (biotrophy down)	513,4	231,3	329,4	548,3	165,3	104,1	25,1	13,9	176,3	168,4	1,2	up	2,8	down	19,1	down	2,2	down
258860_at	AT3G02050	KUP3 (K+ uptake permease 3)	IV (biotrophy down)	45,4	68,0	19,9	23,4	20,4	27,9	17,3	19,1	44,5	29,8	2,6	down	2,3	down	3,1	down	1,5	down
261751_at	AT1G76080	ATCDSP32/CDSP32 (CHLOROPLASTIC DROUGHT-INDUCED STRESS PROTEIN OF 32 KD)	IV (biotrophy down)	116,6	183,5	190,3	188,8	194,7	245,2	40,4	40,3	176,6	134,0	1,3	up	1,5	up	3,7	down	1,0	up
256216_at	AT1G56340	CRT1 (CALRETICULIN 1)	IV (biotrophy down)	2116,9	1291,4	1124,2	845,9	861,3	759,0	1089,6	1266,6	2656,3	2117,9	1,7	down	2,1	down	1,4	down	1,4	up
267612_at	AT2G26690	nitrate transporter (NTP2)	IV (biotrophy down)	272,7	392,5	44,1	169,0	18,4	64,2	35,0	42,3	198,1	145,3	3,1	down	8,1	down	8,6	down	1,9	down
266327_at	AT2G46680	ATHB-7 (ARABIDOPSIS THALIANA HOMEOBOX 7)	IV (biotrophy down)	549,0	1656,9	126,5	886,9	211,6	1187,5	230,2	694,9	926,8	1194,4	2,2	down	1,6	down	2,4	down	1,0	down
258158_at	AT3G17790	ATACP5 (acid phosphatase 5)	IV (biotrophy down)	3991,1	3931,3	1257,3	1527,0	2309,0	1044,2	1916,2	1301,5	3641,0	3565,4	2,8	down	2,4	down	2,5	down	1,1	down
254166_at	AT4G24190	SHD (SHEPHERD)	IV (biotrophy down)	3978,4	2129,9	1769,0	1377,7	974,0	1234,8	974,3	1178,3	2915,2	3076,4	1,9	down	2,8	down	2,8	down	1,0	down
265480_at	AT2G15970	COR413-PM1 (cold regulated 413 plasma membrane 1)	IV (biotrophy down)	949,2	2299,7	375,6	684,2	253,3	652,0	170,8	166,8	108,2	718,8	3,1	down	3,6	down	9,6	down	3,9	down
263320_at	AT2G47180	ATGOLS1 (ARABIDOPSIS THALIANA GALACTINOL SYNTHASE 1);	IV (biotrophy down)	1308,3	718,3	476,9	1931,0	254,8	342,5	49,8	85,4	717,3	502,2	1,2	up	3,4	down	15,0	down	1,7	down
256454_at	AT1G75280	isoflavone reductase, putative	IV (biotrophy down)	3185,0	3653,5	2070,4	3471,1	1210,0	1883,6	2011,7	324,9	2530,2	2338,4	1,2	down	2,2	down	2,9	down	1,4	down
264260_at	AT1G09210	calreticulin 2 (CRT2)	IV (biotrophy down)	1552,2	934,5	662,3	502,5	372,7	338,2	385,4	396,4	1125,3	796,2	2,1	down	3,5	down	3,2	down	1,3	down
246481_s_at	AT5G15960; AT5G15970	[AT5G15960, KIN1];[AT5G15970, KIN2 (COLD-RESPONSIVE 6.6)]	IV (biotrophy down)	6632,9	6060,0	3277,6	2240,4	1983,3	2996,7	1169,9	717,6	784,6	3624,8	2,3	down	2,5	down	6,7	down	2,9	down
267336_at	AT2G19310	similar to HSP18.2 (HEAT SHOCK PROTEIN 18.2); similar to 18.5 kDa class I heat shock protein (HSP 18.5) (GB:P05478)	IV (biotrophy down)	61,9	209,4	46,6	282,6	50,5	92,2	14,6	49,1	68,7	59,0	1,2	up	1,9	down	4,3	down	2,1	down

Genes involved in SA biosynthesis

246272_at	AT4G37150	esterase, MES9	IV (biotrophy down)	145,1	124,4	51,9	277,1	17,6	81,0	11,1	32,6	35,2	145,3	1,2	up	2,7	down	6,2	down	1,5	down
259286_at	AT3G11480	BSMT1; S-adenosylmethionine-dependent methyltransferase		4,6	4,6	4,6	4,6	4,6	4,6	4,6	4,6	4,6	4,6								
267128_at	AT2G23620	esterase		56,3	37,9	38,4	45,2	13,4	67,2	10,9	11,4	10,7	11,0								
261428_at	AT1G18870	ICS2; isochorismate synthase	VIII (Interaction Down)	336,9	41,2	32,4	9,8	8,1	5,7	25,2	8,1	9,8	13,8	9,0	down	27,4	down	11,3	down	16,0	down
260563_at	AT2G43840	UDP-glucoronosyl/UDP-glucosyl transferase family protein		13,6	6,2	7,0	7,5	9,5	7,5	24,9	5,9	19,6	7,7								
267126_s_at	AT2G23600	[AT2G23600, ACL (ACETONE-CYANOHYDRIN LYASE); hydrolase];[AT2G23590, hydrolase, alpha/beta fold family protein]		189,4	629,4	339,0	637,8	292,6	798,1	351,3	802,6	410,7	459,0								
260567_at	AT2G43820	GT/UGT74F2 (UDP-GLUCOSYLTRANSFERASE 74F2)	I (penetration up)	84,8	218,3	555,8	323,0	271,8	236,8	293,5	88,5	103,8	148,3	2,9	up	1,7	up	1,3	up	1,2	down
267123_at	AT2G23560	hydrolase, alpha/beta fold family protein		42,1	18,9	18,9	18,9	18,9	12,3	29,0	9,0	43,2	18,9								
262177_at	AT1G74710	ICS1 (ISOCHORISMATE SYNTHASEI)	VII (Interaction Up)	30,9	13,0	30,0	24,0	48,9	31,0	188,0	72,4	408,8	188,5	1,2	up	1,8	up	5,9	up	13,6	up

SA markers

266385_at	AT2G14610	PR1		5,3	5,1	5,3	5,3	5,3	5,3	5,3	5,6	5,3	5,3
251625_at	AT3G57260	PR2/BGL2		4,6	4,6	4,6	4,6	4,6	4,6	4,6	4,6	23,0	6,8
259925_at	AT1G75040	PR5/Thaumatulin		5,8	5,4	5,4	5,8	5,8	6,2	5,8	5,8	64,0	25,2

Genes involved in JA biosynthesis

257644_at	AT3G25780	AOC3 (ALLENE OXIDE CYCLASE 3)		160,4	58,1	311,6	323,6	280,2	291,8	289,2	246,1	274,1	212,0								
265843_at	AT2G35690	ACX5 (ACYL-COA OXIDASE 5); acyl-CoA oxidase		22,3	47,9	41,6	27,2	46,1	73,6	46,5	65,2	72,7	61,2								
260789_s_at	AT3G06690	[AT3G06690, acyl-CoA oxidase family];[AT1G06290, ACX3 (ACYL-COA OXIDASE 3); acyl-CoA oxidase]		932,2	925,3	1250,6	901,0	1171,7	1293,1	1294,1	1203,9	1310,6	1283,6								
257641_s_at	AT3G25760	[AT3G25760, AOC1 (ALLENE OXIDE CYCLASE 1)];[AT3G25770, AOC2 (ALLENE OXIDE CYCLASE 2)]	II (2,5 down)	146,0	177,5	57,2	41,6	517,8	386,1	237,8	121,8	79,0	105,2	3,3	down	2,8	up	1,1	up	1,8	down
245249_at	AT4G16760	ACX1 (ACYL-COA OXIDASE 1)	VII (Interaction Up)	624,3	762,9	917,6	1300,4	2578,8	2151,4	2402,5	1823,1	3042,1	1962,6	1,6	up	3,4	up	3,0	up	3,6	up
249208_at	AT5G42650	AOS (ALLENE OXIDE SYNTHASE); hydro-lyase/ oxygen binding	VII (Interaction Up)	74,7	47,6	173,8	180,5	255,9	172,9	218,5	88,6	145,4	95,4	2,9	up	3,5	up	2,5	up	2,0	up
246304_at	AT3G51840	ACX4 (ACYL-COA OXIDASE 4); oxidoreductase		822,4	2173,0	760,4	1271,3	985,0	1570,9	1215,1	1899,1	1317,6	2838,2								
263786_at	AT2G46370	JAR1 (JASMONATE RESISTANT 1)	VIII (Interaction Down)	1188,0	1004,4	564,0	818,6	213,8	327,0	325,9	205,2	339,1	376,8	1,6	down	4,1	down	4,1	down	3,1	down
265530_at	AT2G06050	OPR3 (OPDA-REDUCTASE 3); 12-oxophytodienoate reductase	VII (Interaction Up)	443,5	460,1	401,1	710,3	986,9	994,4	582,4	598,3	772,5	704,8	1,2	up	2,2	up	1,3	up	1,6	up
261150_at	AT1G19640	JMT (JASMONIC ACID CARBOXYL METHYLTRANSFERASE); jasmonate O-methyltransferase		4,6	4,6	4,6	4,6	4,6	4,6	4,6	4,6	4,6	4,6								
247176_at	AT5G65110	ACX2 (ACYL-COA OXIDASE 2); acyl-CoA oxidase		309,3	1030,5	490,1	555,9	802,3	1021,8	834,7	1140,7	1231,8	1353,0								
266883_at	AT2G44810	DAD1 (DEFECTIVE ANTHER DEHISCENCE 1); triacylglycerol lipase		7,6	7,7	8,6	8,6	8,6	8,6	8,6	8,7	8,6	8,6								
259518_at	AT1G20510	OPCL1 (OPC-8:0 COA LIGASE1); 4-coumarate-CoA ligase	VII (Interaction Up)	184,0	122,8	441,3	407,2	588,5	521,4	599,0	331,7	466,1	365,3	2,8	up	3,6	up	3,0	up	2,7	up
252618_at	AT3G45140	LOX2 (LIPOXYGENASE 2)		4,6	7,5	4,6	4,7	11,3	4,9	4,6	4,6	4,6	4,6								
261278_at	AT1G05800	lipase class 3 family protein		4,6	4,6	4,6	4,6	4,6	4,6	4,6	4,6	4,6	4,6								

Transcription factors involved in JA and ET signalling pathway

261713_at	AT1G32640	ATMYC2 (JASMONATE INSENSITIVE 1); DNA binding / transcription factor		384,4	716,8	307,3	560,9	908,6	557,2	557,3	527,2	719,0	489,6								
257927_at	AT3G23240	ATERF1/ERF1 (ETHYLENE RESPONSE FACTOR 1); DNA binding / transcription activator/ transcription factor	VII (Interaction Up)	52,8	65,8	254,5	214,3	716,8	631,5	1101,4	525,1	379,7	500,6	4,0	up	11,4	up	13,7	up	7,4	up

JAZ genes

246161_at	AT5G20900	JAZ12/TIFY3B (JASMONATE-ZIM-DOMAIN PROTEIN 12)		876,2	1531,1	588,7	1224,6	588,7	1063,9	640,6	1007,9	937,2	997,0								
266901_at	AT2G34600	JAZ7/TIFY5B (JASMONATE-ZIM-DOMAIN PROTEIN 7)	VII (Interaction Up)	19,6	68,6	15,1	32,3	229,6	65,4	110,4	32,6	59,3	75,1	1,9	down	3,3	up	1,6	up	1,5	up
260429_at	AT1G72450	JAZ6/TIFY11B (JASMONATE-ZIM-DOMAIN PROTEIN 6)	VII (Interaction Up)	457,6	287,6	1425,9	1754,5	2228,1	1945,8	2110,4	1654,8	1149,2	1012,9	4,3	up	5,6	up	5,1	up	2,9	up
250292_at	AT5G13220	JAS1/JAZ10/TIFY9 (JASMONATE-ZIM-DOMAIN PROTEIN 10)	VII (Interaction Up)	21,2	29,6	142,2	162,9	1074,8	504,9	622,1	302,8	396,9	273,9	6,0	up	31,1	up	18,2	up	13,2	up
258189_at	AT3G17860	JAI3/JAZ3/TIFY6B (JASMONATE-ZIM-DOMAIN PROTEIN 3)		776,1	402,2	866,9	526,6	1220,8	482,3	799,8	513,0	365,0	297,7								
261033_at	AT1G17380	JAZ5/TIFY11A (JASMONATE-ZIM-DOMAIN PROTEIN 5)	VII (Interaction Up)	53,6	27,0	285,6	318,2	915,4	710,8	503,4	477,6	587,5	410,8	7,5	up	20,2	up	12,2	up	12,4	up
256159_at	AT1G30135	JAZ8/TIFY5A (JASMONATE-ZIM-DOMAIN PROTEIN 8)	III (biotrophy up)	8,0	10,5	66,1	25,1	1138,1	276,4	1486,0	122,8	333,5	158,2	4,9	up	76,2	up	86,7	up	26,5	up
256017_at	AT1G19180	JAZ1/TIFY10A (JASMONATE-ZIM-DOMAIN PROTEIN 1); protein binding	VII (Interaction Up)	2154,7	2357,7	7269,2	7970,4	8872,5	8121,1	8151,1	7244,3	8196,7	6640,7	3,4	up	3,8	up	3,4	up	3,3	up
260205_at	AT1G70700	JAZ9/TIFY7 (JASMONATE-ZIM-DOMAIN PROTEIN 9)		5,7	5,3	4,6	6,5	44,1	11,9	37,0	9,3	5,8	6,1								
262171_at	AT1G74950	JAZ2/TIFY10B (JASMONATE-ZIM-DOMAIN PROTEIN 2)	VII (Interaction Up)	394,6	521,9	429,1	568,9	1663,6	904,7	1378,2	738,5	1261,4	783,5	1,1	up	2,8	up	2,3	up	2,2	up

Genes involved in ethylen biosynthesis

253999_at	AT4G26200	ACS7 (1-Amino-cyclopropane-1-carboxylate synthase 7); 1-aminocyclopropane-1-carboxylate synthase	I (penetration up)	13,1	16,0	139,9	100,7	9,0	50,2	10,6	39,7	14,1	38,9	8,2	up	2,0	up	1,7	up	1,8	up
265948_at	AT2G19590	ACO1 (ACC OXIDASE 1); 1-aminocyclopropane-1-carboxylate oxidase	I (penetration up)	959,0	84,8	4063,8	890,0	1756,0	914,3	1220,0	763,2	354,7	466,2	4,7	up	2,6	up	1,9	up	1,3	down
247159_at	AT5G65800	ACS5 (ACC SYNTHASE 5); 1-aminocyclopropane-1-carboxylate synthase		4,6	4,6	4,6	4,6	4,6	4,6	4,6	4,6	4,6	4,6								
255177_at	AT4G08040	ACS11 (1-Amino-cyclopropane-1-carboxylate synthase 11); 1-aminocyclopropane-1-carboxylate synthase		4,6	4,6	5,3	4,6	5,1	4,6	4,6	4,6	4,6	4,8								
259439_at	AT1G01480	ACS2 (1-Amino-cyclopropane-1-carboxylate synthase 2)	VII (Interaction Up)	6,2	9,2	22,6	23,8	204,5	121,3	190,6	306,7	945,0	1033,3	3,0	up	21,2	up	32,3	up	128,5	up
266830_at	AT2G22810	ACS4 (1-AMINOCYCLOPROPANE-1-CARBOXYLATE SYNTHASE 4); 1-aminocyclopropane-1-carboxylate synthase		4,6	4,6	4,6	4,6	4,6	4,6	4,6	4,6	4,6	4,6								
254926_at	AT4G11280	ACS6 (1-AMINOCYCLOPROPANE-1-CARBOXYLIC ACID (ACC) SYNTHASE 6)	VII (Interaction Up)	85,7	20,3	1327,2	721,0	2590,6	2032,8	2544,1	1237,9	1763,0	1343,9	19,3	up	43,6	up	35,7	up	29,3	up
246172_s_at	AT3G61510	[AT3G61510, ACS1 (ACC SYNTHASE 1); 1-aminocyclopropane-1-carboxylate synthase];[AT5G28360, ACS3 (1-AMINOCYCLOPROPANE-1-CARBOXYLATE SYNTHASE LIKE PSEUDOGENE); 1-aminocyclopropane-1-carboxylate synthase]		5,0	4,9	4,9	4,9	4,9	4,9	4,9	5,6	4,9	5,2								
252279_at	AT3G49700	ETO3 (ETHYLENE OVERPRODUCING 3); 1-aminocyclopropane-1-carboxylate synthase		4,6	4,6	4,6	7,0	4,6	4,6	4,6	4,6	5,9	4,6								
260637_at	AT1G62380	ACO2 (ACC OXIDASE 2)		1094,2	474,8	1285,8	643,4	1207,3	515,5	1760,7	322,7	1922,6	612,8								
253066_at	AT4G37770	ACS8 (1-Amino-cyclopropane-1-carboxylate synthase 8)		5,0	4,9	5,3	8,9	5,3	7,6	4,9	9,3	5,5	5,3								
265194_at	AT1G05010	ACO4; EFE (ETHYLENE FORMING ENZYME)	VII (Interaction Up)	1187,7	979,4	2065,2	2219,8	2405,3	2154,5	3307,1	2982,2	1803,1	1966,2	2,0	up	2,1	up	2,9	up	1,7	up

ET and JA markers

257365_x_at	AT2G26020	PDF1,2B		4,6	4,8	4,8	4,8	4,8	4,8	4,8	4,8	4,8	4,8								
256243_at	AT3G12500	PR3/CHI	VII (Interaction Up)	7246,5	3809,1	5714,8	4345,8	12585,5	8200,3	13710,2	10157,7	16473,7	13067,7	1,1	down	1,9	up	2,2	up	2,7	up
258791_at	AT3G04720	PR4/hevein	VII (Interaction Up)	5405,5	4799,5	4749,2	4480,0	13934,2	9050,9	15557,5	8526,2	15899,3	13177,2	1,1	down	2,3	up	2,4	up	2,8	up

VQ Motif-containing genes / Plant Physiol. 2012, 159:810-825

259552_at	AT1G21320	VQ2	VII (Interaction Up)	13,8	6,0	87,7	20,1	304,4	62,7	262,1	32,9	86,9	66,3	5,5	up	18,6	up	14,9	up	7,8	up
245666_at	AT1G28280	VQ4	VII (Interaction Up)	224,8	335,2	599,4	467,0	736,3	743,0	715,0	897,8	799,6	995,7	1,9	up	2,6	up	2,9	up	3,2	up
261697_at	AT1G32610	VQ6		5,8	7,7	5,6	5,7	5,7	7,7	8,6	8,0	5,7	5,8								
260261_at	AT1G68450	VQ8	VII (Interaction Up)	68,5	132,2	617,9	672,0	1325,3	1314,0	1242,1	1069,5	1662,0	1180,3	6,4	up	13,2	up	11,5	up	14,2	up
260807_at	AT1G78310	VQ9		74,2	221,0	129,5	146,3	156,0	177,0	130,6	182,9	92,6	151,4								
260804_at	AT1G78410	VQ10	VII (Interaction Up)	7,4	7,1	240,9	247,4	264,4	323,1	294,1	421,9	480,6	379,5	33,7	up	40,5	up	49,4	up	59,4	up
260276_at	AT1G80450	VQ11	VII (Interaction Up)	7,8	18,7	13,3	13,2	20,0	33,5	27,4	29,5	45,4	54,8	1,0	up	2,0	up	2,1	up	3,8	up
266800_at	AT2G22880	VQ12	VII (Interaction Up)	17,0	72,5	1558,6	1313,9	1504,4	1647,0	1123,8	1489,0	497,6	1235,9	32,1	up	35,2	up	29,2	up	19,4	up
266538_at	AT2G35230	VQ14/IKU1		31,1	43,8	23,5	36,7	22,0	42,7	31,9	47,7	27,7	37,8								
267069_at	AT2G41010	VQ15/CAMBP25	I (penetration up)	48,9	147,5	356,1	290,8	190,2	198,8	106,3	130,1	62,1	174,1	3,3	up	2,0	up	1,2	up	1,2	up
267084_at	AT2G41180	VQ16/SIB2	VII (Interaction Up)	8,1	9,3	82,3	67,7	347,1	175,6	492,8	186,7	240,4	157,9	8,6	up	30,0	up	39,1	up	22,9	up
257348_at	AT2G42140	VQ17	VII (Interaction Up)	61,6	111,1	72,6	102,2	196,6	160,6	268,2	201,5	184,5	189,6	1,0	up	2,1	up	2,7	up	2,2	up
257105_at	AT3G15300	VQ19		198,1	222,8	123,3	215,1	78,6	151,0	168,8	133,0	181,4	168,7								
257726_at	AT3G18360	VQ20		5,2	5,2	5,1	5,2	5,2	5,9	5,2	6,5	5,2	5,9								
257751_at	AT3G18690	VQ21/MKS1	VII (Interaction Up)	63,8	116,0	676,1	304,7	706,3	486,9	465,7	446,9	294,2	322,9	5,5	up	6,6	up	5,1	up	3,4	up
256793_at	AT3G22160	VQ22	VII (Interaction Up)	110,9	152,0	404,5	473,6	608,0	610,4	608,4	533,1	485,9	529,7	3,3	up	4,6	up	4,3	up	3,9	up
246293_at	AT3G56710	VQ23/SIB1	VII (Interaction Up)	10,1	18,2	215,7	140,9	724,5	617,1	910,7	841,1	997,1	1363,3	12,6	up	47,5	up	62,0	up	83,6	up
246289_at	AT3G56880	VQ24	I (penetration up)	280,2	865,2	1527,6	1098,7	777,0	982,5	441,6	718,6	288,0	1005,3	2,3	up	1,5	up	1,0	up	1,1	up
251617_at	AT3G58000	VQ25		4,7	4,7	4,7	4,7	4,7	4,7	4,7	5,2	4,7	4,7								
245363_at	AT4G15120	VQ27	VII (Interaction Up)	12,2	14,2	331,2	185,6	633,8	498,3	411,9	504,4	341,7	550,9	19,6	up	43,0	up	34,8	up	33,9	up
254524_at	AT4G20000	VQ28	VII (Interaction Up)	68,4	25,3	307,5	357,8	83,0	170,9	145,8	178,5	77,2	121,7	7,1	up	2,7	up	3,5	up	2,1	up
253060_at	AT4G37710	VQ29	VII (Interaction Up)	4,6	4,6	3250,2	1450,9	8331,1	6313,5	3588,2	7228,6	3461,2	5330,2	509,6	up	1587,4	up	1172,5	up	952,9	up
252905_at	AT4G39720	VQ30	VII (Interaction Up)	50,6	31,3	85,4	57,5	187,9	79,3	101,6	97,5	99,5	140,9	1,7	up	3,3	up	2,4	up	2,9	up
250535_at	AT5G08480	VQ31		5,6	5,6	5,6	4,8	5,5	5,6	6,6	9,3	5,6	6,1								
248868_at	AT5G46780	VQ32	VII (Interaction Up)	303,2	855,5	2245,0	1652,8	1839,0	2009,3	1606,9	2612,5	1277,4	1748,4	3,4	up	3,3	up	3,6	up	2,6	up
248230_at	AT5G53830	VQ33		76,4	43,3	86,1	58,3	115,6	81,3	120,1	77,8	115,0	121,9								
247230_at	AT5G65170	VQ34		6,8	7,5	4,6	5,7	5,7	6,2	5,7	5,6	5,7	5,6								

No probe corresponded to AT1G17147, AT1G21326, AT1G32585, AT1G35830, AT2G33780, AT2G44340, AT3G60090 .

DC1 domain-containing protein genes

259454_at	AT1G44050	DC1 domain-containing protein	I (penetration up)	19,4	10,7	149,6	34,0	35,1	19,8	23,0	15,1	12,9	11,4	6,1	up	1,8	up	1,3	up	1,2	down
255619_s_at	AT5G59920, AT4G01350	[AT5G59920, ULI3 (UV-B light insensitive 3)];[AT4G01350, DC1 domain-containing protein]	VI (Switch down)	375,9	352,6	487,4	512,9	424,3	485,4	300,7	373,9	79,3	204,6	1,4	up	1,2	up	1,1	down	2,6	down
247674_at	AT5G59930	DC1 domain-containing protein / UV-B light-insensitive protein, putative	VII (Interaction Up)	24,1	8,1	105,0	33,1	71,4	55,9	41,6	31,1	25,4	25,8	4,3	up	3,9	up	2,3	up	1,6	up
259369_s_at	AT3G43890, AT1G69150	[AT3G43890, DC1 domain-containing protein];[AT1G69150, DC1 domain-containing protein]	VII (Interaction Up)	11,4	7,9	76,7	34,5	72,4	61,8	34,8	42,2	34,1	45,6	5,8	up	7,0	up	4,0	up	4,1	up
254749_at	AT4G13130	DC1 domain-containing protein	VIII (Interaction Down)	123,6	99,2	66,2	78,5	40,4	41,3	47,2	30,0	15,4	35,9	1,5	down	2,7	down	2,9	down	4,3	down
249364_at	AT5G40590	DC1 domain-containing protein	I (penetration up)	383,5	66,2	5079,6	3339,1	946,3	1061,3	1388,9	386,5	182,6	274,1	18,7	up	4,5	up	3,9	up	1,0	up
267384_at	AT2G44370	DC1 domain-containing protein	I (penetration up)	28,5	81,0	2328,1	2113,5	263,5	765,6	165,2	287,0	7,4	47,2	40,6	up	9,4	up	4,1	up	2,0	down
259260_at	AT3G11370	DC1 domain-containing protein	I (penetration up)	12,9	6,2	100,3	7,1	7,1	5,0	7,1	7,1	7,1	6,5	5,6	up	1,6	down	1,4	down	1,4	down
266247_at	AT2G27660	DC1 domain-containing protein	I (penetration up)	62,1	76,2	806,4	704,0	222,9	334,7	171,9	182,4	86,1	82,1	10,9	up	4,0	up	2,6	up	1,2	up
267240_at	AT2G02680	DC1 domain-containing protein	I (penetration up)	98,7	39,1	571,4	131,4	44,6	55,1	42,4	38,8	9,5	10,2	5,1	up	1,4	down	1,7	down	7,0	down
247675_at	AT5G59940	DC1 domain-containing protein / UV-B light-insensitive protein, putative	VII (Interaction Up)	55,1	86,0	190,4	141,9	267,0	315,2	205,1	297,4	125,2	199,8	2,4	up	4,1	up	3,6	up	2,3	up
267385_at	AT2G44380	DC1 domain-containing protein	I (penetration up)	179,4	389,2	1566,4	1392,6	150,3	430,0	121,8	243,9	21,6	67,1	5,2	up	1,0	up	1,6	down	6,4	down
264282_at	AT1G61840	DC1 domain-containing protein	II (penetration down)	5,3	4,6	236,0	23,6	4,6	4,6	4,6	4,6	4,6	4,6	26,4	up	1,1	down	1,1	down	1,1	down
249097_at	AT5G43520	DC1 domain-containing protein	I (penetration up)	232,1	379,0	6426,6	5874,9	725,2	2477,8	737,8	1228,2	89,1	330,2	20,1	up	5,2	up	3,2	up	1,5	down
