# Supplementary material for: Transcriptome dynamic of Arabidopsis roots infected with Phytophthora parasitica identifies VQ29, a gene induced during the penetration and involved in the restriction of infection
Source: PLoS One. 2017 Dec 27;12(12):e0190341. doi: 10.1371/journal.pone.0190341 (PMC5744986; doi:10.1371/journal.pone.0190341)
Supplement: S8 Table — FC, Fold Change. (PDF) [file pone.0190341.s012.pdf]

S8 Table: Microarray data of genes selected for infection essay of knockout (Ko) lines and described Table 1.

FC, Fold Change.

				Genes expression signals during *A. thaliana* /*P. parasitica* interaction										**Genes expression fold changes compared to NI control**							
				NI		2.5-hai		6-hai		10.5-hai		30-hai		**2.5-hai / Ctrl**		**6-hai / Ctrl**		**10.5-hai / Ctrl**		**30-hai / Ctrl**	
**Affy ID**	**AGI code**	**Gene description**	**Clusters**	rep. 1	rep. 2	rep. 1	rep. 2	rep. 1	rep. 2	rep. 1	rep. 2	rep. 1	rep. 2	FC	direction	FC	direction	FC	direction	FC	direction
262743_at	AT1G29020	calcium-binding EF hand family protein	I (2,5up)	77,2	21,6	955,9	99,2	84,3	24,2	33,1	22,0	17,0	28,9	10,7	up	1,1	up	1,8	down	2,2	down
249097_at	AT5G43520	DC1 domain-containing protein	I (2,5up)	232,1	379,0	6426,6	5874,9	725,2	2477,8	737,8	1228,2	89,1	330,2	20,1	up	5,2	up	3,2	up	1,5	down
265460_at	AT2G46600	calcium-binding protein, putative	I (2,5up)	1096,6	503,8	1894,5	1643,8	1110,8	1160,8	1165,2	916,6	1079,6	1047,4	2,2	up	1,4	up	1,3	up	1,3	up
261846_at	AT1G11540	unknown protein	I (2,5up)	99,9	54,7	1015,4	210,1	141,0	94,9	286,6	62,7	71,0	36,7	7,9	up	1,5	up	2,3	up	1,4	down
250302_at	AT5G11920	ATCWINV6 (6-&1-FRUCTAN EXOHYDROLASE); hydrolase, hydrolyzing O-glycosyl compounds / inulinase/ levanase	I (2,5up)	107,2	78,2	1909,0	686,7	692,5	662,0	553,2	403,7	350,4	347,7	14,0	up	7,3	up	5,2	up	3,8	up
249364_at	AT5G40590	DC1 domain-containing protein	I (2,5up)	383,5	66,2	5079,6	3339,1	946,3	1061,3	1388,9	386,5	182,6	274,1	18,7	up	4,5	up	3,9	up	1,0	up
267384_at	AT2G44370	DC1 domain-containing protein	I (2,5up)	28,5	81,0	2328,1	2113,5	263,5	765,6	165,2	287,0	7,4	47,2	40,6	up	9,4	up	4,1	up	2,0	down
250702_at	AT5G06730	peroxidase, putative	VII (Interaction Up)	54,6	44,4	93,5	100,2	1084,8	1076,5	2598,6	2611,4	6258,1	4773,2	2,0	up	21,8	up	52,6	up	111,4	up
253060_at	AT4G37710	VQ motif-containing protein	VII (Interaction Up)	4,6	4,6	3250,2	1450,9	8331,1	6313,5	3588,2	7228,6	3461,2	5330,2	509,6	up	1587,4	up	1172,5	up	952,9	up
254977_s_at	AT4G10520	[AT4G10530, subtilase family protein];[AT4G10520, subtilase family protein]	VII (Interaction Up)	4,8	5,1	137,8	15,7	612,1	393,0	405,6	232,0	318,9	151,2	15,4	up	100,7	up	63,9	up	47,1	up
